# Activation of allylic esters in an intramolecular vinylogous kinetic resolution reaction with synergistic magnesium catalysts

**DOI:** 10.1038/s41467-020-16486-0

**Published:** 2020-05-22

**Authors:** Dan Li, Yuling Yang, Minmin Zhang, Linqing Wang, Yingfan Xu, Dongxu Yang, Rui Wang

**Affiliations:** Key Laboratory of Preclinical Study for New Drugs of Gansu Province, Institute of Drug Design & Synthesis, School of Basic Medical Sciences, Lanzhou University, Lanzhou, 730000 China

**Keywords:** Asymmetric catalysis, Homogeneous catalysis, Synthetic chemistry methodology

## Abstract

Kinetic resolution (KR) of racemic starting materials is a powerful and practical alternative to prepare valuable enantiomerically enriched compounds. A magnesium-catalyzed kinetic resolution based on a designed intramolecular vinylogous Michael reaction is disclosed. Here we show a synergistic catalytic strategy based on the development of chiral ligands. Substrates containing linear allylic ester structures are designed and synthesized to construct key [6.6.5]-tricyclic chiral skeletons via this kinetic resolution process. Detailed mechanistic studies reveal a rational mechanism for the current intramolecular vinylogous KR reaction. The desired direct intramolecular asymmetric vinylogous Michael reaction of linear allylic esters is realized in high efficiency and enantioselectivity with the synergistic catalytic system.

## Abstract (web summary)

Kinetic resolution allows to obtain enantioenriched compounds from racemic mixtures. Here, the authors report a synergistic magnesium catalyst promoting kinetic resolution of an intramolecular vinylogous Michael reaction to access [6.6.5]-tricyclic chiral skeletons.

## Introduction

**Fig. 1:**
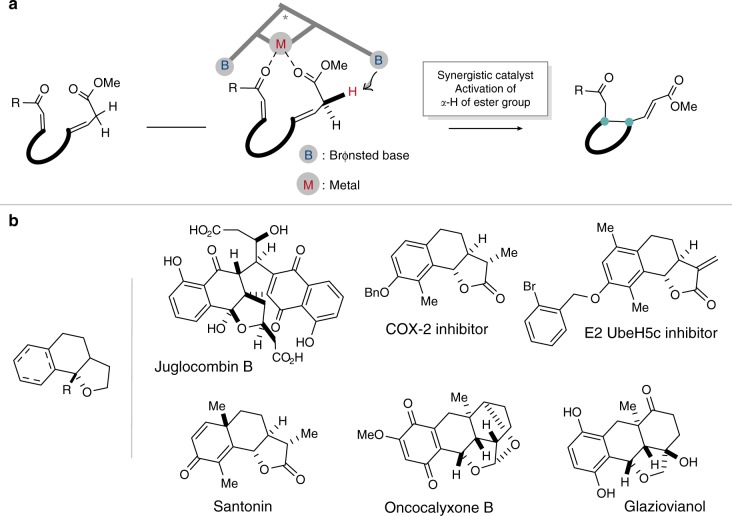
Reaction design and related compounds containing the key [6.6.5]-tricyclic skeletons. **a** Synergistic catalytic strategy for the direct intramolecular asymmetric vinylogous Michael reaction. **b** Related natural products and pharmaceutically active compounds containing the tricyclic skeletons.

Catalytic nonenzymatic kinetic resolution (KR) of racemic starting materials that mediates the selective reaction of one enantiomer has been recognized as a powerful and practical alternative to preparing valuable enantiomerically enriched compounds, and found wide applications in both academia and industry^1–4^. Most documented nonenzymatic KR reactions use intermolecular pathways, with the selection of one appropriate reactive partner to finish the desired KR process. In contrast, the development of intramolecular KR reactions have been relatively less investigated, as they require an ideal match between the designed substrates and small molecule catalysts^5–14^. Herein, we design an intramolecular vinylogous Michael reaction of linear allylic esters for a KR process to build chiral parallel [6.6.5]-tricyclic skeletons, which exist in many natural products and pharmaceutically active compounds, such as Juglocombin B, Glaziovianol, and some COX-2 and ubiquitin-connected enzymes inhibitors (Fig. 1)^15–19^. This reaction also represents one alternative to asymmetric dearomatizative pathways of 1-naphthols^20–26^.

Compared with other linear allylic carbonyl compounds, simple linear allylic esters are less reactive and less investigated in asymmetric reactions^27–29^. To date, there are still very few studies on the direct activation of linear allylic esters in catalytic asymmetric reactions. Moreover, the α-position of linear allylic esters might dominate the C-C bond formation process especially in the reaction with Michael acceptors^30–32^. In most cases, activated or modified allylic esters are often necessary to overcome the low reactivity of these types of substrates^33–39^. For example, in the widely used vinylogous Mukaiyama reaction, it is necessary to prepare the unstable dienolsilanes in a separate step^33–37^. Only until very recently, the Yin group reported direct asymmetric vinylogous aldol reactions of allylic esters using chiral copper catalysts and additive bases^40^. They also achieved the asymmetric alkynylogous aldol reaction by an optimized propargyl copper(I) catalytic method^41^. These reactions are highly efficient and ideal for the direct use of allylic esters as feedstock. However, the direct catalytic asymmetric intramolecular vinylogous reaction of allylic esters has not yet been achieved^42–44^. Herein, by developing a synergistic in situ generated magnesium catalytic strategy^45–55^, we successfully employ the vinylogous Michael reaction of linear allylic esters in a rationally designed intramolecular KR process (Fig. 1).

## Results

### Reaction optimization

**Fig. 2:**
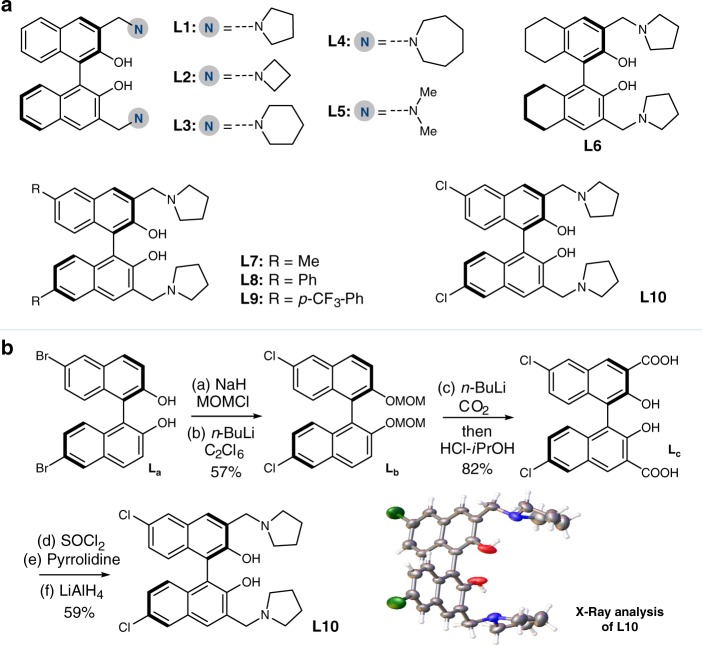
Selection and development of bifunctional chiral ligands and related synthetic methods. **a** Bifunctional chiral ligands screened in the optimization process. **b** Synthetic method of chiral ligand **L10** and related X-ray analysis.

Initially, we designed and synthesized the allylic ester **1a** for the intramolecular KR reaction. Bifunctional diols containing amine groups (Fig. 2) were selected as chiral ligands for sequencing process to the magnesium catalysts. The desired intramolecular vinylogous Michael reaction proceeded primarily from one enantiomer, and resulted in enantiomerically enriched parallel [6.6.5]-tricyclic skeletons (Table 1). Different tertiary amines-modified diol ligands were screened, and pyrrolidine-modified ligand **L1** had better resolution results compared with those of other tertiary amine groups (Table 1, entries 1-5). Further modification at the 6,6’-position of the BINOL skeletons led to the successful synthesis of a series of bifunctional chiral ligands (Fig. 2, **L7**-**L10**). These modifications dramatically affected the efficiency of the magnesium catalysts, and the introduction of chloride was identified as giving the best results for the intramolecular vinylogous KR reaction (Table 1, entry 10). The synthetic route for ligand **L10** is illustrated in Fig. 2.

**Table 1:** Optimization of the vinylogous KR reaction^a^.


Entry	**L**	Solvents	1a* (er)^b^	**2a** (er)^b^	s^c^
1	**L1**	CPME	47 (78.5:21.5)	30 (94.5:5.5)	31
2	**L2**	CPME	82 (55:45)	15 (80:20)	4
3	**L3**	CPME	82 (55:45)	15 (80:20)	25
4	**L4**	CPME	41 (87:13)	31 (92:8)	5
5	**L5**	CPME	72 (57.5:42.5)	11 (88:12)	8
6	**L6**	CPME	61 (51:8)	14 (55:45)	1
7	**L7**	CPME	50 (52.5:47.5)	40 (95:5)	33
8	**L8**	CPME	49 (58.5:21.5)	43 (83.5:16.5)	9
9	**L9**	CPME	59 (74.5:25.5)	32 (85:15)	9
10	**L10**	CPME	45 (94:6)	46 (98.5:1.5)	192
11	**L10**	toluene	45 (85.5:14.5)	38 (95.5:4.5)	45
12	**L10**	o-xylene	38 (87:13)	35 (96:4)	53
13	**L10**	CHCl_3_	73 (54:46)	9 (90.5:9.5)	10
14	**L10**	THF	76 (51:49)	10 (92.5:7.5)	12

CPME, cyclopentyl methyl ether; THF, tetrahydrofuran.

^a^Reactions were carried out with **1a** (0.2 mmol) with Bu_2_Mg and ligands (10 mol%), in solvents (0.2 M) for 9 h.

^b^Isolated yields of 1a* and **2a** were reported and er values were analyzed by chiral HPLC.

^c^s = ln[(1 − C)(1 − ee)]/ln[(1 − C)(1 + ee)], where ee = ee_1a*_/100, ee_1a*_ = (R_1a*_ − S_1a*_)/(R_1a*_ + S_1a*_)*100, C is monitored by HPLC analysis and calculated according to C = ee_1a*_/(ee_1a*_ + ee_**2a**_).

### Substrate scope

**Fig. 3:**
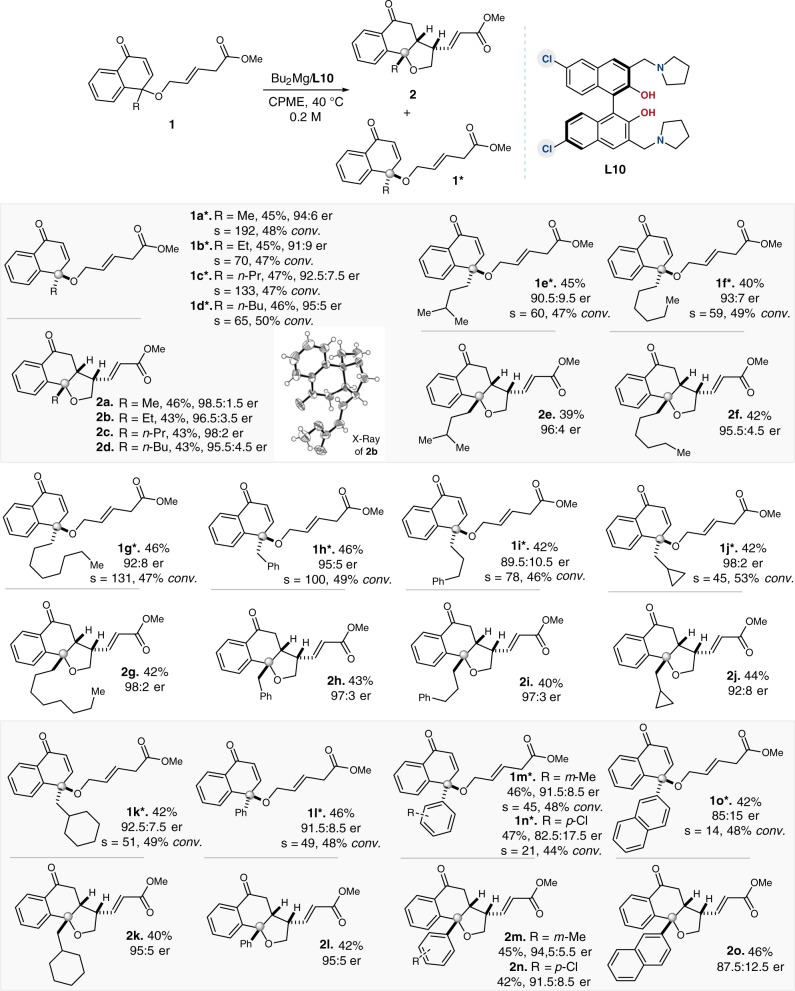
Substrate scope of the KR reaction. See Supplementary Information for the detail experiment processes. All yields shown were based on isolated products. er values were determined by chiral HPLC analysis. s = ln[(1 − C)(1 − ee)]/ln[(1 − C)(1 + ee)], where ee = ee_1a*_/100, ee_1a*_ = (R_1a*_ – S_1a*_)/(R_1a*_ + S_1a*_)*100, C is monitored by HPLC analysis and calculated according to C = ee_1a*_/(ee_1a*_ + ee_**2a**_).

Next, we investigated the scope for the intramolecular vinylogous KR reaction (Fig. 3). The magnesium catalytic system proved to be robust for the selective conversion of different alkyl-substituted substrates, furnished the desired KR process smoothly under mild conditions. A variety of substituted [6.6.5]-tricyclic rings were obtained in high enantioselectivities (92:8-98.5:1.5 er), and the enantiomerically enriched allylic esters 1* were recovered in satisfactory results. Substrates with aryl groups also finished the designed KR process, although relatively higher catalyst loading (20 mol%) was required (Fig. 3). The absolute configuration of the resolution adducts was determined by the X-ray crystallographic analysis of **2b** (Fig. 3).

**Fig. 4:**
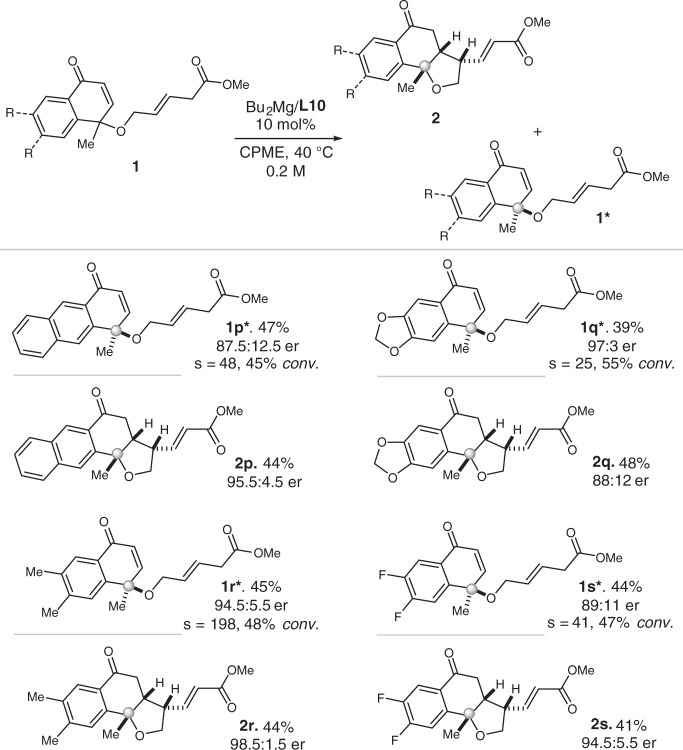
Further extensions of the substrate scope of the KR reaction. All yields shown were based on isolated products. er values were determined by chiral HPLC analysis.

Subsequently, different benzohexene ketone motifs were introduced into the allylic ester substrates and used in the vinylogous KR reaction. Polycyclic structures were established under the magnesium catalytic system. Electron-withdrawing or electron-donating groups were under trial in the KR process (Fig. 4).

**Fig. 5:**
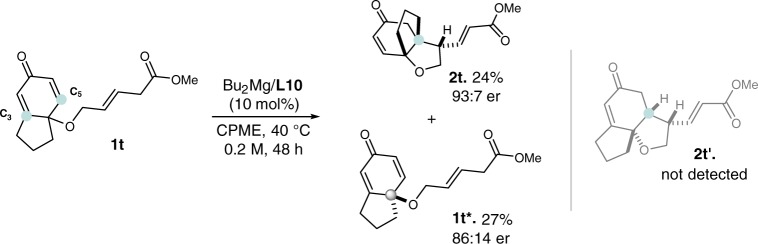
Site-selective results of the substrate 1t in the KR reaction. Isolated yields are reported.

Interestingly, it was observed that for substrate **1t**, bearing two Michael receptor sites, the vinylogous Michael reaction occurred during the KR process to form the quaternary stereocenter, and generate the bridged-ring adduct **2t**^56^. In addition, some of 1t* was recovered at a moderate er value. The common cyclization adduct **2t’** was not observed under the catalytic system, instead, some undetermined decomposition products were generated, resulting in the relatively lower yields of **2t** and 1t* (Fig. 5).

### Transformations

**Fig. 6:**
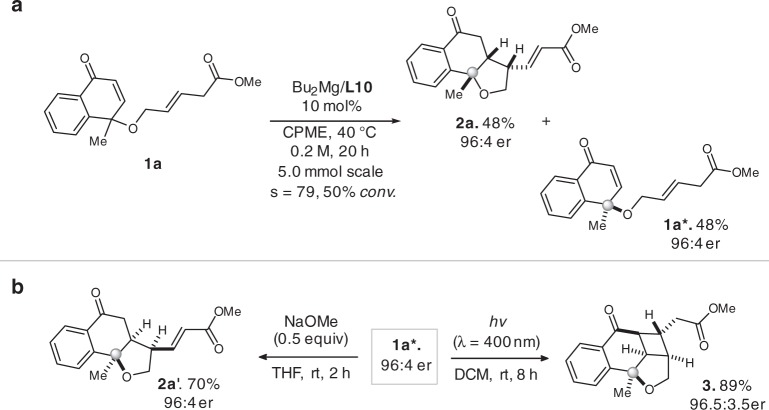
Gram scale trial and related transformation of 1a*. **a** Gram scale experiments of the KR process. **b** Transformations of the isolated chiral allylic ester 1a*.

The vinylogous KR reaction was then carried out at the gram scale and transformations of the recovered 1a* were conducted. As illustrated in Fig. 6, the recovered substrate 1a* formed the cyclization adduct **2a’**, by treatment with NaOMe. Under photocatalytic conditions lead to the polycyclic product **3** after finishing the [2 + 2] cyclization process (Fig. 6)^57–60^.

**Fig. 7:**
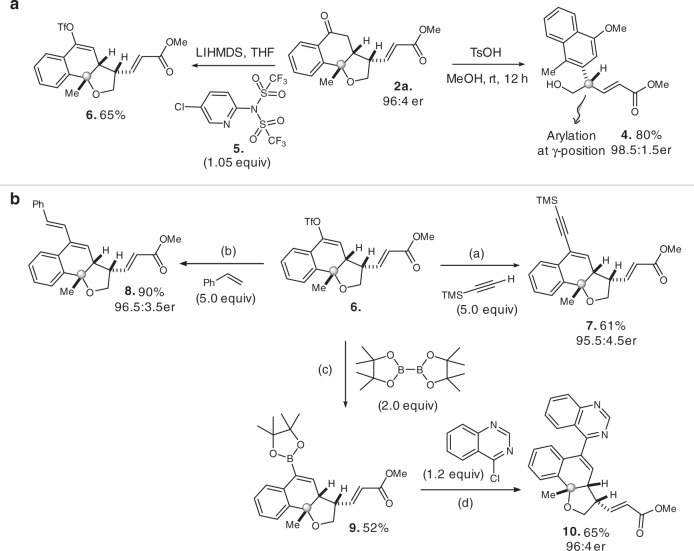
Transformations of resolution products. Conditions: **a** with CuI (2.5 mol%), Pd(PPh_3_)_4_ (2.5 mol%), Et_3_N (2.0 equiv) in DMF at room temperature. **b** with PPh_3_ (20 mol%), Pd(OAc)_2_ (10 mol%), Et_3_N (2.0 equiv) in THF/CH_3_CN at room temperature. **c** with PPh_3_ (6 mol%), PdCl_2_(PPh_3_)_2_ (3 mol%), K_2_CO_3_ (1.5 equiv) in Dioxane at 80 °C. **d** with Pd(PPh_3_)_4_ (5 mol%), K_2_CO_3_ (2.0 equiv) in H_2_O/ Dioxane reflux for 24 h.

To our pleasure, the rearomatization reaction was easily realized by treating **2a** with *p*-toluenesulfonic acid under mild conditions, This reaction can be used for the formal construction of γ-arylation adduct **4** with high enantioselectivity and good yield (Fig. 7).

Additional transformations of the tricyclic rings were performed for this central [6.6.5] skeleton to form compounds that might be useful for pharmaceutical investigations^15–19^. We introduced different functional groups or heterocyclic structures to the central skeletons by selected cross-coupling reactions. These transformations were carried out by established transition-metal mediated coupling reactions as illustrated in Fig. 7.

### Mechanistic studies

**Fig. 8:**
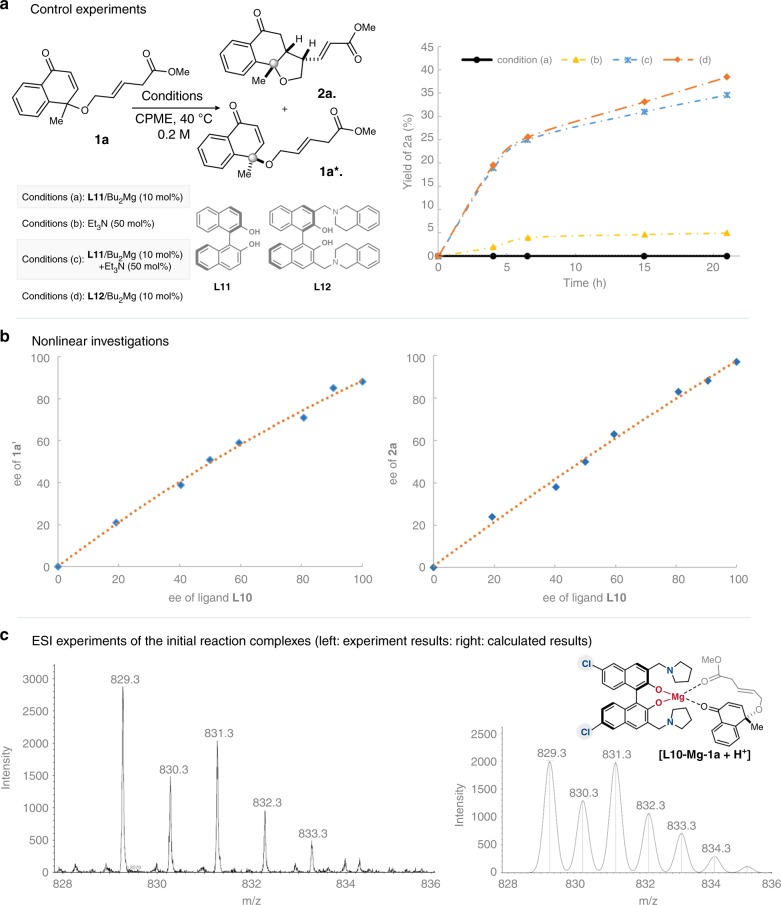
Mechanistic studies for the synergistic catalyst in the KR reaction. **a** Control experiments of the KR reaction under different catalytic conditions. **b** Nonlinear effects studies of the KR reaction. **c** ESI experiments of the initial reaction complexes to investigate the activation mode.

To investigate mechanistic aspects of the intramolecular vinylogous KR reaction, we performed a variety of mechanistic experiments. We first performed control experiments to identify the reason for the high efficiency of the bifunctional magnesium catalyst. As illustrated in Fig. 8, simple in situ generated magnesium catalyst from BINOL cannot promote the intramolecular vinylogous reaction (Fig. 8, a). Introduction of tertiary amine at high loading mediated generation of the trace cyclization adduct, and the combined use of the BINOL-Mg catalyst and tertiary amine activated the allylic ester **1a** to form intramolecular vinylogous Michael adduct **2a**. These results indicate the magnesium center and the Brønsted base can synergistically activate the designed allylic ester substrate. The developed bifunctional magnesium catalyst is more effective in the vinylogous KR reaction even with the ligand **L12** with lower Brønsted basicity (Fig. 8, a). Subsequently, studies of nonlinear effects revealed the synergistic catalyst interacts with the bidentate substrate as a mono-species (Fig. 8, b)^61,62^. Further investigations on ESI experiments of the initial reaction complexes clearly indicated the coordination results of the immediately introduced bidentate substrate **1a** to the bifunctional magnesium catalyst, which is well in accordance with the calculated results (Fig. 8, c).

### Proposed mechanism

**Fig. 9:**
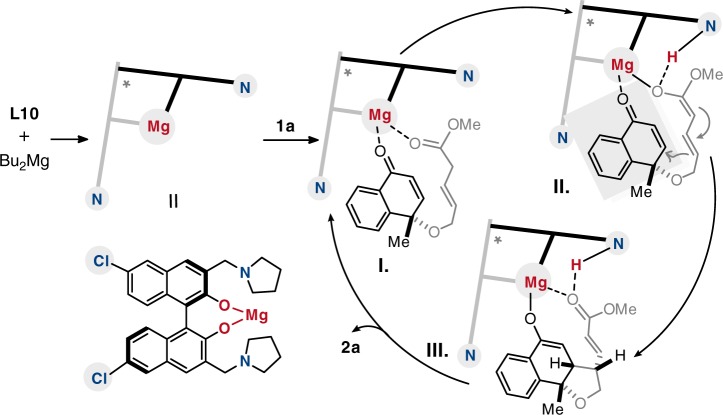
Proposed mechanism. Possible reaction mechanism for the synergistic magnesium catalyst promoted KR reaction.

Combination with the mechanistic insights, a possible mechanism cycle of the intramolecular vinylogous KR reaction is proposed (Fig. 9). The bifunctional magnesium catalyst is smoothly generated from **L10** and Bu_2_Mg after the neutralization process, then the bidentate substrate coordinates to the magnesium center and the tertiary amine synergistically promotes enolation of the allylic ester. At the same time, the bidentate coordination results in synchronous activation of the Michael receptor to promote the intramolecular vinylogous reaction in the well-controlled chiral environment (Fig. 9, II and III). Finally, the protonation process and the entry of another molecule of **1a** lead to the release of the KR product **2a**.

## Discussion

In summary, we have accomplished a direct catalytic asymmetric intramolecular vinylogous Michael reaction. Bifunctional chiral ligands were developed to generate synergistic magnesium catalysts. Using the designed allylic ester substrates, the KR process successfully led to the expected [6.6.5]-tricyclic key skeletons. Several transformations were conducted to give types of chiral polycyclic structures and derivatives of the [6.6.5]-tricyclic skeletons, as well as the enantioselective γ-arylation adduct. Combinational mechanistic insights, including control experiments, nonlinear effects studies and relative ESI investigations, led to the proposal of a possible mechanism of this intramolecular vinylogous KR reaction. Further developments of the reported synergistic magnesium catalyst in asymmetric reactions are underway in our laboratory.

## Methods

### General procedure for the vinylogous KR reaction

To a stirred solution of **L10** (10.42 mg, 0.02 mmol) in CPME (0.5 mL) was added Bu_2_Mg (20 μL, 1.0 M in heptane, 0.02 mmol) under an argon atmosphere, the mixture was then stirred at room temperature for 30 min to generate the catalyst. The substrate **1** (0.2 mmol) in CPME (0.5 mL) was quickly added to the flask containing the in situ generated magnesium catalyst. After the addition, the reaction was stirred at 40 °C and analyzed by TLC. The reaction was quenched with saturated NH_4_Cl and extracted with CH_2_Cl_2_. The organic layer was dried over anhydrous Na_2_SO_4_ and concentrated under vacuum. Then the residue was purified by column chromatography to afford the product 1^*^ and **2**.

## Supplementary information

Supplementary Information

Peer Review File

## Data Availability

Detailed experimental procedures and characterization of compounds can be found in the Supplementary Information. The X-ray crystallographic coordinates for structures reported in this article have been deposited at the Cambridge Crystallographic Data Center (**L10**: CCDC 1978958; **2b**: CCDC 1978955). These data could be obtained free of charge from The Cambridge Crystallographic Data Center via www.ccdc.cam.ac.uk/data_request/cif. All data are available from the authors upon request.

## References

[1] Keith JM, Larrow JF, Jacobsen EN (2001). Practical considerations in kinetic resolution reactions. Adv. Synth. Catal..

[2] Robinson DEJE, Bull SD (2003). Kinetic resolution strategies using non-enzymatic catalysts. Tetrahedron: Asymmetry.

[3] Vedejs E, Jure M (2005). Efficiency in nonenzymatic kinetic resolution. Angew. Chem. Int. Ed..

[4] Pellissier H (2011). Catalytic non‐enzymatic kinetic resolution. Adv. Synth. Catal..

[5] La DS (1998). Mo-catalyzed asymmetric synthesis of dihydrofurans. catalytic kinetic resolution and enantioselective desymmetrization through ring-closing metathesis. J. Am. Chem. Soc..

[6] Tanaka K, Fu GC (2002). Enantioselective synthesis of cyclopentenones via rhodium-catalyzed kinetic resolution and desymmetrization of 4-alkynals. J. Am. Chem. Soc..

[7] Tanaka K, Fu GC (2003). Parallel kinetic resolution of 4-alkynals catalyzed by Rh(I)/Tol-BINAP: synthesis of enantioenriched cyclobutanones and cyclopentenones. J. Am. Chem. Soc..

[8] Čorić I, Müller S, List B (2010). Kinetic resolution of homoaldols via catalytic asymmetric transacetalization. J. Am. Chem. Soc..

[9] Wang Y, Zheng K, Hong R (2012). Chiral silver phosphate-catalyzed cycloisomeric kinetic resolution of α-allenic alcohols. J. Am. Chem. Soc..

[10] Ogasawara M (2012). Kinetic resolution of planar-chiral (η6-arene)chromium complexes by molybdenum-catalyzed asymmetric ring-closing metathesis. Angew. Chem. Int. Ed..

[11] Liu Y (2016). Synergistic kinetic resolution and asymmetric propargyl Claisen rearrangement for the synthesis of chiral allenes. Angew. Chem. Int. Ed..

[12] Loh CCJ (2016). Rhodium-catalyzed asymmetric cycloisomerization and parallel kinetic resolution of racemic oxabicycles. Angew. Chem. Int. Ed..

[13] Bohan PT, Toste FD (2017). Well-defined chiral gold(III) complex catalyzed direct enantioconvergent kinetic resolution of 1,5-enynes. J. Am. Chem. Soc..

[14] Deng L (2019). Kinetic resolution via Rh-catalyzed C-C activation of cyclobutanones at room temperature. J. Am. Chem. Soc..

[15] Löbermann F, Weisheit L, Trauner D (2013). Intramolecular vinyl quinone Diels-Alder reactions: asymmetric entry to the cordiachrome core and synthesis of (-)-isoglaziovianol. Org. Lett..

[16] Chen H (2017). Discovery of potent small-molecule inhibitors of ubiquitin-conjugating enzyme UbcH5c from α-santonin derivatives. J. Med. Chem..

[17] Yang B, Lin K, Shi Y, Gao S (2017). Ti(O*i*-Pr)_4_-promoted photoenolization Diels-Alder reaction to construct polycyclic rings and its synthetic applications. Nat. Commun..

[18] Coricello A (2018). Rational drug design and synthesis of new α-santonin derivatives as potential COX-2 inhibitors. Bioorg. Med Chem. Lett..

[19] Kamo S (2019). Synthetic and biological studies of juglorubin and related naphthoquinones. J. Org. Chem..

[20] Zhuo C, Zhang W, You S-L (2012). Catalytic asymmetric dearomatization reactions. Angew. Chem. Int. Ed..

[21] Rousseaux S, García-Fortanet J, Sanchez MADA, Buchwald SL (2011). Palladium(0)-catalyzed arylative dearomatization of phenols. J. Am. Chem. Soc..

[22] Yang L (2015). Palladium-catalyzed dynamic kinetic asymmetric transformation of racemic biaryls: axial-to-central chirality transfer. J. Am. Chem. Soc..

[23] Cheng Q, Wang Y, You S-L (2016). Chemo-, diastereo-, and enantioselective iridium-catalyzed allylic intramolecular dearomatization reaction of naphthol derivatives. Angew. Chem. Int. Ed..

[24] Bai L (2016). Palladium(0)-catalyzed intermolecular carbocyclization of (1,n)-diynes and bromophenols: an efficient route to tricyclic scaffolds. Angew. Chem. Int. Ed..

[25] Zhao G, Xu G, Qian C, Tang W (2017). Efficient enantioselective syntheses of (+)-dalesconol A and B. J. Am. Chem. Soc..

[26] Xia Z-L, Zheng C, Xu R-Q, You S-L (2019). Chiral phosphoric acid catalyzed aminative dearomatization of α-naphthols/Michael addition sequence. Nat. Commun..

[27] Zhu B (2013). Direct asymmetric vinylogous Aldol reaction of allyl ketones with isatins: divergent synthesis of 3-hydroxy-2-oxindole derivatives. Angew. Chem. Int. Ed..

[28] Gu Y, Wang Y, Yu T-Y, Liang Y-M, Xu P-F (2014). Rationally designed multifunctional supramolecular iminium catalysis: direct vinylogous Michael addition of unmodified linear dienol substrates. Angew. Chem. Int. Ed..

[29] Ran G-Y, Yang X-X, Yue J-F, Du W, Chen Y-C (2019). Asymmetric allylic alkylation with deconjugated carbonyl compounds: direct vinylogous umpolung strategy. Angew. Chem. Int. Ed..

[30] Yamaguchi A, Matsunaga S, Shibasaki M (2009). Catalytic asymmetric synthesis of alpha-alkylidene-beta-hydroxy esters via dynamic kinetic asymmetric transformation involving Ba-catalyzed direct Aldol reaction. J. Am. Chem. Soc..

[31] Iriarte I (2017). Controlling the α/γ-reactivity of vinylogous ketone enolates in organocatalytic enantioselective Michael reactions. Angew. Chem. Int. Ed..

[32] Frias M, Mas-Ballesté R, Arias S, Alvarado C, Alemán J (2017). Asymmetric synthesis of Rauhut-Currier type products by a regioselective Mukaiyama reaction under bifunctional catalysis. J. Am. Chem. Soc..

[33] Denmark SE, Beutner GL (2003). Lewis Base Activation of Lewis acids. vinylogous Aldol reactions. J. Am. Chem. Soc..

[34] Moreau X, Bazán-Tejeda B, Campagne J-M (2005). Catalytic and asymmetric vinylogous Mukaiyama reactions on aliphatic ketones: formal asymmetric synthesis of taurospongin A. J. Am. Chem. Soc..

[35] Ratjen L, Garca PG, Lay F, Beck ME, List B (2011). Disulfonimide-catalyzed asymmetric vinylogous and bisvinylogous Mukaiyama Aldol reactions. Angew. Chem. Int. Ed..

[36] Gupta V, Sudhir VS, Mandal T, Schneider C (2012). Organocatalytic, highly enantioselective vinylogous Mukaiyama-Michael reaction of acyclic dienol silyl ethers. Angew. Chem. Int. Ed..

[37] Curti C (2015). Pushing the boundaries of vinylogous reactivity: catalytic enantioselective Mukaiyama Aldol Rreactions of highly unsaturated 2-silyloxyindoles. Chem. Eur. J..

[38] Zhang H-J, Shi C-Y, Zhong F, Yin L (2017). Direct asymmetric vinylogous and bisvinylogous mannich-type reaction catalyzed by a copper(I) complex. J. Am. Chem. Soc..

[39] Zhong F, Yue W-J, Zhang H-J, Zhang C-Y, Yin L (2018). Catalytic asymmetric construction of halogenated stereogenic carbon centers by direct vinylogous Mannich-type reaction. J. Am. Chem. Soc..

[40] Zhang H-J, Yin L (2018). Asymmetric synthesis of α,β-unsaturated δ-lactones through copper(I)-catalyzed direct vinylogous Aldol reaction. J. Am. Chem. Soc..

[41] Zhong F, Xue Q-Y, Yin L (2020). Construction of chiral 2,3-allenols through a copper(I)-catalyzed asymmetric direct alkynylogous Aldol reaction. Angew. Chem. Int. Ed..

[42] Gray D, Gallagher T (2006). A flexible strategy for the synthesis of tri- and tetracyclic lupin alkaloids: synthesis of (+)-cytisine, (±)-anagyrine, and (±)-thermopsine. Angew. Chem. Int. Ed..

[43] Gallagher T (2010). Intramolecular 1,6-addition to 2-pyridones. Mechanism Synth. scope. J. Org. Chem..

[44] Harish B, Subbireddy M, Obulesu O, Suresh S (2019). One-Pot allylation–intramolecular vinylogous Michael addition-isomerization cascade of *o*-hydroxycinnamates and congeners: synthesis of substituted benzofuran derivatives. Org. Lett..

[45] Yang D, Wang L, Li D, Wang R (2019). Magnesium catalysis in asymmetric synthesis. Chem.

[46] Trost BM, Malhotra S, Fried BA (2009). Magnesium-catalyzed asymmetric direct Aldol addition of ethyl diazoacetate to aromatic, aliphatic, and alpha, beta-unsaturated aldehydes. J. Am. Chem. Soc..

[47] Yoshino T, Morimoto H, Lu G, Matsunaga S, Shibasaki M (2009). Construction of contiguous tetrasubstituted chiral carbon stereocenters via direct catalytic asymmetric Aldol reaction of alpha-isothiocyanato esters with ketones. J. Am. Chem. Soc..

[48] Hatano M, Horibe T, Ishihara K (2010). Magnesium(II)-binaphtholate as a practical chiral catalyst for the enantioselective direct Mannich-type reaction with malonates. Org. Lett..

[49] Hatano M, Horibe T, Ishihara K (2013). Chiral magnesium(II) binaphtholates as cooperative Brønsted/Lewis acid-base catalysts for the highly enantioselective addition of phosphorus nucleophiles to α,β-unsaturated esters and ketones. Angew. Chem. Int. Ed..

[50] Yang D (2013). Direct site-specific and highly enantioselective γ-functionalization of linear α,β-unsaturated ketones: bifunctional catalytic strategy. Angew. Chem. Int. Ed..

[51] Yang D (2015). Intermolecular enantioselective dearomatization reaction of β-naphthol using meso-aziridine: a bifunctional in situ generated magnesium catalyst. Angew. Chem. Int. Ed..

[52] Yang D (2015). Application of a C-C bond-forming conjugate addition reaction in asymmetric dearomatization of β-naphthols. Angew. Chem. Int. Ed..

[53] Hatano M, Nishikawa K, Ishihara K (2017). Enantioselective cycloaddition of styrenes with aldimines catalyzed by a chiral magnesium potassium binaphthyldisulfonate cluster as a chiral Brønsted acid catalyst. J. Am. Chem. Soc..

[54] Wang L (2018). The Important role of the byproduct triphenylphosphine oxide in the magnesium(II)-catalyzed enantioselective reaction of hemiacetals and phosphorus ylides. Angew. Chem. Int. Ed..

[55] Falconnet A, Magre M, Maity B, Cavallo L, Rueping M (2019). Asymmetric magnesium-catalyzed hydroboration by metal-ligand cooperative catalysis. Angew. Chem. Int. Ed..

[56] Hashimoto T, Nakatsu H, Maruoka K (2015). Catalytic asymmetric Diels-Alder reaction of quinone imine ketals: a site-divergent approach. Angew. Chem. Int. Ed..

[57] Brimioulle R, Bauer A, Bach T (2015). Enantioselective lewis acid catalysis in Intramolecular [2+2] photocycloaddition reactions: a mechanistic comparison between representative coumarin and enone substrates. J. Am. Chem. Soc..

[58] Wang H, Cao X, Chen X, Fang W, Dolg M (2015). Regulatory mechanism of the enantioselective intramolecular enone [2+2] photocycloaddition reaction mediated by a chiral Lewis acid catalyst containing heavy atoms. Angew. Chem. Int. Ed..

[59] Brenninger C, Pöthig A, Bach T (2017). Brønsted acid catalysis in visible-light-induced [2+2] photocycloaddition reactions of enone dithianes. Angew. Chem. Int. Ed..

[60] Poplata S, Bauer A, Storch G, Bach T (2019). Intramolecular [2+2] photocycloaddition of cyclic enones: selectivity control by lewis acids and mechanistic implications. Chem. Eur. J..

[61] Satyanarayana T, Abraham S, Kagan HB (2009). Nonlinear effects in asymmetric catalysis. Angew. Chem. Int. Ed..

[62] Noble-Terán ME, Buhse T, Cruz J-M, Coudret C, Micheau J-C (2016). Nonlinear effects in asymmetric synthesis: a practical tool for the discrimination between monomer and dimer catalysis. ChemCatChem.

