# Supplementary material for: Activation of allylic esters in an intramolecular vinylogous kinetic resolution reaction with synergistic magnesium catalysts
Source: Nat Commun. 2020 May 22;11:2559. doi: 10.1038/s41467-020-16486-0 (PMC7244749; doi:10.1038/s41467-020-16486-0)
Supplement: Supplementary file 1 — Supplementary Information [file 41467_2020_16486_MOESM1_ESM.pdf]

Supplementary Information

Activation of Allylic Esters in an Intramolecular Vinylogous Kinetic Resolution Reaction with Synergistic Magnesium Catalysts

Li et al.

Supplementary Methods

General Remarks

All reactions were performed under an argon atmosphere and solvents were dried according to established procedures. ^{1}H NMR (300 MHz), and ^{13}C NMR (75 M or 150 MHz) spectra were obtained in $CDCl_3$ or DMSO. The chemical shifts are reported in ppm relative to internal standard TMS (^{1}H NMR), to residual signals of the solvents ($CHCl_3$, 7.26 ppm for ^{1}H NMR and 77.0 ppm for ^{13}C NMR). IR spectra were recorded on a FT-IR spectrometer with KBr pellet and only major peaks were reported in cm^{-1}. High resolution massspectra (HRMS) were obtained by the ESI ionization sources. The er values determination were carried out using chiral HPLC on Waters with a 2996UV-detector. Bu_2Mg (1.0 M in heptane) is commercially available at Sigma-Aldrich.

Supplementary Discussion

Mechanistic Studies for the Synergistic Catalyst in the KR Reaction

(1) Control experiments

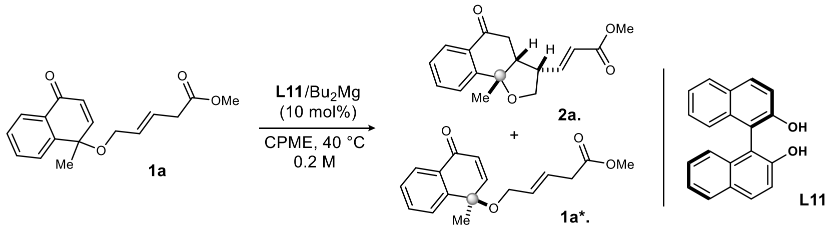

time (h)	Conversions (%)
0	0
4	0
6.5	0
15	0
21	0

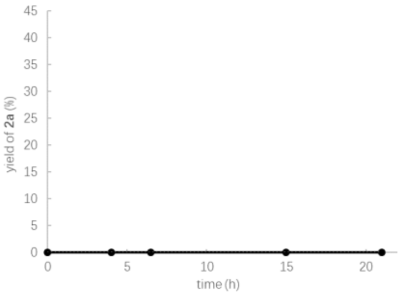

Supplementary Figure 1. Control experiment using **L11** as chiral ligand

Methods: To a stirred solution of **L11** (5.72 mg, 0.02 mmol) in CPME (0.5 mL) was added **1a** (0.2 mmol) in CPME (0.5 mL) and stirred at 40 ℃ for each identified time. The mixture was quenched with saturated NH_4Cl and extracted with CH_2Cl_2. The organic layer was dried over Na_2SO_4 and concentrated under vacuum for ^{1}H NMR studies to confirm the conversions.

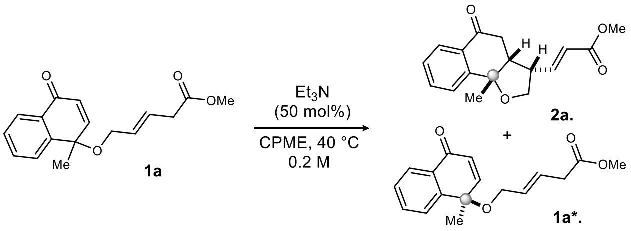

time (h)	Conversions (%)
0	0
4	2
6.5	4
15	4.7
21	5

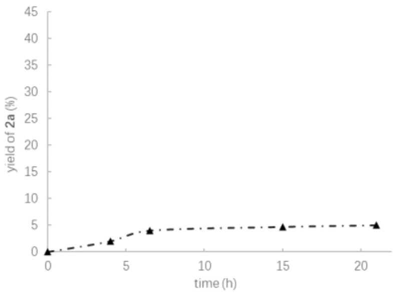

Supplementary Figure 2. Control experiment using trimethylamine (Et_3N) as catalyst

Methods: To a stirred solution of **1a** (0.2 mmol) in CPME (1.0 mL) was added Et_3N (14 µL, 0.1 mmol) and stirred at 40 ℃ for each identified time. The reaction was quenched with saturated NH_4Cl and extracted with CH_2Cl_2. The organic layer was dried over Na_2SO_4 and concentrated under vacuum for ^1H NMR studies to confirm the conversions.

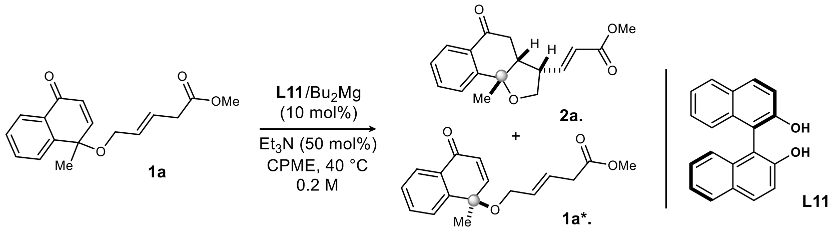

time (h)	Conversions (%)
0	0
4	18.9
6.5	25
15	31
21	34.6

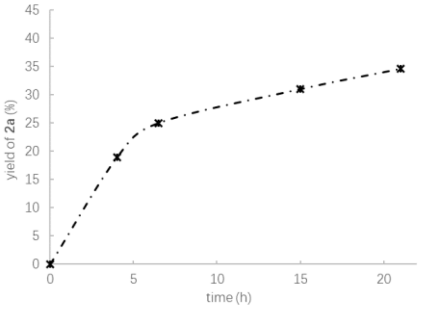

Supplementary Figure 3. Control experiment using combinational catalyst

Methods: To a stirred solution of **L11** (5.72 mg, 0.02 mmol) in CPME (0.5 mL) was added Bu_2Mg (20 µL, 1.0 M in heptane, 0.02 mmol) under an argon atmosphere, the mixture was then stirred at room temperature for 30 min to generate the catalyst. **1a** (0.2 mmol) and Et_3N (14 µL, 0.1 mmol) in CPME (0.5 mL) was quickly added to the flask containing the in situ generated magnesium catalyst. After the addition, the reaction was stirred at 40 ℃ for each identified time. The reaction was quenched with saturated NH_4Cl and extracted with CH_2Cl_2. The organic layer was dried over Na_2SO_4 and concentrated under vacuum for ^1H NMR studies to confirm the conversions.

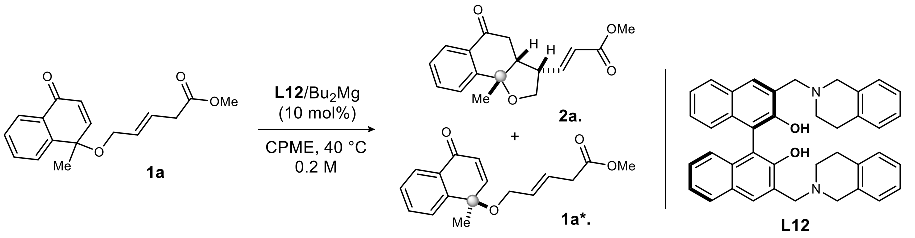

time (h)	Conversions (%)
0	0
4	19.6
6.5	25.6
15	33.2
21	38.5

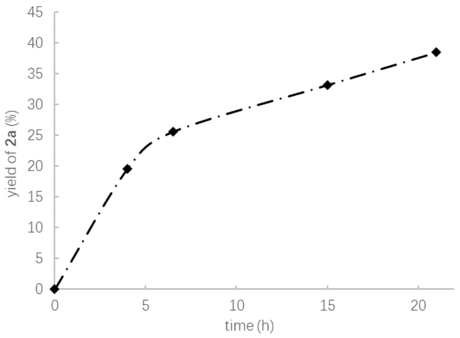

Supplementary Figure 4. Control experiment using **L12** as chiral ligand

Methods: To a stirred solution of **L12** (11.52 mg, 0.02 mmol) in CPME (0.5 mL) was added Bu_2Mg (20 μL, 1.0 M in heptane, 0.02 mmol) under an argon atmosphere, the mixture was then stirred at room temperature for 30 min to generate the catalyst. **1a** (0.2 mmol) in CPME (0.5 mL) was quickly added to the flask containing the in situ generated magnesium catalyst. After the addition, the reaction was stirred at 40 ℃ for each identified time. The reaction was quenched with saturated NH_4Cl and extracted with CH_2Cl_2. The organic layer was dried over Na_2SO_4 and concentrated under vacuum for ^{1}H NMR studies to confirm the conversions.

(2) Nonlinear effects studies

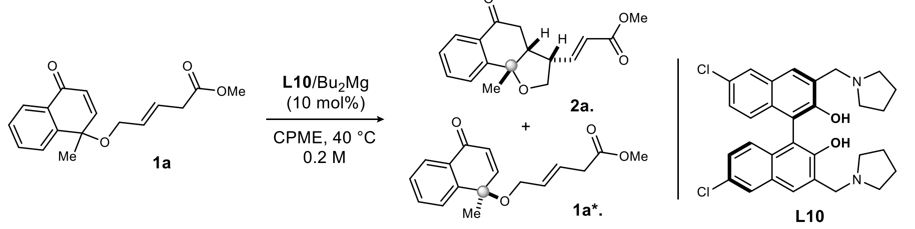

ee (**L10**)	ee (**1a***)
0	0
19.2	21
40.4	39
50	51
59.6	59
80.8	71
90.4	85
100	88

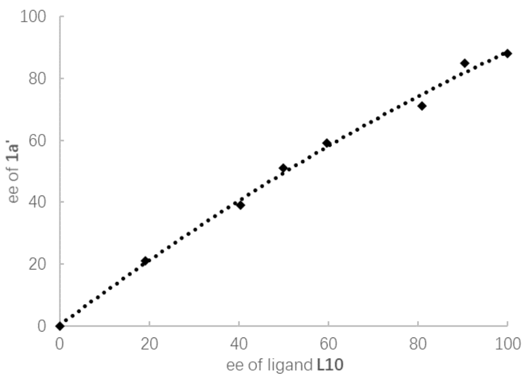

Supplementary Figure 5. Nonlinear investigation results between **1a*** and **L10**

ee (**L10**)	ee (**1a***)
0	0
19.2	24
40.4	38
50	50
59.6	63
80.8	83
90.4	88
100	97

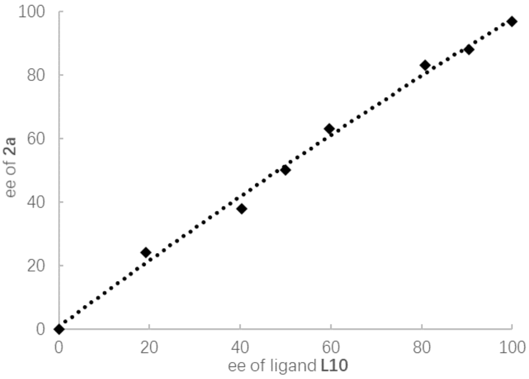

Supplementary Figure 6. Nonlinear investigation results between **2a** and **L10**

Methods: To a stirred solution of **L10** (11.52 mg, 0.02 mmol) with different *ee* value from different ratio of (*R*)-**L10** and (*S*)-**L10** in CPME (0.5 mL), was added Bu_2Mg (20 µL, 1.0 M in heptane, 0.02 mmol) under an argon atmosphere The mixture was then stirred at room temperature for 30 min to generate the catalyst. **1a** (0.2 mmol) in CPME (0.5 mL) was quickly added to the flask containing the in situ generated magnesium catalyst. After the addition, the reaction was stirred at 40 ℃ for 9 hours. The reaction was quenched with saturated NH_4Cl and extracted with CH_2Cl_2. The organic layer was dried over Na_2SO_4 and concentrated under vacuum. Then the residue was purified by column chromatography to afford the resolution product **1a*** and **2a**. The ee value determination was carried out using chiral HPLC on Waters with a 2996UV-detector.

(3) ESI analysis of the initial reaction mixtures

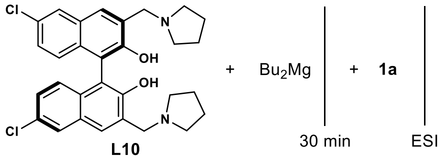

L10

Supplementary Figure 7. The generation process of the initial reaction mixtures

Methods: To a stirred solution of **L10** (11.52 mg, 0.02 mmol) in CPME (0.5 mL) was added Bu_2Mg (20 µL, 1.0 M in heptane, 0.02 mmol) under an argon atmosphere, the mixture was then stirred at room temperature for 30 min to generate the catalyst. **1a** (0.2 mmol) in CPME (0.5 mL) was quickly added to the flask containing the in situ generated magnesium catalyst. The mixture was immediately analyzed on high resolution masss pectra (HRMS) by the ESI ionization sources.

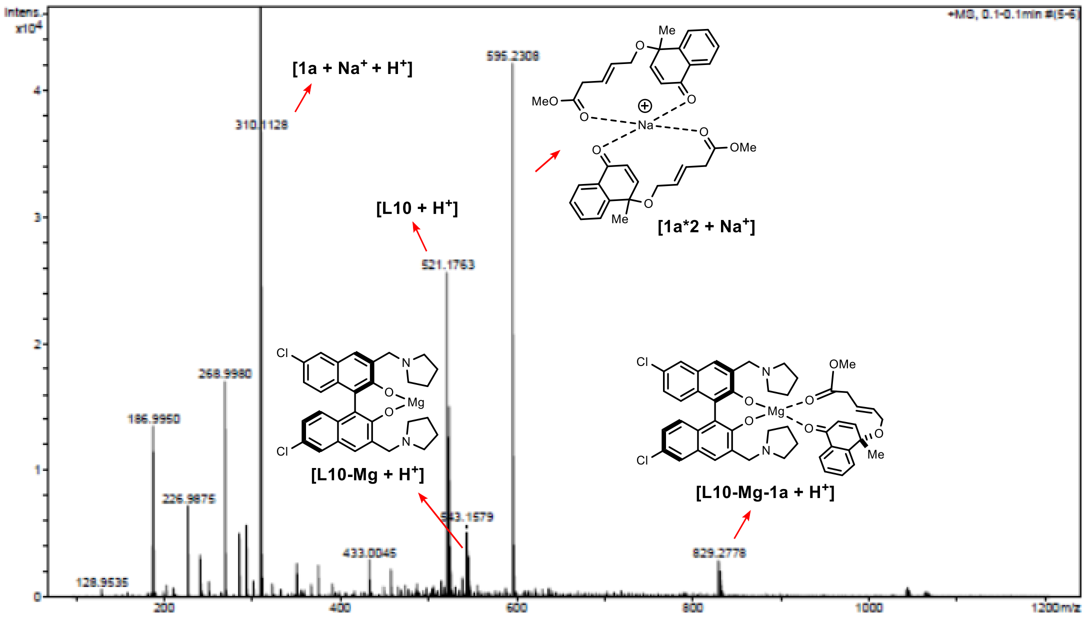

Supplementary Figure 8. ESI analysis results of the initial reaction mixtures

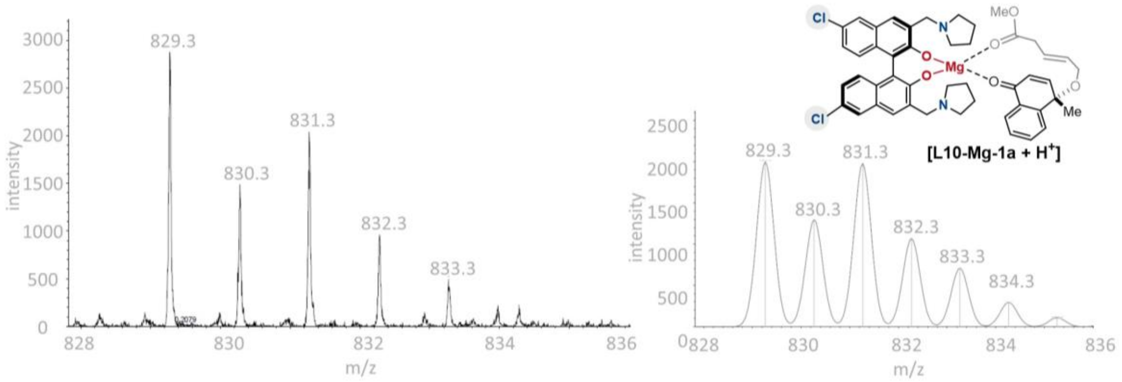

left: experiment results right: calculated results

Supplementary Figure 9. Key catalytic active species captured by the ESI analysis

Based on these investigations, the real catalytic sepcies and active intermediate are identified, so a possible mechanism is proposed in the maintext.

Experimental Procedures for the Vinylogous KR Reaction

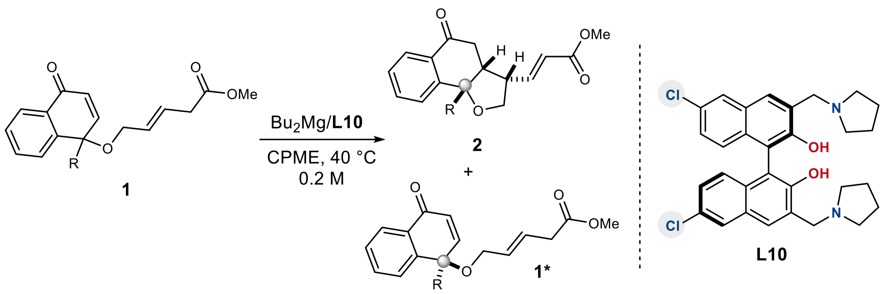

Supplementary Figure 10. The general kinetic resolution reaction

To a stirred solution of **L10** (10.42 mg, 0.02 mmol) in CPME (0.5 mL) was added Bu_2Mg (20 μL, 1.0 M in heptane, 0.02 mmol) under an argon atmosphere, the mixture was then stirred at room temperature for 30 min to generate the catalyst. The substrate **1** (0.2 mmol) in CPME (0.5 mL) was quickly added to the flask containing the in situ generated magnesium catalyst. After the addition, the reaction was stirred at 40 °C and analyzed by TLC. The reaction was quenched with saturated NH_4Cl and extracted with CH_2Cl_2. The organic layer was dried over Na_2SO_4 and concentrated under vacuum. Then the residue was purified by column chromatography to afford the product **1*** and **2**.

(For aryl-substituted substrates, the reaction might need relatively higher loading of catalyst amount and longer reaction time)

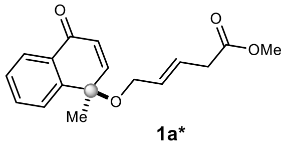

1a*

With 10 mol% catalyst at 40 °C for 9 h.

Purified by silica gel chromatography (ethyl acetate/petroleum ether 10:1); Colorless oil; 13.9 mg, 45% yield; 94:6 *er* determined by HPLC on a Chiralpak IC-H column (hexane/2-propanol = 40/60, flow rate = 1.0 mL/min, t_{minor} = 10.0min, t_{major} = 8.6 min);

$[\alpha]^{rt}_D$ = -78.9 (*c* = 0.95, $CHCl_3$);

^{1}H NMR (300 MHz, $CDCl_3$) δ 8.13 (d, *J* = 7.8 Hz, 1H), 7.80 – 7.57 (m, 2H), 7.46 (dd, *J* = 9.7, 4.8 Hz, 1H), 6.97 (d, *J* = 10.3 Hz, 1H), 6.48 (d, *J* = 10.3 Hz, 1H), 5.93 – 5.41 (m, 2H), 3.82 – 3.61 (m, 4H), 3.52 (dd, *J* = 11.7, 5.5 Hz, 1H), 3.07 (d, *J* = 6.6 Hz, 2H), 1.63 (s, 3H);

^{13}C NMR (75 MHz, $CDCl_3$) δ 184.2, 171.7, 152.5, 145.0, 133.3, 131.2, 130.3, 130.0, 128.2, 126.7, 126.2, 125.2, 73.7, 65.5, 51.8, 37.5, 30.5;

IR (KBr): 2980, 2857, 1983, 1738, 1668, 1602, 1458, 1409, 1300, 1084, 769, 573 cm^{-1};

HRMS (ESI): $C_{17}H_{18}NaO_4$ $[M+Na]^+$ calcd: 309.1097, found: 309.1098.

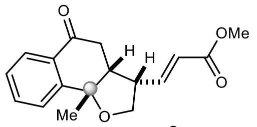

2a

Purified by silica gel chromatography (ethyl acetate/petroleum ether 4:1); White solid, m.p 85-87 °C; 14.2 mg, 46% yield; 98.5:1.5 *er* determined by HPLC on a Chiralpak IA-H column (hexane/2-propanol = 40/60, flow rate = 1.0 mL/min, t_{minor} = 9.8 min, t_{major} = 8.9 min);

$[\alpha]^{rt}_D$ = 100.8 (*c* = 0.98, $CHCl_3$);

^{1}H NMR (300 MHz, $CDCl_3$) δ 7.96 (d, *J* = 7.1 Hz, 1H), 7.78 – 7.54 (m, 2H), 7.45 – 7.36 (m, 1H), 6.22 (dd, *J* = 15.5, 9.8 Hz, 1H), 5.75 (dd, *J* = 15.6, 0.6 Hz, 1H), 4.16 (dd, *J* = 9.1, 7.8 Hz, 1H), 3.65 (s, 3H), 3.44 (dd, *J* = 9.2, 6.0 Hz, 1H), 3.38 – 3.19 (m, 1H), 2.82 (d, *J* = 2.1 Hz, 3H), 1.66 (s, 3H);

^{13}C NMR (75 MHz, $CDCl_3$) δ 195.2, 165.7, 146.7, 146.0, 134.7, 131.4, 128.1, 126.7, 126.2, 124.0, 80.4, 70.2, 51.6, 48.1, 45.5, 36.0, 28.5;

IR (KBr): 2950, 1722, 1686, 1655, 1600, 1275, 1255, 1171, 773, 639 cm^{-1};

HRMS (ESI): $C_{17}H_{18}NaO_4$ [M+Na]$^+$ calcd: 309.1097, found: 309.1096.

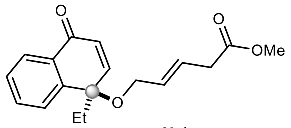

1b*

With 10 mol% catalyst at 40 °C for 12 h.

Purified by silica gel chromatography (ethyl acetate/petroleum ether 10:1); Colorless oil; 14.5 mg, 45% yield; 91:9 *er* determined by HPLC on a Chiralpak IC-H column (hexane/2-propanol = 40/60, flow rate = 1.0 mL/min, t_{minor} = 8.7 min, t_{major} = 7.6 min);

$[\alpha]^{rt}_D$ = -45.0 (*c* = 1.00, $CHCl_3$);

^{1}H NMR (300 MHz, $CDCl_3$) δ 8.13 (d, *J* = 7.8 Hz, 1H), 7.63 (d, *J* = 3.3 Hz, 2H), 7.54 – 7.34 (m, 1H), 6.89 (d, *J* = 10.3 Hz, 1H), 6.57 (d, *J* = 10.3 Hz, 1H), 5.69 (tdd, *J* = 21.1, 15.3, 6.2 Hz, 2H), 3.86 – 3.64 (m, 4H), 3.57 (dd, *J* = 11.6, 5.3 Hz, 1H), 3.07 (d, *J* = 6.6 Hz, 2H), 2.01 (dd, *J* = 14.4, 7.1 Hz, 2H), 0.57 (t, *J* = 7.5 Hz, 3H);

^{13}C NMR (75 MHz, $CDCl_3$) δ 184.5, 171.8, 151.5, 143.9, 133.2, 132.4, 131.6, 130.5, 128.1, 126.5, 125.9, 125.0, 77.8, 65.3, 51.8, 37.5, 35.7, 8.0;

IR (KBr): 2969, 1985, 1740, 1669, 1601, 1296, 1166, 843, 768, 574 cm^{-1};

HRMS (ESI): $C_{18}H_{20}NaO_4$ [M+Na]$^+$ calcd: 323.1254, found: 323.1253.

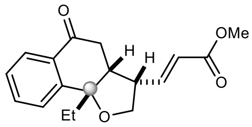

2b

Purified by silica gel chromatography (ethyl acetate/petroleum ether 4:1); White solid, m.p 65-67 °C; 13.8 mg, 43% yield; 96.5:3.5 *er* determined by HPLC on a Chiralpak IA-H column (hexane/2-propanol = 40/60, flow rate = 1.0 mL/min, t_{minor} = 5.0 min, t_{major} = 4.5 min);

$[\alpha]^{rt}_D$ = 87.0 (*c* = 1.00, $CHCl_3$);

^{1}H NMR (300 MHz, $CDCl_3$) δ 7.95 (d, *J* = 7.8 Hz, 1H), 7.64 (d, *J* = 3.9 Hz, 2H), 7.41 (dt, *J* = 8.1, 4.2 Hz, 1H), 6.19 (dd, *J* = 15.6, 9.9 Hz, 1H), 5.74 (d, *J* = 15.6 Hz, 1H), 4.14 (dd, *J* = 8.9, 7.8 Hz, 1H), 3.64 (s, 3H), 3.51 (dd, *J* = 9.2, 5.6 Hz, 1H), 3.37 – 3.16 (m, 1H), 2.98 – 2.85 (m, 1H), 2.80 (d, *J* = 5.0 Hz, 2H), 2.08 – 1.86 (m, 2H), 0.91 (t, *J* = 7.5 Hz, 3H);

^{13}C NMR (75 MHz, $CDCl_3$) δ 195.9, 165.8, 146.1, 145.9, 134.4, 132.1, 128.0, 127.0, 126.1, 123.9, 82.6, 70.1, 51.5, 45.9, 44.6, 36.0, 34.1, 8.6;

IR (KBr): 2937, 1960, 1724, 1686, 1600, 1287, 1171, 988, 768, 598 cm^{-1};

HRMS (ESI): $C_{18}H_{20}NaO_4$ [M+Na]$^+$ calcd: 323.1254, found: 323.1252.

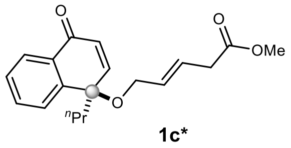

1c*

With 10 mol% catalyst at 40 °C for 12 h.

Purified by silica gel chromatography (ethyl acetate/petroleum ether 10:1); Colorless oil; 15.8 mg, 47% yield; 92.5:7.5 *er* determined by HPLC on a Chiralpak IC-H column (hexane/2-propanol = 40/60, flow rate = 1.0 mL/min, t_{minor} = 8.4 min, t_{major} = 7.6 min);

$[\alpha]^{rt}_{D}$ = -40.4 (*c* = 0.99, $CHCl_3$);

^{1}H NMR (300 MHz, $CDCl_3$) δ 8.12 (d, *J* = 7.8 Hz, 1H), 7.62 (t, *J* = 5.9 Hz, 2H), 7.45 (dt, *J* = 8.1, 4.1 Hz, 1H), 6.92 (d, *J* = 10.3 Hz, 1H), 6.55 (d, *J* = 10.3 Hz, 1H), 5.87 – 5.35 (m, 2H), 3.85 – 3.63 (m, 4H), 3.55 (dd, *J* = 11.9, 5.4 Hz, 1H), 3.07 (d, *J* = 6.7 Hz, 2H), 1.95 (dd, *J* = 9.6, 4.4 Hz, 2H), 1.23 – 1.03 (m, 1H), 0.82 – 0.70 (m, 4H);

^{13}C NMR (75 MHz, $CDCl_3$) δ 184.5, 171.8, 151.9, 144.2, 133.2, 132.1, 131.3, 130.5, 128.1, 126.6, 126.0, 125.0, 77.2, 65.1, 51.9, 45.2, 37.5, 16.9, 14.0;

IR (KBr): 2959, 2873, 1740, 1669, 1601, 1456, 1299, 1165, 769, 574 cm^{-1};

HRMS (ESI): $C_{19}H_{22}NaO_4$ $[M+Na]^+$ calcd: 337.1410, found: 337.1408.

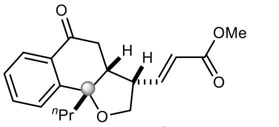

2c

Purified by silica gel chromatography (ethyl acetate/petroleum ether 4:1); White solid, m.p 78-80 °C; 14.4 mg, 43% yield; 98:2 *er* determined by HPLC on a Chiralpak IA-H column (hexane/2-propanol = 40/60, flow rate = 1.0 mL/min, t_{minor} = 5.2 min, t_{major} = 4.5 min);

$[\alpha]^{rt}_{D}$ = 99.4 (*c* = 0.96, $CHCl_3$);

^{1}H NMR (300 MHz, $CDCl_3$) δ 7.95 (d, *J* = 7.8 Hz, 1H), 7.64 (d, *J* = 3.7 Hz, 2H), 7.52 – 7.36 (m, 1H), 6.16 (dd, *J* = 15.6, 9.9 Hz, 1H), 5.73 (d, *J* = 15.6 Hz, 1H), 4.14 (dd, *J* = 9.1, 7.7 Hz, 1H), 3.64 (s, 3H), 3.49 (dd, *J* = 9.3, 5.6 Hz, 1H), 3.34 – 3.06 (m, 1H), 2.93 (dt, *J* = 9.4, 4.9 Hz, 1H), 2.86 – 2.74 (m, 2H), 1.96 – 1.80 (m, 2H), 1.39 (ddd, *J* = 27.4, 14.0, 8.1 Hz, 1H), 1.32 – 1.21 (m, 1H), 0.89 (t, *J* = 7.3 Hz, 3H);

^{13}C NMR (75 MHz, $CDCl_3$) δ 195.5, 165.8, 146.2, 146.1, 134.4, 132.0, 128.0, 127.0, 126.2, 123.9, 82.4, 70.1, 51.6, 45.8, 45.2, 43.7, 35.9, 17.6, 14.4;

IR (KBr): 2958, 2872, 1725, 1686, 1600, 1435, 1256, 1170, 769, 596 cm^{-1};

HRMS (ESI): $C_{19}H_{22}NaO_4$ $[M+Na]^+$ calcd: 337.1410, found: 337.1405.

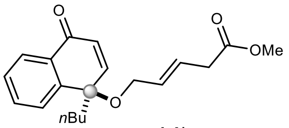

1d*

With 10 mol% catalyst at 40 °C for 17 h.

Purified by silica gel chromatography (ethyl acetate/petroleum ether 10:1); Colorless oil; 16.1 mg, 46% yield; 95:5 *er* determined by HPLC on a Chiralpak IC-H column (hexane/2-propanol = 40/60, flow rate = 1.0 mL/min, t_{minor} = 7.9 min, t_{major} = 7.1 min);

$[\alpha]^{rt}_{D}$ = -23.7 (*c* = 1.01, $CHCl_3$);

^{1}H NMR (300 MHz, $CDCl_3$) δ 8.12 (d, *J* = 7.7 Hz, 1H), 7.73 – 7.56 (m, 2H), 7.45 (dt, *J* = 8.2, 4.2 Hz, 1H), 6.91 (d, *J* = 10.3 Hz, 1H), 6.55 (d, *J* = 10.3 Hz, 1H), 6.03 – 5.45 (m, 2H), 3.95 – 3.64 (m, 4H), 3.55 (dd, *J* = 11.8, 5.4 Hz, 1H), 3.07 (d, *J* = 6.7 Hz, 2H), 2.16 – 1.89 (m, 2H), 1.24 – 0.99 (m, 3H), 0.95 – 0.79 (m, 1H), 0.73 (dd, *J* = 17.5, 10.2 Hz, 3H), 0.62 (dd, *J* = 12.8, 7.3 Hz, 1H);
^{13}C NMR (75 MHz, $CDCl_3$) δ 184.5, 171.8, 151.9, 144.2, 133.3, 132.2, 131.4, 130.4, 128.1, 126.6, 126.0, 125.1, 77.2, 65.2, 51.9, 42.7, 37.5, 25.6, 22.6, 13.8;
IR (KBr): 2955, 1740, 1669, 1601, 1456, 1298, 1167, 844, 768, 574 cm^{-1};
HRMS (ESI): $C_{20}H_{24}NaO_4$ $[M+Na]^+$ calcd: 351.1567, found: 351.1568.

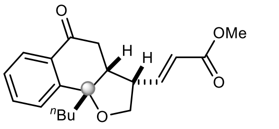

2d

Purified by silica gel chromatography (ethyl acetate/petroleum ether 4:1); Colorless oil; 15.0 mg, 43% yield; 95.5:4.5 *er* determined by HPLC on a Chiralpak IA-H column (hexane/2-propanol = 40/60, flow rate = 1.0 mL/min, t_{minor} = 4.8 min, t_{major} = 4.2 min);
$[\alpha]^{rt}_D$ = 94.0 (*c* = 0.98, $CHCl_3$);
^{1}H NMR (300 MHz, $CDCl_3$) δ 7.95 (d, *J* = 7.8 Hz, 1H), 7.65 (d, *J* = 3.8 Hz, 2H), 7.41 (dt, *J* = 8.3, 4.3 Hz, 1H), 6.17 (dd, *J* = 15.6, 9.9 Hz, 1H), 5.74 (d, *J* = 15.5 Hz, 1H), 4.14 (dd, *J* = 9.1, 7.7 Hz, 1H), 3.64 (s, 3H), 3.49 (dd, *J* = 9.3, 5.7 Hz, 1H), 3.35 – 3.20 (m, 1H), 2.93 (dt, *J* = 9.5, 4.9 Hz, 1H), 2.86 – 2.73 (m, 2H), 1.90 (t, *J* = 7.1 Hz, 2H), 1.42 – 1.15 (m, 4H), 0.86 (t, *J* = 7.0 Hz, 3H);
^{13}C NMR (75 MHz, $CDCl_3$) δ 195.6, 165.8, 146.2, 146.1, 134.5, 132.0, 128.0, 127.0, 126.2, 123.9, 82.4, 70.1, 51.6, 45.9, 45.1, 41.2, 36.0, 26.4, 23.1, 14.0;
IR (KBr): 2953, 1724, 1669, 1686, 1600, 1435, 1258, 1170, 990, 769, 596 cm^{-1};
HRMS (ESI): $C_{20}H_{24}NaO_4$ $[M+Na]^+$ calcd: 351.1567, found: 351.1566.

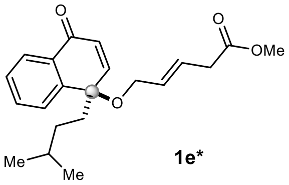

1e*

With 10 mol% catalyst at 40 °C for 17 h.
Purified by silica gel chromatography (ethyl acetate/petroleum ether 10:1); Colorless oil; 16.4 mg, 45% yield; 90.5:9.5 *er* determined by HPLC on a Chiralpak IC-H column (hexane/2-propanol = 40/60, flow rate = 1.0 mL/min, t_{minor} = 7.4 min, t_{major} = 6.6 min);
$[\alpha]^{rt}_D$ = -18.2 (*c* = 0.99, $CHCl_3$);
^{1}H NMR (300 MHz, $CDCl_3$) δ 8.13 (d, *J* = 7.8 Hz, 1H), 7.63 (d, *J* = 3.7 Hz, 2H), 7.50 – 7.31 (m, 1H), 6.89 (d, *J* = 10.3 Hz, 1H), 6.56 (d, *J* = 10.3 Hz, 1H), 5.90 – 5.44 (m, 2H), 3.84 – 3.64 (m, 4H), 3.54 (dd, *J* = 11.7, 5.2 Hz, 1H), 3.07 (d, *J* = 6.6 Hz, 2H), 2.16 – 1.84 (m, 2H), 1.36 (td, *J* = 13.3, 6.6 Hz, 1H), 1.00 (td, *J* = 11.8, 5.5 Hz, 1H), 0.74 (dd, *J* = 13.8, 6.6 Hz, 6H), 0.52 (tt, *J* = 12.3, 6.2 Hz, 1H);
^{13}C NMR (75 MHz, $CDCl_3$) δ 184.5, 171.8, 151.8, 144.1, 133.3, 132.2, 131.4, 130.4, 128.1, 126.5, 125.9, 125.1, 77.2, 65.2, 51.9, 40.8, 37.5, 32.2, 28.0, 22.4, 22.3;
IR (KBr): 2954, 1741, 1670, 1601, 1456, 1298, 1166, 1081, 769, 574 cm^{-1};

HRMS (ESI): $C_{21}H_{26}NaO_4$ $[M+Na]^+$ calcd: 365.1723, found: 365.1723.

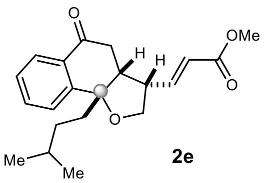

2e

Purified by silica gel chromatography (ethyl acetate/petroleum ether 4:1); Colorless oil; 14.2 mg, 39% yield; 96:4 *er* determined by HPLC on a Chiralpak IA-H column (hexane/2-propanol = 40/60, flow rate = 1.0 mL/min, t_{minor} = 4.7 min, t_{major} = 4.1 min);

$[\alpha]^{rt}_D$ = 81.2 (*c* = 1.01, $CHCl_3$);

^{1}H NMR (300 MHz, $CDCl_3$) δ 7.96 (d, *J* = 7.8 Hz, 1H), 7.65 (d, *J* = 3.8 Hz, 2H), 7.42 (dt, *J* = 8.2, 4.3 Hz, 1H), 6.18 (dd, *J* = 15.6, 9.9 Hz, 1H), 5.74 (d, *J* = 15.6 Hz, 1H), 4.14 (dd, *J* = 9.0, 7.8 Hz, 1H), 3.64 (s, 3H), 3.49 (dd, *J* = 9.2, 5.7 Hz, 1H), 3.35 – 3.18 (m, 1H), 2.93 (dt, *J* = 9.5, 4.9 Hz, 1H), 2.80 (d, *J* = 4.6 Hz, 2H), 1.97 – 1.82 (m, 2H), 1.47 (td, *J* = 13.2, 6.6 Hz, 1H), 1.29 (m, *J* = 10.9, 6.9 Hz, 1H), 1.16 – 1.04 (m, 1H), 0.84 (t, *J* = 6.4 Hz, 6H);

^{13}C NMR (75 MHz, $CDCl_3$) δ 195.6, 165.8, 146.2, 146.1[7], 134.5, 132.0, 128.0, 126.9, 126.1, 123.9, 82.5, 70.1, 51.6, 45.9, 45.0, 39.3, 36.0, 33.0, 28.4, 22.5, 22.4;

IR (KBr): 2953, 2869, 1725, 1687, 1600, 1274, 1170, 995, 768, 596 cm^{-1};

HRMS (ESI): $C_{21}H_{26}NaO_4$ $[M+Na]^+$ calcd: 365.1723, found: 365.1720.

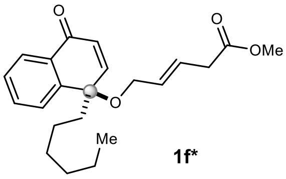

1f*

With 20 mol% catalyst at 40 °C for 12 h.

Purified by silica gel chromatography (ethyl acetate/petroleum ether 10:1); Colorless oil; 15.1 mg, 40% yield; 93:7 *er* determined by HPLC on a Chiralpak IC-H column (hexane/2-propanol = 40/60, flow rate = 1.0 mL/min, t_{minor} = 14.4 min, t_{major} = 13.2 min);

$[\alpha]^{rt}_D$ = -24.7 (*c* = 1.13, $CHCl_3$);

^{1}H NMR (300 MHz, $CDCl_3$) δ 8.12 (d, *J* = 7.8 Hz, 1H), 7.63 (d, *J* = 3.8 Hz, 2H), 7.45 (dt, *J* = 8.2, 4.2 Hz, 1H), 6.91 (d, *J* = 10.3 Hz, 1H), 6.55 (d, *J* = 10.3 Hz, 1H), 5.87 – 5.50 (m, 2H), 3.82 – 3.62 (m, 4H), 3.55 (dd, *J* = 11.8, 5.1 Hz, 1H), 3.07 (d, *J* = 6.7 Hz, 2H), 1.96 (d, *J* = 7.8 Hz, 2H), 1.14 (d, *J* = 14.2 Hz, 7H), 0.79 (t, *J* = 6.6 Hz, 3H), 0.73 – 0.52 (m, 1H);

^{13}C NMR (75 MHz, $CDCl_3$) δ 184.5, 171.8, 151.9, 144.2, 133.2, 132.2, 131.3, 130.5, 128.1, 126.5, 126.0, 125.0, 77.2, 65.2, 51.8, 42.9, 37.5, 31.4, 29.2, 23.4, 22.4, 14.0;

IR (KBr): 2928, 1932, 1741, 1670, 1601, 1298, 1164, 971, 767, 574 cm^{-1};

HRMS (ESI): $C_{22}H_{28}NaO_4$ $[M+Na]^+$ calcd: 379.1880, found: 379.1881.

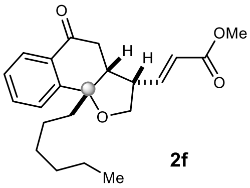

2f

Purified by silica gel chromatography (ethyl acetate/petroleum ether 4:1); Colorless oil; 15.9 mg, 42% yield; 95.5:4.5 *er* determined by HPLC on a Chiralpak IA-H column (hexane/2-propanol = 40/60, flow rate = 1.0 mL/min, t_{minor} = 9.4 min, t_{major} = 8.2 min);

$[\alpha]^{rt}_{D}$ = 93.4 (*c* = 0.96, $CHCl_3$);

^{1}H NMR (300 MHz, $CDCl_3$) δ 7.95 (d, *J* = 7.8 Hz, 1H), 7.64 (d, *J* = 3.8 Hz, 2H), 7.41 (dt, *J* = 8.3, 4.3 Hz, 1H), 6.17 (dd, *J* = 15.6, 9.9 Hz, 1H), 5.73 (d, *J* = 15.6 Hz, 1H), 4.13 (dd, *J* = 9.0, 7.7 Hz, 1H), 3.64 (s, 3H), 3.49 (dd, *J* = 9.2, 5.6 Hz, 1H), 3.36 – 3.18 (m, 1H), 2.93 (dt, *J* = 9.4, 4.8 Hz, 1H), 2.80 (d, *J* = 4.1 Hz, 2H), 1.90 (t, *J* = 6.9 Hz, 2H), 1.36 (d, *J* = 9.4 Hz, 1H), 1.24 (m, *J* = 7.6 Hz, 7H), 0.84 (t, *J* = 6.1 Hz, 3H);

^{13}C NMR (75 MHz, $CDCl_3$) δ 195.5, 165.8, 146.2, 134.5, 132.0, 128.0, 127.0, 126.2, 123.9, 82.4, 70.1, 51.5, 45.9, 45.1, 41.5, 36.0, 31.6, 29.6, 24.2, 22.5, 14.0;

IR (KBr): 2931, 1725, 1687, 1655, 1600, 1276, 1170, 1042, 768, 596 cm^{-1};

HRMS (ESI): $C_{22}H_{28}NaO_4$ $[M+Na]^+$ calcd: 379.1880, found: 379.1883.

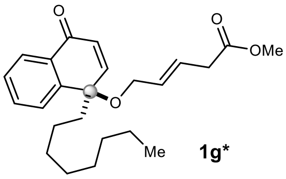

1g*

With 10 mol% catalyst at 40 °C for 9 h.

Purified by silica gel chromatography (ethyl acetate/petroleum ether 10:1); Colorless oil; 18.7 mg, 46% yield; 92:8 *er* determined by HPLC on a Chiralpak IC-H column (hexane/2-propanol = 40/60, flow rate = 1.0 mL/min, t_{minor} = 7.5 min, t_{major} = 6.8 min);

$[\alpha]^{rt}_{D}$ = -13.9 (*c* = 1.01, $CHCl_3$);

^{1}H NMR (300 MHz, $CDCl_3$) δ 8.12 (d, *J* = 7.8 Hz, 1H), 7.62 (t, *J* = 6.1 Hz, 2H), 7.51 – 7.32 (m, 1H), 6.91 (d, *J* = 10.4 Hz, 1H), 6.55 (d, *J* = 10.3 Hz, 1H), 5.93 – 5.42 (m, 2H), 3.78 – 3.61 (m, 4H), 3.55 (dd, *J* = 11.8, 5.4 Hz, 1H), 3.07 (d, *J* = 6.7 Hz, 2H), 1.94 (t, *J* = 8.6 Hz, 2H), 1.33 – 1.05 (m, 12H), 0.83 (t, *J* = 6.8 Hz, 3H);

^{13}C NMR (75 MHz, $CDCl_3$) δ 184.5, 171.8, 151.9, 144.2, 133.2, 132.2, 131.3, 130.5, 128.1, 126.6, 126.0, 125.0, 77.2, 65.2, 51.9, 42.9, 37.5, 31.7, 29.5, 29.2, 29.1, 23.4, 22.6, 14.1;

IR (KBr): 2926, 1958, 1742, 1670, 1602, 1298, 1166, 971, 768, 574 cm^{-1};

HRMS (ESI): $C_{24}H_{32}NaO_4$ $[M+Na]^+$ calcd: 407.2193, found: 407.2191.

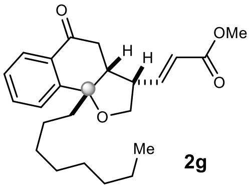

2g

Purified by silica gel chromatography (ethyl acetate/petroleum ether 4:1); Colorless oil; 17.1 mg, 42% yield; 98:2 *er* determined by HPLC on a Chiralpak IA-H column (hexane/2-propanol = 40/60, flow rate = 1.0 mL/min, t_{minor} = 4.9 min, t_{major} = 4.3 min);

$[\alpha]^{rt}_{D}$ = 87.4 (*c* = 1.00, $CHCl_3$);

^{1}H NMR (300 MHz, $CDCl_3$) δ 7.95 (d, *J* = 7.8 Hz, 1H), 7.64 (d, *J* = 3.8 Hz, 2H), 7.41 (dt, *J* = 8.1, 4.2 Hz, 1H), 6.17 (dd, *J* = 15.5, 9.9 Hz, 1H), 5.73 (d, *J* = 15.6 Hz, 1H), 4.27 – 4.05 (m, 1H), 3.66 (d, *J* = 11.1 Hz, 3H), 3.49 (dd, *J* = 9.2, 5.7 Hz, 1H), 3.35 – 3.14 (m, 1H), 2.93 (dt, *J* = 9.4, 4.8 Hz, 1H), 2.80 (d, *J* = 4.2 Hz, 2H), 1.91 (d, *J* = 6.8 Hz, 2H), 1.22 (m, 12H), 0.86 (t, *J* = 6.5 Hz, 3H);

^{13}C NMR (75 MHz, $CDCl_3$) δ 195.5, 165.7, 146.2, 134.4, 132.0, 128.0, 127.0, 126.2, 123.9, 82.4, 70.1, 51.5, 45.9, 45.1, 41.5, 36.0, 31.8, 30.0, 29.4, 29.2, 24.2, 22.6, 14.1;

IR (KBr): 2928, 2855, 1726, 1688, 1655, 1600, 1436, 1275, 1170, 989, 768 cm^{-1};

HRMS (ESI): $C_{24}H_{32}NaO_4$ $[M+Na]^+$ calcd: 407.2193, found: 407.2195.

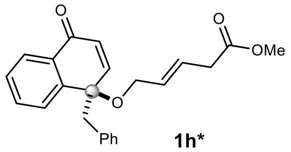

1h*

With 10 mol% catalyst at 40 °C for 12 h.

Purified by silica gel chromatography (ethyl acetate/petroleum ether 10:1); Colorless oil; 17.7 mg, 46% yield; 95:5 *er* determined by HPLC on a Chiralpak IC-H column (hexane/2-propanol = 40/60, flow rate = 1.0 mL/min, t_{minor} = 9.6 min, t_{major} = 8.3 min);

$[\alpha]^{rt}_{D}$ = 64.0 (*c* = 0.97, $CHCl_3$);

^{1}H NMR (300 MHz, $CDCl_3$) δ 7.99 (d, *J* = 7.8 Hz, 1H), 7.90 – 7.59 (m, 2H), 7.44 (t, *J* = 7.4 Hz, 1H), 7.08 (q, *J* = 6.5 Hz, 3H), 6.88 (d, *J* = 10.3 Hz, 1H), 6.76 (d, *J* = 6.6 Hz, 2H), 6.40 (d, *J* = 10.4 Hz, 1H), 5.72 (tdd, *J* = 20.7, 15.1, 6.0 Hz, 2H), 3.98 – 3.53 (m, 5H), 3.22 (dd, *J* = 35.3, 13.0 Hz, 2H), 3.09 (d, *J* = 6.6 Hz, 2H);

^{13}C NMR (75 MHz, $CDCl_3$) δ 183.8, 171.9, 151.1, 143.7, 134.6, 132.9, 132.2, 131.3, 130.6, 130.4, 128.3, 127.7, 126.9, 126.5, 126.4, 124.8, 77.7, 65.4, 51.9, 50.0, 37.6;

IR (KBr): 2951, 2925, 1956, 1738, 1669, 1601, 1454, 1299, 768, 573 cm^{-1};

HRMS (ESI): $C_{23}H_{22}NaO_4$ $[M+Na]^+$ calcd: 385.1410, found: 385.1407.

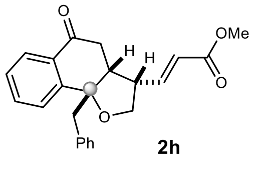

2h

Purified by silica gel chromatography (ethyl acetate/petroleum ether 4:1); Colorless oil; 16.5 mg, 43% yield; 97:3 *er* determined by HPLC on a Chiralpak IA-H column (hexane/2-propanol = 40/60, flow rate = 1.0 mL/min, t_{minor} = 5.4 min, t_{major} = 4.8 min);

$[\alpha]^{rt}_{D}$ = 53.1 (*c* = 0.98, $CHCl_3$);

^{1}H NMR (300 MHz, $CDCl_3$) δ 7.94 (d, *J* = 7.8 Hz, 1H), 7.65 (d, *J* = 13.3 Hz, 2H), 7.44 (dd, *J* = 9.5, 4.1 Hz, 1H), 7.24 (d, *J* = 12.2 Hz, 3H), 7.04 (s, 2H), 6.12 (dd, *J* = 15.5, 10.1 Hz, 1H), 5.66 (d, *J* = 15.5 Hz, 1H), 4.10 – 3.85 (m, 1H), 3.60 (s, 3H), 3.47 (dd, *J* =

9.2, 4.8 Hz, 1H), 3.16 (dt, *J* = 32.3, 16.2 Hz, 3H), 2.81 (t, *J* = 7.3 Hz, 1H), 2.66 (d, *J* = 18.5 Hz, 1H), 2.46 (dd, *J* = 18.5, 6.9 Hz, 1H);

^{13}C NMR (75 MHz, $CDCl_3$) δ 195.0, 165.7, 146.2, 135.9, 134.5, 132.0, 130.3, 128.3, 128.2, 127.2, 127.0, 126.1, 123.9, 82.2, 70.1, 51.5, 47.7, 45.9, 44.5, 35.7;

IR (KBr): 2949, 1958, 1724, 1686, 1600, 1255, 1171, 856, 704, 658 cm^{-1};

HRMS (ESI): $C_{23}H_{22}NaO_4$ [M+Na]$^+$ calcd: 385.1410, found: 385.1408.

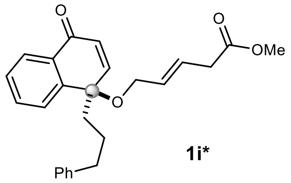

1i*

With 10 mol% catalyst at 40 °C for 19 h.

Purified by silica gel chromatography (ethyl acetate/petroleum ether 10:1); Colorless oil; 17.3 mg, 42% yield; 89.5:10.5 *er* determined by HPLC on a Chiralpak IC-H column (hexane/2-propanol = 40/60, flow rate = 1.0 mL/min, t_{minor} = 10.7 min, t_{major} = 9.3 min);

$[\alpha]^{rt}_D$ = -3.0 (*c* = 0.99, $CHCl_3$);

^{1}H NMR (300 MHz, $CDCl_3$) δ 8.11 (d, *J* = 7.7 Hz, 1H), 7.75 – 7.52 (m, 2H), 7.44 (dd, *J* = 10.4, 4.0 Hz, 1H), 7.29 – 7.09 (m, 3H), 7.00 (d, *J* = 7.0 Hz, 2H), 6.87 (d, *J* = 10.3 Hz, 1H), 6.54 (d, *J* = 10.3 Hz, 1H), 5.87 – 5.50 (m, 2H), 3.78 – 3.57 (m, 4H), 3.52 (dd, *J* = 11.6, 5.6 Hz, 1H), 3.05 (d, *J* = 6.6 Hz, 2H), 2.60 – 2.32 (m, 2H), 2.09 – 1.85 (m, 2H), 1.56 – 1.34 (m, 1H), 1.12 – 0.90 (m, 1H);

^{13}C NMR (75 MHz, $CDCl_3$) δ 184.4, 171.8, 151.6, 144.0, 141.4, 133.3, 132.2, 131.5, 130.4, 128.3, 128.2, 126.6, 126.0, 125.9, 125.1, 77.0, 65.2, 51.9, 42.3, 37.5, 35.6, 25.2;

IR (KBr): 2948, 1956, 1739, 1668, 1601, 1455, 1298, 1081, 769, 574 cm^{-1};

HRMS (ESI): $C_{25}H_{26}NaO_4$ [M+Na]$^+$ calcd: 413.1723, found: 413.1724.

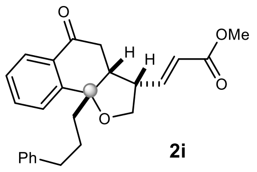

2i

Purified by silica gel chromatography (ethyl acetate/petroleum ether 4:1); Colorless oil; 14.5 mg, 40% yield; 97:3 *er* determined by HPLC on a Chiralpak IA-H column (hexane/2-propanol = 40/60, flow rate = 1.0 mL/min, t_{minor} = 5.8 min, t_{major} = 5.2 min);

$[\alpha]^{rt}_D$ = 70.6 (*c* = 0.99, $CHCl_3$);

^{1}H NMR (300 MHz, $CDCl_3$) δ 7.94 (d, *J* = 7.8 Hz, 1H), 7.69 – 7.56 (m, 2H), 7.40 (ddd, *J* = 8.3, 5.0, 3.5 Hz, 1H), 7.25 (t, *J* = 7.1 Hz, 2H), 7.22 – 7.05 (m, 3H), 6.14 (dd, *J* = 15.6, 9.9 Hz, 1H), 5.71 (d, *J* = 15.6 Hz, 1H), 4.19 – 4.05 (m, 1H), 3.63 (s, 3H), 3.48 (dd, *J* = 9.2, 5.6 Hz, 1H), 3.38 – 3.11 (m, 1H), 2.90 (dd, *J* = 9.4, 4.8 Hz, 1H), 2.76 (d, *J* = 4.7 Hz, 2H), 2.57 (dd, *J* = 11.2, 6.8 Hz, 2H), 2.02 – 1.87 (m, 2H), 1.73 (dd, *J* = 17.7, 11.8 Hz, 1H), 1.66 – 1.47 (m, 1H);

^{13}C NMR (75 MHz, $CDCl_3$) δ 195.4, 165.8, 146.1, 145.9, 141.7, 134.5, 131.9, 128.4, 128.3[5], 128.1, 127.0, 126.2, 125.9, 123.9, 82.3, 70.2, 51.6, 45.7, 45.2, 40.8, 36.0, 35.9, 26.1;

IR (KBr): 2946, 1955, 1724, 1686, 1600, 1452, 1276, 1043, 701, 592 cm^{-1};

HRMS (ESI): $C_{25}H_{26}NaO_4$ $[M+Na]^+$ calcd: 413.1723, found: 413.1721.

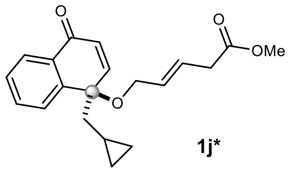

1j*

With 10 mol% catalyst at 40 °C for 11 h.

Purified by silica gel chromatography (ethyl acetate/petroleum ether 10:1); Colorless oil; 14.6 mg, 42% yield; 98:2 *er* determined by HPLC on a Chiralpak IC-H column (hexane/2-propanol = 40/60, flow rate = 1.0 mL/min, t_{minor} = 9.1 min, t_{major} = 7.5 min);

$[\alpha]^{rt}_D$ = -16.5 (*c* = 0.98, $CHCl_3$);

^{1}H NMR (300 MHz, $CDCl_3$) δ 8.20 (d, *J* = 7.7 Hz, 1H), 7.78 – 7.58 (m, 2H), 7.58 – 7.46 (m, 1H), 7.10 (d, *J* = 10.3 Hz, 1H), 6.63 (d, *J* = 10.3 Hz, 1H), 5.94 – 5.43 (m, 2H), 3.95 – 3.56 (m, 5H), 3.14 (d, *J* = 6.7 Hz, 2H), 2.14 (dd, *J* = 13.5, 5.7 Hz, 1H), 1.93 – 1.70 (m, 1H), 0.39 (dt, *J* = 13.5, 8.1 Hz, 2H), 0.24 – 0.12 (m, 1H), 0.01 (m, *J* = 9.1, 4.9 Hz, 1H), -0.32 (m, *J* = 9.7, 5.0 Hz, 1H);

^{13}C NMR (75 MHz, $CDCl_3$) δ 184.5, 171.8, 152.2, 144.6, 133.0, 132.3, 131.1, 130.5, 128.0, 126.5, 126.1, 124.9, 77.6, 65.1, 51.8, 48.4, 37.5, 5.8, 4.7, 4.5;

IR (KBr): 2926, 1959, 1740, 1669, 1601, 1300, 1165, 843, 767, 574 cm^{-1};

HRMS (ESI): $C_{20}H_{22}NaO_4$ $[M+Na]^+$ calcd: 349.1410, found: 349.1412.

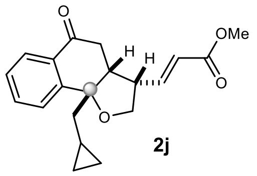

2j

Purified by silica gel chromatography (ethyl acetate/petroleum ether 4:1); White solid, m.p 75-77 °C; 15.3 mg, 44% yield; 92:8 *er* determined by HPLC on a Chiralpak IA-H column (hexane/2-propanol = 40/60, flow rate = 1.0 mL/min, t_{minor} = 5.3 min, t_{major} = 4.6 min);

$[\alpha]^{rt}_D$ = 90.0 (*c* = 1.03, $CHCl_3$);

^{1}H NMR (300 MHz, $CDCl_3$) δ 7.93 (d, *J* = 7.7 Hz, 1H), 7.62 (q, *J* = 7.4 Hz, 2H), 7.38 (t, *J* = 7.3 Hz, 1H), 6.15 (dd, *J* = 15.5, 9.9 Hz, 1H), 5.70 (d, *J* = 15.6 Hz, 1H), 4.24 – 3.98 (m, 1H), 3.60 (s, 3H), 3.46 (dd, *J* = 9.2, 5.4 Hz, 1H), 3.33 – 3.18 (m, 1H), 3.18 – 2.98 (m, 1H), 2.84 (qd, *J* = 18.4, 4.6 Hz, 2H), 1.92 (dd, *J* = 14.3, 6.4 Hz, 1H), 1.75 (dd, *J* = 14.3, 7.0 Hz, 1H), 0.60 (m, *J* = 12.6, 6.1 Hz, 1H), 0.48 – 0.27 (m, 2H), 0.11 – -0.24 (m, 2H);

^{13}C NMR (75 MHz, $CDCl_3$) δ 195.6, 165.8, 146.4, 146.3, 134.4, 132.1, 128.0, 127.0, 126.1, 123.9, 82.8, 70.1, 51.5, 46.6, 46.1, 45.0, 36.1, 6.3, 4.9;

IR (KBr): 2924, 1848, 1724, 1685, 1599, 1435, 1257, 1021, 768, 595 cm^{-1};

HRMS (ESI): $C_{20}H_{22}NaO_4$ $[M+Na]^+$ calcd: 349.1410, found: 349.1410.

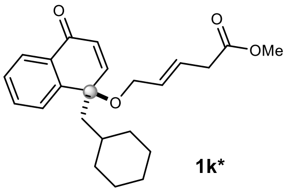

1k*

With 10 mol% catalyst at 40 °C for 11 h.

Purified by silica gel chromatography (ethyl acetate/petroleum ether 10:1); Colorless oil; 16.4 mg, 42% yield; 92.5:7.5 *er* determined by HPLC on a Chiralpak IC-H column (hexane/2-propanol = 40/60, flow rate = 1.0 mL/min, t_{minor} = 7.9 min, t_{major} = 7.0 min);

$[\alpha]^{rt}_{D}$ = -16.2 (*c* = 0.99, $CHCl_3$);

^{1}H NMR (300 MHz, $CDCl_3$) δ 8.13 (d, *J* = 7.7 Hz, 1H), 7.73 – 7.55 (m, 2H), 7.53 – 7.38 (m, 1H), 6.93 (d, *J* = 10.3 Hz, 1H), 6.56 (d, *J* = 10.3 Hz, 1H), 5.83 – 5.44 (m, 2H), 3.74 – 3.60 (m, 4H), 3.49 (dd, *J* = 11.8, 5.5 Hz, 1H), 3.06 (d, *J* = 6.7 Hz, 2H), 1.91 (dd, *J* = 14.3, 4.5 Hz, 2H), 1.61 (m, *J* = 25.4, 11.2 Hz, 2H), 1.43 (m, *J* = 19.1, 10.0 Hz, 2H), 0.99 (m, *J* = 33.6, 16.9 Hz, 6H), 0.65 (t, *J* = 10.2 Hz, 1H);

^{13}C NMR (75 MHz, $CDCl_3$) δ184.5, 171.8, 151.9, 144.4, 133.1, 132.0, 131.1, 130.5, 128.1, 126.6, 126.5, 124.8, 76.9, 64.7, 51.8, 50.4, 37.5, 35.0, 34.3, 33.2, 26.0[3], 26.0;

IR (KBr): 2924, 1982, 1741, 1669, 1601, 1299, 1164, 843, 767, 573 cm^{-1};

HRMS (ESI): $C_{23}H_{28}NaO_4$ $[M+Na]^+$ calcd: 391.1880, found: 391.1889.

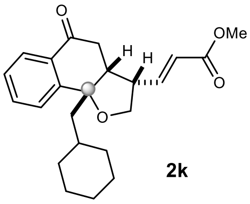

2k

Purified by silica gel chromatography (ethyl acetate/petroleum ether 4:1); White solid, m.p 61-63 °C; 15.6 mg, 40% yield; 95:5 *er* determined by HPLC on a Chiralpak IA-H column (hexane/2-propanol = 40/60, flow rate = 1.0 mL/min, t_{minor} = 4.8 min, t_{major} = 4.3 min);

$[\alpha]^{rt}_{D}$ = 99.3 (*c* = 0.98, $CHCl_3$);

^{1}H NMR (300 MHz, $CDCl_3$) δ 7.95 (d, *J* = 7.8 Hz, 1H), 7.71 – 7.55 (m, 2H), 7.46 – 7.34 (m, 1H), 6.12 (dd, *J* = 15.5, 9.9 Hz, 1H), 5.72 (d, *J* = 15.6 Hz, 1H), 4.27 – 4.04 (m, 1H), 3.63 (s, 3H), 3.44 (dd, *J* = 9.3, 5.6 Hz, 1H), 3.35 – 3.18 (m, 1H), 2.94 (dt, *J* = 9.5, 4.7 Hz, 1H), 2.80 (d, *J* = 4.6 Hz, 2H), 1.77 (m, *J* = 30.9, 14.7, 5.4 Hz, 3H), 1.63 (m, *J* = 15.1 Hz, 4H), 1.40 (m, 1H), 1.24 – 1.10 (m, 3H), 0.94 (m, *J* = 21.6, 13.0 Hz, 2H);

^{13}C NMR (75 MHz, $CDCl_3$) δ 195.5, 165.8, 146.7, 146.5, 134.4, 131.8, 128.0, 127.0, 126.3, 123.9, 82.8, 70.0, 51.5, 48.1, 45.4, 45.1, 35.8, 35.1, 34.9, 33.7, 26.3, 26.1;

IR (KBr): 3356, 2922, 1845, 1725, 1687, 1600, 1450, 1281, 1170, 768, 589 cm^{-1};

HRMS (ESI): $C_{23}H_{28}NaO_4$ $[M+Na]^+$ calcd: 391.1880, found: 391.1886.

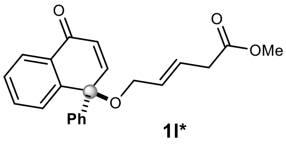

1l*

With 20 mol% catalyst at 40 °C for 10 h.

Purified by silica gel chromatography (ethyl acetate/petroleum ether 10:1); Colorless oil; 17.0 mg, 46% yield; 91.5:8.5 *er* determined by HPLC on a Chiralpak IC-H column (hexane/2-propanol = 40/60, flow rate = 1.0 mL/min, t_{minor} = 16.3 min, t_{major} = 14.2 min);

$[\alpha]^{rt}_D$ = 69.8 (c = 0.92, $CHCl_3$);

^{1}H NMR (300 MHz, $CDCl_3$) δ 8.17 (d, J = 7.8 Hz, 1H), 7.53 (t, J = 7.5 Hz, 1H), 7.44 (t, J = 7.5 Hz, 1H), 7.41 – 7.32 (m, 3H), 7.32 – 7.21 (m, 3H), 6.90 (d, J = 10.2 Hz, 1H), 6.52 (d, J = 10.2 Hz, 1H), 5.94 – 5.57 (m, 2H), 3.96 (dd, J = 11.7, 4.9 Hz, 1H), 3.84 – 3.64 (m, 4H), 3.10 (d, J = 6.7 Hz, 2H);

^{13}C NMR (75 MHz, $CDCl_3$) δ 184.7, 171.9, 151.3, 144.4, 141.9, 133.5, 131.8, 130.4, 129.2, 128.5, 128.4, 127.7, 126.4, 126.1, 124.6, 64.8, 51.9, 37.6 18.4;

IR (KBr): 2925, 1959, 1739, 1668, 1600, 1298, 1166, 761, 630, 536 cm^{-1};

HRMS (ESI): $C_{22}H_{20}NaO_4$ $[M+Na]^+$ calcd: 371.1254, found: 371.1257.

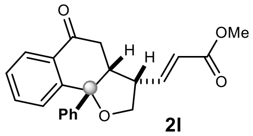

2l

Purified by silica gel chromatography (ethyl acetate/petroleum ether 4:1); White solid, m.p 130-132 °C; 15.5 mg, 42% yield; 95:5 *er* determined by HPLC on a Chiralpak IA-H column (hexane/2-propanol = 40/60, flow rate = 1.0 mL/min, t_{minor} = 12.8 min, t_{major} = 12.1 min);

$[\alpha]^{rt}_D$ = 163.6 (c = 1.07, $CHCl_3$);

^{1}H NMR (300 MHz, $CDCl_3$) δ 8.01 (d, J = 6.9 Hz, 1H), 7.50 (dd, J = 10.7, 4.2 Hz, 1H), 7.41 (t, J = 7.5 Hz, 1H), 7.32 (dt, J = 16.2, 6.4 Hz, 5H), 7.14 (d, J = 7.4 Hz, 1H), 6.62 (dd, J = 15.6, 8.9 Hz, 1H), 5.80 (d, J = 15.7 Hz, 1H), 4.39 (t, J = 8.5 Hz, 1H), 3.98 (t, J = 8.4 Hz, 1H), 3.70 (s, 3H), 3.41 – 3.22 (m, 1H), 3.12 (m, J = 14.3, 7.4 Hz, 1H), 2.78 (m, J = 7.0, 3.7 Hz, 2H);

^{13}C NMR (75 MHz, $CDCl_3$) δ196.5, 165.9, 145.1, 144.5, 144.1, 134.6, 132.0, 130.0, 128.4, 127.5, 125.8^2, 125.8, 124.2, 85.5, 70.7, 51.7, 50.4, 44.7, 36.1;

IR (KBr): 2925, 1724, 1691, 1599, 1446, 1265, 1054, 951, 610, 520 cm^{-1};

HRMS (ESI): $C_{22}H_{20}NaO_4$ $[M+Na]^+$ calcd: 371.1254, found: 371.1255.

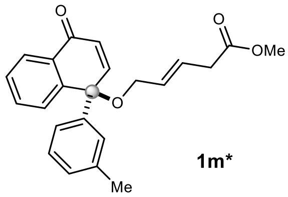

1m*

With 20 mol% catalyst at 40 °C for 10 h.

Purified by silica gel chromatography (ethyl acetate/petroleum ether 10:1); Colorless oil; 17.7 mg, 46% yield; 91.5:8.5 *er* determined by HPLC on a Chiralpak IC-H column (hexane/2-propanol = 40/60, flow rate = 1.0 mL/min, t_{minor} = 17.3 min, t_{major} = 15.3 min);

$[\alpha]^{rt}_D$ = 79.0 (c = 1.04, $CHCl_3$);

¹H NMR (300 MHz, $CDCl_3$) δ 8.17 (d, *J* = 7.6 Hz, 1H), 7.53 (t, *J* = 7.4 Hz, 1H), 7.42 (dd, *J* = 19.2, 7.6 Hz, 2H), 7.15 (s, 3H), 7.05 (s, 1H), 6.89 (d, *J* = 10.2 Hz, 1H), 6.51 (m, *J* = 10.2 Hz, 1H), 5.78 (ddd, *J* = 36.2, 15.2, 8.4 Hz, 2H), 3.95 (dd, *J* = 11.9, 5.2 Hz, 1H), 3.72 (m, *J* = 15.1 Hz, 4H), 3.10 (d, *J* = 6.5 Hz, 2H), 2.29 (s, 3H);

¹³C NMR (75 MHz, $CDCl_3$) δ 184.8, 171.9, 151.4, 144.5, 141.8, 138.2, 133.4, 131.8, 130.5, 129.1, 128.5, 128.4, 128.3, 126.6, 126.4, 124.6, 123.2, 64.8, 51.9, 37.6, 21.6;

IR (KBr): 2923, 1739, 1668, 1601, 1455, 1299, 1050, 768, 634, 512 cm^{-1};

HRMS (ESI): $C_{23}H_{22}NaO_4$ $[M+Na]^+$ calcd: 385.1410, found: 385.1410.

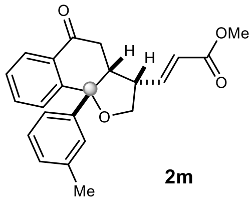

2m

Purified by silica gel chromatography (ethyl acetate/petroleum ether 4:1); White solid, m.p 86-88 °C; 17.3 mg, 45% yield; 94.5:5.5 *er* determined by HPLC on a Chiralpak IA-H column (hexane/2-propanol = 40/60, flow rate = 1.0 mL/min, t_{minor} = 10.8 min, t_{major} = 10.0 min);

$[\alpha]^{rt}_D$ = 137.9 (*c* = 1.09, $CHCl_3$);

¹H NMR (300 MHz, $CDCl_3$) δ 8.01 (d, *J* = 7.8 Hz, 1H), 7.50 (dd, *J* = 10.7, 4.3 Hz, 1H), 7.41 (t, *J* = 7.5 Hz, 1H), 7.26 – 6.95 (m, 5H), 6.62 (dd, *J* = 15.6, 8.9 Hz, 1H), 5.80 (d, *J* = 15.7 Hz, 1H), 4.38 (t, *J* = 8.5 Hz, 1H), 3.97 (t, *J* = 8.4 Hz, 1H), 3.70 (s, 3H), 3.41 – 3.22 (m, 1H), 3.12 (dd, *J* = 14.4, 7.3 Hz, 1H), 2.84 – 2.67 (m, 2H), 2.33 (s, 3H);

¹³C NMR (75 MHz, $CDCl_3$) δ 196.6, 165.9, 145.0, 144.5, 144.2, 138.2, 134.6, 132.0, 130.0, 128.4, 128.3, 126.4, 125.7, 124.2, 123.0, 85.5, 70.7, 51.7, 50.4, 44.7, 36.2, 21.6;

IR (KBr): 2950, 1724, 1691, 1600, 1435, 1264, 1054, 778, 614, 558 cm^{-1};

HRMS (ESI): $C_{23}H_{22}NaO_4$ $[M+Na]^+$ calcd: 385.1410, found: 385.1411.

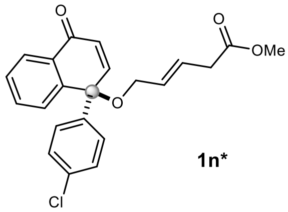

1n*

With 20 mol% catalyst at 40 °C for 36 h.

Purified by silica gel chromatography (ethyl acetate/petroleum ether 10:1); Colorless oil; 19.0 mg, 47% yield; 82.5:17.5 *er* determined by HPLC on a Chiralpak IC-H column (hexane/2-propanol = 40/60, flow rate = 1.0 mL/min, t_{minor} = 15.6 min, t_{major} = 13.7min);

$[\alpha]^{rt}_D$ = 50.6 (*c* = 1.09, $CHCl_3$);

¹H NMR (300 MHz, $CDCl_3$) δ 8.17 (d, *J* = 7.7 Hz, 1H), 7.54 (td, *J* = 7.6, 1.4 Hz, 1H), 7.46 (dd, *J* = 10.8, 4.3 Hz, 1H), 7.36 – 7.21 (m, 5H), 6.84 (d, *J* = 10.2 Hz, 1H), 6.53 (d, *J* = 10.2 Hz, 1H), 5.93 – 5.55 (m, 2H), 3.95 (dd, *J* = 12.0, 5.5 Hz, 1H), 3.76 – 3.62 (m, 4H), 3.10 (d, *J* = 6.7 Hz, 2H);

¹³C NMR (75 MHz, $CDCl_3$) δ184.4, 171.8, 150.7, 143.9, 140.6, 133.6[1], 133.6, 131.7, 130.2, 129.5, 128.7, 128.6, 128.3, 127.5, 126.5, 124.8, 64.8, 51.9, 37.5;

IR (KBr): 2925, 1738, 1670, 1600, 1488, 1378, 1297, 1092, 768, 631 cm^{-1};

HRMS (ESI): $C_{22}H_{19}ClNaO_4$ $[M+Na]^+$ calcd: 405.0864, found: 405.0868.

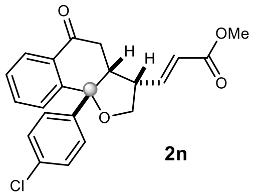

2n

Purified by silica gel chromatography (ethyl acetate/petroleum ether 4:1); Colorless oil; 17.0 mg, 42% yield; 92:8 *er* determined by HPLC on a Chiralpak IA-H column (hexane/2-propanol = 40/60, flow rate = 1.0 mL/min, t_{minor} = 21.4 min, t_{major} = 19.2 min);

$[\alpha]^{rt}_D$ = 99.7 (*c* = 0.96, $CHCl_3$);

^{1}H NMR (300 MHz, $CDCl_3$) δ 8.01 (d, *J* = 7.8 Hz, 1H), 7.52 (dd, *J* = 10.7, 4.4 Hz, 1H), 7.42 (dd, *J* = 10.8, 4.2 Hz, 1H), 7.31 (d, *J* = 8.6 Hz, 2H), 7.21 (d, *J* = 8.6 Hz, 2H), 7.13 (d, *J* = 7.7 Hz, 1H), 6.59 (dd, *J* = 15.6, 8.9 Hz, 1H), 5.81 (d, *J* = 15.6 Hz, 1H), 4.37 (t, *J* = 8.6 Hz, 1H), 3.97 (t, *J* = 8.4 Hz, 1H), 3.70 (s, 3H), 3.28 (p, *J* = 8.1 Hz, 1H), 3.06 (dd, *J* = 14.1, 7.5 Hz, 1H), 2.86 – 2.65 (m, 2H);

^{13}C NMR (75 MHz, $CDCl_3$) δ 196.0, 165.8, 144.1, 143.7, 143.6, 134.7, 133.5, 132.0, 129.8, 128.7, 128.6, 127.3, 125.9, 124.4, 85.1, 70.8, 51.7, 50.4, 44.7, 36.0;

IR (KBr): 2925, 1724, 1691, 1599, 1435, 1265, 1013, 767, 610, 526 cm^{-1};

HRMS (ESI): $C_{22}H_{19}ClNaO_4$ $[M+Na]^+$ calcd: 405.0864, found: 405.0868.

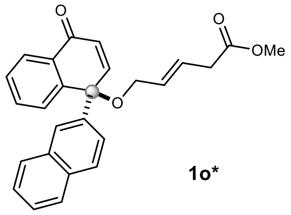

1o*

With 20 mol% catalyst at 40 °C for 45 h.

Purified by silica gel chromatography (ethyl acetate/petroleum ether 10:1); Colorless oil; 17.6 mg, 42% yield; 85:15 *er* determined by HPLC on a Chiralpak IC-H column (hexane/2-propanol = 40/60, flow rate = 1.0 mL/min, t_{minor} = 17.8 min, t_{major} = 15.3 min);

$[\alpha]^{rt}_D$ = 41.5 (*c* = 0.87, $CHCl_3$);

^{1}H NMR (300 MHz, $CDCl_3$) δ 8.26 – 8.10 (m, 1H), 8.04 (s, 1H), 7.85 – 7.73 (m, 2H), 7.69 (d, *J* = 8.7 Hz, 1H), 7.56 – 7.35 (m, 5H), 7.30 – 7.17 (m, 1H), 6.94 (d, *J* = 10.2 Hz, 1H), 6.57 (d, *J* = 10.2 Hz, 1H), 5.91 – 5.62 (m, 2H), 4.02 (dd, *J* = 12.0, 5.3 Hz, 1H), 3.85 – 3.62 (m, 4H), 3.13 (d, *J* = 6.7 Hz, 2H);

^{13}C NMR (75 MHz, $CDCl_3$) δ 185.7, 171.9, 151.1, 144.3, 139.0, 133.5, 133.2, 132.7, 131.9, 130.5, 129.5, 128.5, 128.4[5], 128.3, 128.2, 127.6, 126.5, 126.3, 124.9, 124.8, 124.0, 77.6, 65.0, 51.9, 37.6;

IR (KBr): 2925, 1932, 1736, 1668, 1600, 1455, 1298, 1122, 766, 571 cm^{-1};

HRMS (ESI): $C_{26}H_{22}NaO_4$ $[M+Na]^+$ calcd: 421.1410, found: 421.1412.

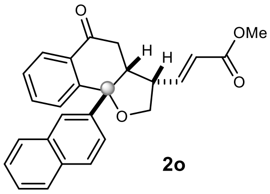

2o

Purified by silica gel chromatography (ethyl acetate/petroleum ether 4:1); Colorless oil; 19.4 mg, 46% yield; 87.5:12.5 *er* determined by HPLC on a Chiralpak IA-H column (hexane/2-propanol = 40/60, flow rate = 1.0 mL/min, t_{minor} = 14.7 min, t_{major} = 12.8 min);

$[\alpha]^{rt}_{D}$ = 105.9 (c = 0.96, $CHCl_3$);

^{1}H NMR (300 MHz, $CDCl_3$) δ 8.05 (d, J = 7.3 Hz, 1H), 7.90 – 7.79 (m, 4H), 7.56 – 7.41 (m, 4H), 7.24 (d, J = 10.9 Hz, 2H), 7.15 (d, J = 7.7 Hz, 1H), 6.68 (dd, J = 15.6, 8.7 Hz, 1H), 5.79 (d, J = 15.7 Hz, 1H), 4.48 (t, J = 8.5 Hz, 1H), 4.08 (t, J = 8.5 Hz, 1H), 3.70 (s, 3H), 3.41 – 3.14 (m, 2H), 2.82 (dd, J = 6.8, 4.6 Hz, 2H);

^{13}C NMR (75 MHz, $CDCl_3$) δ 196.5, 165.9, 144.2, 143.8, 142.3, 134.6, 132.8, 132.6, 132.0, 130.3, 128.6, 128.5, 128.2, 127.6, 126.6, 126.4, 125.9, 124.7, 124.3, 123.8, 85.8, 77.2, 70.9, 51.7, 50.1, 44.7, 36.2;

IR (KBr): 2926, 2360, 1724, 1691, 1599, 1353, 1265, 1054, 754, 605 cm^{-1};

HRMS (ESI): $C_{26}H_{22}NaO_4$ $[M+Na]^+$ calcd: 421.1410, found: 421.1411.

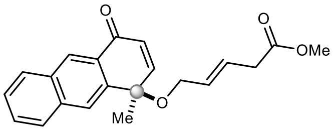

1p*

With 10 mol% catalyst at 40 °C for 12 h.

Purified by silica gel chromatography (ethyl acetate/petroleum ether 10:1); Colorless oil; 16.8 mg, 47% yield; 87.5:12.5 *er* determined by HPLC on a Chiralpak IC-H column (hexane/2-propanol = 40/60, flow rate = 1.0 mL/min, t_{minor} = 20.8 min, t_{major} = 17.8 min);

$[\alpha]^{rt}_{D}$ = -114.7 (c = 0.99, $CHCl_3$);

^{1}H NMR (300 MHz, $CDCl_3$) δ 8.72 (s, 1H), 8.13 (s, 1H), 8.02 (d, J = 8.0 Hz, 1H), 7.92 (d, J = 8.2 Hz, 1H), 7.59 (dt, J = 21.1, 7.3 Hz, 2H), 7.26 (s, 1H), 7.03 (d, J = 10.4 Hz, 1H), 6.56 (d, J = 10.4 Hz, 1H), 6.06 – 5.39 (m, 2H), 3.75 (dd, J = 11.7, 5.7 Hz, 1H), 3.70 – 3.49 (m, 4H), 3.06 (d, J = 6.6 Hz, 2H), 1.72 (s, 3H);

^{13}C NMR (75 MHz, $CDCl_3$) δ 184.6, 171.8, 152.9, 139.9, 135.4, 132.3, 130.5, 130.4, 129.8, 128.8, 128.6, 128.0, 127.0, 125.8, 125.1, 74.0, 65.4, 51.8, 37.5, 31.6;

IR (KBr): 2928, 1959, 1738, 1669, 1621, 1386, 1288, 1049, 753, 613 cm^{-1};

HRMS (ESI): $C_{21}H_{20}NaO_4$ $[M+Na]^+$ calcd: 359.1254, found: 359.1256.

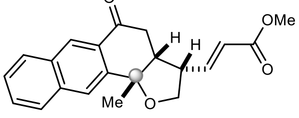

2p

Purified by silica gel chromatography (ethyl acetate/petroleum ether 4:1); Colorless oil; 15.8 mg, 44% yield; 95.5:4.5 *er* determined by HPLC on a Chiralpak IC-H column (hexane/2-propanol = 40/60, flow rate = 1.0 mL/min, t_{minor} = 37.6 min, t_{major} = 32.8 min);

$[\alpha]^{rt}_{D}$ = 211.9 (*c* = 1.06, $CHCl_3$);

^{1}H NMR (300 MHz, $CDCl_3$) δ 8.57 (s, 1H), 8.16 (s, 1H), 7.99 (d, *J* = 8.1 Hz, 1H), 7.90 (d, *J* = 8.0 Hz, 1H), 7.57 (dt, *J* = 14.8, 6.9 Hz, 2H), 6.17 (dd, *J* = 15.5, 10.0 Hz, 1H), 5.76 (d, *J* = 15.6 Hz, 1H), 4.22 (t, *J* = 8.6 Hz, 1H), 3.70 – 3.45 (m, 4H), 3.42 – 3.26 (m, 1H), 2.97 – 2.71 (m, 3H), 1.74 (s, 3H);

^{13}C NMR (75 MHz, $CDCl_3$) δ 195.6, 165.7, 146.1, 141.8, 136.5, 132.2, 130.0, 129.2, 128.8, 128.2, 127.8, 126.9, 126.1, 124.2, 80.7, 70.2, 51.5, 48.2, 45.6, 36.3, 29.2;

IR (KBr): 2927, 1723, 1689, 1626, 1447, 1272, 1045, 753, 622, 479 cm^{-1};

HRMS (ESI): $C_{21}H_{20}NaO_4$ $[M+Na]^+$ calcd: 359.1254, found: 359.1256.

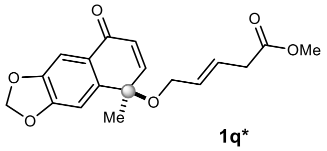

1q*

With 10 mol% catalyst at 40 °C for 12 h.

Purified by silica gel chromatography (ethyl acetate/petroleum ether 10:1); Colorless oil; 13.7 mg, 39% yield; 97:3 *er* determined by HPLC on a Chiralpak IC-H column (hexane/2-propanol = 40/60, flow rate = 1.0 mL/min, t_{minor} = 35.6 min, t_{major} = 23.7 min);

$[\alpha]^{rt}_{D}$ = -98.6 (*c* = 1.04, $CHCl_3$);

^{1}H NMR (300 MHz, $CDCl_3$) δ 7.52 (s, 1H), 7.06 (s, 1H), 6.89 (d, *J* = 10.2 Hz, 1H), 6.42 (d, *J* = 10.2 Hz, 1H), 6.07 (m, *J* = 4.1 Hz, 2H), 5.89 – 5.52 (m, 2H), 3.77 – 3.62 (m, 4H), 3.54 (dd, *J* = 11.7, 5.5 Hz, 1H), 3.08 (d, *J* = 6.7 Hz, 2H), 1.58 (s, 3H);

^{13}C NMR (75 MHz, $CDCl_3$) δ 182.9, 171.8, 152.3, 151.8, 148.1, 142.1, 130.3, 129.8, 126.6, 125.2, 105.6, 105.5[6], 101.9, 73.9, 65.4, 51.9, 37.5, 30.4;

IR (KBr): 2927, 1960, 1738, 1665, 1616, 1479, 1296, 1036, 720, 572 cm^{-1};

HRMS (ESI): $C_{18}H_{18}NaO_6$ $[M+Na]^+$ calcd: 353.0996, found: 353.0992.

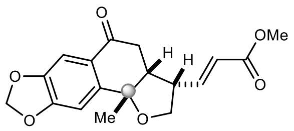

2q

Purified by silica gel chromatography (ethyl acetate/petroleum ether 4:1); White solid, m.p 93-95 °C; 17.0 mg, 48% yield; 88:12 *er* determined by HPLC on a Chiralpak IA-H column (hexane/2-propanol = 40/60, flow rate = 1.0 mL/min, t_{minor} = 11.9 min, t_{major} = 10.3 min);

$[\alpha]^{rt}_{D}$ = 128.2 (*c* = 1.01, $CHCl_3$);

^{1}H NMR (300 MHz, $CDCl_3$) δ 7.38 (s, 1H), 7.07 (s, 1H), 6.25 (dd, *J* = 15.5, 9.9 Hz, 1H), 6.05 (d, *J* = 4.0 Hz, 2H), 5.75 (d, *J* = 15.5 Hz, 1H), 4.15 (t, *J* = 8.6 Hz, 1H), 3.66 (s, 3H), 3.41 (dd, *J* = 9.2, 6.1 Hz, 1H), 3.29 (dd, *J* = 15.3, 7.8 Hz, 1H), 2.77 (s, 3H), 1.62 (s, 3H);

^{13}C NMR (75 MHz, $CDCl_3$) δ 193.2, 165.8, 153.3, 148.1, 146.3, 144.0, 126.9, 124.0, 105.7, 105.2, 101.9, 80.6, 70.2, 51.6, 48.1, 45.5, 35.5, 28.2;

IR (KBr): 2926, 1936, 1723, 1675, 1617, 1479, 1256, 1034, 718, 591 cm^{-1};

HRMS (ESI): $C_{18}H_{18}NaO_6$ $[M+Na]^+$ calcd: 353.0996, found: 353.0994.

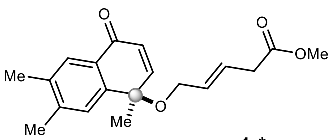

1r*

With 10 mol% catalyst at 40 °C for 10 h.

Purified by silica gel chromatography (ethyl acetate/petroleum ether 10:1); Colorless oil; 15.1 mg, 45% yield; 94.5:5.5 *er* determined by HPLC on a Chiralpak IC-H column (hexane/2-propanol = 40/60, flow rate = 1.0 mL/min, t_{minor} = 11.4 min, t_{major} = 9.5 min);

$[\alpha]^{rt}_{D}$ = -87.5 (*c* = 0.96, $CHCl_3$);

^{1}H NMR (300 MHz, $CDCl_3$) δ 7.88 (s, 1H), 7.41 (s, 1H), 6.90 (d, *J* = 10.3 Hz, 1H), 6.44 (d, *J* = 10.3 Hz, 1H), 5.69 (tdd, *J* = 21.2, 15.3, 6.3 Hz, 2H), 3.79 – 3.58 (m, 4H), 3.52 (dd, *J* = 11.7, 5.5 Hz, 1H), 3.07 (d, *J* = 6.7 Hz, 2H), 2.35 (d, *J* = 11.9 Hz, 6H), 1.60 (s, 3H);

^{13}C NMR (75 MHz, $CDCl_3$) δ 184.4, 171.8, 152.2, 143.2, 142.6, 137.1, 130.5, 130.2, 129.2, 127.4, 127.1, 125.1, 73.5, 65.3, 51.9, 37.6, 30.4, 20.4, 19.5;

IR (KBr): 2926, 1932, 1740, 1667, 1610, 1410, 1304, 1084, 836, 762 cm^{-1};

HRMS (ESI): $C_{19}H_{22}NaO_4$ $[M+Na]^+$ calcd: 337.1410, found: 337.1407.

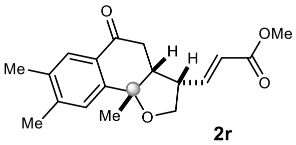

2r

Purified by silica gel chromatography (ethyl acetate/petroleum ether 4:1); White solid, m.p 88-90 °C; 14.8 mg, 44% yield; 98.5:1.5 *er* determined by HPLC on a Chiralpak IA-H column (hexane/2-propanol = 40/60, flow rate = 1.0 mL/min, t_{minor} = 6.7 min, t_{major} = 5.9 min);

$[\alpha]^{rt}_{D}$ = 151.5 (*c* = 0.99, $CHCl_3$);

^{1}H NMR (300 MHz, $CDCl_3$) δ 7.73 (s, 1H), 7.42 (s, 1H), 6.26 (dd, *J* = 15.5, 9.9 Hz, 1H), 5.76 (d, *J* = 15.5 Hz, 1H), 4.14 (dd, *J* = 8.9, 8.0 Hz, 1H), 3.66 (s, 3H), 3.41 (dd, *J* = 9.1, 6.4 Hz, 1H), 3.36 – 3.16 (m, 1H), 2.77 (d, *J* = 2.1 Hz, 3H), 2.32 (d, *J* = 15.5 Hz, 6H), 1.64 (s, 3H);

^{13}C NMR (75 MHz, $CDCl_3$) δ 195.3, 165.9, 146.3, 144.8, 144.1, 137.0, 129.5, 127.5, 126.9, 124.0, 80.4, 70.1, 51.6, 48.2, 45.6, 36.0, 28.4, 20.4, 19.4;

IR (KBr): 2926, 1916, 1724, 1683, 1610, 1256, 1044, 887, 621, 514 cm^{-1};

HRMS (ESI): $C_{19}H_{22}NaO_4$ $[M+Na]^+$ calcd: 337.1410, found: 337.1407.

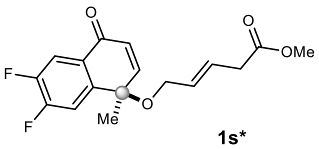

1s*

With 10 mol% catalyst at 40 °C for 12 h.

Purified by silica gel chromatography (ethyl acetate/petroleum ether 10:1); Colorless oil; 15.1 mg, 44% yield; 89:11 *er* determined by HPLC on a Chiralpak IC-H column (hexane/2-propanol = 40/60, flow rate = 1.0 mL/min, t_{minor} = 8.1 min, t_{major} = 7.1 min);

$[\alpha]^{rt}_D$ = -53.6 (*c* = 0.99, $CHCl_3$);

^{1}H NMR (300 MHz, $CDCl_3$) δ 8.01 – 7.67 (m, 1H), 7.48 (dd, *J* = 10.5, 7.4 Hz, 1H), 7.01 (d, *J* = 10.3 Hz, 1H), 6.49 (d, *J* = 10.3 Hz, 1H), 5.87 – 5.52 (m, 2H), 3.84 – 3.64 (m, 4H), 3.54 (dd, *J* = 11.6, 5.3 Hz, 1H), 3.09 (d, *J* = 6.7 Hz, 2H), 1.62 (s, 3H);

^{13}C NMR (75 MHz, $CDCl_3$) δ 181.8, 171.6, 155.7 (d, *J* = 12.8), 152.7, 152.2 (dd, *J* = 16.1, *J* = 13.1), 148.7 (d, *J* = 13.5), 143.0 (dd, *J* = 6.4, *J* = 3.4), 129.8, 129.7, 128.6, 125.4, 115.4 (dd, *J* = 27.0, *J* = 18.8), 73.3, 65.6, 51.8, 37.4, 30.4;

IR (KBr): 2954, 2640, 1740, 1674, 1505, 1333, 1087, 836, 652, 532 cm^{-1};

HRMS (ESI): $C_{17}H_{16}F_2NaO_4$ $[M+Na]^+$ calcd: 345.0909, found: 345.0906.

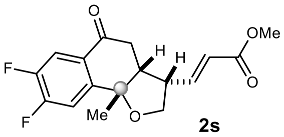

2s

Purified by silica gel chromatography (ethyl acetate/petroleum ether 4:1); White solid, m.p 81-83 °C; 14.1 mg, 41% yield; 94.5:5.5 *er* determined by HPLC on a Chiralpak IA-H column (hexane/2-propanol = 40/60, flow rate = 1.0 mL/min, t_{minor} = 5.5 min, t_{major} = 4.6 min);

$[\alpha]^{rt}_D$ = 77.4 (*c* = 1.03, $CHCl_3$);

^{1}H NMR (300 MHz, $CDCl_3$) δ 7.75 (t, *J* = 9.2 Hz, 1H), 7.59 – 7.38 (m, 1H), 6.18 (dd, *J* = 15.4, 9.8 Hz, 1H), 5.76 (d, *J* = 15.5 Hz, 1H), 4.18 (t, *J* = 8.5 Hz, 1H), 3.67 (s, 3H), 3.45 (dd, *J* = 9.0, 5.9 Hz, 1H), 3.31 (s, 1H), 2.83 (s, 3H), 1.64 (s, 3H);

^{13}C NMR (75 MHz, $CDCl_3$) δ192.7, 165.6, 156.6 (d, *J* = 13.5), 153.1 (d, *J* = 13.5), 152.0 (d, *J* = 13.5), 148.7 (d, *J* = 13.5), 145.4, 145.0 (dd, *J* = 6.0, *J* = 3.8), 124. 4, 115.4 (dd, *J* = 47.0, *J* = 18.0), 79.9, 70.4, 51.7, 48.1, 45.3, 35.7, 28.4;

IR (KBr): 2917, 2143, 1715, 1617, 1506, 1362, 1222, 1092, 786, 530 cm^{-1};

HRMS (ESI): $C_{17}H_{16}F_2NaO_4$ $[M+Na]^+$ calcd: 345.0909, found: 345.0907.

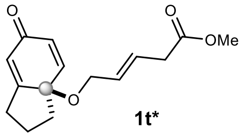

1t*

Purified by silica gel chromatography (ethyl acetate/petroleum ether 7:1); Colorless oil; 7.7 mg, 27% yield; 86:14 *er* determined by HPLC on a Chiralpak OD-H column (hexane/2-propanol = 40/60, flow rate = 1.0 mL/min, t_{minor} = 19.2 min, t_{major} = 17.4 min);

$[\alpha]^{rt}_D$ = 43.0 (*c* = 1.00, $CHCl_3$);

^{1}H NMR (300 MHz, $CDCl_3$) δ 6.81 (t, *J* = 9.4 Hz, 1H), 6.29 (dd, *J* = 10.0, 1.5 Hz, 1H), 6.18 (s, 1H), 5.86 – 5.50 (m, 2H), 3.79 – 3.55 (m, 5H), 3.08 (d, *J* = 6.8 Hz, 2H), 2.69 (dd, *J* = 21.1, 7.4 Hz, 1H), 2.52 – 2.38 (m, 1H), 2.30 – 2.05 (m, 2H), 1.99 – 1.84 (m, 1H);

^{13}C NMR (75 MHz, $CDCl_3$) δ 186.2, 171.8, 166.8, 145.4, 131.5, 130.3, 125.1, 125.0^6, 78.4, 64.2, 51.9, 37.5, 35.6, 28.7, 21.7;

IR (KBr): 2923, 1740, 1645, 1606, 1457, 1264, 1076, 895, 759, 636 cm^{-1};

HRMS (ESI): $C_{15}H_{18}NaO_4$ $[M+Na]^+$ calcd: 285.1097, found: 285.1106.

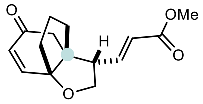

2t

Purified by silica gel chromatography (ethyl acetate/petroleum ether 4:1); Colorless oil; 6.8 mg, 24% yield; 93:7 *er* determined by HPLC on a Chiralpak IC-H column (hexane/2-propanol = 40/60, flow rate = 1.0 mL/min, t_{minor} = 8.9 min, t_{major} = 7.6 min);

$[\alpha]^{rt}_{D}$ = -103.0 (*c* = 1.00, $CHCl_3$);

^{1}H NMR (300 MHz, $CDCl_3$) δ 8.13 (d, *J* = 7.8 Hz, 1H), 7.63 (d, *J* = 3.3 Hz, 2H), 7.54 – 7.34 (m, 1H), 6.89 (d, *J* = 10.3 Hz, 1H), 6.57 (d, *J* = 10.3 Hz, 1H), 5.69 (tdd, *J* = 21.1, 15.3, 6.2 Hz, 2H), 3.86 – 3.64 (m, 4H), 3.57 (dd, *J* = 11.6, 5.3 Hz, 1H), 3.07 (d, *J* = 6.6 Hz, 2H), 2.01 (dd, *J* = 14.4, 7.1 Hz, 2H), 0.57 (t, *J* = 7.5 Hz, 3H);

^{13}C NMR (75 MHz, $CDCl_3$) δ 184.5, 171.8, 151.5, 143.9, 133.2, 132.4, 131.6, 130.5, 128.1, 126.5, 125.9, 125.0, 77.8, 65.3, 51.8, 37.5, 35.7, 8.0.

IR (KBr): 2953, 1932, 1724, 1686, 1604, 1436, 1265, 1076, 778, 632 cm^{-1};

HRMS (ESI): $C_{15}H_{18}NaO_4$ $[M+Na]^+$ calcd: 285.1097, found: 285.1108.

X-Ray Analysis of Product 2b

Supplementary Figure 11. X-ray analysis and data of product **2b**

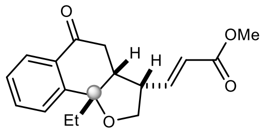

2b

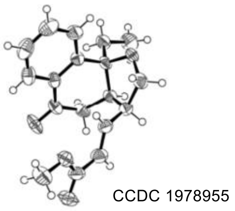

Bond precision: C-C = 0.0036 A Wavelength=1.54184

Cell: a=7.0777(2) b=9.8388(3) c=22.1253(6)

alpha=90 beta=90 gamma=90

Temperature: 292 K

	Calculated	Reported
Volume	1540.72(8)	1540.73(8)
Space group	P 21 21 21	P 21 21 21
Hall group	P 2ac 2ab	P 2ac 2ab
Moiety formula	C18 H20 O4	C18 H20 O4
Sum formula	C18 H20 O4	C18 H20 O4
Mr	300.34	300.34

Dx,g cm-3	1.295	1.295
Z	4	4
Mu (mm-1)	0.739	0.739
F000	640.0	640.0
F000'	642.02	
h,k,lmax	8,11,26	8,11,26
Nref	2728[1600]	2635
Tmin,Tmax	0.875,0.915	0.231,1.000
Tmin'	0.869	

Correction method= # Reported T Limits: Tmin=0.231 Tmax=1.000 AbsCorr = MULTI-SCAN

Data completeness= 1.65/0.97 Theta(max)= 66.589

R(reflections)= 0.0387(2468) wR2(reflections)= 0.1020(2635)

S = 1.073 Npar= 201

(X-Ray diffraction of Cu Kα radiation was used, the absolute configuration was unequivocally determined through anomalous dispersion effects with a Flack x parameter of 0.02(11). According to Howard. D. Flack, The use of X-ray Crystallography to Determine Absolute Configuration (II). Acta Chim. Slov. 55, 689–691 (2008).)

Gram Scale Trial of the Vinylogous KR Reaction and Transformation of the Resolution Products

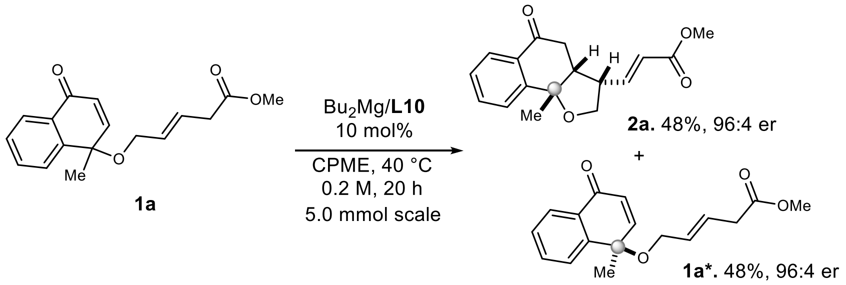

Supplementary Figure 12. Gram scale experiment of the KR reaction

To a stirred solution of **L10** (26.05 mg, 0.50 mmol) in CPME (10.0 mL) was added Bu_2Mg (500 µL, 1.0 M in heptane, 0.50 mmol) under an argon atmosphere, the mixture was then stirred at room temperature for 30 min to generate the catalyst. **1a** (5.0 mmol) in CPME (10.0 mL) was quickly added to the flask containing the in situ generated magnesium catalyst. After the addition, the reaction was stirred at 40 °C for 20 h. The reaction was quenched with saturated NH_4Cl and extracted with DCM. The organic layer was dried over Na_2SO_4 and concentrated under vacuum. Then the residue was purified by column chromatography (ethyl acetate/petroleum ether 10:1-4:1) to afford the product **1a*** (0.69 g, 48% yield) and **2a** (0.69 g, 48% yield).

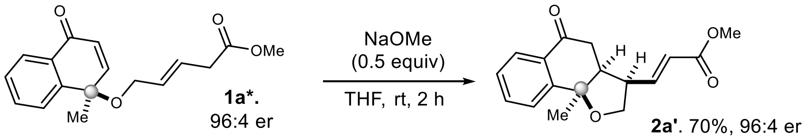

Supplementary Figure 13. Base promoted cyclization reaction of **1a***

To a stirred solution of **1a*** (0.1 mmol) in dried THF (1.0 mL, 0.1 M) was added NaOMe (0.05 mmol, 0.5 equiv) in one portion, the mixture was stirred for further 2 hours at room temperature. After completed, the mixture was quenched with H_2O, extracted with EA, dried with Na_2SO_4, then purified via flash column chromatography (ethyl acetate/petroleum ether 7:1-4:1) to give products **2a'** (21.6 mg, 70% yield).

White solid, m.p 65-67 ℃; 96.5:3.5 *er* determined by HPLC on a Chiralpak IA-H column (hexane/2-propanol = 40/60, flow rate = 1.0 mL/min, t_{minor} = 8.7 min, t_{major} = 9.5 min);

$[\alpha]^{rt}_D$ = -99.0 (*c* = 1.00, $CHCl_3$);

^{1}H NMR (300 MHz, $CDCl_3$) δ 7.96 (d, *J* = 7.9 Hz, 1H), 7.66 (q, *J* = 8.1 Hz, 2H), 7.41 (t, *J* = 7.3 Hz, 1H), 6.22 (dd, *J* = 15.5, 9.8 Hz, 1H), 5.75 (d, *J* = 15.6 Hz, 1H), 4.16 (t, *J* = 8.4 Hz, 1H), 3.65 (s, 3H), 3.50 – 3.37 (m, 1H), 3.33 (d, *J* = 7.0 Hz, 1H), 2.82 (s, 3H), 1.66 (s, 3H);

^{13}C NMR (75 MHz, $CDCl_3$) δ 195.2, 165.7, 146.7, 146.0, 134.7, 131.4, 128.1, 126.7, 126.2, 124.1, 80.4, 70.2, 51.6, 48.1, 45.5, 36.0, 28.5;

IR (KBr): 2952, 1847, 1724, 1687, 1600, 1276, 1171, 987, 774, 596 cm^{-1};

HRMS (ESI): $C_{17}H_{18}NaO_4$ $[M+Na]^+$ calcd: 309.1097, found: 309.1101.

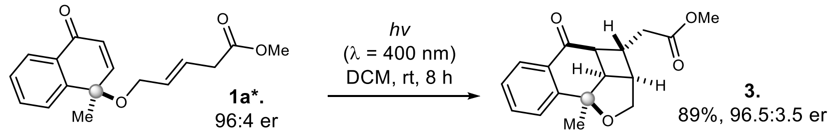

Supplementary Figure 14. Photocatalytic intramolecular [2+2] cyclization process of **1a***

The separated **1a*** (0.1 mmol) was dissolved in DCM (2.0 mL), then it was stirred at room temperature under 400 nm blue light for 8 hours. The reaction was quenched with saturated NH_4Cl solution and extracted with DCM. The organic layer was dried over Na_2SO_4 and concentrated under vacuum. Then the residue was purified by column chromatography (ethyl acetate/petroleum ether 4:1) to afford the product **3** (27.4 mg, 89% yield).

White solid, m.p 85-87 ℃; 96.5:3.5 *er* determined by HPLC on a Chiralpak IA-H column (hexane/2-propanol = 100/10, flow rate = 1.0 mL/min, t_{minor} = 30.4 min, t_{major} = 32.3 min);

$[\alpha]^{rt}_{D}$ = 63.0 (*c* = 1.00, $CHCl_3$);

^{1}H NMR (300 MHz, $CDCl_3$) δ 7.97 (d, *J* = 7.6 Hz, 1H), 7.82 – 7.56 (m, 2H), 7.46 (t, *J* = 7.4 Hz, 1H), 3.86 (s, 2H), 3.69 (s, 3H), 3.12 (t, *J* = 8.1 Hz, 1H), 2.97 (s, 1H), 2.93 – 2.76 (m, 2H), 2.73 – 2.53 (m, 2H), 1.61 (s, 3H);

^{13}C NMR (75 MHz, $CDCl_3$) δ 198.6, 172.0, 144.1, 133.6, 132.2, 128.5, 127.3, 126.6, 77.8, 70.6, 51.6, 44.6, 44.5, 42.9, 39.7, 39.3 23.0;

IR (KBr): 2953, 1978, 1734, 1677, 1601, 1374, 1275, 1037, 759, 643 cm^{-1};

HRMS (ESI): $C_{17}H_{18}NaO_4$ $[M+Na]^+$ calcd: 309.1097, found: 309.1102.

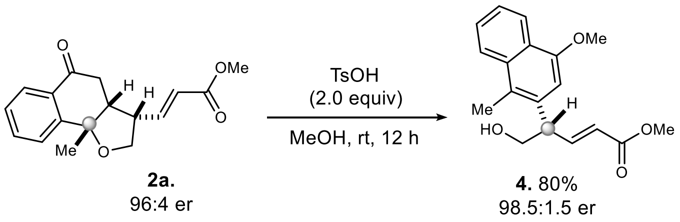

Supplementary Figure 15. Acid promoted the rearomatization reaction of **2a**

To a stirred solution of **2a** (0.1 mmol) in MeOH (1.0 mL, 0.1 M) was added TsOH (0.2 mmol, 2.0 equiv) in one portion, the mixture was stirred for further 12 hours at room temperature. After completed, the mixture was quenched with NH_4Cl aq, extracted with EA, dried with Na_2SO_4, then purified via flash column chromatography (ethyl acetate/petroleum ether 7:1) to give the product **4** (25.8 mg, 80% yield).

Colorless oil; 98.5:1.5 *er* determined by HPLC on a Chiralpak OD-H column (hexane/2-propanol = 80/20, flow rate = 1.0 mL/min, t_{minor} = 14.0 min, t_{major} = 15.2 min);

$[\alpha]^{rt}_{D}$ = -24.0 (*c* = 1.00, $CHCl_3$);

^{1}H NMR (300 MHz, $CDCl_3$) δ 8.26 (d, *J* = 8.2 Hz, 1H), 8.00 (d, *J* = 8.4 Hz, 1H), 7.50 (dt, *J* = 14.9, 7.1 Hz, 2H), 7.21 (dd, *J* = 15.7, 6.3 Hz, 1H), 6.61 (s, 1H), 5.88 (d, *J* = 15.8 Hz, 1H), 4.28 (dd, *J* = 13.5, 6.7 Hz, 1H), 4.12 – 3.81 (m, 5H), 3.69 (d, *J* = 9.9 Hz, 3H), 2.57 (s, 3H), 1.73 (s, 1H);

^{13}C NMR (75 MHz, $CDCl_3$) δ 166.9, 154.3, 148.2, 133.9, 133.3, 126.8, 125.1, 125.0, 124.3, 124.2, 122.6, 122.3, 102.6, 65.2, 55.5, 51.6, 47.1;

IR (KBr): 3423, 2950, 1721, 1650, 1596, 1459, 1240, 1116, 847, 761, 628 cm^{-1};

HRMS (ESI): $C_{18}H_{20}NaO_4$ $[M+Na]^+$ calcd: 323.1254, found: 323.1254.

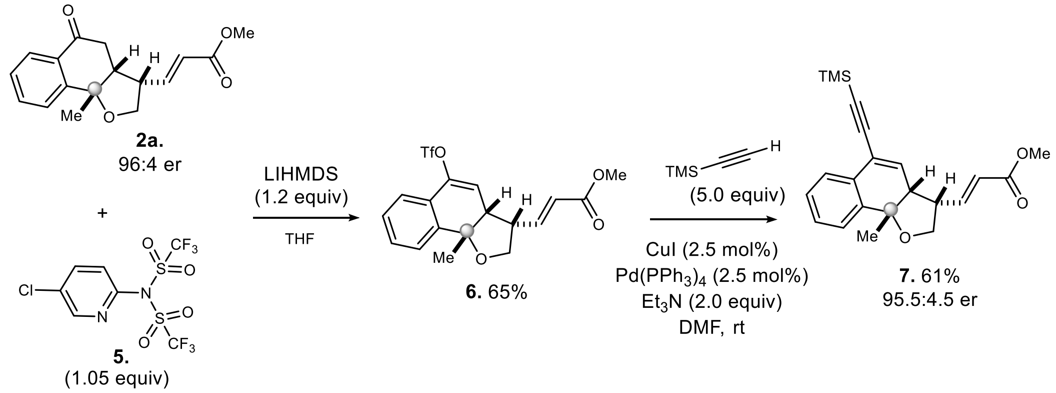

Supplementary Figure 16. Further transformations of **2a**

The separated product **2a** (1.0 equiv) was dissolved in THF, then LIHMDS (1.2 equiv) was added under an argon atmosphere at -40 °C. The mixture was stirred for further 0.5 hours, followed by cooling to -70 °C and bistrifluoromethyl-sulfonylaminochloropyridine (1.05 equiv) was added. The reaction was slowly warmed to room temperature and stirred for further 12 hours. After completed, the mixture was quenched with saturated NH_4Cl solution and extracted with EA. The organic layer was dried over Na_2SO_4 and concentrated under vacuum. Then the residue was purified by column chromatography (ethyl acetate/petroleum ether 10:1) to afford the product **6** (65% yield).

Compound **6** (0.1 mmol), $Pd(PPh_3)_4$ (2.5 mol%), Et_3N (2.0 equiv) and CuI (2.5 mol%) was stirred in DMF, then Trimethylsilylacetylene (5.0 equiv) was added under an argon atmosphere at rt. After completed, the mixture was quenched with H_2O, extracted with EA, dried with Na_2SO_4, then purified via flash column chromatography (ethyl acetate/petroleum ether 20:1) to give the product **7** (23.7 mg, 61% yield).

Colorless oil; 95.5:4.5 *er* determined by HPLC on a Chiralpak OD-H column (hexane/2-propanol = 100/10, flow rate = 1.0 mL/min, t_{minor} = 10.3 min, t_{major} = 9.5 min);

$[\alpha]^{rt}_D$ = 243.0 (*c* = 1.00, $CHCl_3$);

^{1}H NMR (300 MHz, $CDCl_3$) ^{1}H NMR (300 MHz, $CDCl_3$) δ 7.67 – 7.59 (m, 1H), 7.59 – 7.48 (m, 1H), 7.42 – 7.21 (m, 2H), 6.46 – 6.19 (m, 2H), 5.71 (d, *J* = 15.6 Hz, 1H), 4.11 (t, *J* = 6.6 Hz, 1H), 3.66 (s, 3H), 3.48 – 3.20 (m, 2H), 2.96 (dd, *J* = 9.4, 6.5 Hz, 1H), 1.47 (s, 3H), 0.29 (s, 9H);

^{13}C NMR (75 MHz, $CDCl_3$) δ 166.2, 146.5, 138.5, 131.6, 130.4, 129.2, 127.7, 125.5, 124.5, 122.5, 122.4, 102.1, 96.6, 81.9, 70.7, 51.4, 49.1, 47.8, 29.5, 0.0;

IR (KBr): 2959, 2151, 1727, 1656, 1601, 1436, 1251, 846, 762, 645 cm^{-1};

HRMS (ESI): $C_{22}H_{26}NaO_3Si$ $[M+Na]^+$ calcd: 389.1543, found: 389.1546.

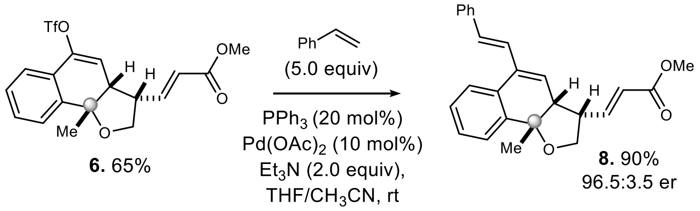

Supplementary Figure 17. Further transformation of **6** by a Heck reaction

Compound **6** (0.1 mmol), $Pd(OAc)_2$ (10 mol%), Et_3N (5.0 equiv) and PPh_3 (20 mol%) was stirred in THF/CH_3CN (3/1), then Styrene (5.0 equiv) was added under an argon atmosphere at rt. After completed, the mixture was quenched with H_2O, extracted with EA, dried with Na_2SO_4, then purified via flash column chromatography (ethyl acetate/petroleum ether 10:1) to give the product **8** (35.5 mg, 90% yield).

Colorless oil; 96.5:3.5 *er* determined by HPLC on a Chiralpak IC-H column (hexane/2-propanol = 100/10, flow rate = 1.0 mL/min, t_{minor} = 15.6 min, t_{major} = 14.6 min);

$[\alpha]^{rt}_{D}$ = 43.0 (*c* = 1.00, $CHCl_3$);

^{1}H NMR (300 MHz, $CDCl_3$) δ 7.65 – 7.58 (m, 1H), 7.47 (d, *J* = 7.3 Hz, 2H), 7.43 – 7.17 (m, 6H), 7.07 (d, *J* = 15.9 Hz, 1H), 6.84 (d, *J* = 15.9 Hz, 1H), 6.30 (dd, *J* = 15.5, 9.5 Hz, 1H), 6.04 (d, *J* = 6.4 Hz, 1H), 5.67 (d, *J* = 15.6 Hz, 1H), 4.13 (t, *J* = 6.9 Hz, 1H), 3.61 (s, 3H), 3.32 (dt, *J* = 15.4, 4.5 Hz, 2H), 3.07 – 2.85 (m, 1H), 1.50 (s, 3H);

^{13}C NMR (75 MHz, $CDCl_3$) δ 166.3, 147.7, 139.6, 137.3, 136.7, 132.5, 131.3, 128.8, 128.7, 127.7, 127.6, 126.6, 126.4, 125.0, 124.1, 122.0, 82.3, 70.6, 51.4, 48.9, 47.4, 29.0;

IR (KBr): 2968, 1959, 1723, 1654, 1600, 1436, 1271, 1040, 757, 700 cm^{-1};

HRMS (ESI): $C_{25}H_{24}NaO_3$ $[M+Na]^+$ calcd: 395.1618, found: 395.1618.

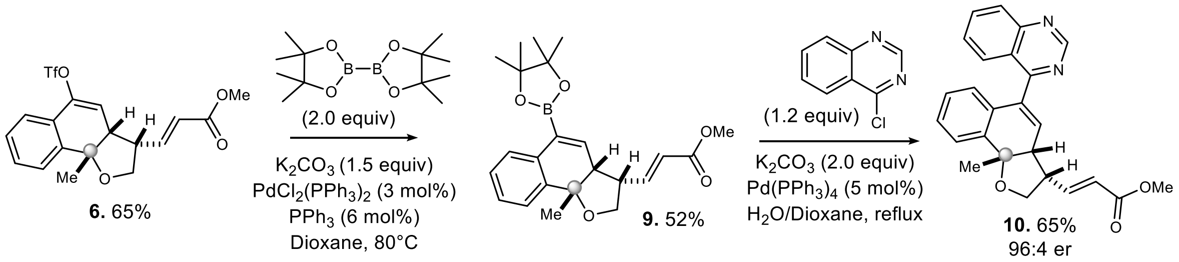

Supplementary Figure 18. Further transformations of **6** by cross-coupling reactions

Compound **6** (1.0 equiv), $PdCl_2(PPh_3)_2$ (3 mol%), K_2CO_3 (1.5 equiv) and PPh_3 (6 mol%) was stirred in 1,4-dioxane, then $(Bpin)_2$ (1.1 equiv) in 1,4-dioxane was added under an argon atmosphere at 80 °C. After completed, the mixture was quenched with H_2O, extracted with EA, dried with Na_2SO_4, then purified via flash column chromatography to give the product **9** (52% yield).

To a solution of **9** (0.1 mmol), $Pd(PPh_3)_4$ (5 mol%), K_2CO_3 (2.0 equiv) in 1,4-dioxane/H_2O (2/1) was added 4-chloroquinazoline (1.2 equiv) under an argon atmosphere. The reaction was stirred at reflux conditions, after completed analyzed by TLC, the mixture was quenched with H_2O, extracted with EA, dried with Na_2SO_4, then purified via flash column chromatography (ethyl acetate/petroleum ether 4:1) to give the product **10** (27.3 mg, 65% yield).

Colorless oil; 96:4 *er* determined by HPLC on a Chiralpak OD-H column (hexane/2-propanol = 100/10, flow rate = 1.0 mL/min, t_{minor} = 29.8 min, t_{major} = 25.5 min);

$[\alpha]^{rt}_D = 242.0$ (c = 1.00, $CHCl_3$);

^{1}H NMR (300 MHz, $CDCl_3$) δ 9.38 (s, 1H), 8.09 (t, J = 12.0 Hz, 2H), 7.96 – 7.85 (m, 1H), 7.71 (d, J = 7.2 Hz, 1H), 7.53 (s, 1H), 7.35 (t, J = 7.0 Hz, 1H), 7.11 (t, J = 7.0 Hz, 1H), 6.86 – 6.47 (m, 2H), 6.06 (s, 1H), 5.86 (d, J = 15.5 Hz, 1H), 4.38 – 4.14 (m, 1H), 3.69 (s, 3H), 3.60 – 3.39 (m, 2H), 3.23 – 3.07 (m, 1H), 1.65 (s, 3H);

^{13}C NMR (75 MHz, $CDCl_3$) δ 166.2, 154.8, 150.8, 139.3, 134.2, 131.6, 129.4, 128.8, 128.0, 127.8, 127.1, 125.6, 125.1, 124.4, 122.8, 82.3, 70.6, 51.6, 49.1, 47.7, 29.8;

IR (KBr): 2962, 1925, 1723, 1612, 1566, 1491, 1275, 1074, 770, 587 cm^{-1};

HRMS (ESI): $C_{25}H_{22}N_2NaO_3$ $[M+Na]^+$ calcd: 421.1523, found: 421.1532.

General Synthesis Methods of the Initial Materials

General Procedure A:

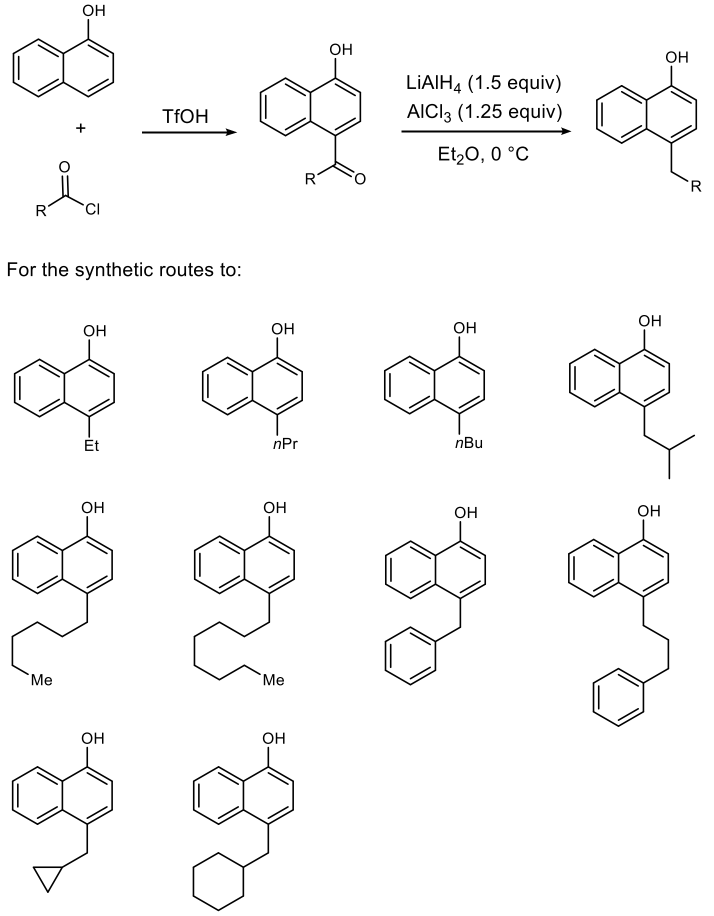

Supplementary Figure 19. Synthetic method of different 1-naphthols

Methods: To a solution of 1-naphthol (1.0 equiv) in TfOH (10 mL) at 0°C was added chloride (1.0 equiv). The mixture was stirred at 0 °C for 0.5-1.0 hours. After completed, the mixture was quenched with H_2O, extracted with CH_2Cl_2, then the separated organic phase was washed with saturated $NaHCO_3$ solution, dried with Na_2SO_4, and purified via flash column chromatography to give pure products.

General Procedure B:

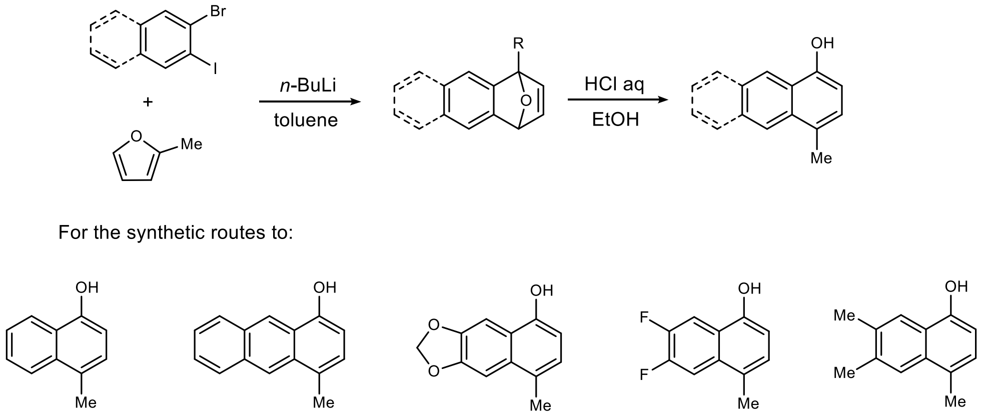

Supplementary Figure 20. Alternative synthetic method of different phenols

Methods: To a solution of halide (1.0 equiv) and 2-methylfuran (2.0 equiv) in dried toluene at -20 °C was added ^{n}BuLi (1.5 equiv, 2.5 M in hexane) under an argon atmosphere. The mixture was stirred at -20°C for 2 hours. After completed, the reaction was quenched with saturated NH_4Cl and extracted with EA. The organic layer was dried over Na_2SO_4 and concentrated under vacuum. Then the residue was purified by column chromatography to afford the products.

General Procedure C:

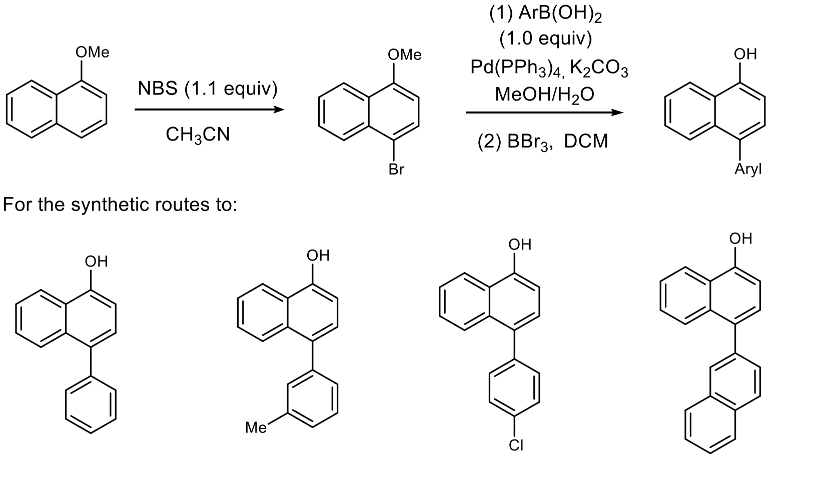

Supplementary Figure 21. Synthetic method of aryl-substituted 1-naphthols

Method: To a solution of 1-Methoxynaphthalene (1.0 equiv) in CH_3CN at room temperature was added NBS (1.1 equiv). The mixture was stirred at room temperature overnight. After completed, the mixture was concentrated under vacuum. Then the residue was purified by column chromatography to afford the products.

The compound obtained above (1.0 equiv) in $MeOH/H_2O$ (10/1) was successively added $Pd(PPh_3)_4$ (5 mol%), K_2CO_3 (2.0 equiv), and $ArB(OH)_2$ (1.0 equiv) at room temperature, followed by refluxing for 24 hours under an argon atmosphere. The

solution was cooled to room temperature and extracted with CH_2Cl_2, washed with water and brine, dried over $MgSO_4$, and concentrated to give a crude product. Then, it was dissolved in DCM, and BBr_3 (1.2 equiv) was added at room temperature, after the reaction was completed, H_2O was added cautiously at 0 °C, extracted with CH_2Cl_2, washed with water and brine, dried over $MgSO_4$, then purified by column chromatography to afford the pure products.

General Procedure D:

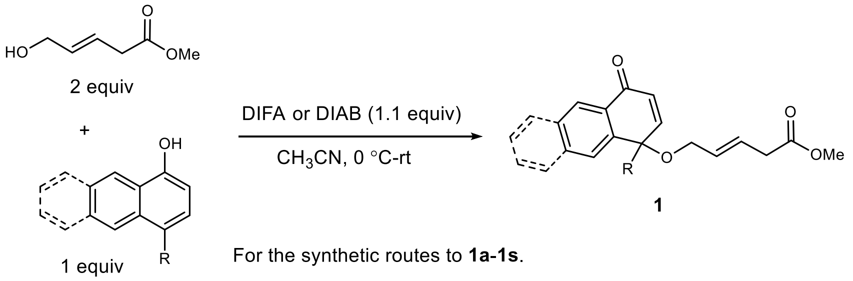

Supplementary Figure 22. General oxidative method of 1-naphthols

Methods: To a solution of substituted-naphthol (1.0 equiv) in CH_3CN at 0°C was added DIFA or DIAB (1.1 equiv). The mixture was stirred for further 6-8 hours. After completed, the mixture was quenched with H_2O, extracted with EA, and the separated organic phase was washed with saturated $NaHCO_3$ solution, dried with Na_2SO_4, then purified via flash column chromatography to give products **1**.

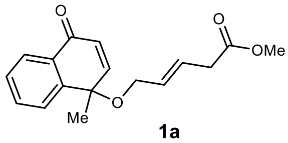

1a

^{1}H NMR (300 MHz, $CDCl_3$) δ 8.13 (d, *J* = 7.6 Hz, 1H), 7.65 (q, *J* = 7.7 Hz, 2H), 7.56 – 7.37 (m, 1H), 6.97 (d, *J* = 10.3 Hz, 1H), 6.49 (d, *J* = 10.3 Hz, 1H), 5.89 – 5.45 (m, 2H), 3.78 – 3.58 (m, 4H), 3.51 (dd, *J* = 11.7, 5.6 Hz, 1H), 3.07 (d, *J* = 6.7 Hz, 2H), 1.63 (s, 3H); **^{13}C NMR** (75 MHz, $CDCl_3$) δ 184.2, 171.8, 152.6, 145.0, 133.3, 131.2, 130.3, 130.1, 128.2, 126.8, 126.2, 125.3, 73.7, 65.5, 51.9, 37.5, 30.5; **HRMS** (ESI): $C_{17}H_{18}NaO_4$ $[M+Na]^+$ calcd: 309.1097, found: 309.1098.

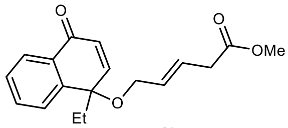

1b

^{1}H NMR (300 MHz, $CDCl_3$) δ 8.13 (d, *J* = 7.9 Hz, 1H), 7.69 – 7.56 (m, 2H), 7.46 (ddd, *J* = 8.2, 5.1, 3.4 Hz, 1H), 6.89 (d, *J* = 10.3 Hz, 1H), 6.58 (d, *J* = 10.4 Hz, 1H), 5.87 – 5.47 (m, 2H), 3.84 – 3.62 (m, 3H), 3.57 (dd, *J* = 11.8, 5.1 Hz, 1H), 3.07 (d, *J* = 6.6 Hz, 2H), 2.01 (dt, *J* = 7.5, 6.2 Hz, 2H), 0.56 (t, *J* = 7.5 Hz, 3H); **^{13}C NMR** (75 MHz, $CDCl_3$) δ 184.5, 171.8, 151.5, 143.9, 133.3, 132.4, 131.6, 130.5, 128.1, 126.5, 126.0, 125.0, 77.8, 65.3, 51.9, 37.5, 35.7, 8.0; **HRMS** (ESI): $C_{18}H_{20}NaO_4$ $[M+Na]^+$ calcd: 323.1254, found: 323.1253.

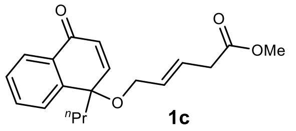

1c

^{1}H NMR (300 MHz, $CDCl_3$) δ 8.12 (d, *J* = 7.8 Hz, 1H), 7.63 (d, *J* = 3.7 Hz, 2H), 7.52 – 7.40 (m, 1H), 6.92 (d, *J* = 10.4 Hz, 1H), 6.55 (d, *J* = 10.3 Hz, 1H), 5.85 – 5.49 (m, 2H), 3.81 – 3.64 (m, 4H), 3.55 (dd, *J* = 11.9, 5.4 Hz, 1H), 3.07 (d, *J* = 6.6 Hz, 2H), 2.01 – 1.86 (m, 2H), 1.00 (dd, *J* = 17.6, 7.6 Hz, 1H), 0.86 – 0.67 (m, 4H); **^{13}C NMR** (75 MHz, $CDCl_3$) δ 184.5, 171.9, 151.9, 144.3, 133.2, 132.1, 131.3, 130.5, 128.1, 126.6, 126.0, 125.0, 77.2, 65.2, 51.9, 45.2, 37.5, 16.9, 14.0; **HRMS** (ESI): $C_{19}H_{22}NaO_4$ $[M+Na]^+$ calcd: 337.1410, found: 337.1408.

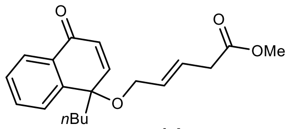

1d

^{1}H NMR (300 MHz, $CDCl_3$) δ 8.12 (d, *J* = 7.8 Hz, 1H), 7.63 (d, *J* = 3.7 Hz, 2H), 7.54 – 7.37 (m, 1H), 6.91 (d, *J* = 10.4 Hz, 1H), 6.55 (d, *J* = 10.3 Hz, 1H), 5.84 – 5.46 (m, 2H), 3.84 – 3.63 (m, 4H), 3.55 (dd, *J* = 11.4, 5.0 Hz, 1H), 3.07 (d, *J* = 6.1 Hz, 2H), 1.96 (dd, *J* = 16.0, 8.5 Hz, 2H), 1.26 – 1.04 (m, 3H), 0.70 (dt, *J* = 16.0, 7.4 Hz, 4H); **^{13}C NMR** (75 MHz, $CDCl_3$) δ 184.5, 171.8, 151.9, 144.2, 133.3, 132.2, 131.3, 130.5, 128.1, 126.6, 126.0, 125.0, 77.2, 65.2, 51.9, 42.7, 37.5, 25.6, 22.6, 13.8; **HRMS** (ESI): $C_{20}H_{24}NaO_4$ $[M+Na]^+$ calcd: 351.1567, found: 351.1568.

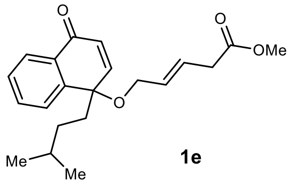

1e

^{1}H NMR (300 MHz, $CDCl_3$) δ 8.13 (d, *J* = 7.8 Hz, 1H), 7.64 (d, *J* = 3.7 Hz, 2H), 7.53 – 7.38 (m, 1H), 6.89 (d, *J* = 10.3 Hz, 1H), 6.56 (d, *J* = 10.3 Hz, 1H), 5.96 – 5.53 (m, 2H), 3.83 – 3.62 (m, 4H), 3.54 (dd, *J* = 11.5, 5.3 Hz, 1H), 3.07 (d, *J* = 6.6 Hz, 2H), 2.01 (ddd, *J* = 18.0, 12.5, 5.5 Hz, 2H), 1.47 – 1.29 (m, 2H), 1.00 (ddd, *J* = 18.0, 12.0, 5.7 Hz, 2H), 0.74 (dd, *J* = 13.8, 6.6 Hz, 6H), 0.60 – 0.41 (m, 1H); **^{13}C NMR** (75 MHz, $CDCl_3$) δ 184.5, 171.8, 151.8, 144.1, 133.3, 132.2, 131.4, 130.4, 128.1, 126.5, 125.9, 125.1, 77.2, 65.2, 51.8, 40.8, 37.5, 32.2, 28.0, 22.4, 22.2; **HRMS** (ESI): $C_{21}H_{26}NaO_4$ $[M+Na]^+$ calcd: 365.1723, found: 365.1723.

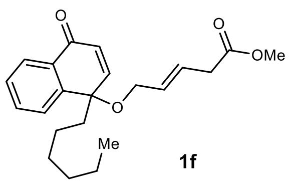

1f

^{1}H NMR (300 MHz, $CDCl_3$) δ 8.12 (d, *J* = 7.8 Hz, 1H), 7.63 (d, *J* = 3.8 Hz, 2H), 7.45 (dt, *J* = 8.2, 4.2 Hz, 1H), 6.91 (d, *J* = 10.3 Hz, 1H), 6.55 (d, *J* = 10.3 Hz, 1H), 5.92 – 5.54 (m, 2H), 3.81 – 3.64 (m, 4H), 3.55 (dd, *J* = 11.9, 4.9 Hz, 1H), 3.07 (d, *J* = 6.7 Hz, 2H), 2.01 – 1.89 (m, 2H), 1.14 (m, *J* = 14.3 Hz, 7H), 0.79 (t, *J* = 6.6 Hz, 3H), 0.73 – 0.61 (m, 1H); **^{13}C NMR** (75 MHz, $CDCl_3$) δ 184.5, 171.8, 151.9, 144.2, 133.2, 132.2, 131.3, 130.4, 128.1, 126.5, 126.0, 125.0, 77.2, 65.1, 51.8, 42.9, 37.5, 31.4, 29.1, 23.4, 22.4, 14.0; **HRMS** (ESI): $C_{22}H_{28}NaO_4$ $[M+Na]^+$ calcd: 379.1880, found: 379.1881.

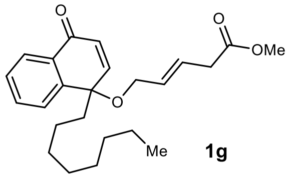

1g

^{1}H NMR (300 MHz, $CDCl_3$) δ 8.12 (d, *J* = 7.8 Hz, 1H), 7.62 (t, *J* = 6.1 Hz, 2H), 7.51 – 7.32 (m, 1H), 6.91 (d, *J* = 10.4 Hz, 1H), 6.55 (d, *J* = 10.3 Hz, 1H), 5.93 – 5.42 (m, 2H), 3.78 – 3.61 (m, 4H), 3.55 (dd, *J* = 11.8, 5.4 Hz, 1H), 3.07 (d, *J* = 6.7 Hz, 2H), 1.94 (t, *J* = 8.6 Hz, 2H), 1.33 – 1.05 (m, 12H), 0.83 (t, *J* = 6.8 Hz, 3H); **^{13}C NMR** (75 MHz, $CDCl_3$) δ 184.5, 171.8, 151.9, 144.2, 133.2, 132.2, 131.3, 130.5, 128.1, 126.6, 126.0, 125.0, 77.2, 65.2, 51.9, 42.9, 37.5, 31.7, 29.5, 29.2, 29.1, 23.4, 22.6, 14.1; **HRMS** (ESI): $C_{24}H_{32}NaO_4$ $[M+Na]^+$ calcd: 407.2193, found: 407.2191.

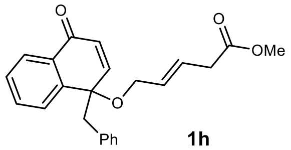

1h

^{1}H NMR (300 MHz, $CDCl_3$) δ 7.98 (d, *J* = 7.8 Hz, 1H), 7.68 (dt, *J* = 13.8, 7.8 Hz, 2H), 7.44 (dd, *J* = 10.8, 4.2 Hz, 1H), 7.17 – 6.99 (m, 3H), 6.88 (d, *J* = 10.4 Hz, 1H), 6.75 (d, *J* = 6.2 Hz, 2H), 6.39 (d, *J* = 10.4 Hz, 1H), 5.92 – 5.50 (m, 2H), 3.83 – 3.53 (m, 5H), 3.22 (dd, *J* = 35.6, 13.0 Hz, 2H), 3.09 (d, *J* = 6.7 Hz, 2H); **^{13}C NMR** (75 MHz, $CDCl_3$) δ 183.8, 171.9, 151.1, 143.7, 134.6, 133.0, 132.2, 131.3, 130.6, 130.4, 128.3, 127.7, 126.9, 126.5, 126.4, 124.8, 77.7, 65.4, 51.9, 50.0, 37.6; **HRMS** (ESI): $C_{23}H_{22}NaO_4$ $[M+Na]^+$ calcd: 385.1410, found: 385.1407.

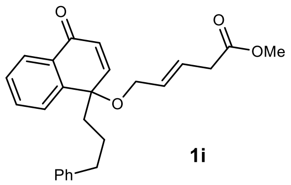

1i

^{1}H NMR (300 MHz, $CDCl_3$) δ 8.11 (d, *J* = 7.7 Hz, 1H), 7.64 – 7.53 (m, 2H), 7.48 – 7.38 (m, 1H), 7.24 – 7.10 (m, 3H), 6.99 (d, *J* = 7.0 Hz, 2H), 6.87 (d, *J* = 10.3 Hz, 1H), 6.53 (d, *J* = 10.3 Hz, 1H), 5.81 – 5.51 (m, 2H), 3.76 – 3.62 (m, 4H), 3.52 (dd, *J* = 11.7, 5.3 Hz, 1H), 3.05 (d, *J* = 6.7 Hz, 2H), 2.45 (td, *J* = 7.4, 2.6 Hz, 2H), 2.05 – 1.95 (m, 2H), 1.57 – 1.40 (m, 1H), 1.11 – 0.97 (m, 1H); **^{13}C NMR** (75 MHz, $CDCl_3$) δ 184.3, 171.7, 151.6, 144.0, 141.4, 133.3, 132.2, 131.5, 130.4, 128.3, 128.2, 126.6, 126.0, 125.9, 125.1, 77.0, 65.2, 51.9, 42.3, 37.5, 35.6, 25.2; **HRMS** (ESI): $C_{25}H_{26}NaO_4$ $[M+Na]^+$ calcd: 413.1723, found: 413.1724.

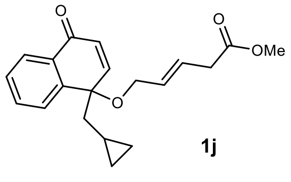

1j

^{1}H NMR (300 MHz, $CDCl_3$) δ 8.20 (d, *J* = 7.7 Hz, 1H), 7.77 – 7.62 (m, 2H), 7.52 (ddd, *J* = 8.2, 5.4, 3.1 Hz, 1H), 7.11 (d, *J* = 10.3 Hz, 1H), 6.64 (d, *J* = 10.3 Hz, 1H), 6.03 – 5.57 (m, 2H), 3.91 – 3.50 (m, 5H), 3.14 (d, *J* = 6.6 Hz, 2H), 2.14 (dd, *J* = 13.5, 5.7 Hz, 1H), 1.81 (dd, *J* = 13.5, 7.3 Hz, 1H), 0.53 – 0.30 (m, 2H), 0.20 (dt, *J* = 13.8, 7.3 Hz, 1H), 0.01 (dt, *J* = 9.0, 4.5 Hz, 1H), -0.33 (td, *J* = 9.7, 4.9 Hz, 1H); **^{13}C NMR** (75 MHz, $CDCl_3$) δ 184.5, 171.8, 152.2, 144.6, 133.0, 132.3, 131.1, 130.5, 128.1, 126.5, 126.1, 124.9, 77.6, 65.1, 51.8, 48.4, 37.5, 5.8, 4.7, 4.5; **HRMS** (ESI): $C_{20}H_{22}NaO_4$ $[M+Na]^+$ calcd: 349.1410, found: 349.1412.

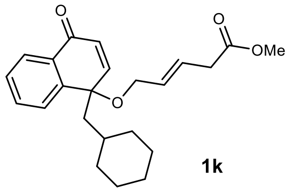

1k

^{1}H NMR (300 MHz, $CDCl_3$) δ 8.12 (d, *J* = 7.9 Hz, 1H), 7.71 – 7.54 (m, 2H), 7.54 – 7.39 (m, 1H), 6.94 (d, *J* = 10.3 Hz, 1H), 6.56 (d, *J* = 10.3 Hz, 1H), 5.82 – 5.49 (m, 2H), 3.79 – 3.57 (m, 4H), 3.49 (dd, *J* = 11.9, 5.3 Hz, 1H), 3.06 (d, *J* = 6.7 Hz, 2H), 1.93 (d, *J* = 4.7 Hz, 2H), 1.61 (dd, *J* = 27.2, 11.6 Hz, 2H), 1.43 (dd, *J* = 18.1, 10.2 Hz, 2H), 1.13 – 0.86 (m, 6H); **^{13}C NMR** (75 MHz, $CDCl_3$) δ 184.4, 171.8, 151.9, 144.3, 133.0, 132.0, 131.1, 130.5, 128.1, 126.5^{3}, 126.5, 124.8, 76.7, 64.7, 51.8, 50.4, 37.5, 35.0, 34.3, 33.1, 26.0, 25.9; **HRMS** (ESI): $C_{23}H_{28}NaO_4$ $[M+Na]^+$ calcd: 391.1880, found: 391.1889.

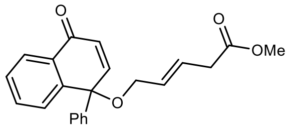

1l

^{1}H NMR (300 MHz, $CDCl_3$) δ 8.18 (dd, *J* = 13.5, 5.7 Hz, 1H), 7.52 (dd, *J* = 8.6, 5.1 Hz, 1H), 7.45 (d, *J* = 7.4 Hz, 1H), 7.38 (dd, *J* = 14.1, 6.6 Hz, 3H), 7.26 (dt, *J* = 8.8, 6.7 Hz, 3H), 6.89 (d, *J* = 10.2 Hz, 1H), 6.51 (d, *J* = 10.2 Hz, 1H), 5.93 – 5.58 (m, 2H), 3.96 (dd, *J* = 12.2, 4.6 Hz, 1H), 3.81 – 3.63 (m, 4H), 3.10 (d, *J* = 6.5 Hz, 2H); **^{13}C NMR** (75 MHz, $CDCl_3$) δ 184.7, 171.9, 151.3, 144.4, 141.9, 133.5, 131.8, 130.4, 129.2, 128.9, 128.5, 128.4, 128.3, 128.2, 127.7, 126.4, 126.1, 124.6, 64.8,51.9, 37.6; **HRMS** (ESI): $C_{22}H_{20}NaO_4$ $[M+Na]^+$ calcd: 371.1254, found: 371.1257.

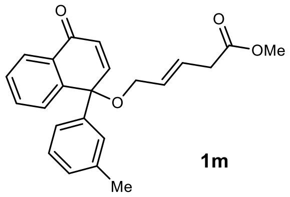

1m

^{1}H NMR (300 MHz, $CDCl_3$) δ 8.16 (dd, *J* = 7.7, 0.8 Hz, 1H), 7.52 (td, *J* = 7.5, 1.3 Hz, 1H), 7.43 (dd, *J* = 11.9, 4.4 Hz, 2H), 7.17 (t, *J* = 6.9 Hz, 3H), 7.03 (d, *J* = 5.4 Hz, 1H), 6.88 (d, *J* = 10.2 Hz, 1H), 6.50 (d, *J* = 10.2 Hz, 1H), 5.90 – 5.58 (m, 2H), 3.95 (dd, *J* = 12.0, 4.7 Hz, 1H), 3.84 – 3.60 (m, 4H), 3.10 (d, *J* = 6.5 Hz, 2H), 2.28 (s, 3H); **^{13}C NMR** (75 MHz, $CDCl_3$) δ 184.8, 171.9, 151.4, 144.5, 141.8, 138.2, 133.5, 131.7, 130.5, 129.1, 128.5, 128.4, 128.3^{7}, 128.3, 126.6, 126.4, 124.6, 123.2, 77.5, 64.8, 51.9, 37.6, 21.6; **HRMS** (ESI): $C_{23}H_{22}NaO_4$ $[M+Na]^+$ calcd: 385.1410, found: 385.1410.

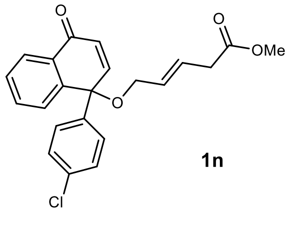

1n

^{1}H NMR (300 MHz, $CDCl_3$) δ 8.17 (d, *J* = 7.7 Hz, 1H), 7.58 – 7.50 (m, 1H), 7.47 – 7.41 (m, 1H), 7.37 – 7.17 (m, 5H), 6.84 (d, *J* = 10.2 Hz, 1H), 6.52 (d, *J* = 10.2 Hz, 1H), 5.97 – 5.59 (m, 2H), 3.94 (dd, *J* = 11.8, 5.3 Hz, 1H), 3.82 – 3.51 (m, 4H), 3.10 (d, *J* = 6.6

Hz, 2H); **^{13}C NMR** (75 MHz, $CDCl_3$) δ 184.4, 171.9, 150.7, 143.9, 140.5, 133.6, 131.7, 130.2, 129.5, 128.7, 128.6, 128.3, 127.5, 126.5, 124.8, 64.8, 51.9, 37.5, 29.7; **HRMS** (ESI): $C_{22}H_{19}ClNaO_4$ $[M+Na]^+$ calcd: 405.0864, found: 405.0868.

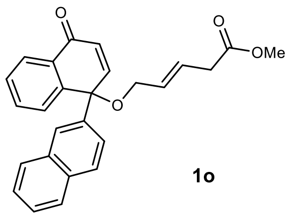

1o

^{1}H NMR (300 MHz, $CDCl_3$) δ 8.29 – 8.15 (m, 1H), 8.04 (s, 1H), 7.77 (ddd, *J* = 23.0, 14.5, 6.1 Hz, 3H), 7.57 – 7.36 (m, 5H), 7.28 – 7.13 (m, 1H), 6.94 (d, *J* = 10.2 Hz, 1H), 6.57 (d, *J* = 10.2 Hz, 1H), 5.98 – 5.66 (m, 2H), 4.02 (dd, *J* = 12.0, 5.3 Hz, 1H), 3.87 – 3.57 (m, 4H), 3.13 (d, *J* = 6.7 Hz, 2H); **^{13}C NMR** (75 MHz, $CDCl_3$) δ 184.7, 171.9, 151.1, 144.4, 139.1, 133.5, 133.2, 132.7, 131.9, 130.5, 129.5, 128.5, 128.4[5], 128.3, 128.2, 127.6, 126.5, 126.3, 124.9, 124.8, 77.6, 65.0, 51.9, 37.6; **HRMS** (ESI): $C_{26}H_{22}NaO_4$ $[M+Na]^+$ calcd: 421.1410, found: 421.1412.

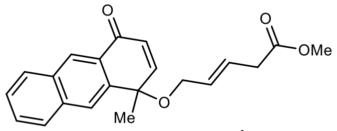

1p

^{1}H NMR (300 MHz, $CDCl_3$) δ 8.72 (s, 1H), 8.13 (s, 1H), 8.02 (d, *J* = 8.0 Hz, 1H), 7.92 (d, *J* = 8.2 Hz, 1H), 7.59 (dt, *J* = 21.1, 7.3 Hz, 2H), 7.26 (s, 1H), 7.03 (d, *J* = 10.4 Hz, 1H), 6.56 (d, *J* = 10.4 Hz, 1H), 6.06 – 5.39 (m, 2H), 3.75 (dd, *J* = 11.7, 5.7 Hz, 1H), 3.70 – 3.49 (m, 4H), 3.06 (d, *J* = 6.6 Hz, 2H), 1.72 (s, 3H); **^{13}C NMR** (75 MHz, $CDCl_3$) δ 184.6, 171.8, 152.9, 139.9, 135.4, 132.3, 130.5, 130.4, 129.8, 128.8, 128.6, 128.0, 127.0, 125.8, 125.1, 74.0, 65.4, 51.8, 37.5, 31.6; **HRMS** (ESI): $C_{21}H_{20}NaO_4$ $[M+Na]^+$ calcd: 359.1254, found: 359.1256.

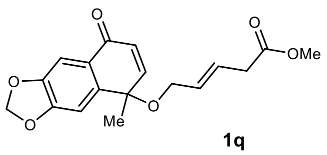

1q

^{1}H NMR (300 MHz, $CDCl_3$) δ 7.52 (s, 1H), 7.06 (s, 1H), 6.89 (d, *J* = 10.2 Hz, 1H), 6.42 (d, *J* = 10.2 Hz, 1H), 6.07 (d, *J* = 4.1 Hz, 2H), 5.86 – 5.48 (m, 2H), 3.75 – 3.65 (m, 4H), 3.54 (dd, *J* = 11.7, 5.5 Hz, 1H), 3.08 (d, *J* = 6.7 Hz, 2H), 1.58 (s, 3H); **^{13}C NMR** (75 MHz, $CDCl_3$) δ 182.9, 171.8, 152.3, 151.8, 148.1, 142.1, 130.2, 129.8, 126.6, 125.2, 105.6, 105.5[6], 101.9, 73.9, 65.4, 51.9, 37.5, 30.4; **HRMS** (ESI): $C_{18}H_{18}NaO_6$ $[M+Na]^+$ calcd: 353.0996, found: 353.0992.

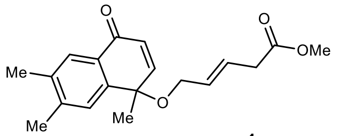

1r

^{1}H NMR (300 MHz, $CDCl_3$) δ 7.88 (s, 1H), 7.41 (s, 1H), 6.90 (d, *J* = 10.3 Hz, 1H), 6.44 (d, *J* = 10.3 Hz, 1H), 5.81 – 5.46 (m, 2H), 3.78 – 3.58 (m, 4H), 3.52 (dd, *J* = 11.7, 5.4 Hz, 1H), 3.07 (d, *J* = 6.6 Hz, 2H), 2.35 (d, *J* = 12.2 Hz, 6H), 1.60 (s, 3H); **^{13}C NMR** (75

MHz, $CDCl_3$) δ 184.4, 171.8, 152.2, 143.2, 142.6, 137.1, 130.5, 130.2, 129.2, 127.4, 127.1, 125.1, 73.5, 65.3, 51.9, 37.6, 30.4, 20.4, 19.5; **HRMS** (ESI): $C_{19}H_{22}NaO_4$ $[M+Na]^+$ calcd: 337.1410, found: 337.1407.

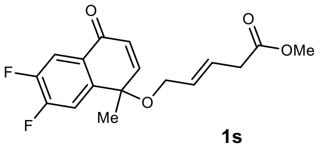

1s

^{1}H NMR (300 MHz, $CDCl_3$) δ 8.01 – 7.67 (m, 1H), 7.48 (dd, *J* = 10.5, 7.4 Hz, 1H), 7.01 (d, *J* = 10.3 Hz, 1H), 6.49 (d, *J* = 10.3 Hz, 1H), 5.87 – 5.52 (m, 2H), 3.84 – 3.64 (m, 4H), 3.54 (dd, *J* = 11.6, 5.3 Hz, 1H), 3.09 (d, *J* = 6.7 Hz, 2H), 1.62 (s, 3H); **^{13}C NMR** (75 MHz, $CDCl_3$) δ192.7, 165.6, 156.6 (d, *J* = 13.5), 153.1 (d, *J* = 13.5), 152.0 (d, *J* = 13.5), 148.7 (d, *J* = 13.5), 145.4, 145.0 (dd, *J* = 6.0, *J* = 3.8), 124.4, 115.4 (dd, *J* = 47.0, *J* = 18.0), 79.9, 70.4, 51.7, 48.1, 45.3, 35.7, 28.4; **HRMS** (ESI): $C_{17}H_{16}F_2NaO_4$ $[M+Na]^+$ calcd: 345.0909, found: 345.0906.

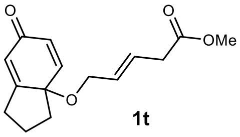

1t

^{1}H NMR (300 MHz, $CDCl_3$) δ 6.81 (t, *J* = 9.4 Hz, 1H), 6.29 (dd, *J* = 10.0, 1.5 Hz, 1H), 6.18 (s, 1H), 5.86 – 5.50 (m, 2H), 3.79 – 3.55 (m, 5H), 3.08 (d, *J* = 6.8 Hz, 2H), 2.69 (dd, *J* = 21.1, 7.4 Hz, 1H), 2.52 – 2.38 (m, 1H), 2.30 – 2.05 (m, 2H), 1.99 – 1.84 (m, 1H); **^{13}C NMR** (75 MHz, $CDCl_3$) δ 186.2, 171.8, 166.8, 145.4, 131.5, 130.3, 125.1, 125.0[6], 78.4, 64.2, 51.9, 37.5, 35.6, 28.7, 21.7; **HRMS** (ESI): $C_{15}H_{18}NaO_4$ $[M+Na]^+$ calcd: 285.1097, found: 285.1106.

Synthesis Procedure of L10

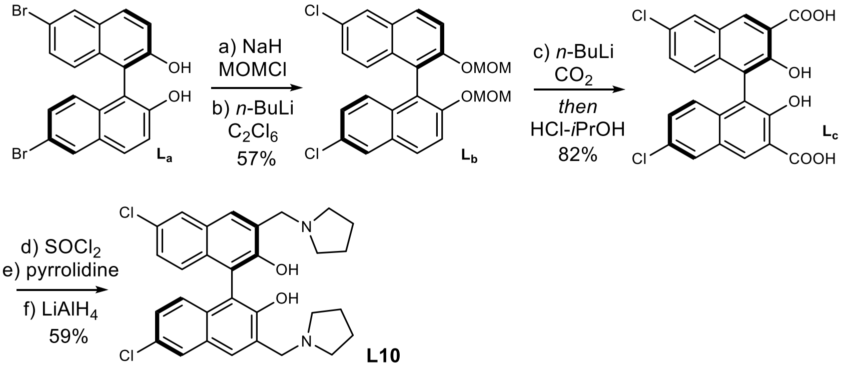

Supplementary Figure 23. Synthetic methods of chiral ligand **L10**

Step 1: **L**$_a$ (1.0 equiv) was dissolved in dried THF, after cooling to 0 °C, NaH (2.5 equiv) was added, then the mixture was stirred for further 0.5 hours, MOMCl (2.5 equiv) was added. After the reaction was completed, H_2O was carefully added, then the mixture was extracted with EA, dried with Na_2SO_4, filtration, and concentrated to give a crude product which was used directly in the next step without further purification. To the crude product in dried THF, *n*BuLi (2.5 equiv) was added under an argon atmosphere at -78 °C, then C_2Cl_6 (3.0 equiv) in THF was added after 0.5 hours, the mixture was stirred at

room temperature overnight. After completed, the reaction was quenched with saturated NH_4Cl and extracted with CH_2Cl_2. The organic layer was dried over Na_2SO_4 and concentrated to give a crude product which was purified via flash column chromatography to give **L**$_b$ in 57% yield.

Step 2: **L**$_b$ (1.0 equiv) was dissolved in dried Et_2O, *n*BuLi (2.5 equiv) was added at room temperature, then the mixture was stirred for further 2 hours at the same temperature, CO_2 gas was pumped through a balloon at 0 °C for 0.5 hours. After the reaction was completed, H_2O was added to queched the reaction, the aqueous was separated and acidified to PH=2 with hydrochloric acid. The mixture was extracted with EA, dried with Na_2SO_4, filtration, and concentrated to give a crude product **L**$_c$ in 82% yield.

Step 3: **L**$_c$ obtained above was treated with $SOCl_2$ for 4 hours at reflux. Removal of the excess $SOCl_2$ in vacuo to afforded the crude acyl-chloride as a dark brown oil. A solution of pyrrolidine (10 equiv) in THF was then added dropwise to the chloride in THF at 0 °C. The reaction mixture was stirred at room temperature overnight. After the reaction was completed, the mixture was poured into 1 N HCl. Extracted with CH_2Cl_2, dried with Na_2SO_4, filtration, and concentrated to give a crude product which was purified via flash column chromatography to give amide in 70% yield.

Step 4: To a solution of above amide (2.5 mmol) in dry THF (10 mL) at 0°C was added $LiAlH_4$ (2.2 equiv). The mixture was stirred at reflux for several hours. After completed, the mixture was cooled to 0 °C, quenched with saturated KF solution, filtered through celite, and purified via flash column chromatography to give pure **L10** (85% yield). **^{1}H NMR** (300 MHz, $CDCl_3$) δ 11.89 (s, 2H), 7.74 (s, 2H), 7.53 (s, 2H), 7.23 – 6.98 (m, 4H), 4.30 (d, J = 13.7 Hz, 2H), 3.90 (d, J = 13.8 Hz, 2H), 2.68 (m, 8H), 1.80 (m, 8H). **^{13}C NMR** (75 MHz, $CDCl_3$) δ 154.5, 132.0, 128.7, 128.5, 126.8, 126.4, 126.1, 116.1, 59.4, 53.5, 23.8; **HRMS** (ESI): $C_{30}H_{30}Cl_2N_2NaO_2$ $[M+Na]^+$ calcd: 543.1577, found: 543.1568.

X-Ray analysis of L10

Supplementary Figure 24. X-ray analysis and relative data of **L10**

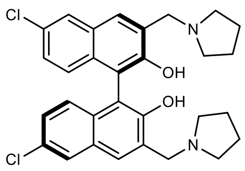

L10

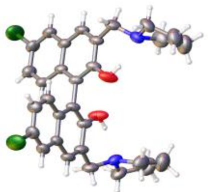

CCDC 1978958

Bond precision: C-C = 0.0043 A Wavelength=1.54184

Cell: a=6.6516(3) b=19.0036(8) c=21.3259(8)

alpha=90 beta=90 gamma=90

Temperature: 293 K

Calculated Reported

Volume	2695.69(19)	2695.7(2)
Space group	C 2 2 21	C 2 2 21
Hall group	C 2c 2	C 2c 2
Moiety formula	C30 H30 Cl2 N2 O2	C15 H15 Cl N O
Sum formula	C30 H30 Cl2 N2 O2	C15 H15 Cl N O
Mr	521.46	260.73
Dx,g cm-3	1.285	1.285
Z	4	8
Mu (mm-1)	2.397	2.397
F000	1096.0	1096.0
F000'	1101.60	
h,k,lmax	7,22,25	7,22,25
Nref	2364[1359]	2076
Tmin,Tmax	0.682,0.715	0.670,1.000
Tmin'	0.576	

Correction method= # Reported T Limits: Tmin=0.670 Tmax=1.000 AbsCorr = MULTI-SCAN

Data completeness= 1.53/0.88 Theta(max)= 66.411

R(reflections)= 0.0344(1918) wR2(reflections)= 0.0898(2076)

S = 1.060 Npar= 164

Flack x parameter = 0.034(13)

Supplementary Figure 25. ^{13}C NMR spectrum of compound **1a***

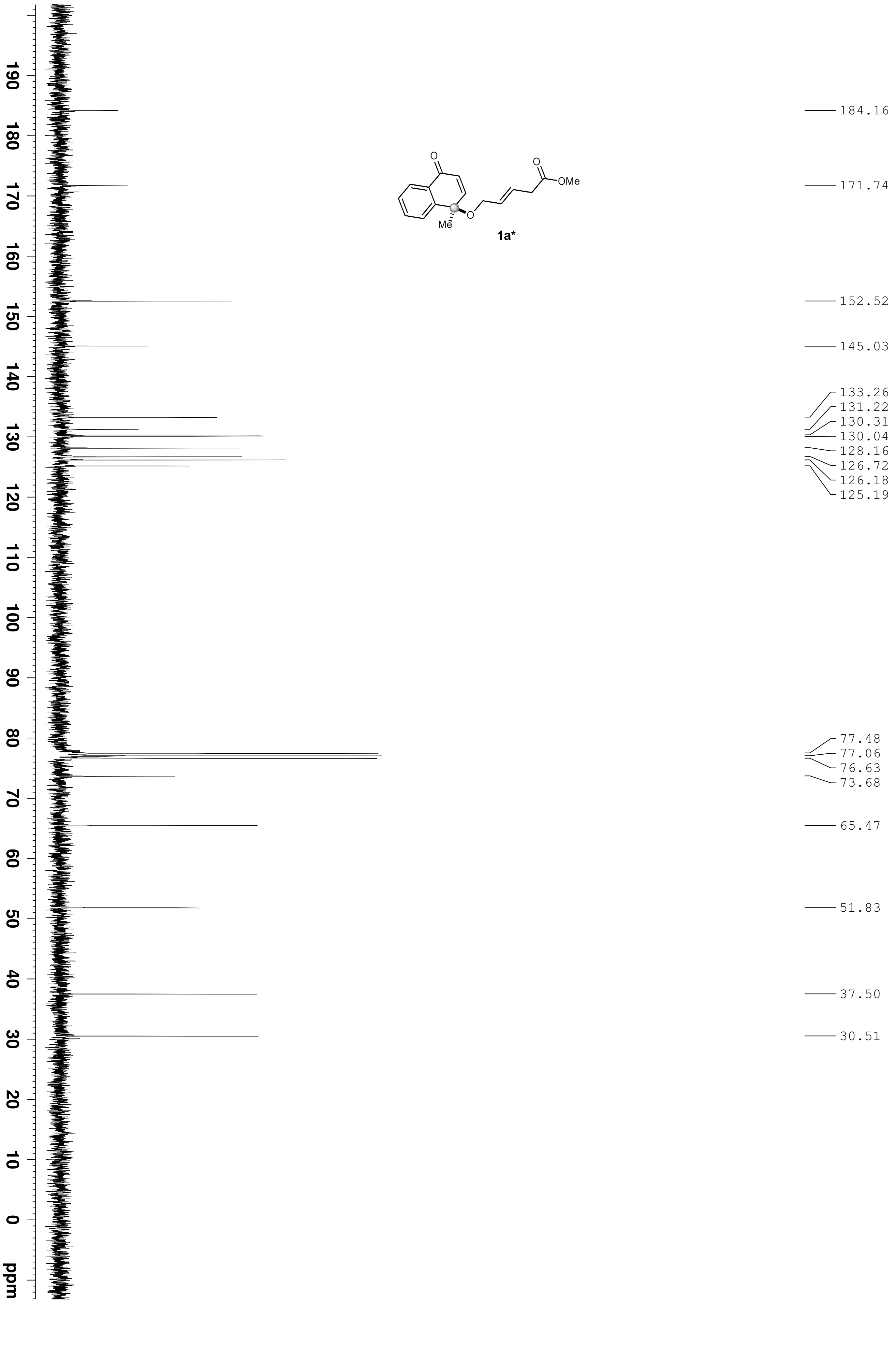

Supplementary Figure 26. ^{1}H NMR spectrum of compound **1a***

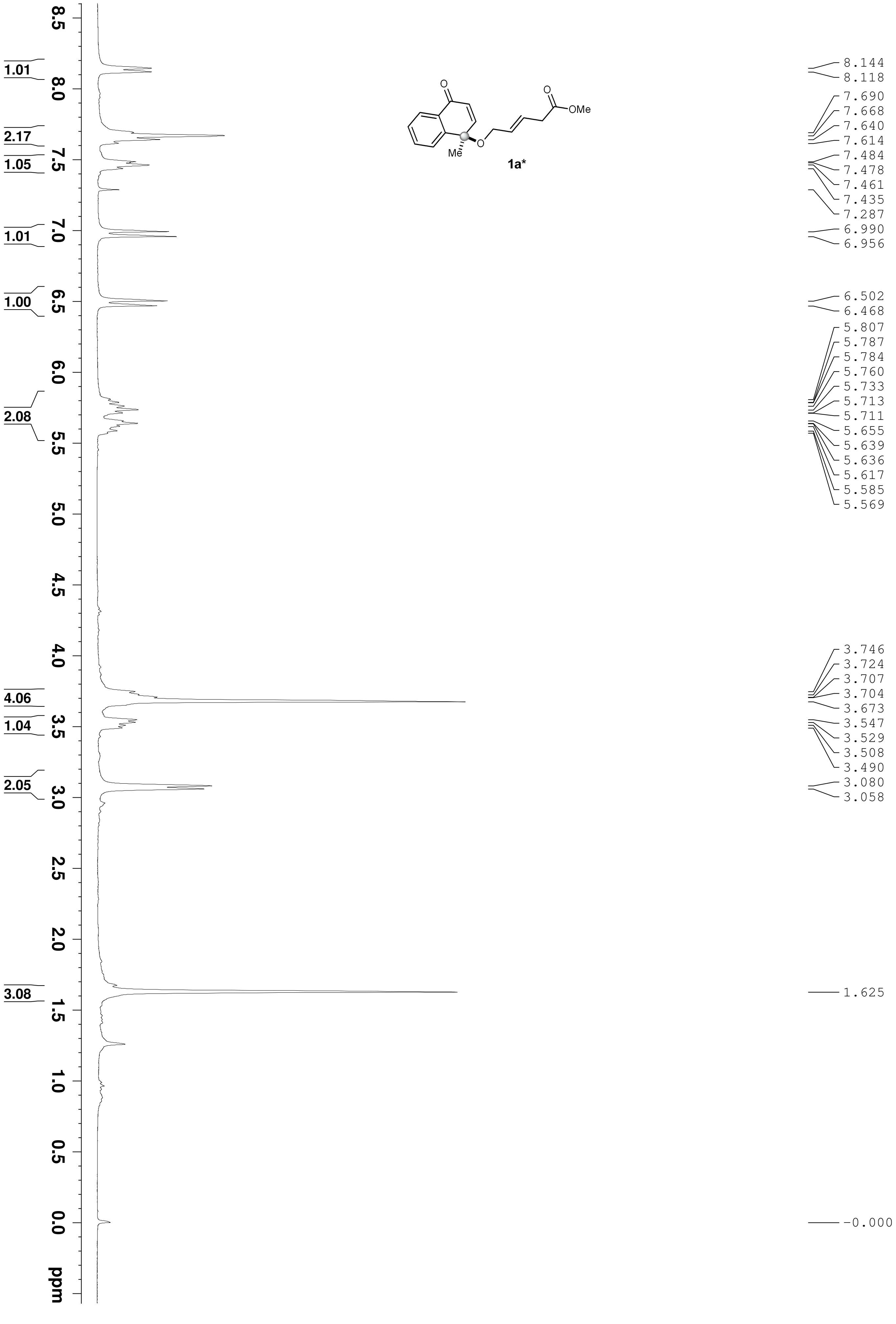

Supplementary Figure 27. ^{13}C NMR spectrum of compound **1b***

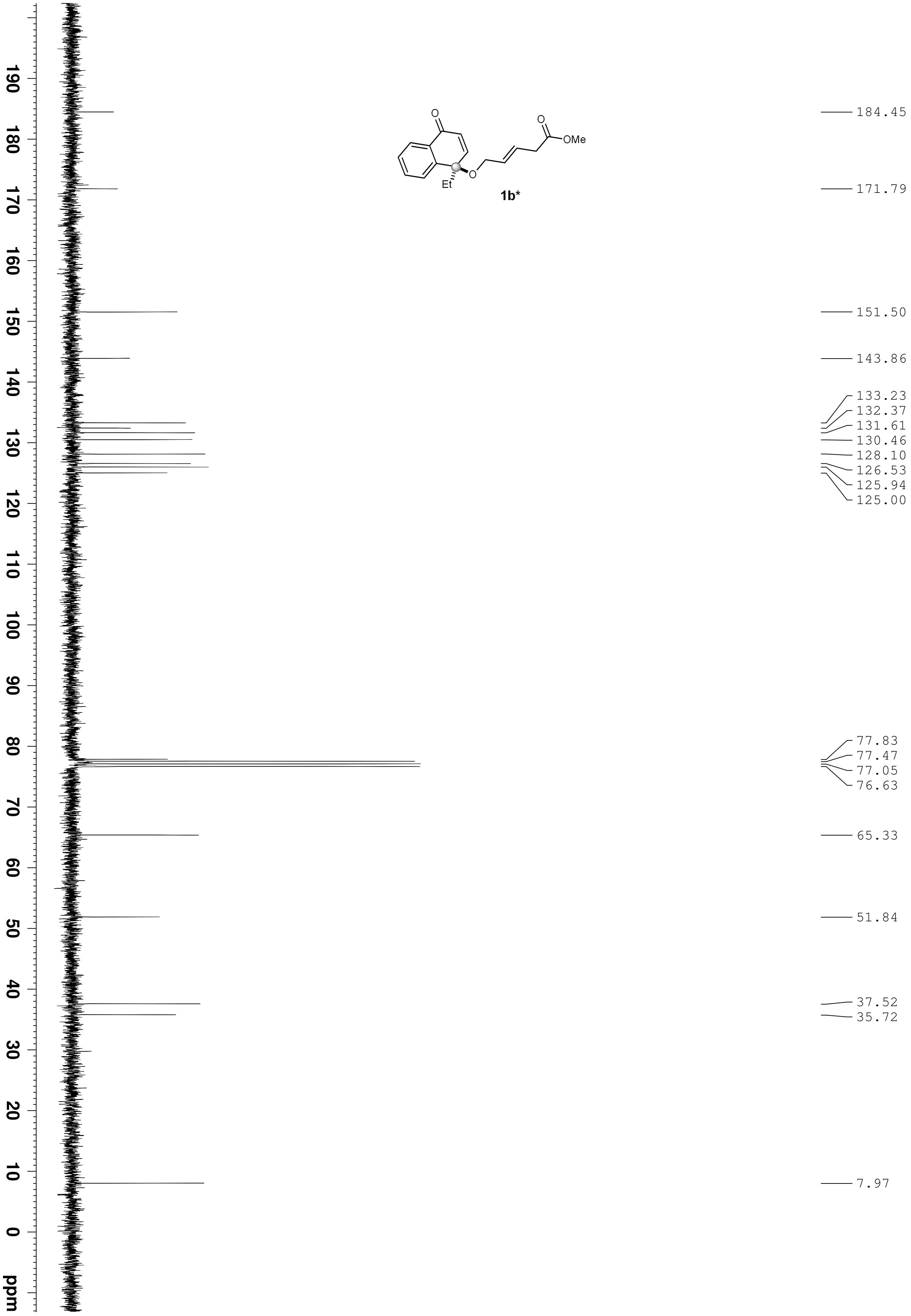

Supplementary Figure 28. ^{1}H NMR spectrum of compound **1b***

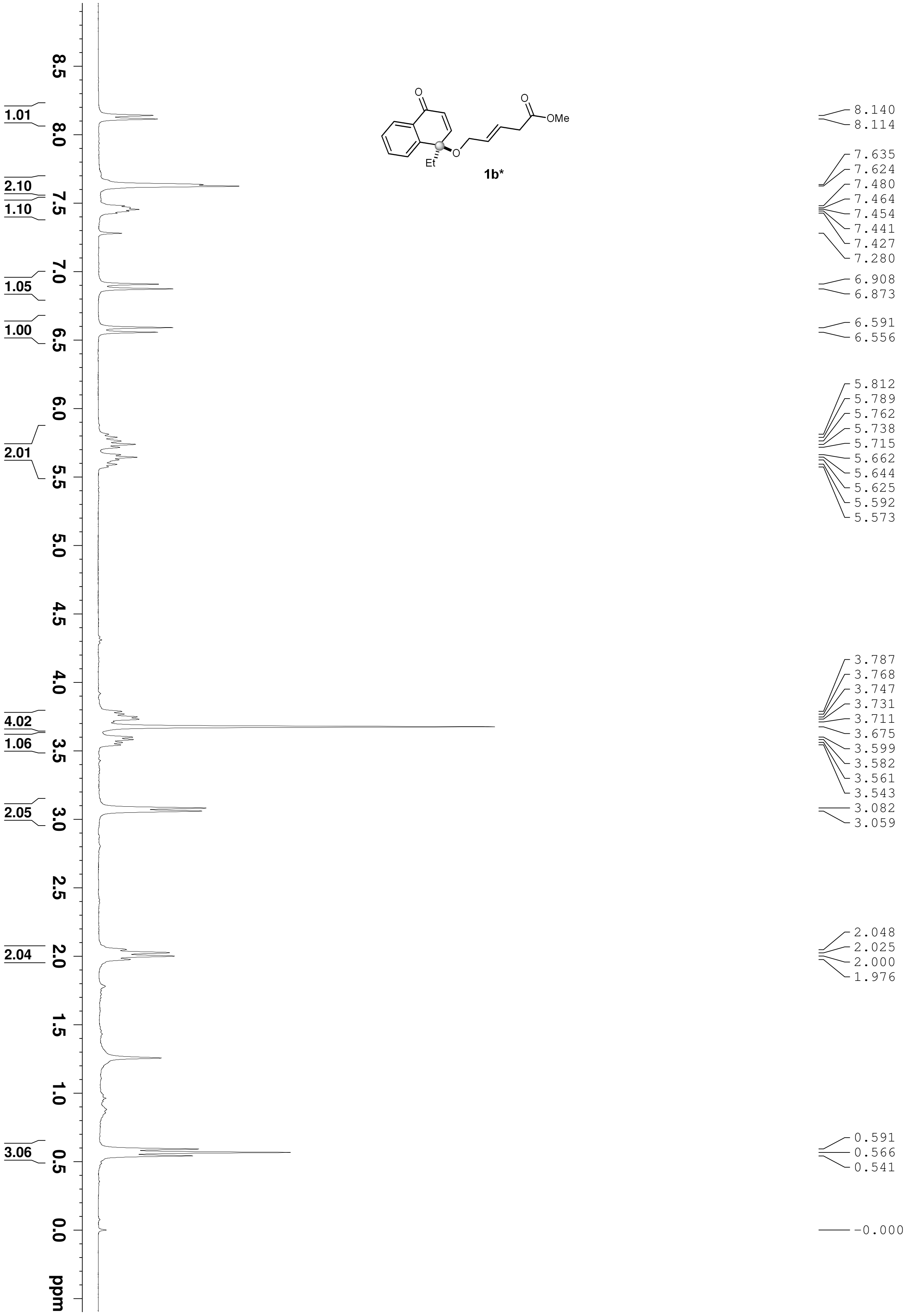

Supplementary Figure 29. ^{13}C NMR spectrum of compound **1c***

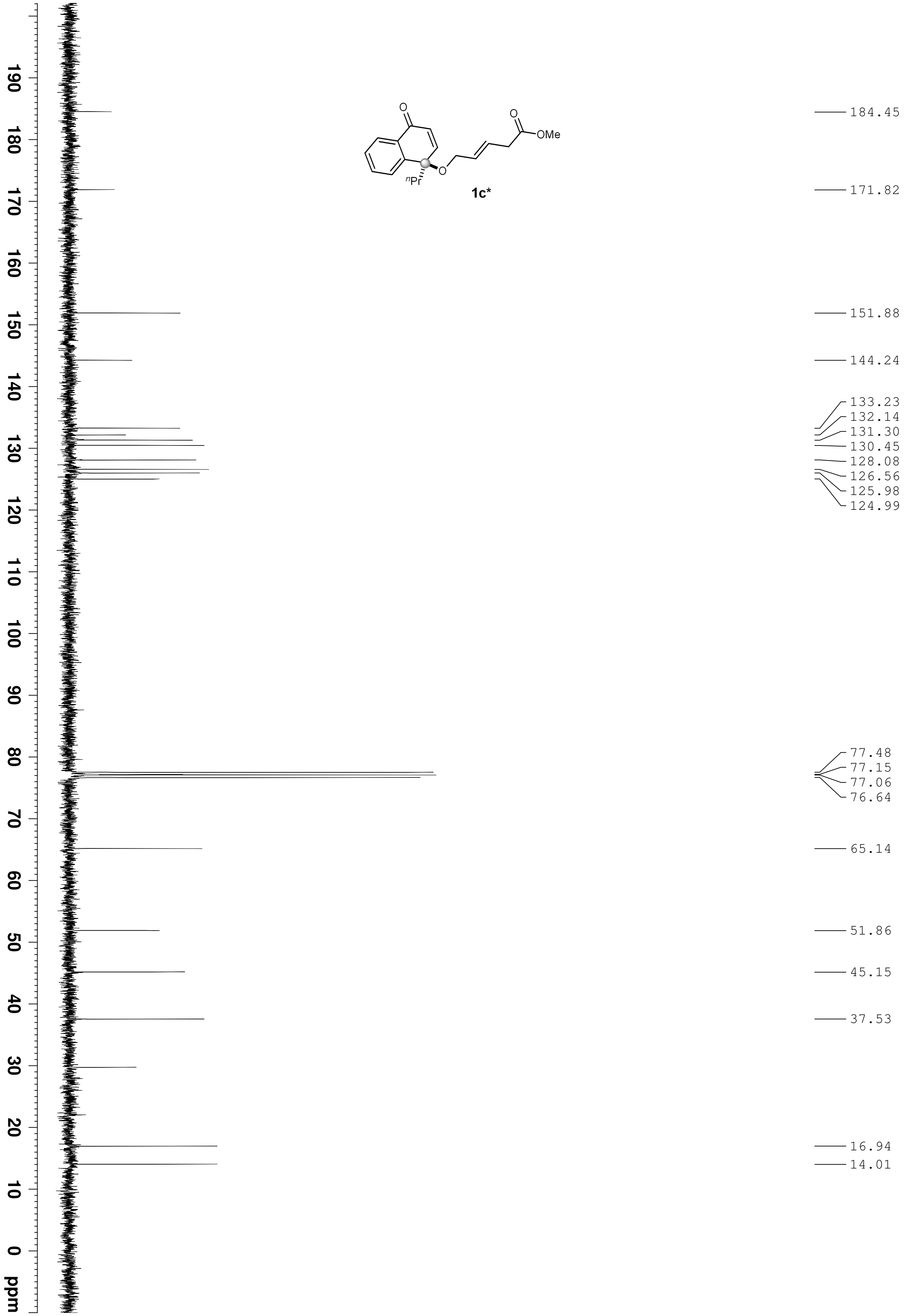

Supplementary Figure 30. ^{1}H NMR spectrum of compound **1c***

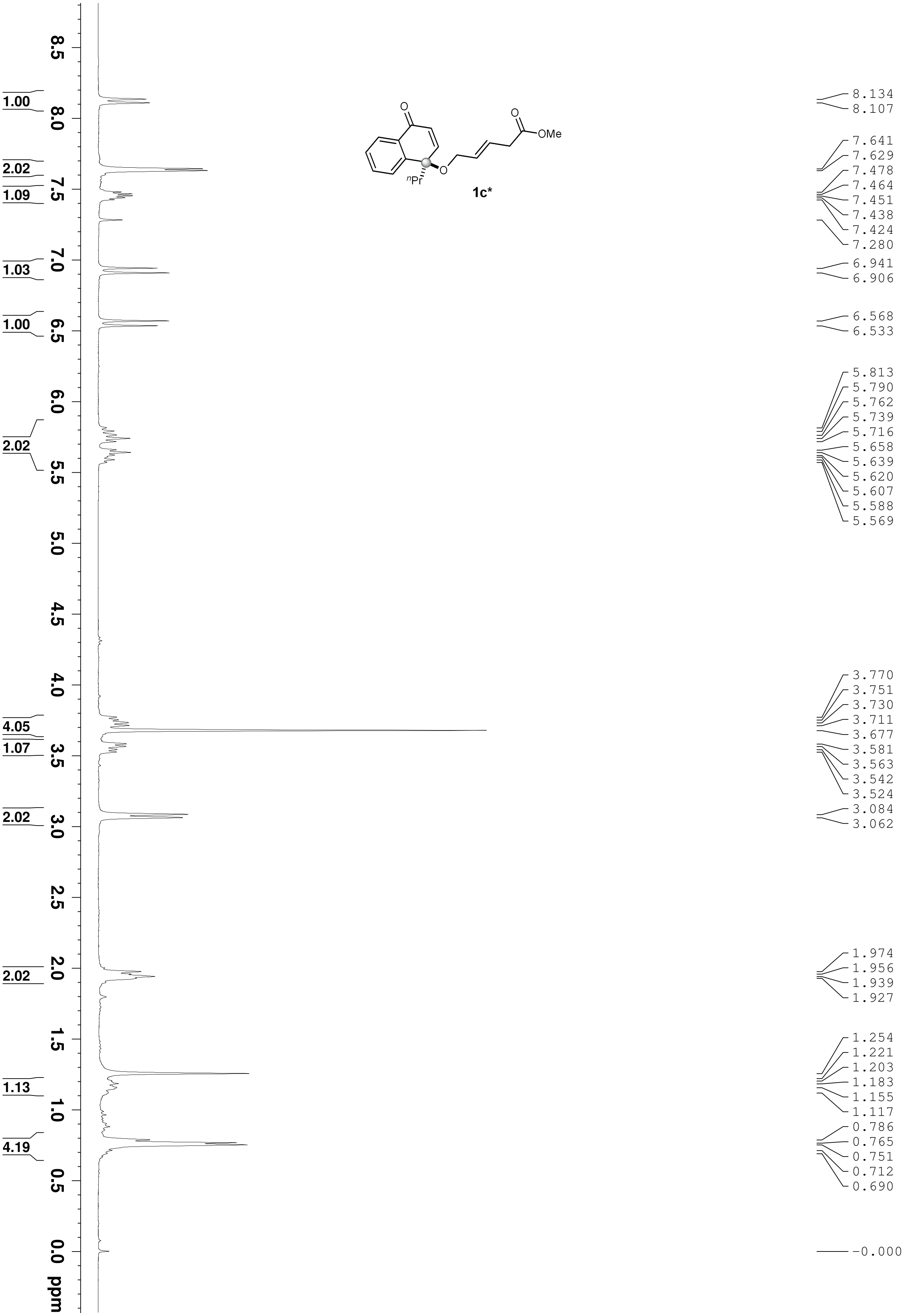

Supplementary Figure 31. ^{13}C NMR spectrum of compound **1d***

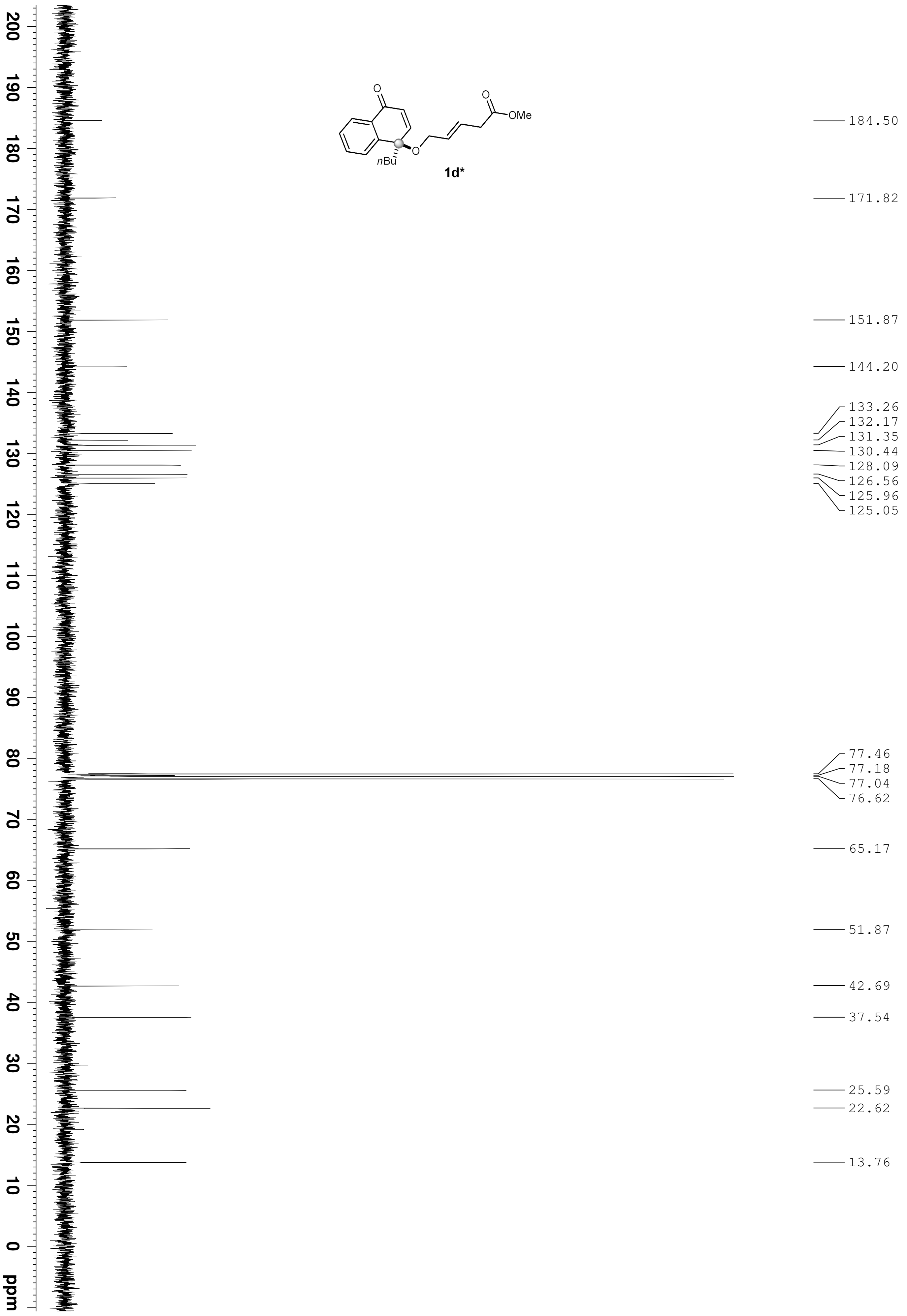

Supplementary Figure 32. ^{1}H NMR spectrum of compound **1d***

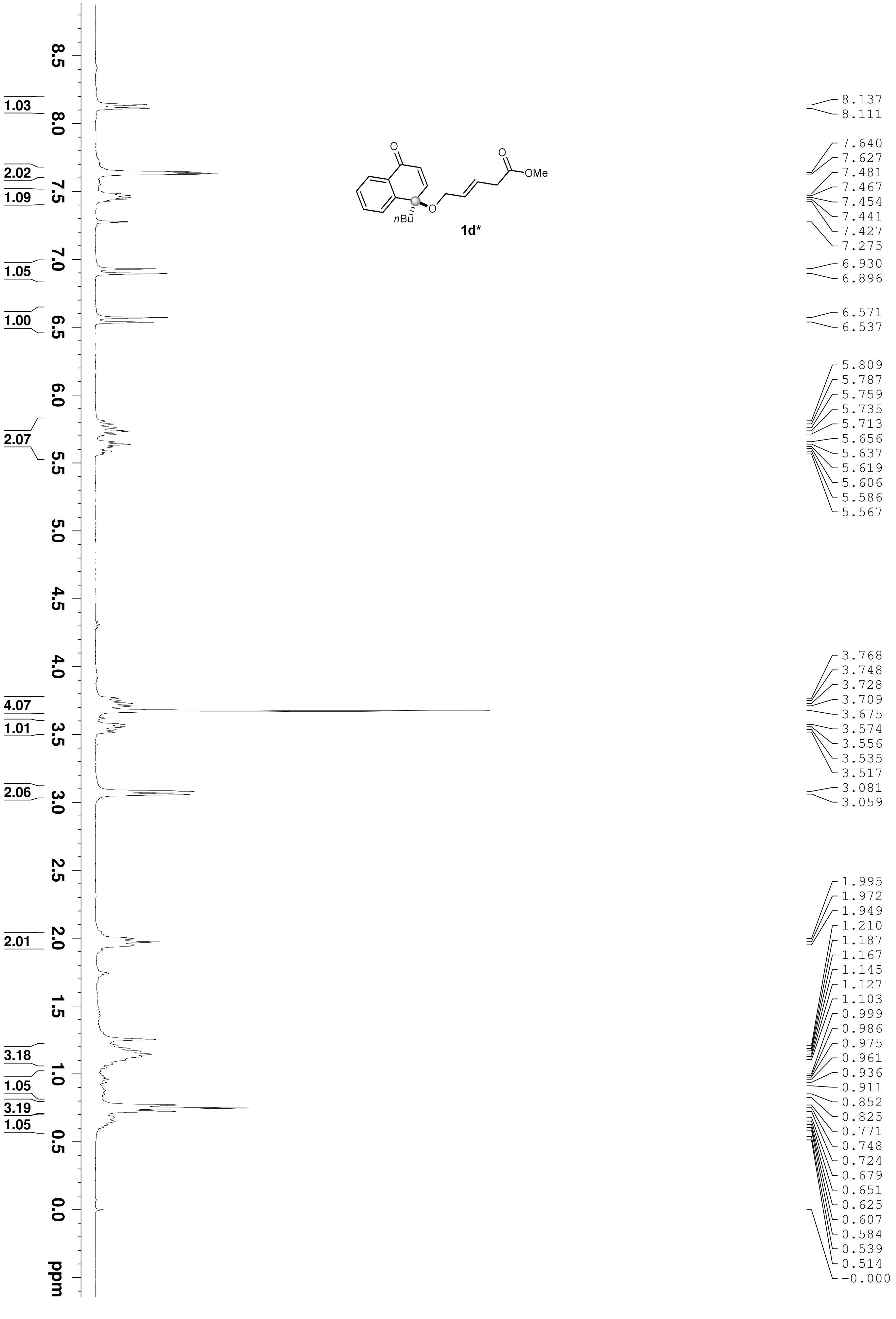

Supplementary Figure 33. ^{13}C NMR spectrum of compound **1e***

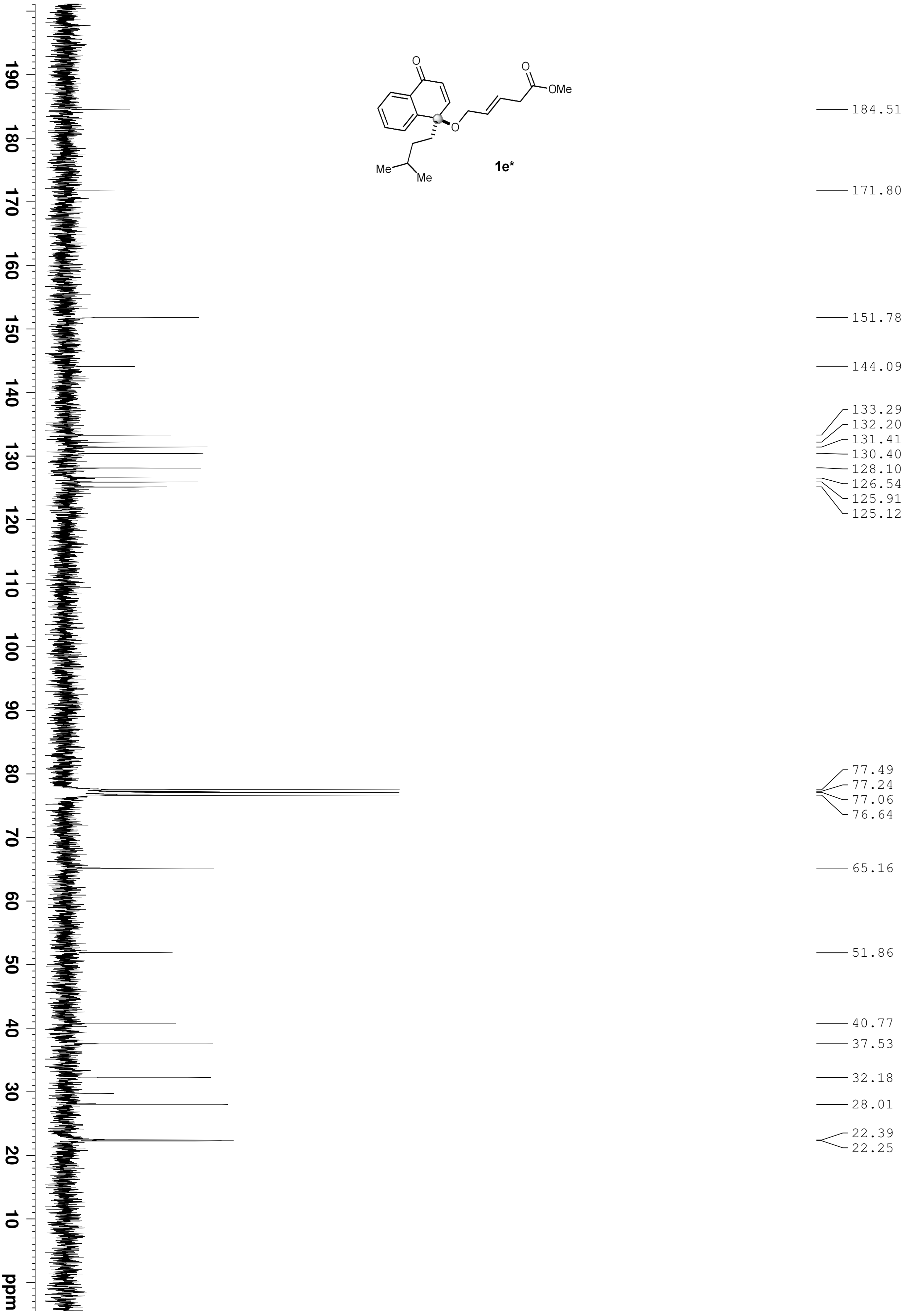

Supplementary Figure 34. ^{1}H NMR spectrum of compound **1e***

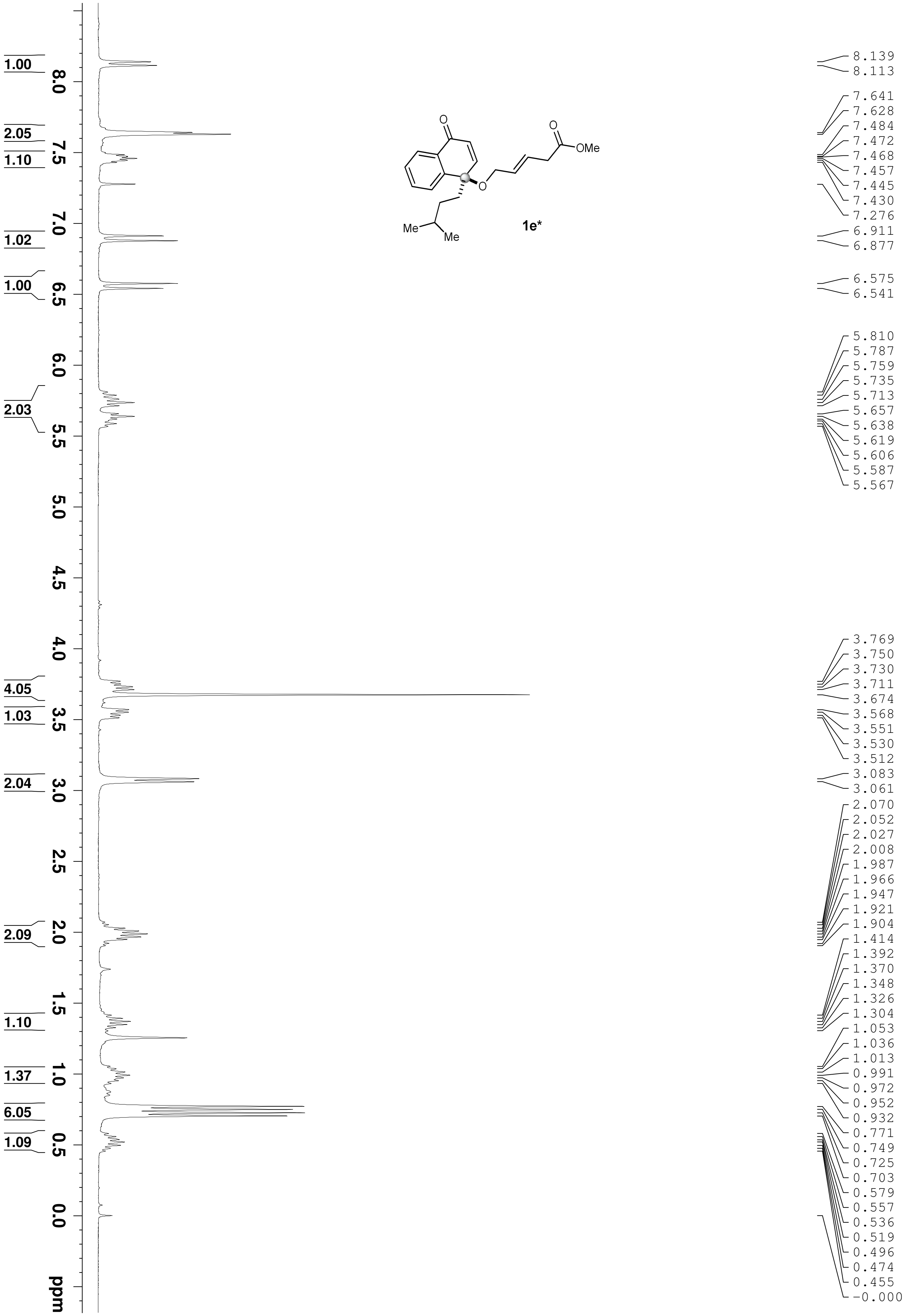

Supplementary Figure 35. ^{13}C NMR spectrum of compound **1f***

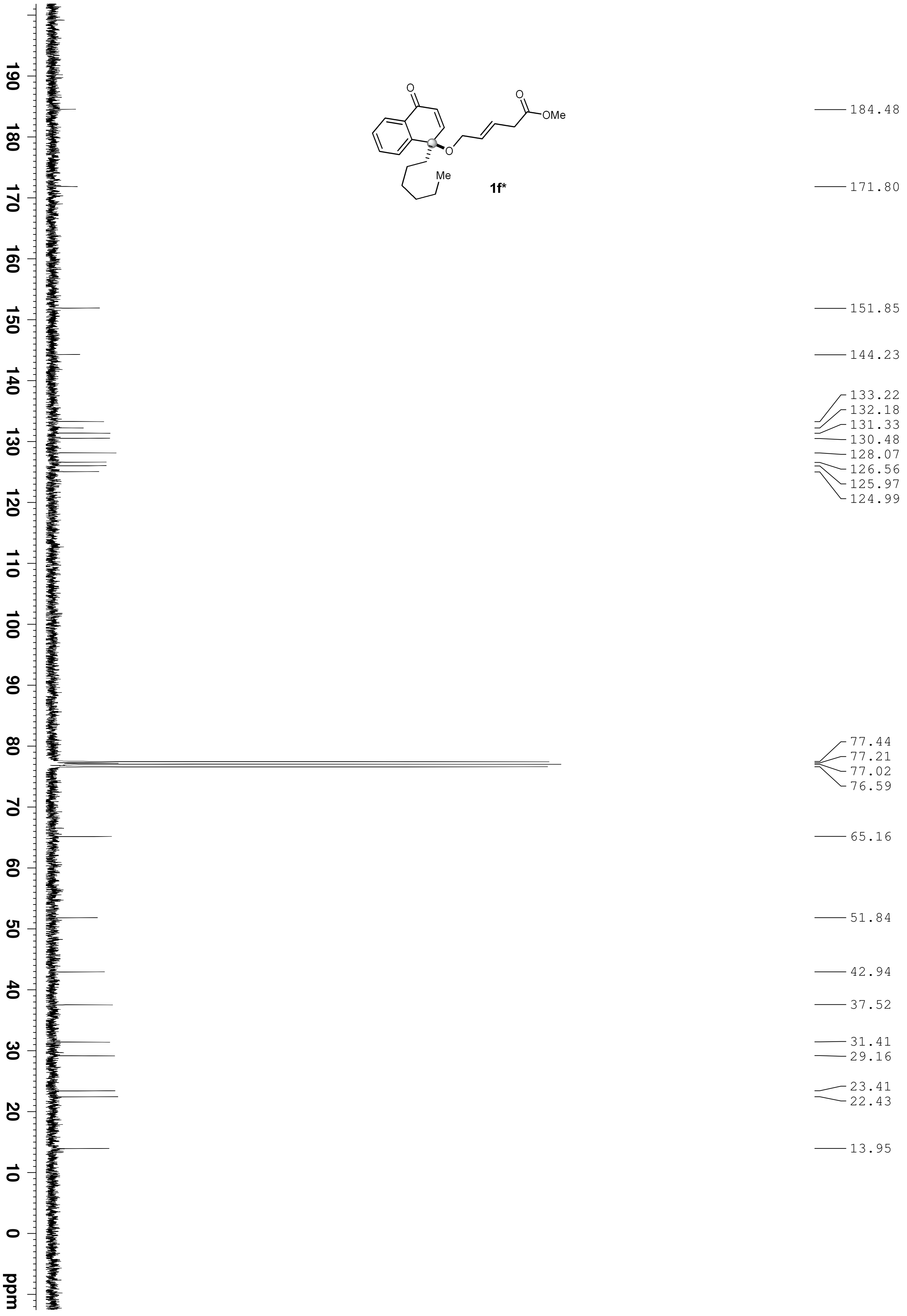

Supplementary Figure 36. ^{1}H NMR spectrum of compound **1f***

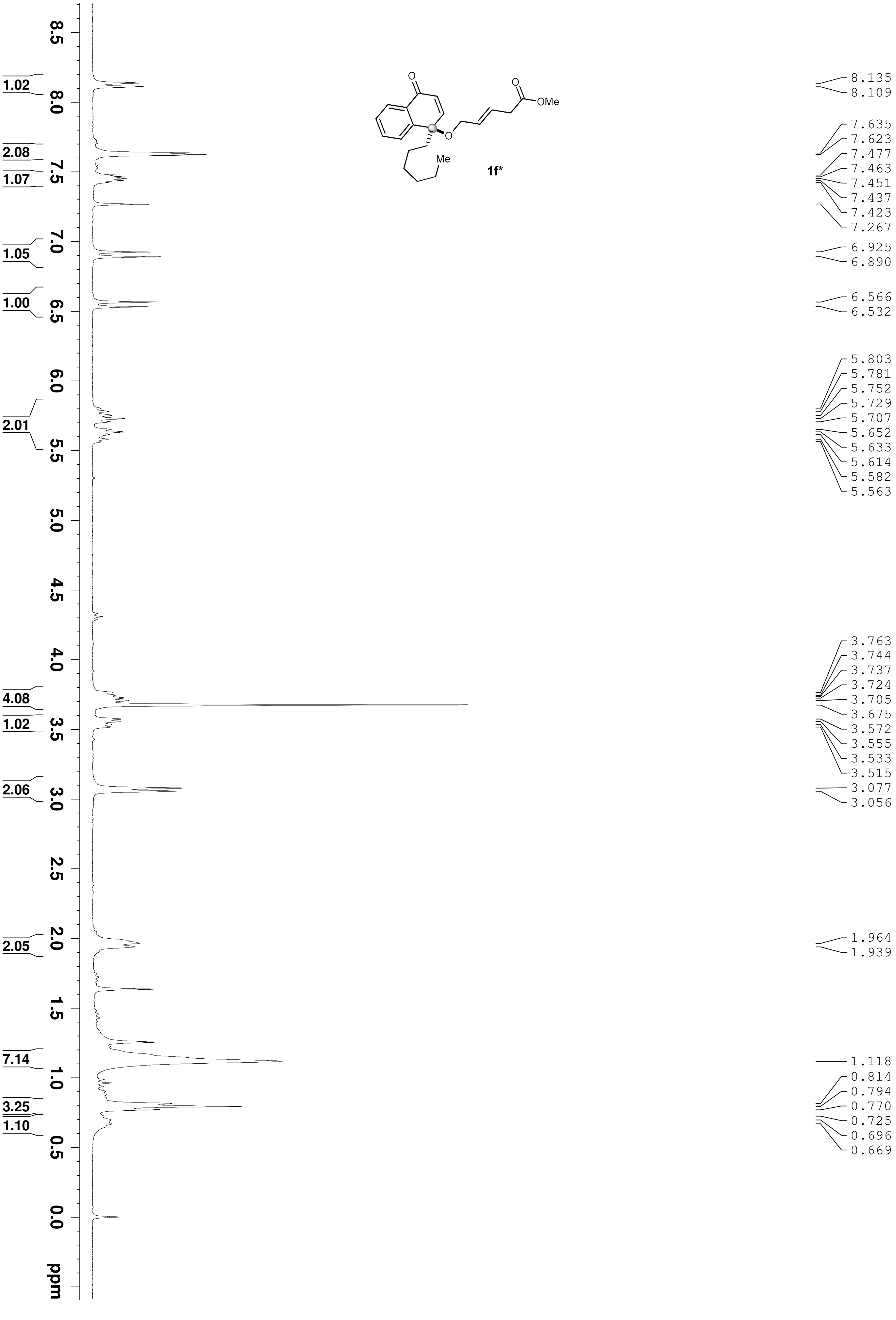

Supplementary Figure 37. ^{13}C NMR spectrum of compound **1g***

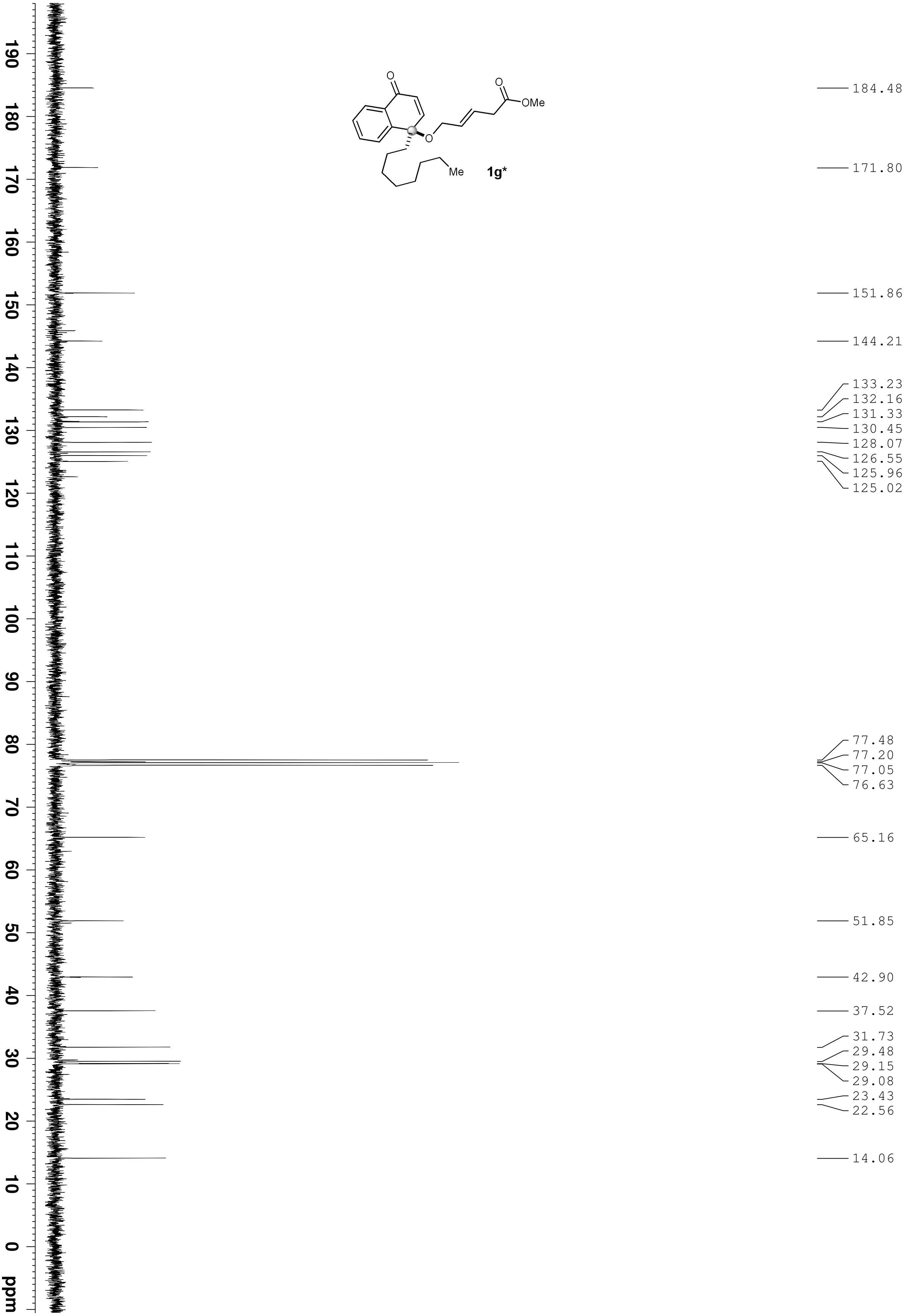

Supplementary Figure 38. ^{1}H NMR spectrum of compound **1g***

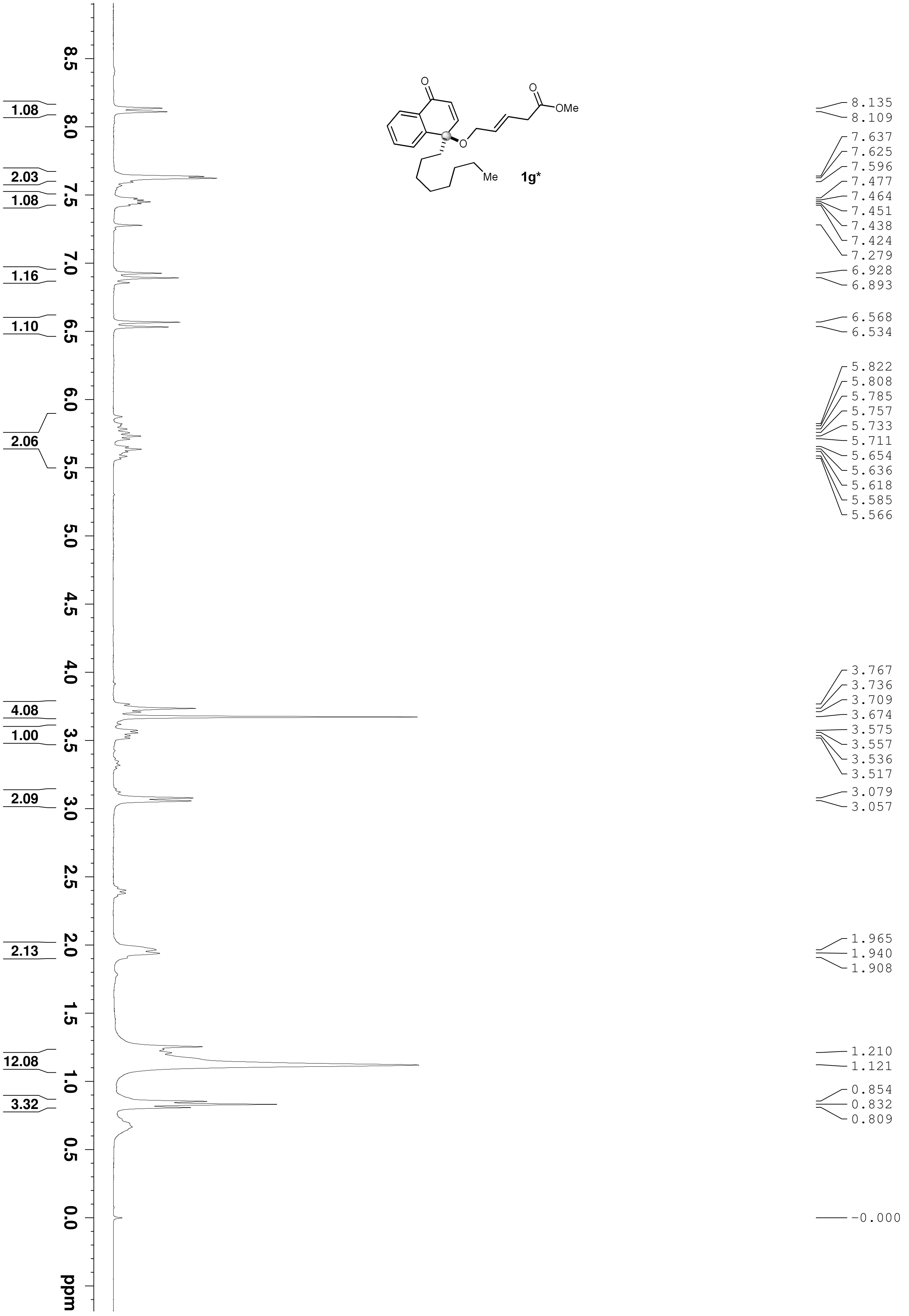

Supplementary Figure 39. ^{13}C NMR spectrum of compound **1h***

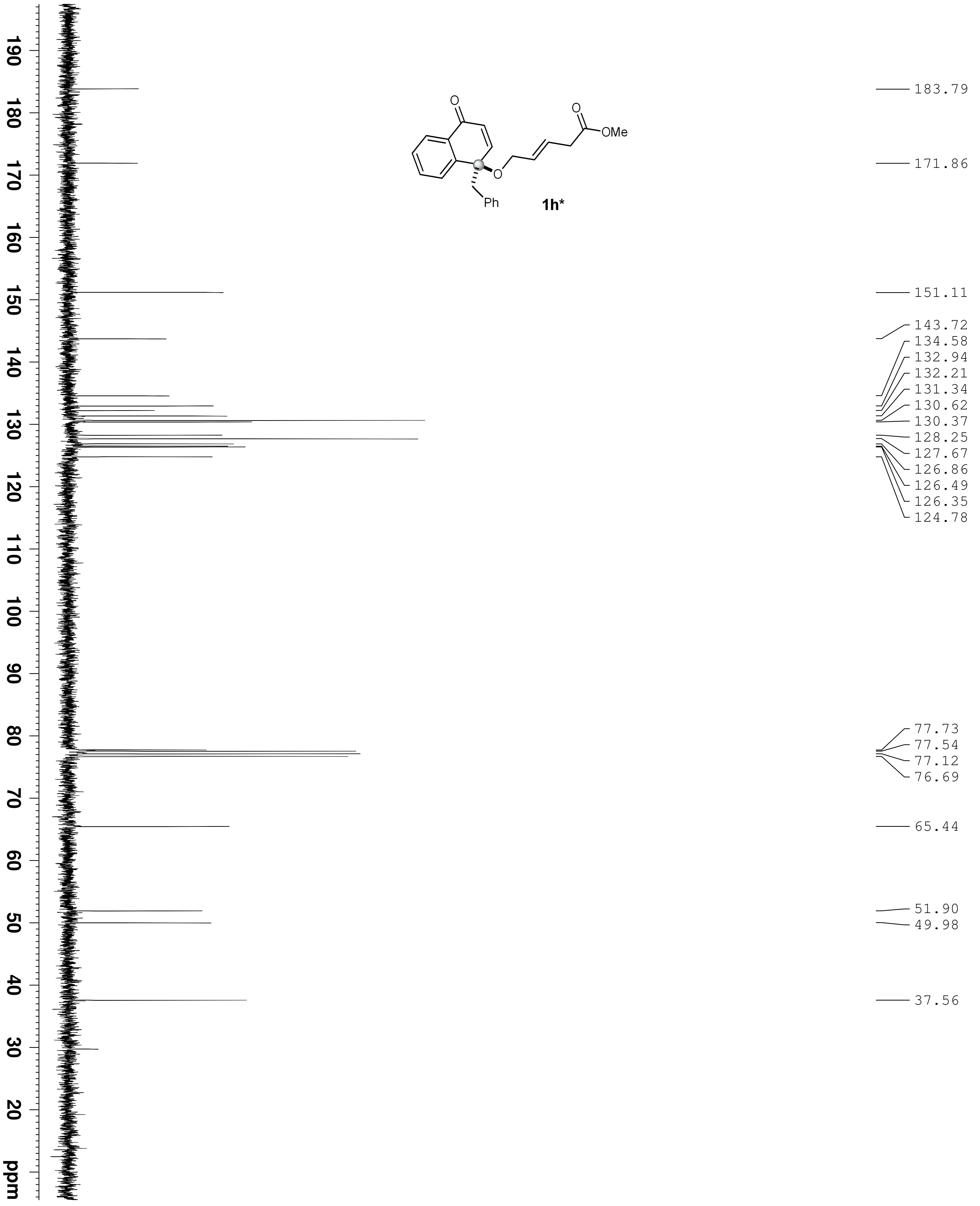

Supplementary Figure 40. ^{1}H NMR spectrum of compound **1h***

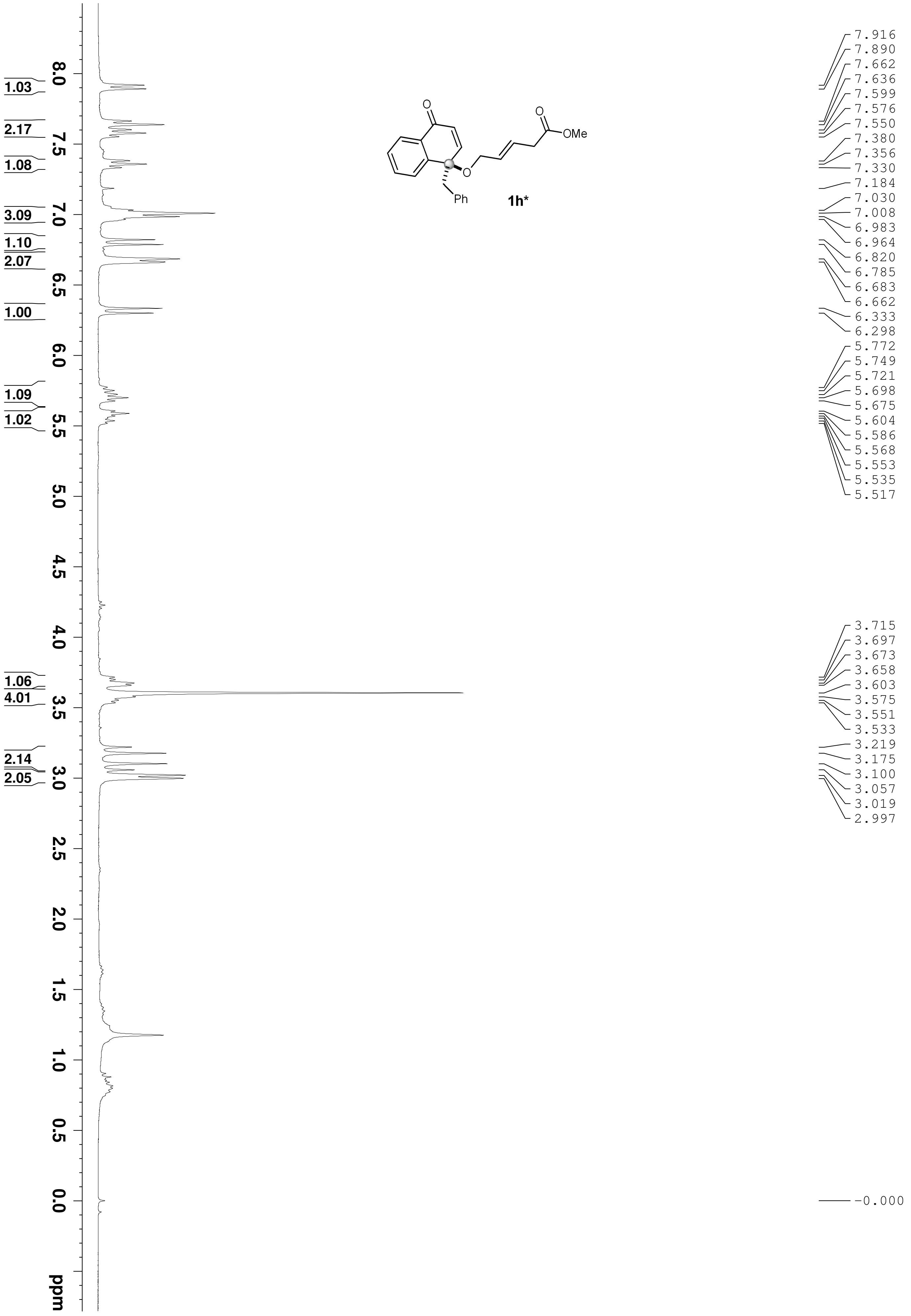

Supplementary Figure 41. ^{13}C NMR spectrum of compound **1i***

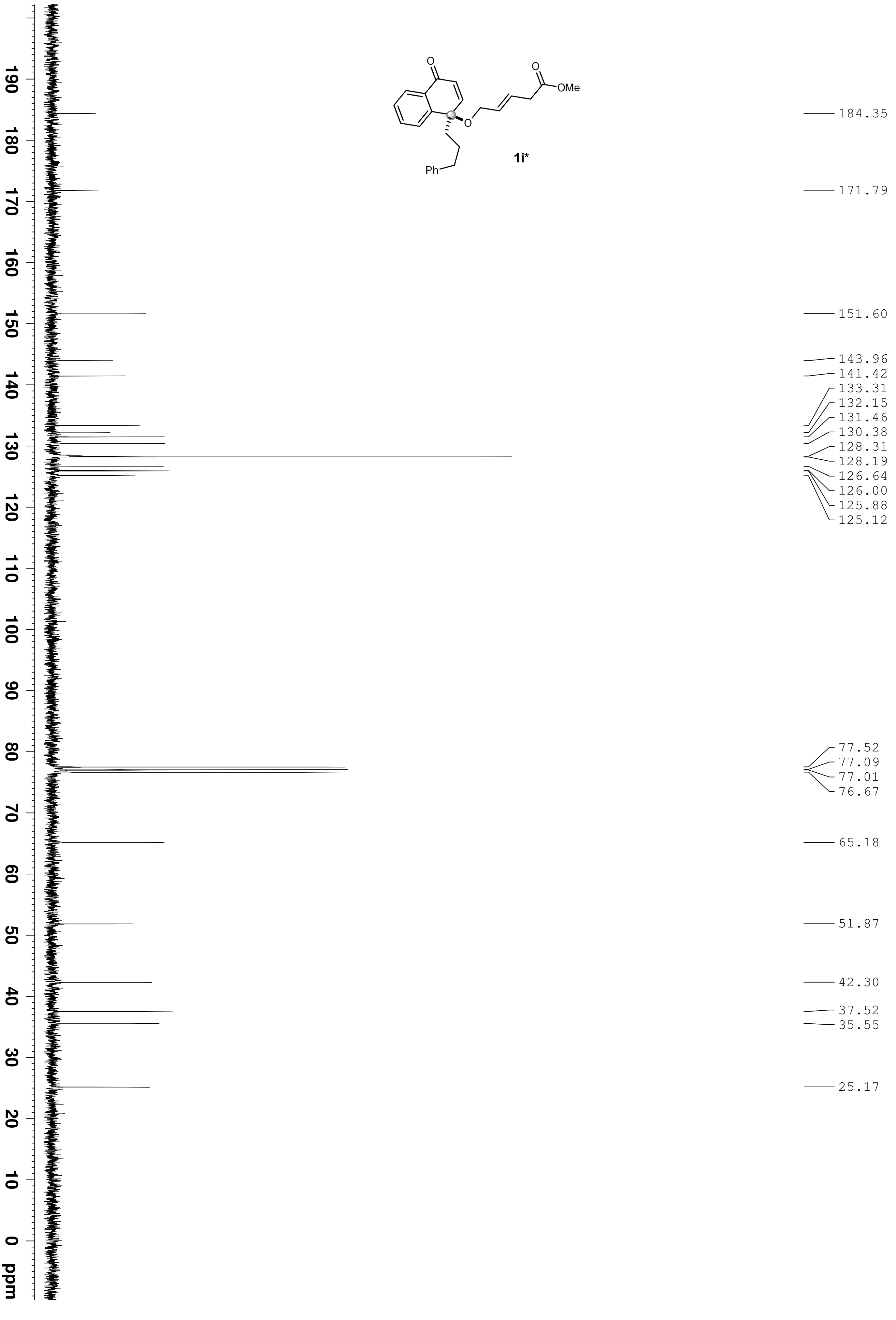

Supplementary Figure 42. ^{1}H NMR spectrum of compound **1i***

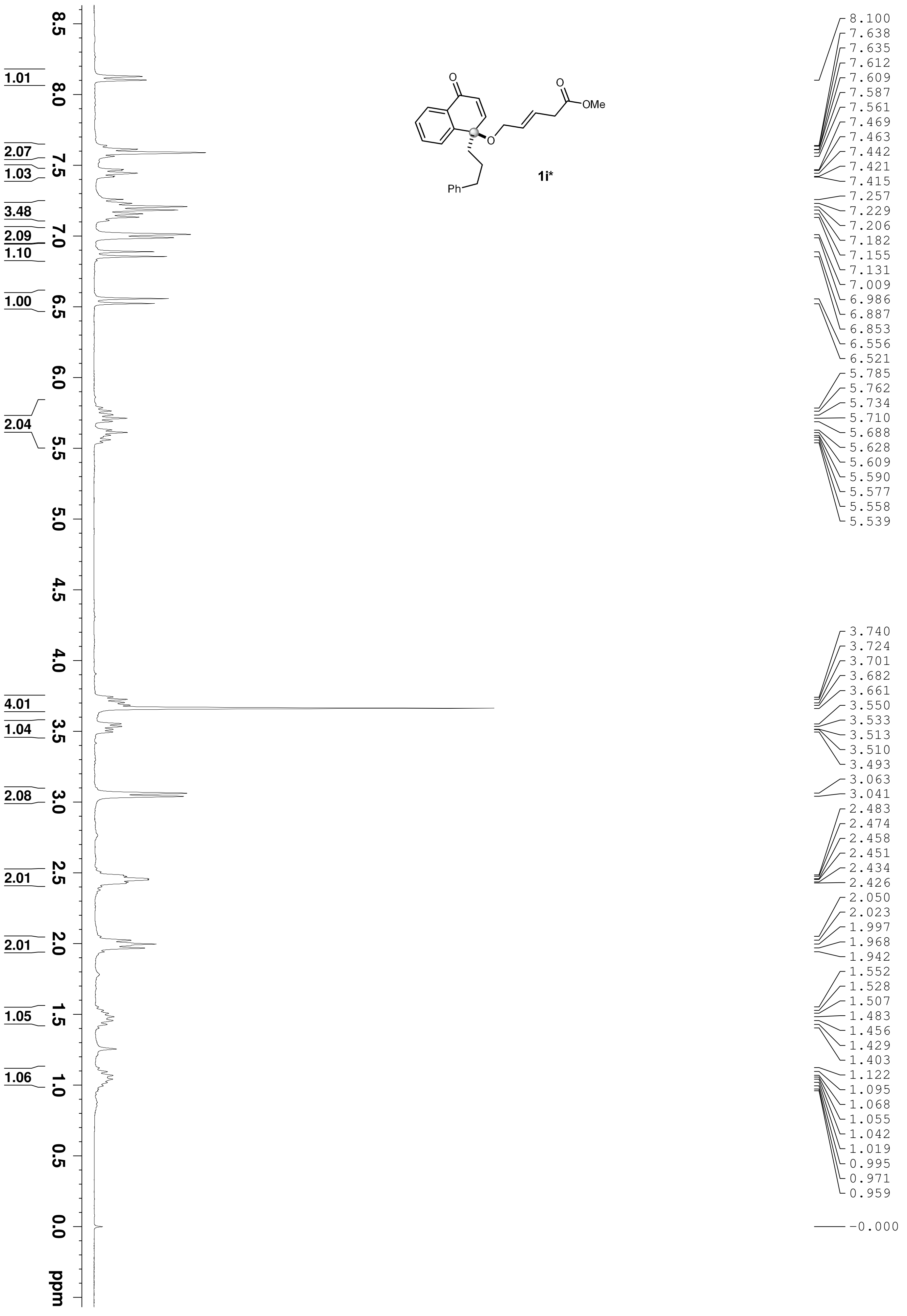

Supplementary Figure 43. ^{13}C NMR spectrum of compound **1j***

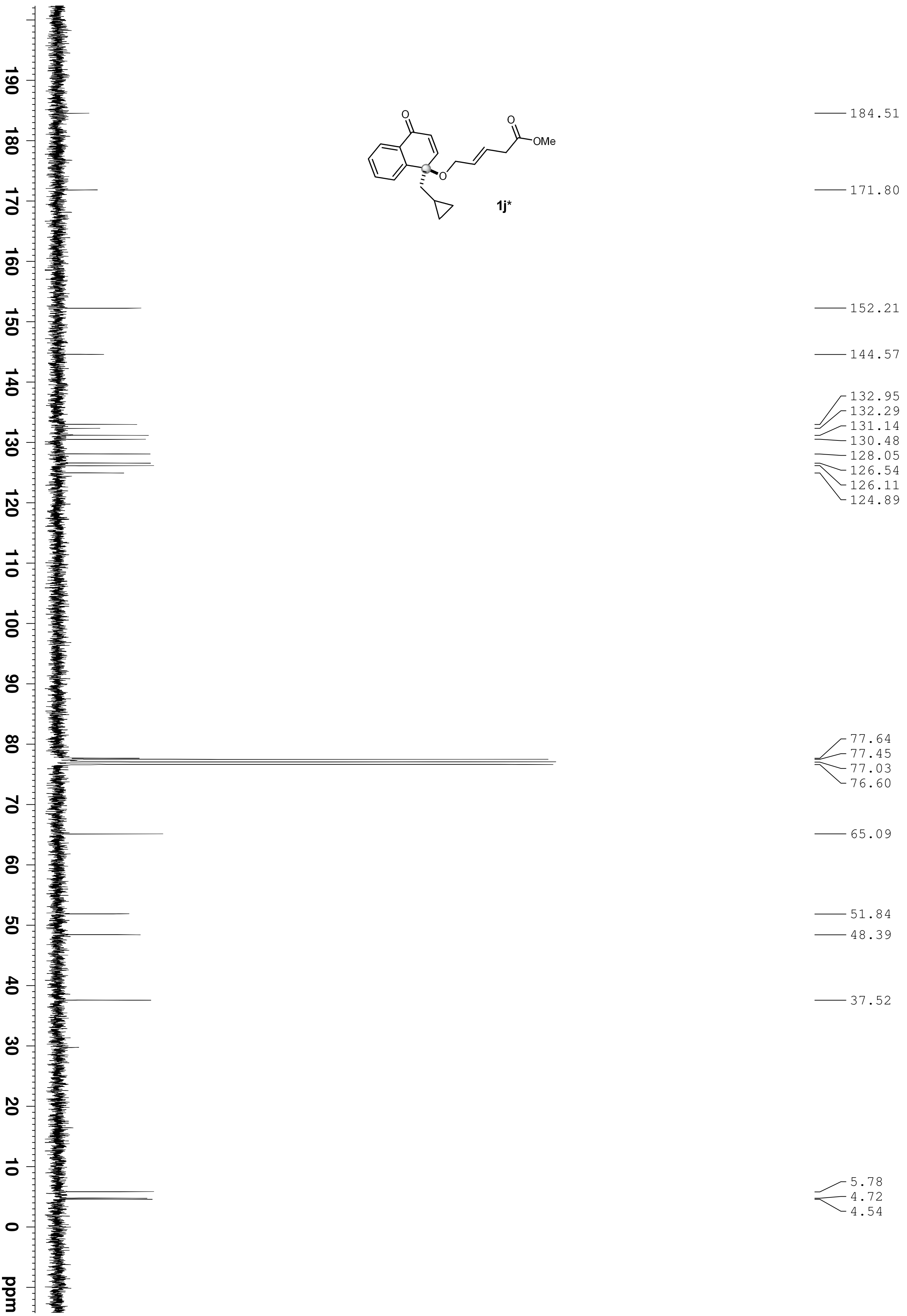

Supplementary Figure 44. ^{1}H NMR spectrum of compound **1j***

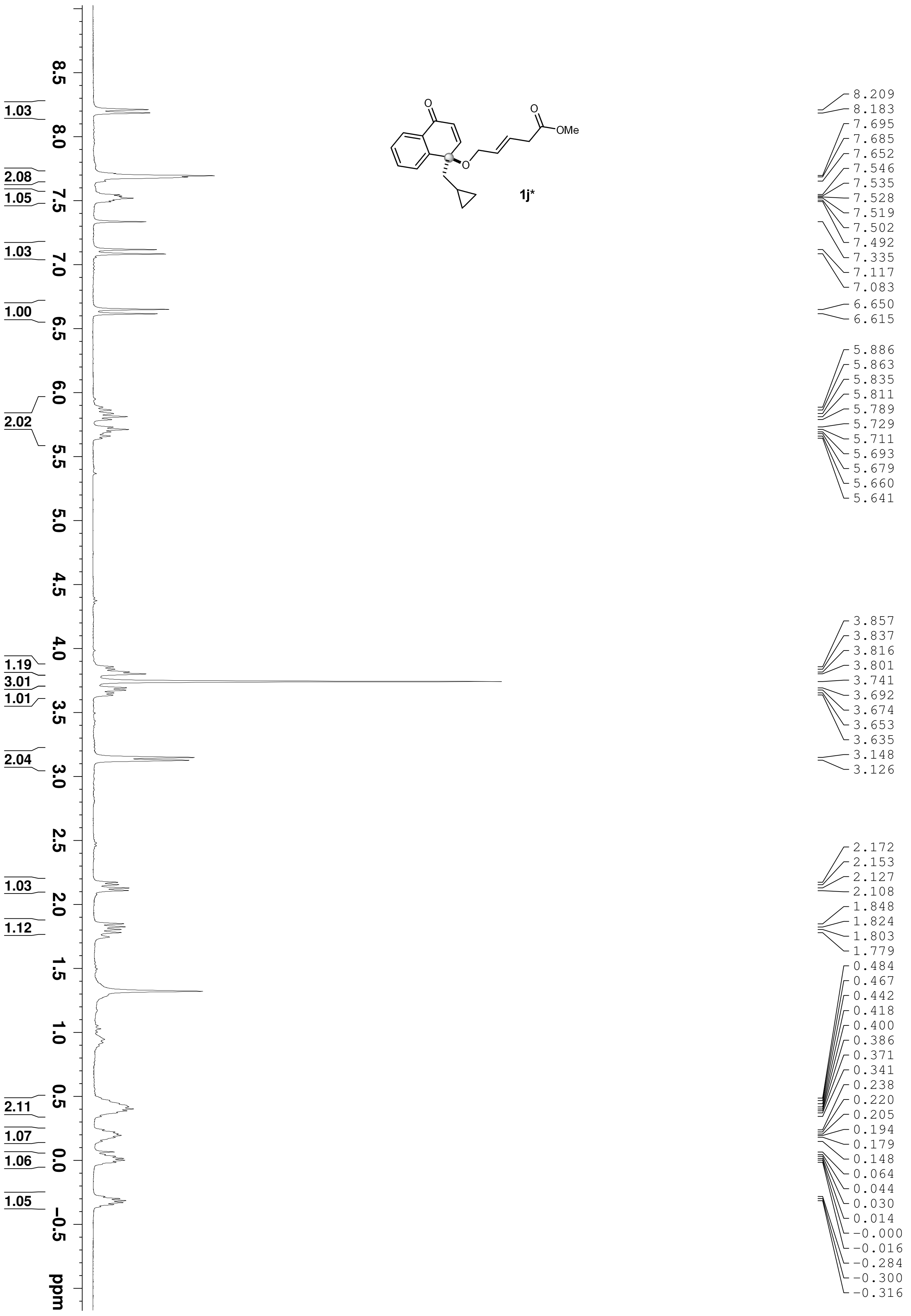

Supplementary Figure 45. ^{13}C NMR spectrum of compound **1k***

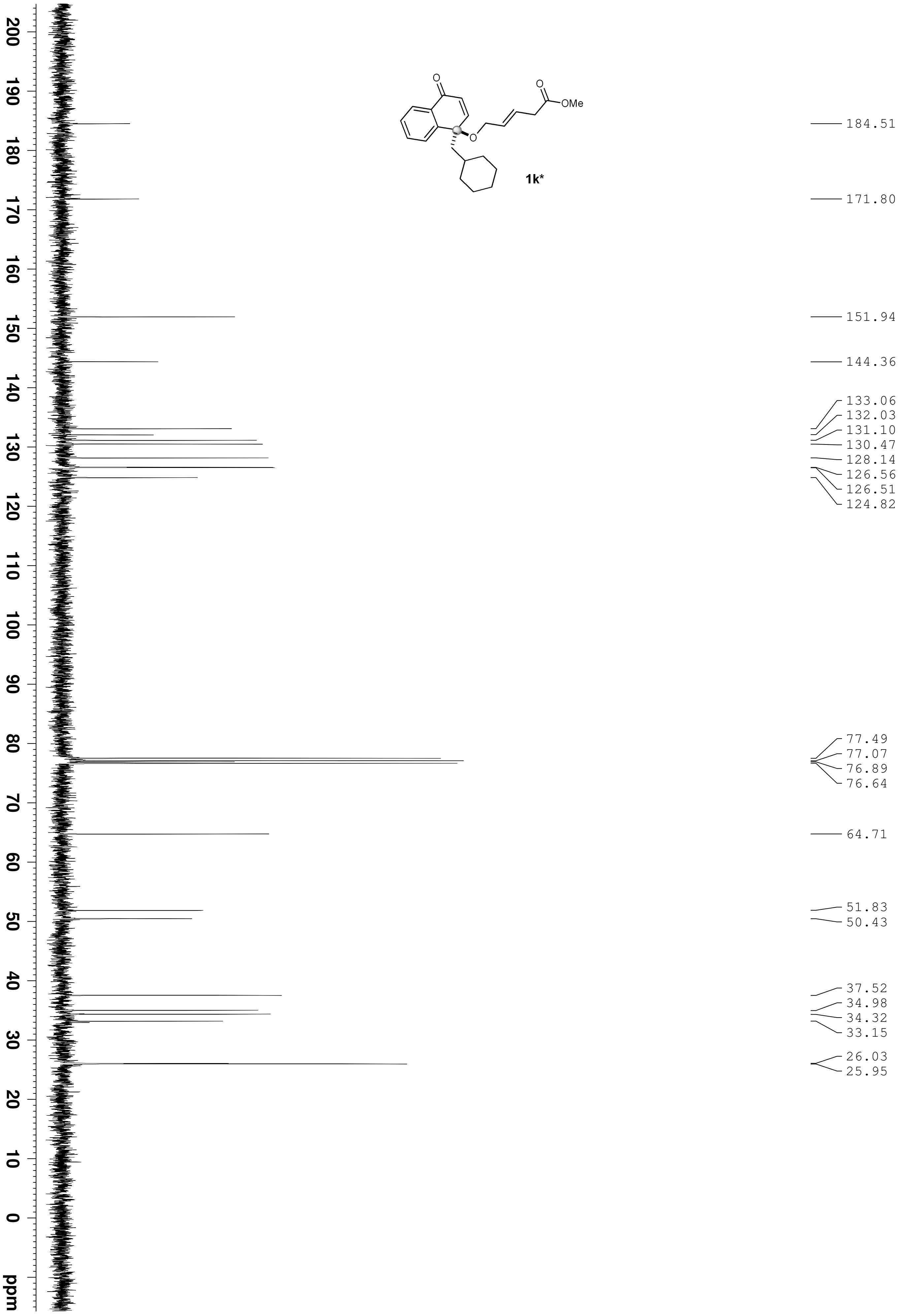

Supplementary Figure 46. ^{1}H NMR spectrum of compound **1k***

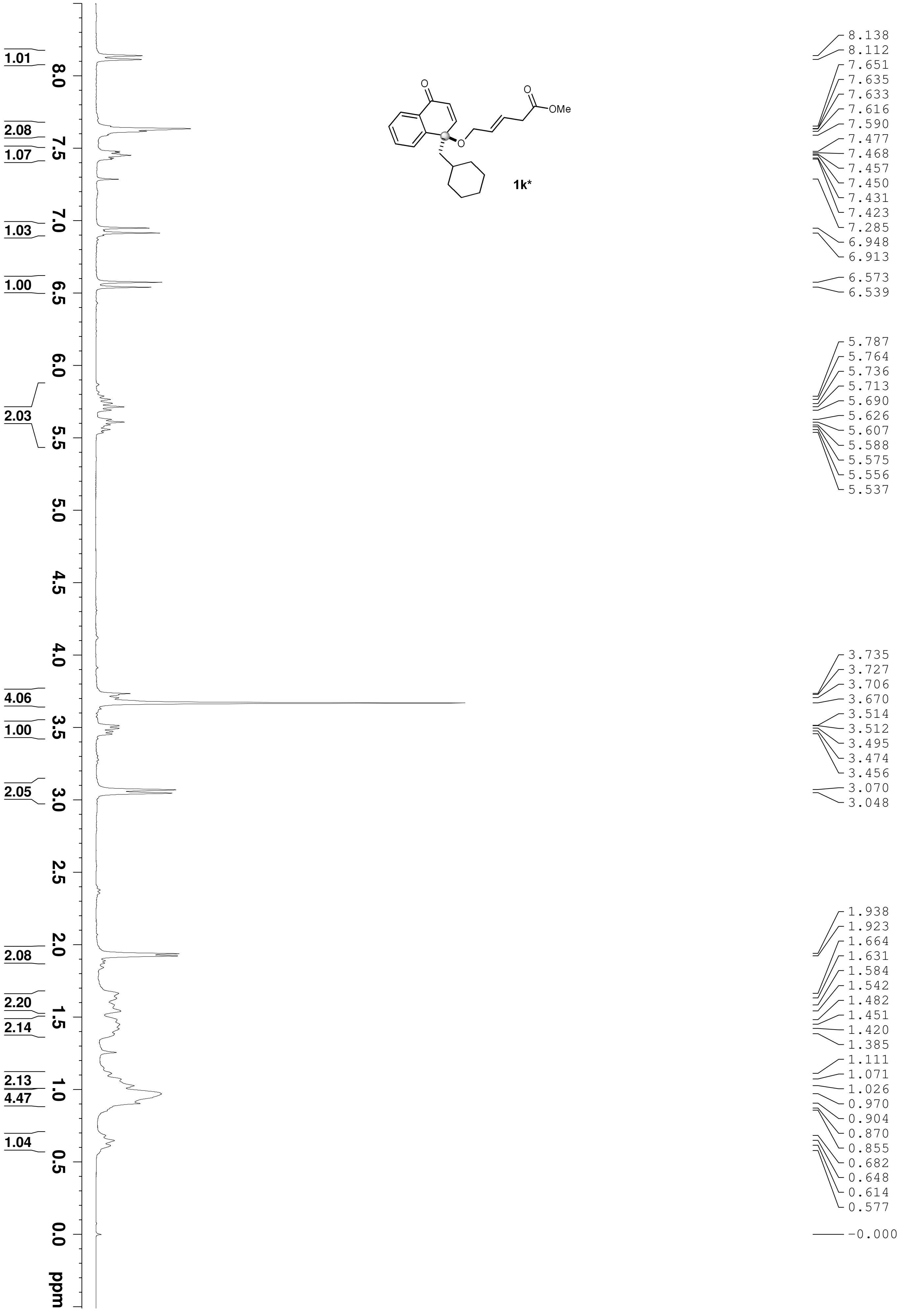

Supplementary Figure 47. ^{13}C NMR spectrum of compound **1l***

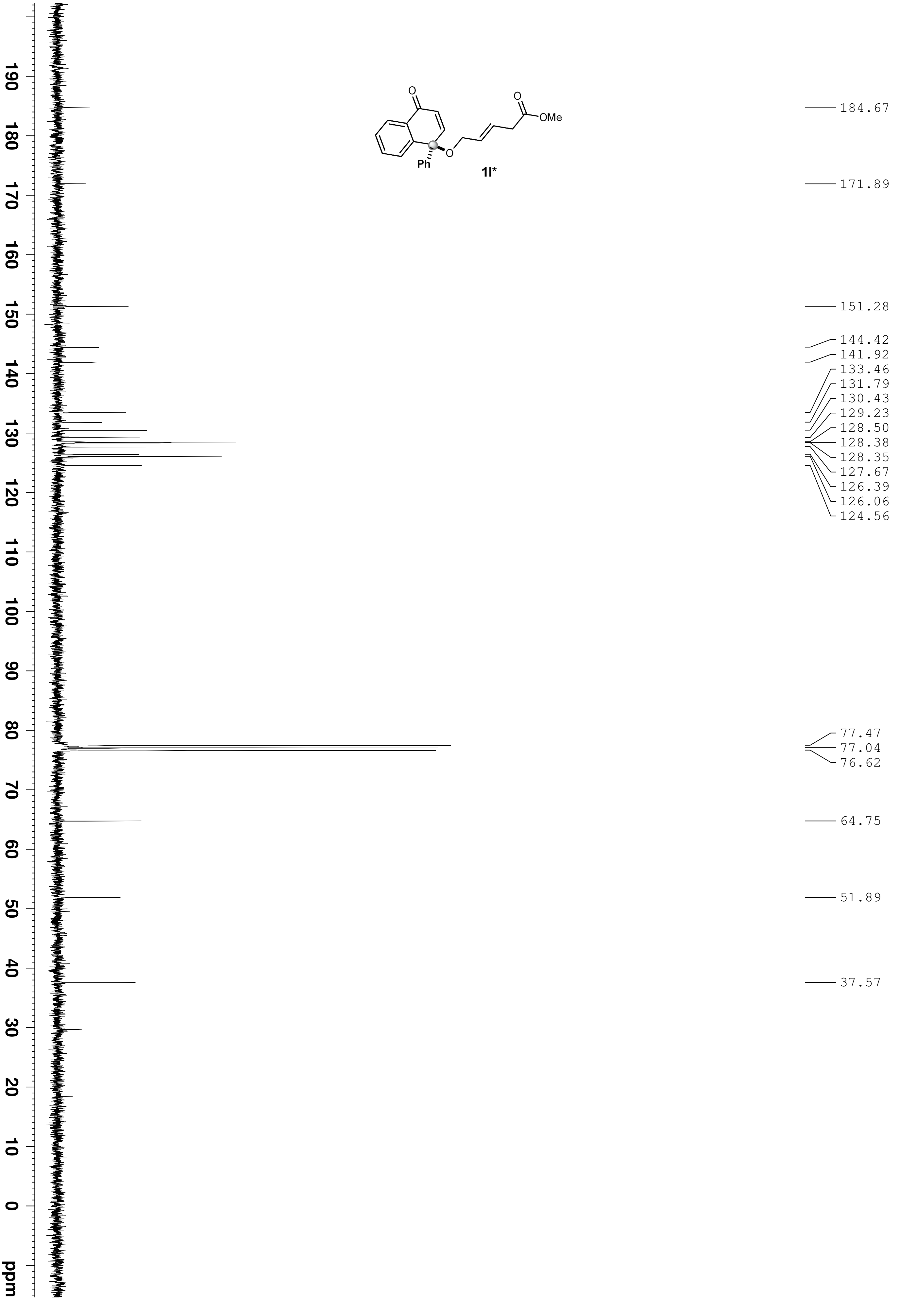

Supplementary Figure 48. ^{1}H NMR spectrum of compound **1l***

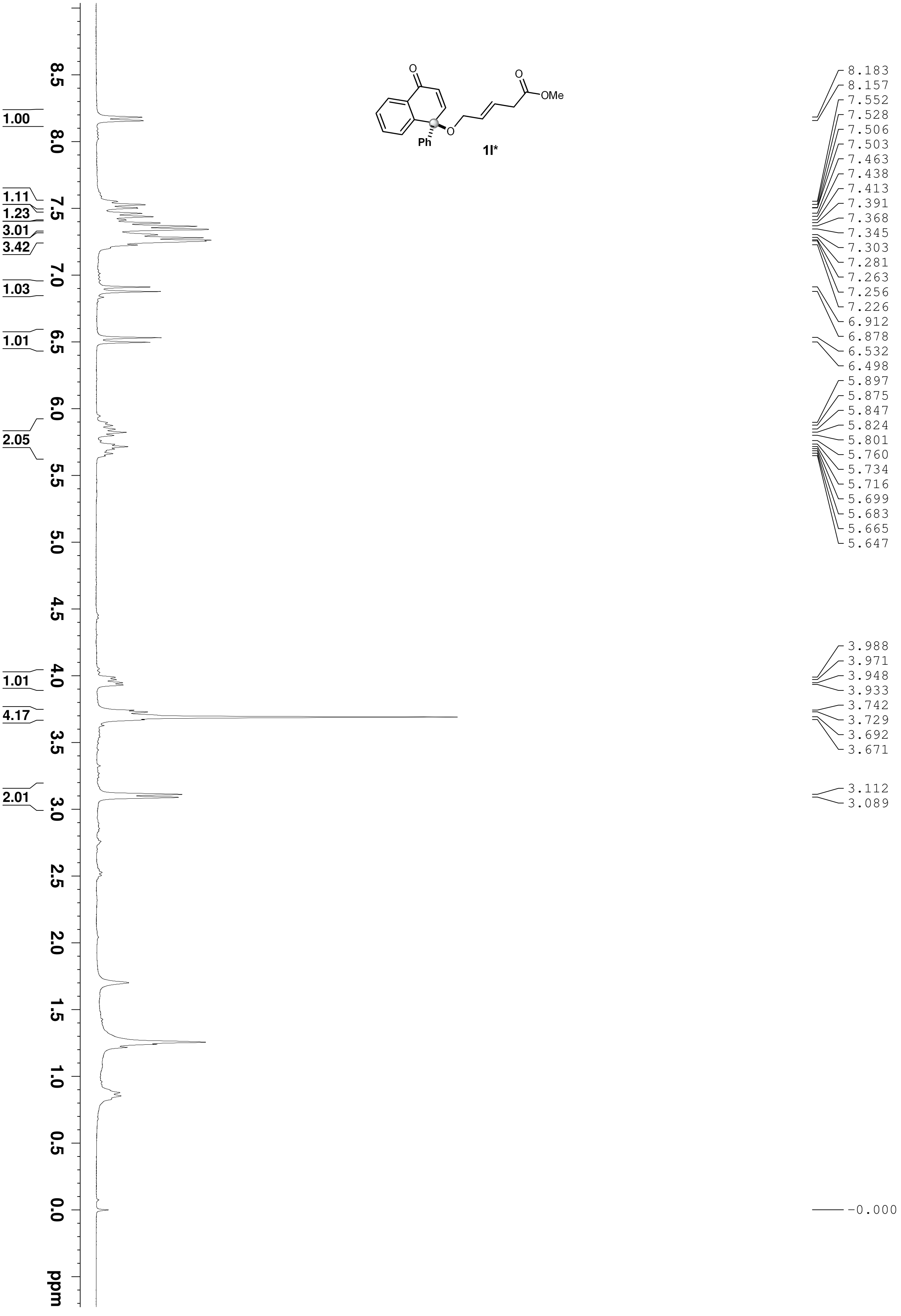

Supplementary Figure 49. ^{13}C NMR spectrum of compound **1m***

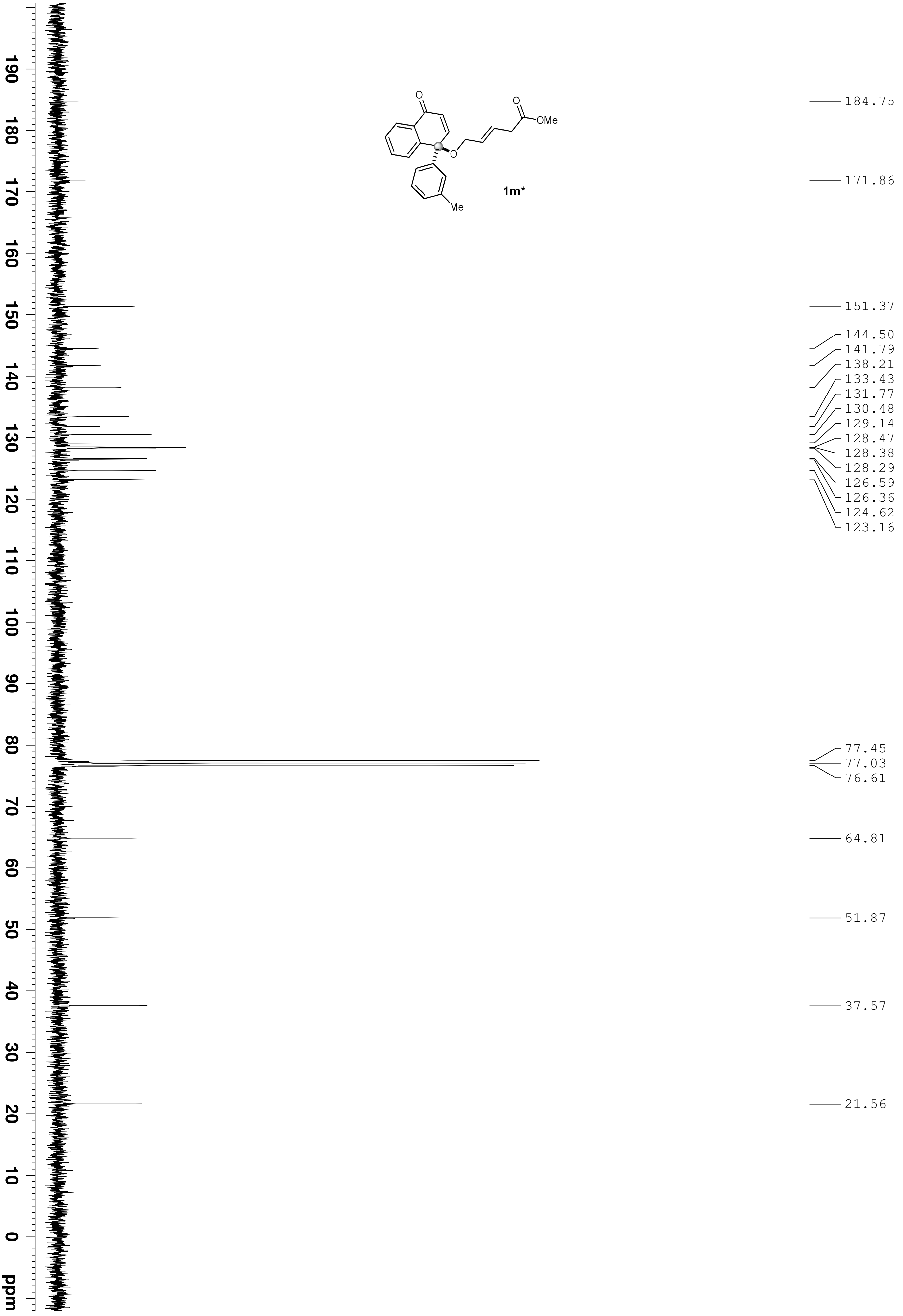

Supplementary Figure 50. ^{1}H NMR spectrum of compound **1m***

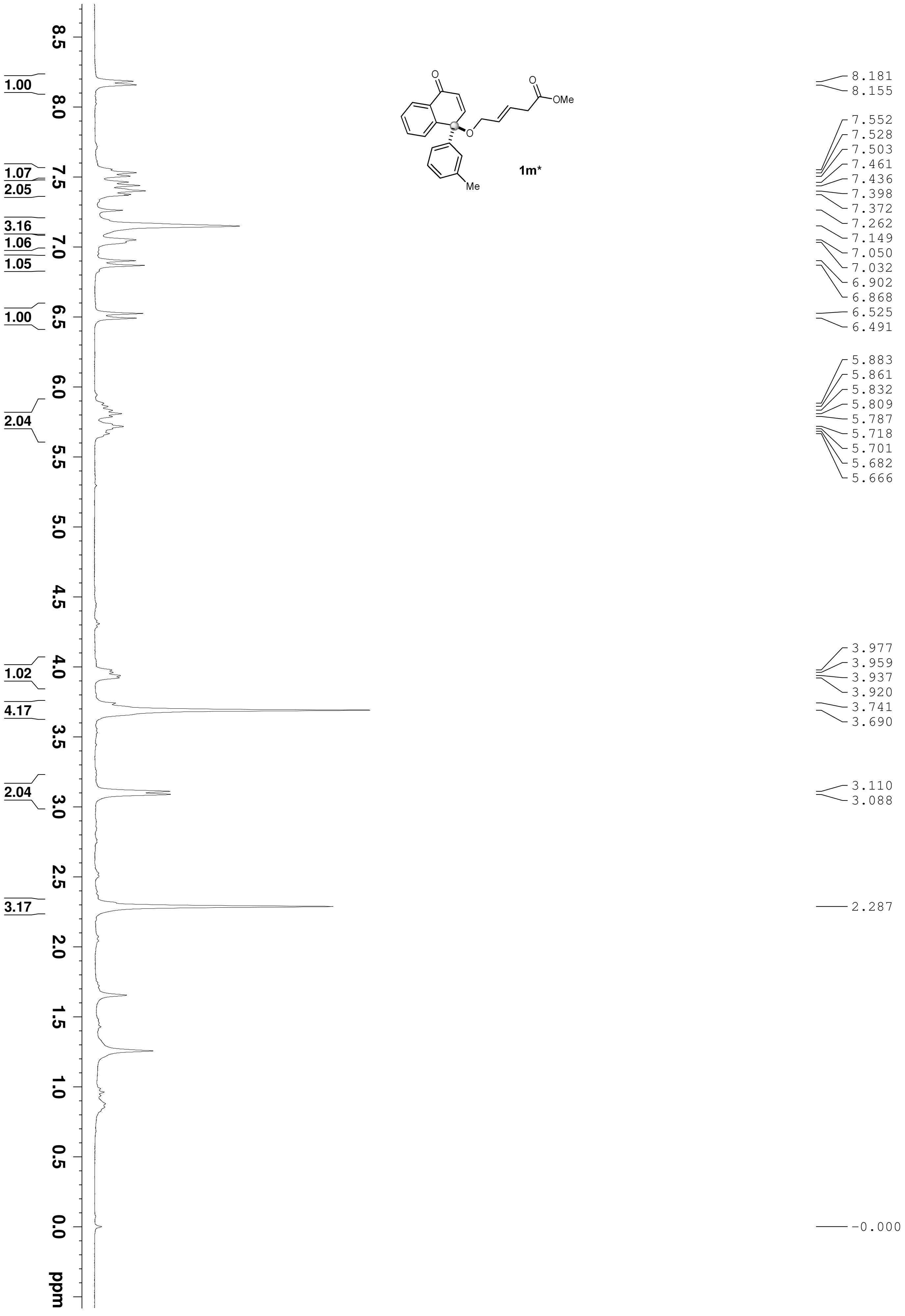

Supplementary Figure 51. ^{13}C NMR spectrum of compound **1n***

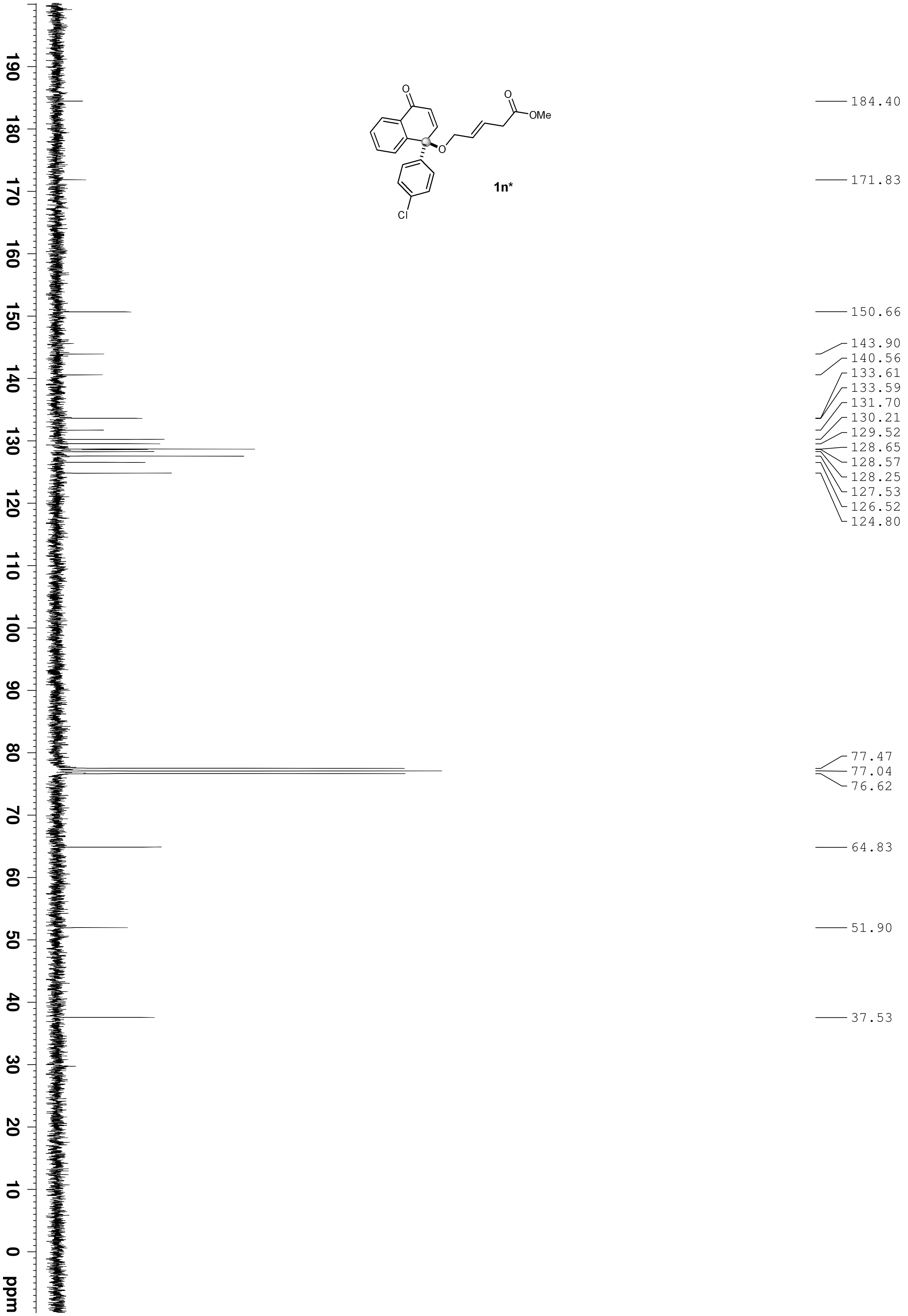

Supplementary Figure 52. ^{1}H NMR spectrum of compound **1n***

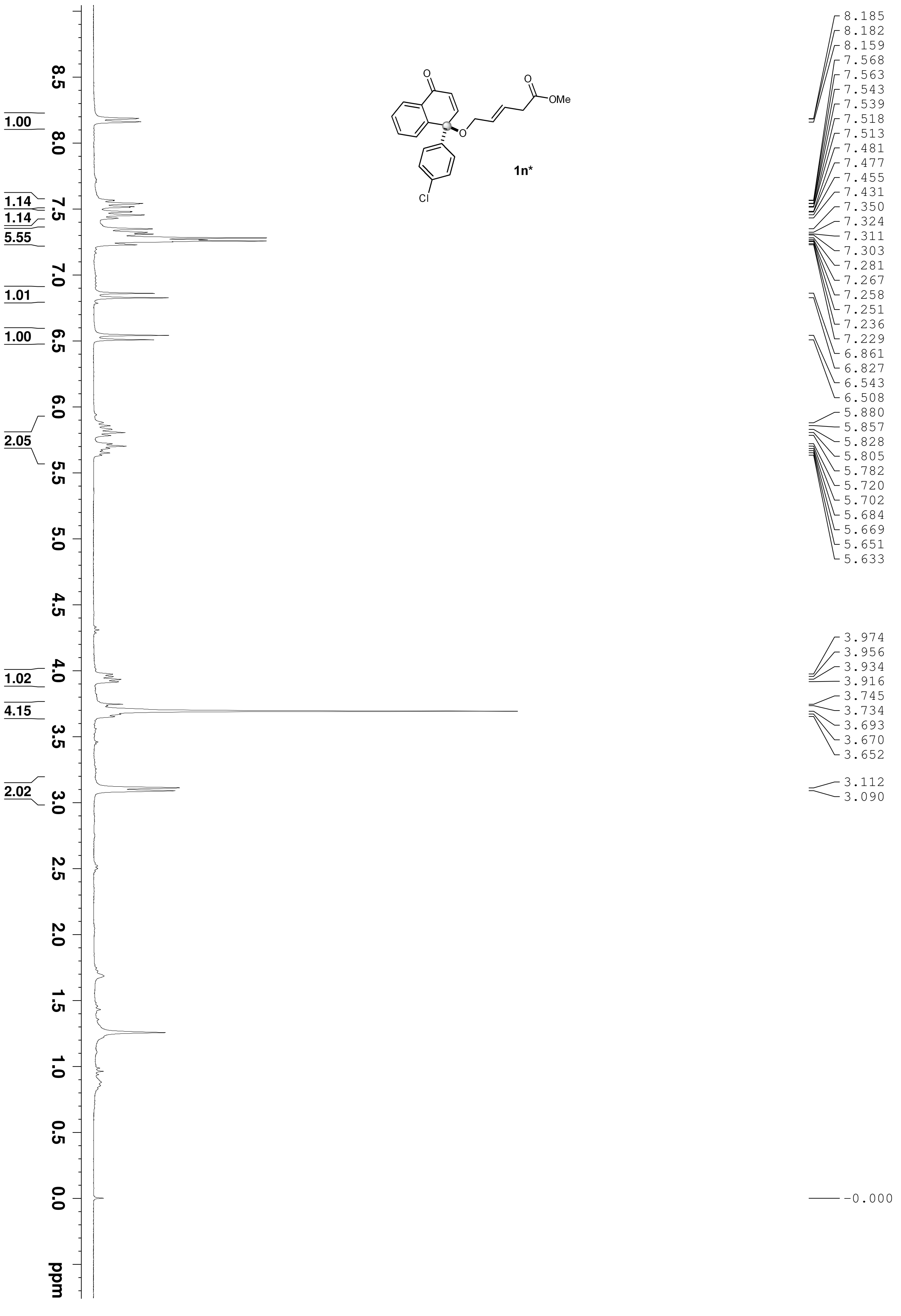

Supplementary Figure 53. ^{13}C NMR spectrum of compound **1o***

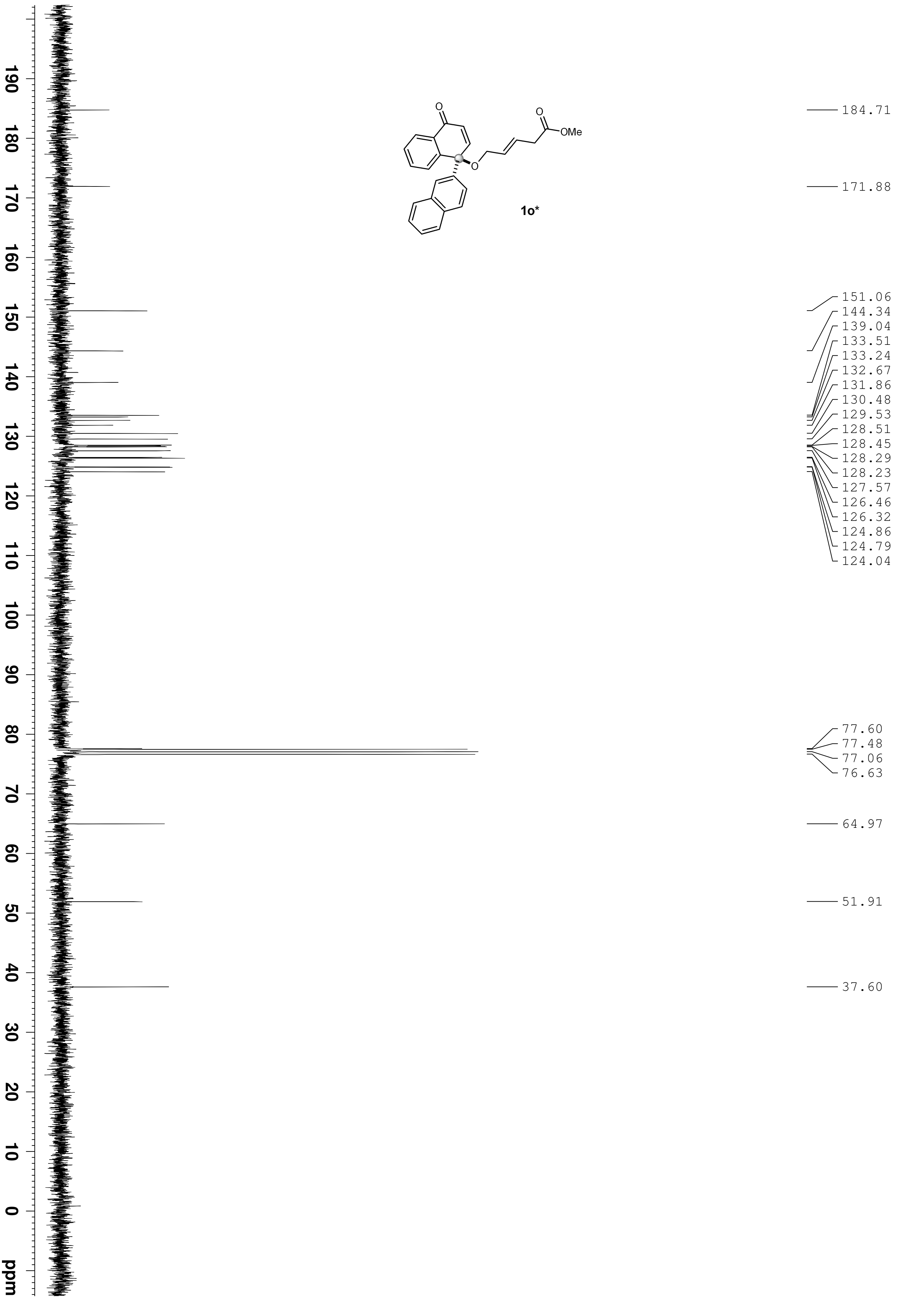

Supplementary Figure 54. ^{1}H NMR spectrum of compound **1o***

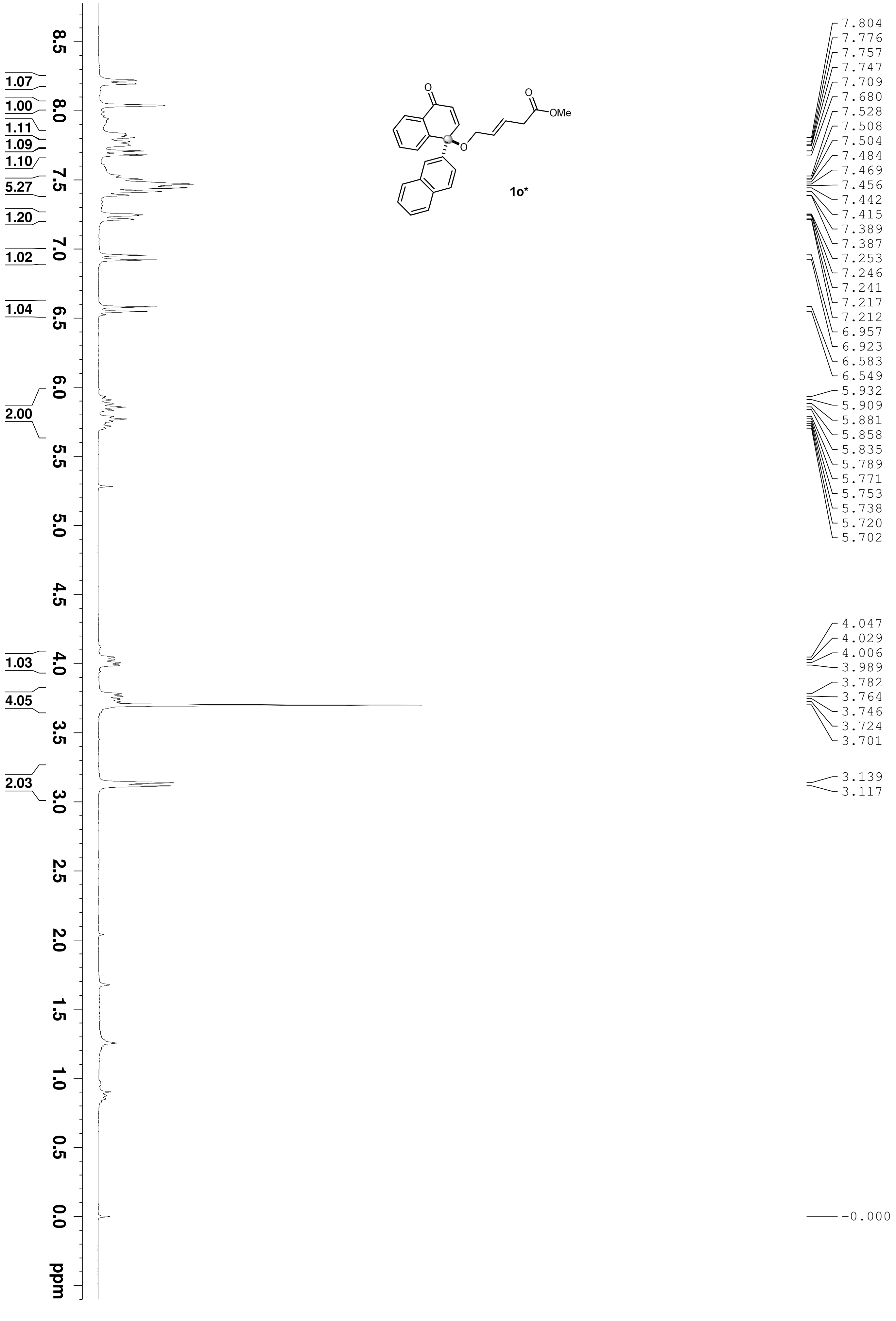

Supplementary Figure 55. ^{13}C NMR spectrum of compound **1p***

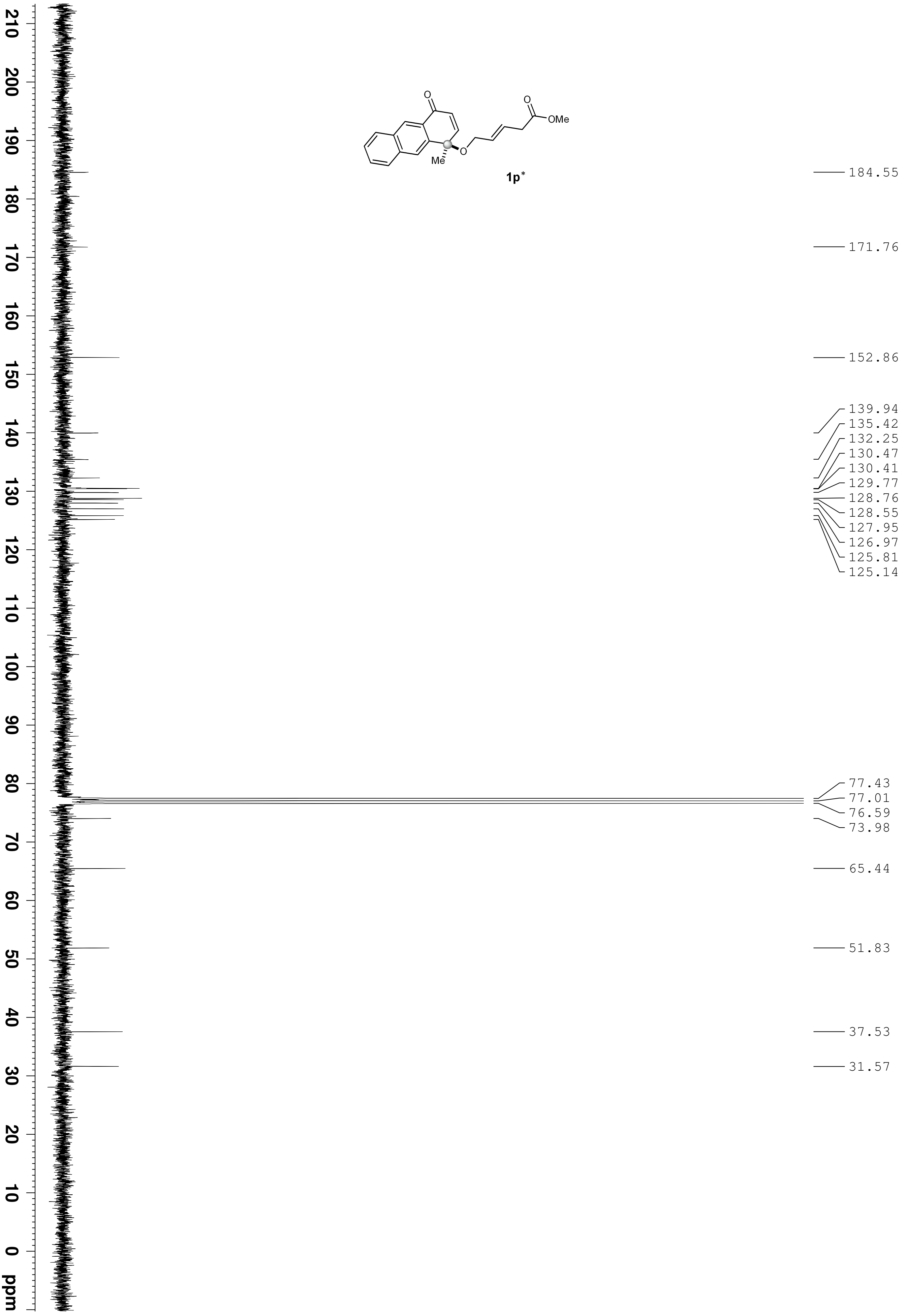

Supplementary Figure 56. ^{1}H NMR spectrum of compound **1p***

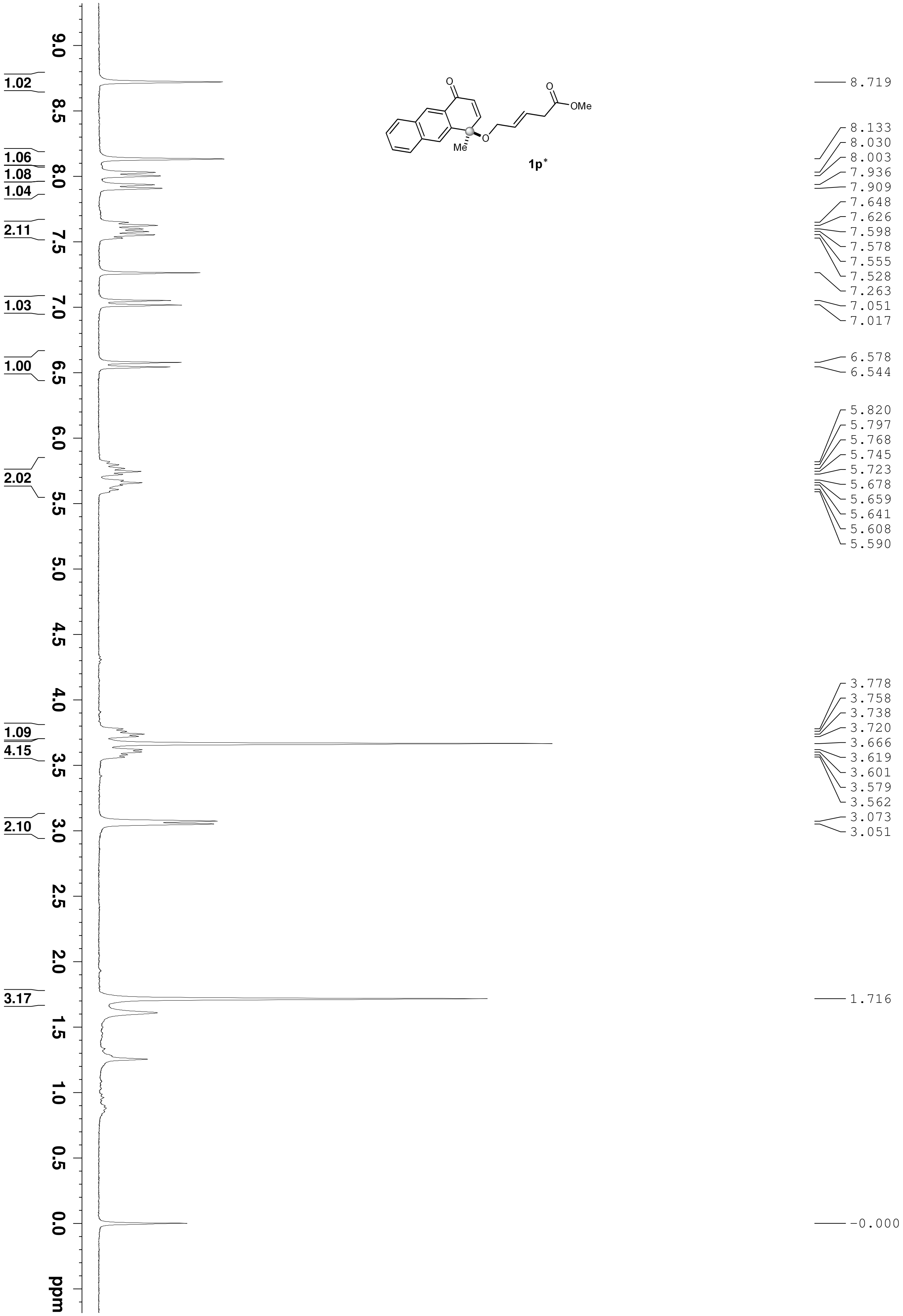

Supplementary Figure 57. ^{13}C NMR spectrum of compound **1q***

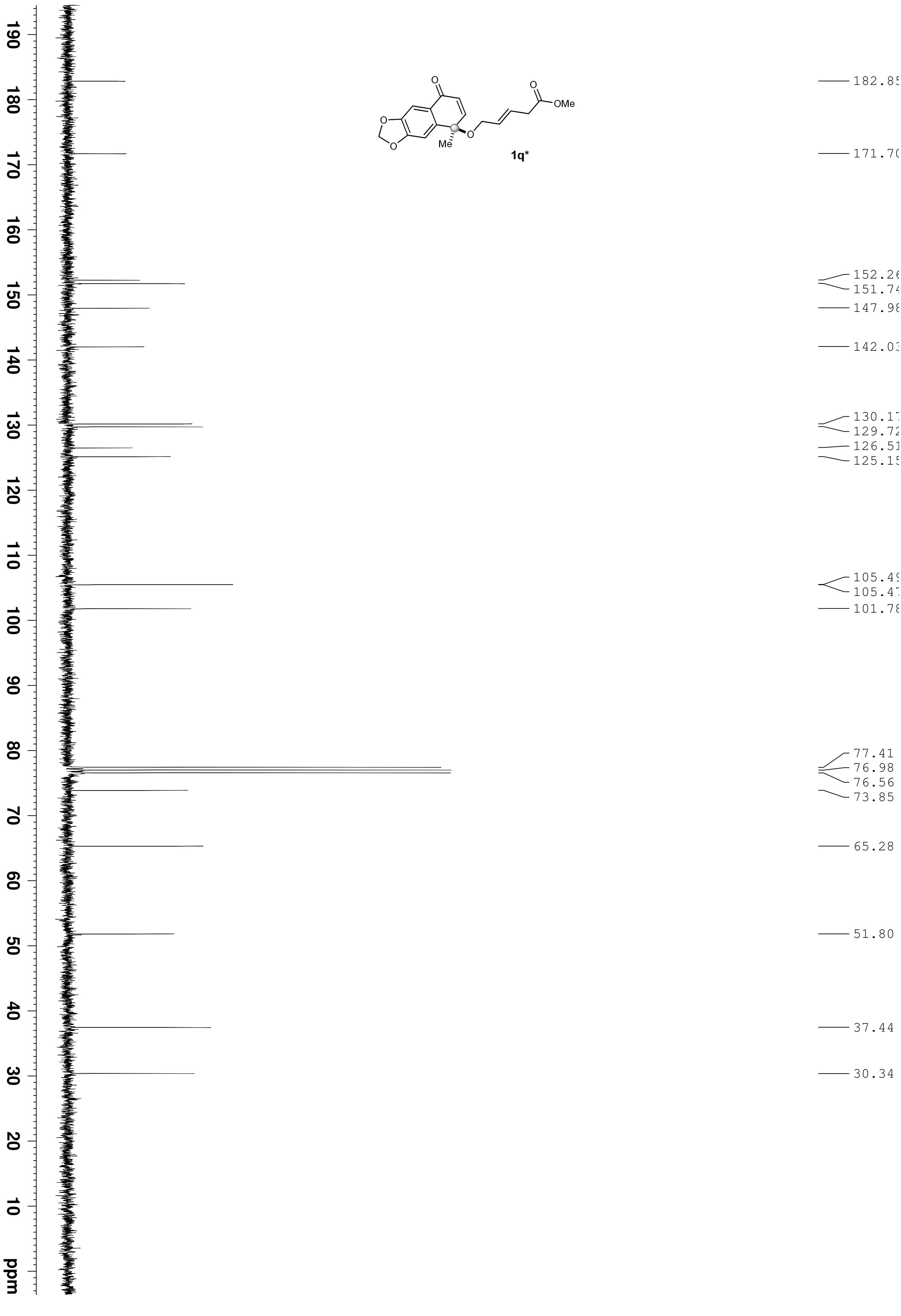

Supplementary Figure 58. ^{1}H NMR spectrum of compound **1q***

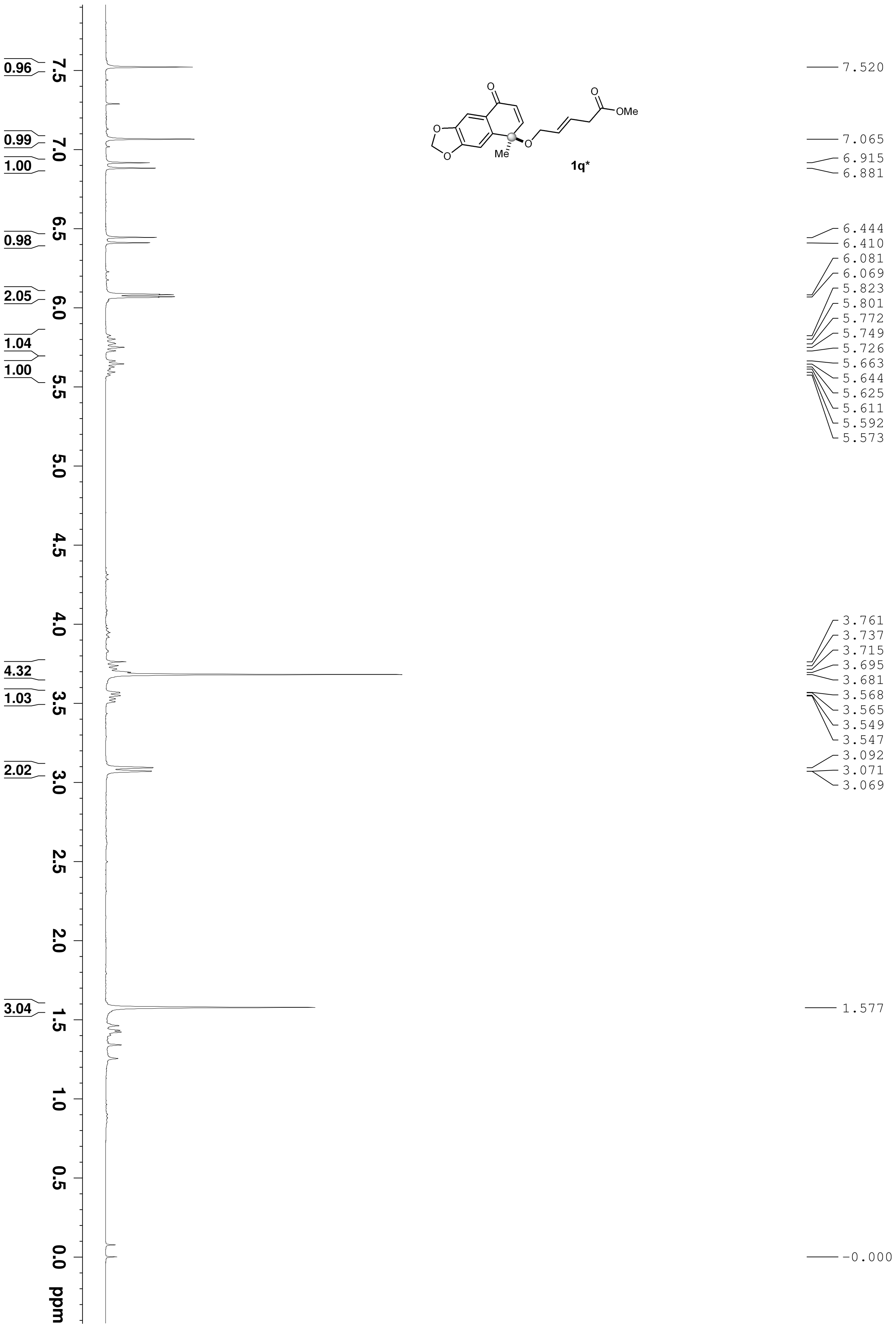

Supplementary Figure 59. ^{13}C NMR spectrum of compound **1r***

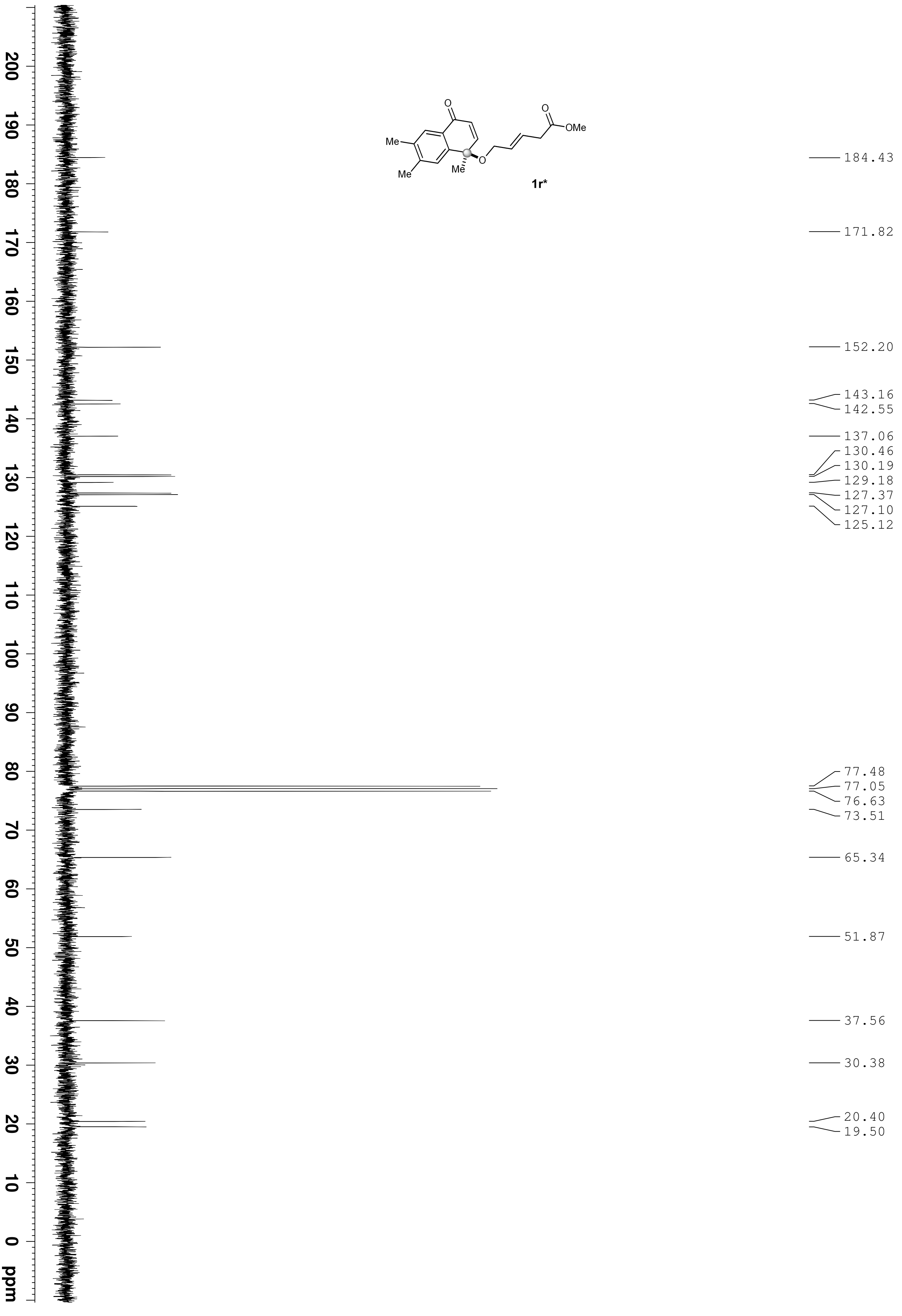

Supplementary Figure 60. ^{1}H NMR spectrum of compound **1r***

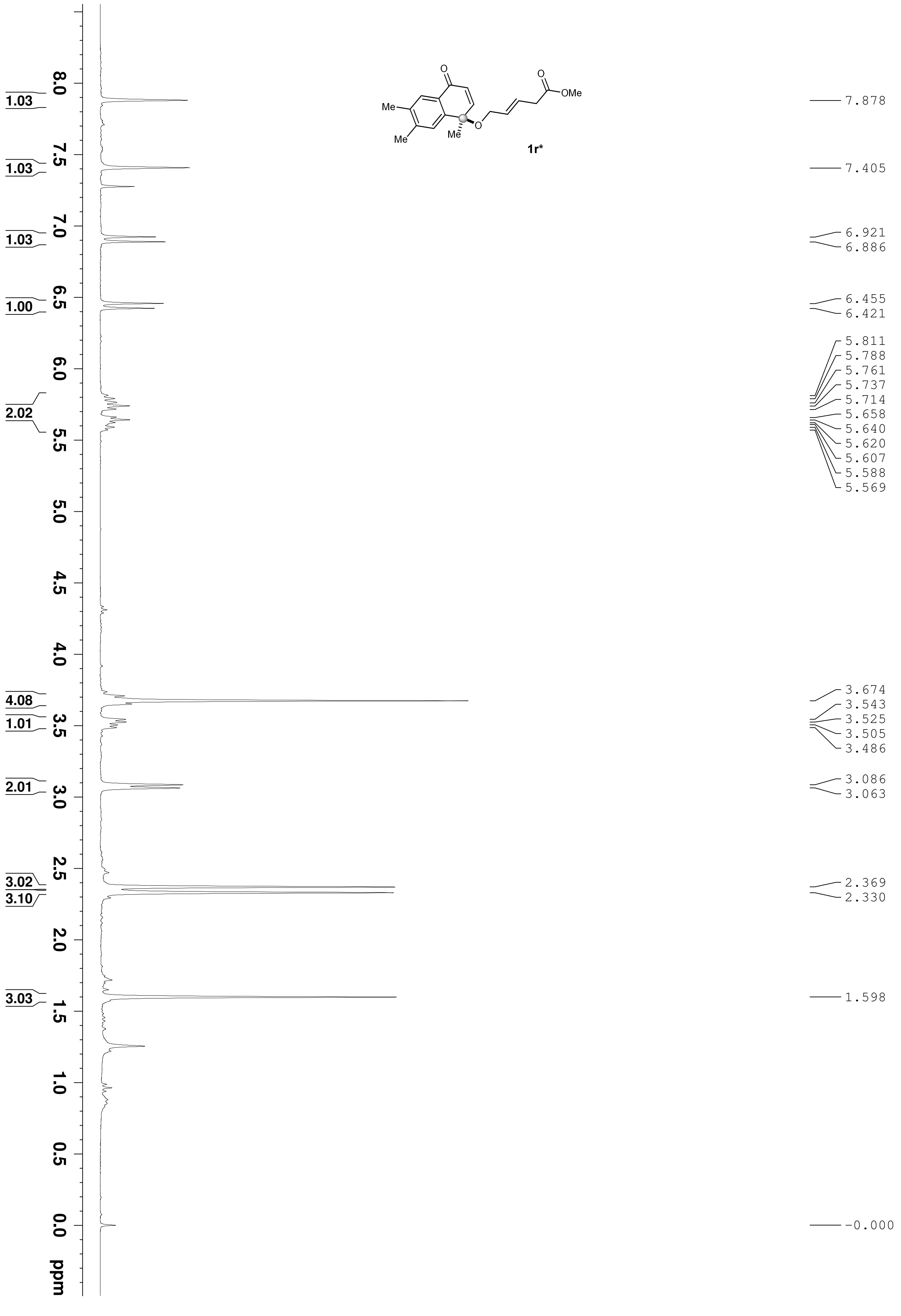

Supplementary Figure 61. ^{13}C NMR spectrum of compound **1s***

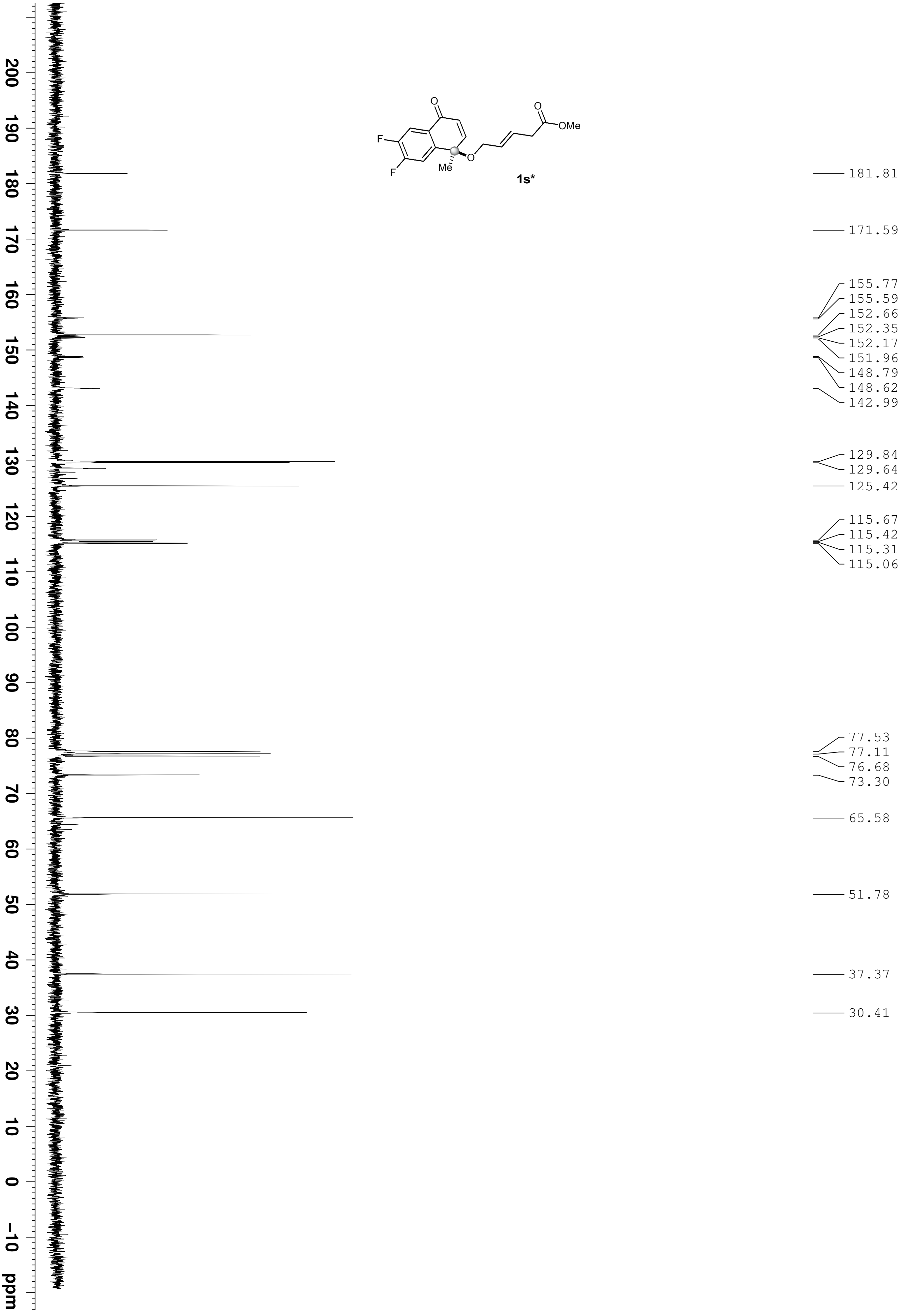

Supplementary Figure 62. ^{1}H NMR spectrum of compound **1s***

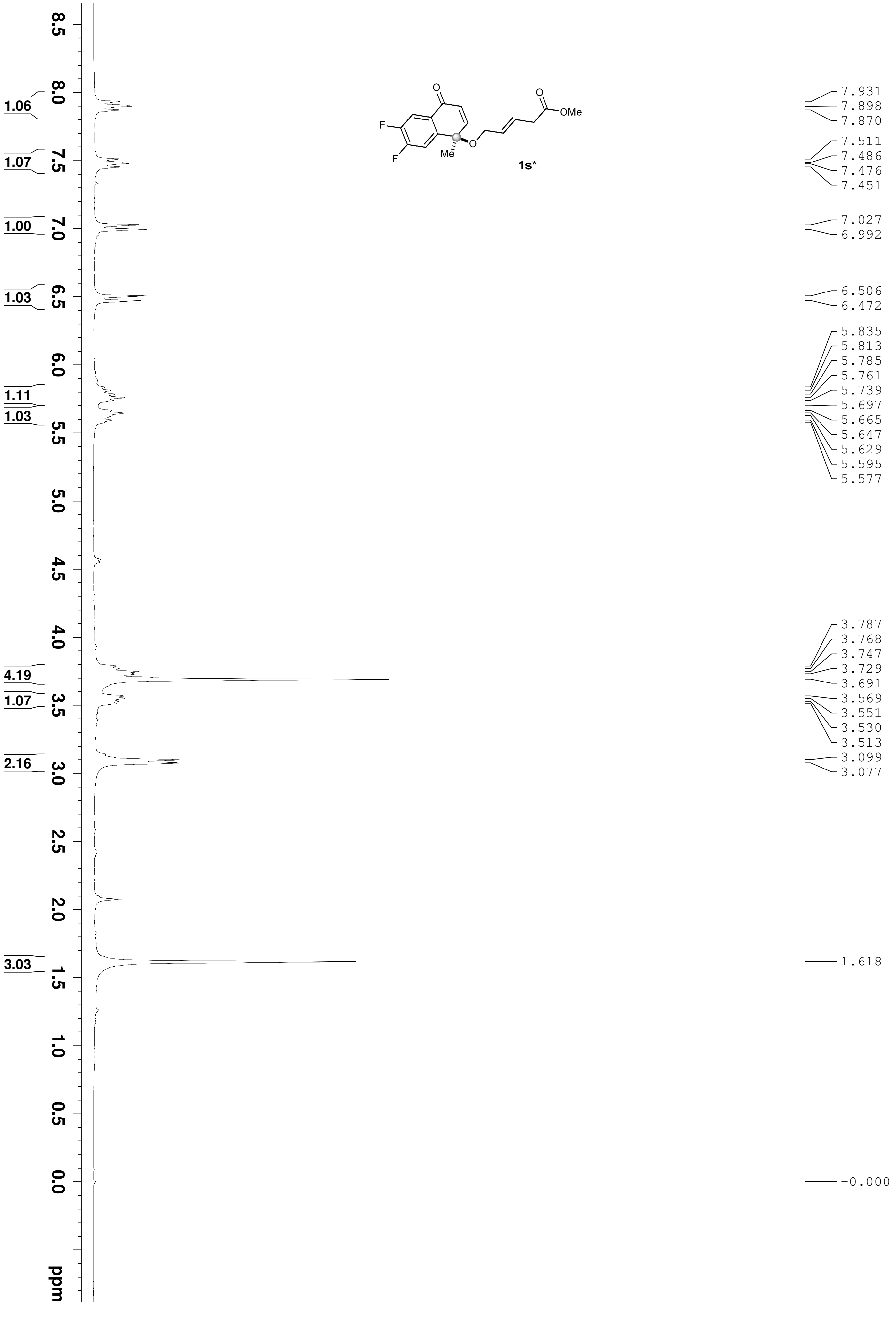

Supplementary Figure 63. ^{13}C NMR spectrum of compound **1t***

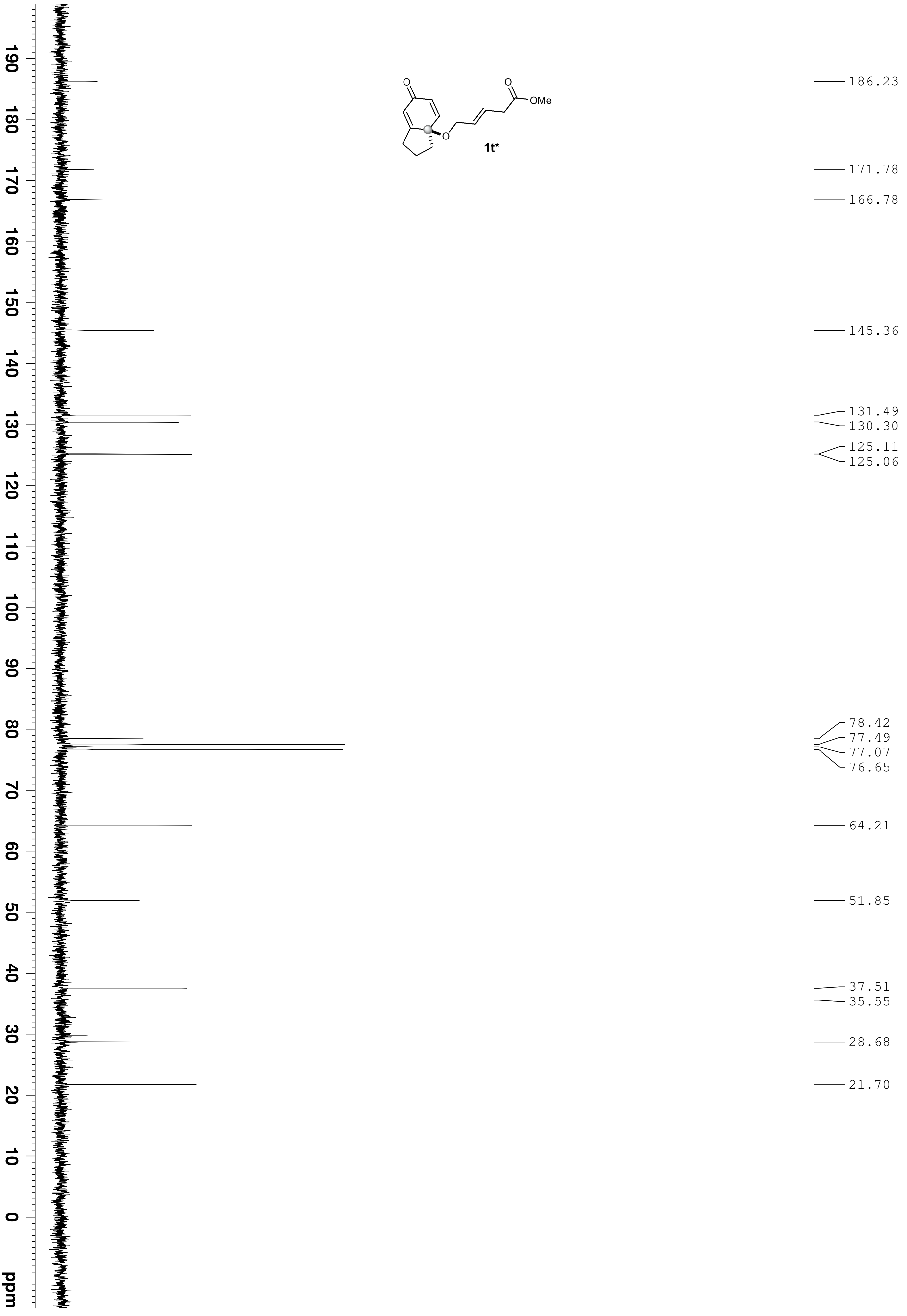

Supplementary Figure 64. ^{1}H NMR spectrum of compound **1t***

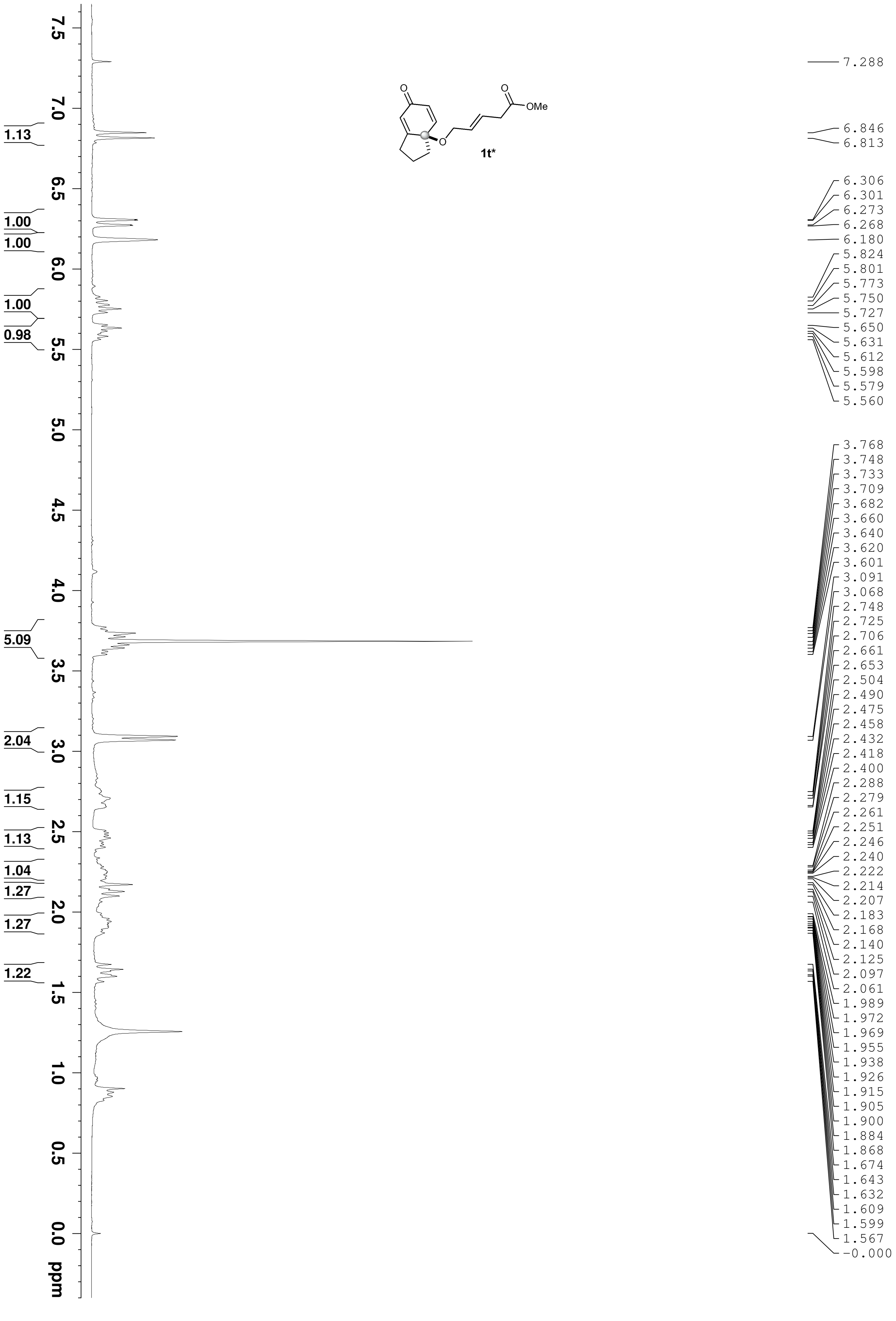

Supplementary Figure 65. ^{13}C NMR spectrum of compound **2a**

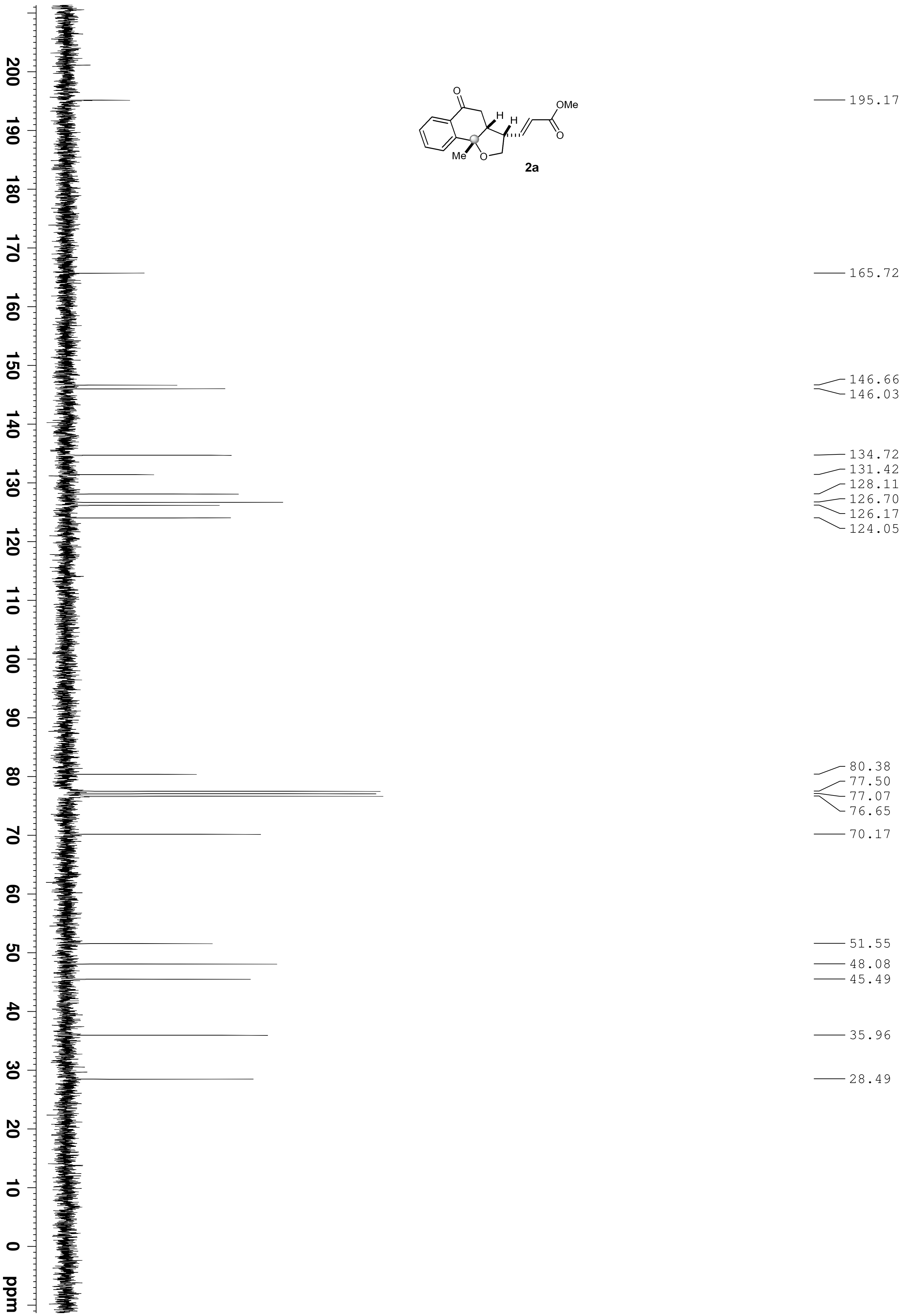

Supplementary Figure 66. ^{1}H NMR spectrum of compound **2a**

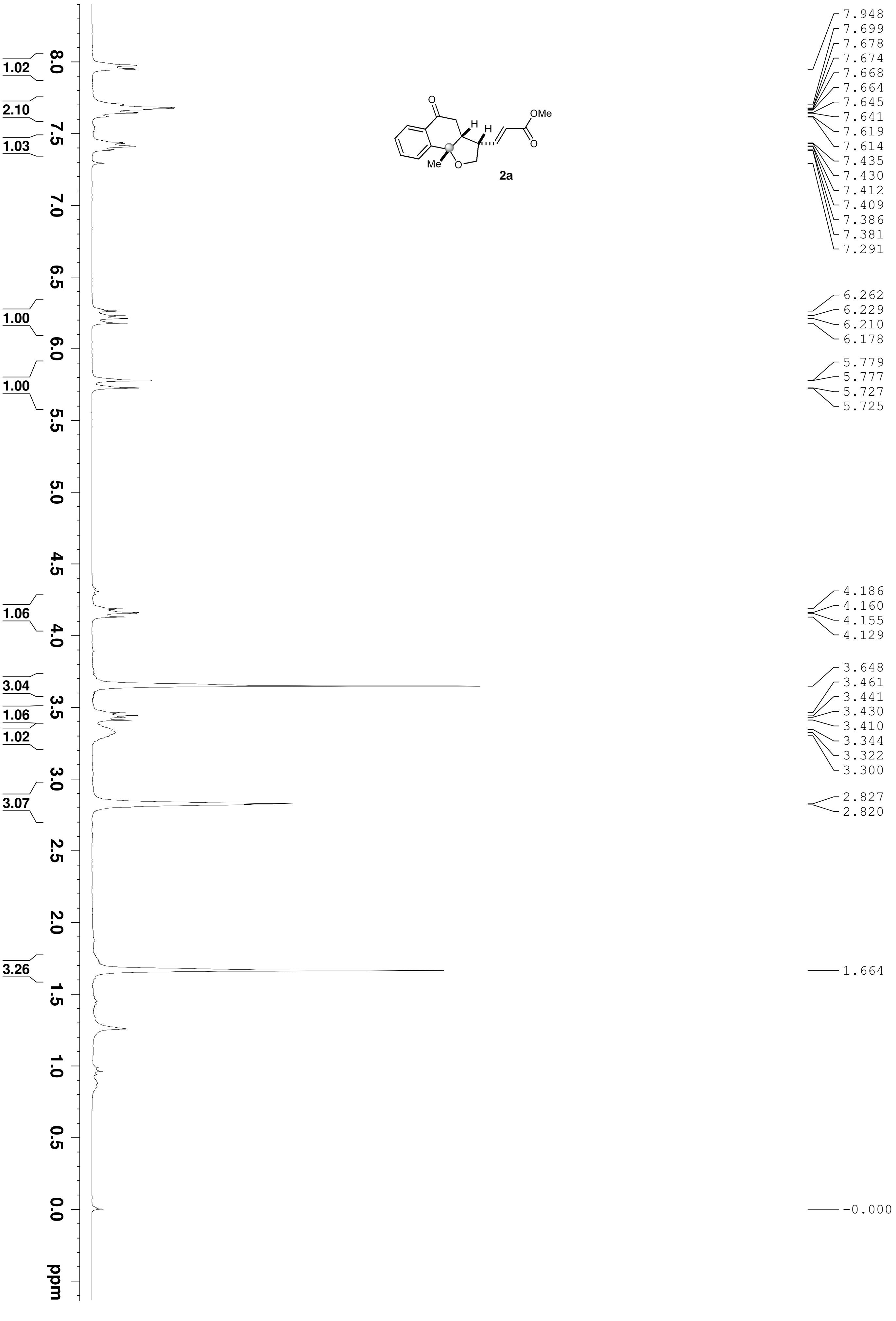

Supplementary Figure 67. ^{13}C NMR spectrum of compound **2a'**

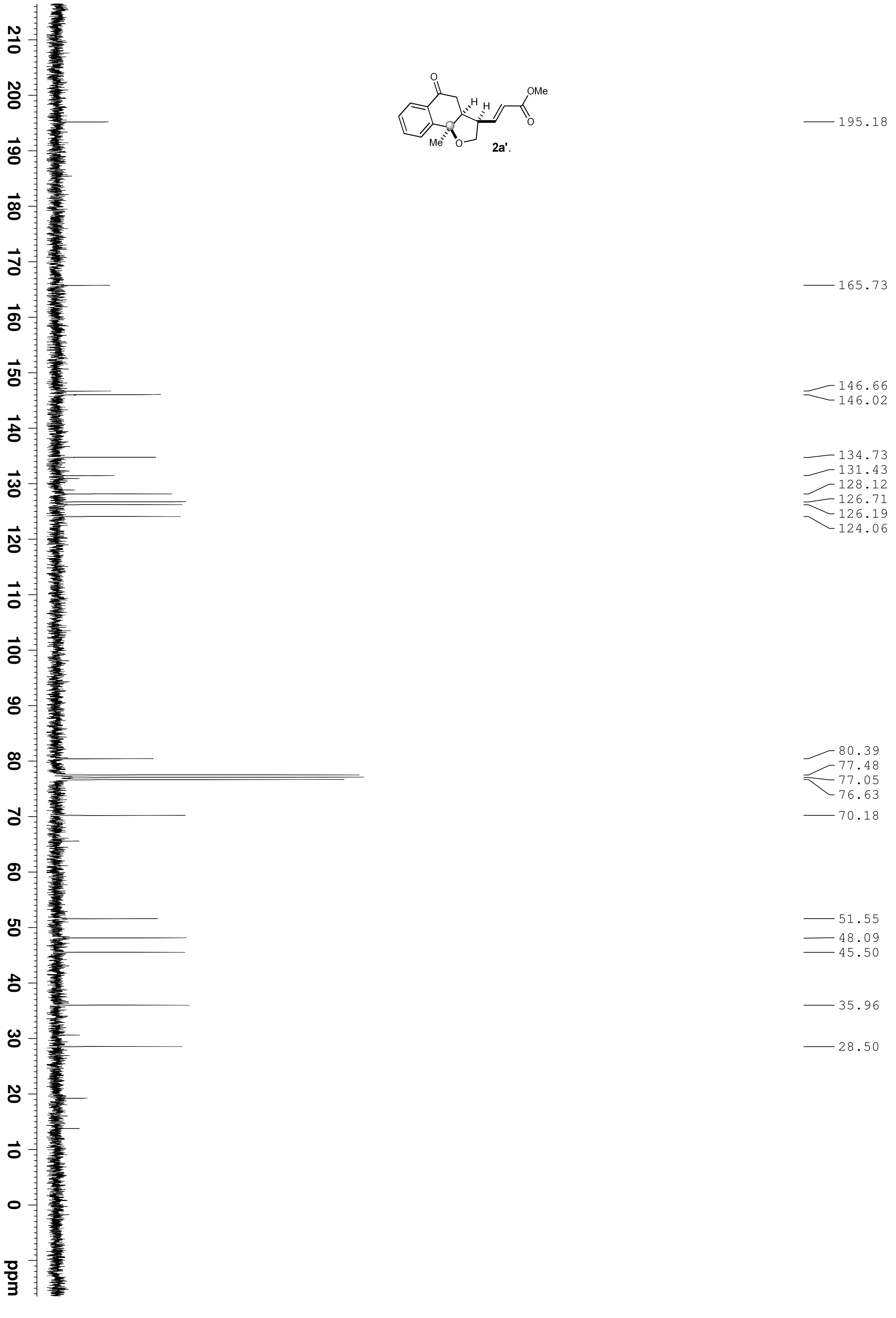

Supplementary Figure 68. ^{1}H NMR spectrum of compound **2a'**

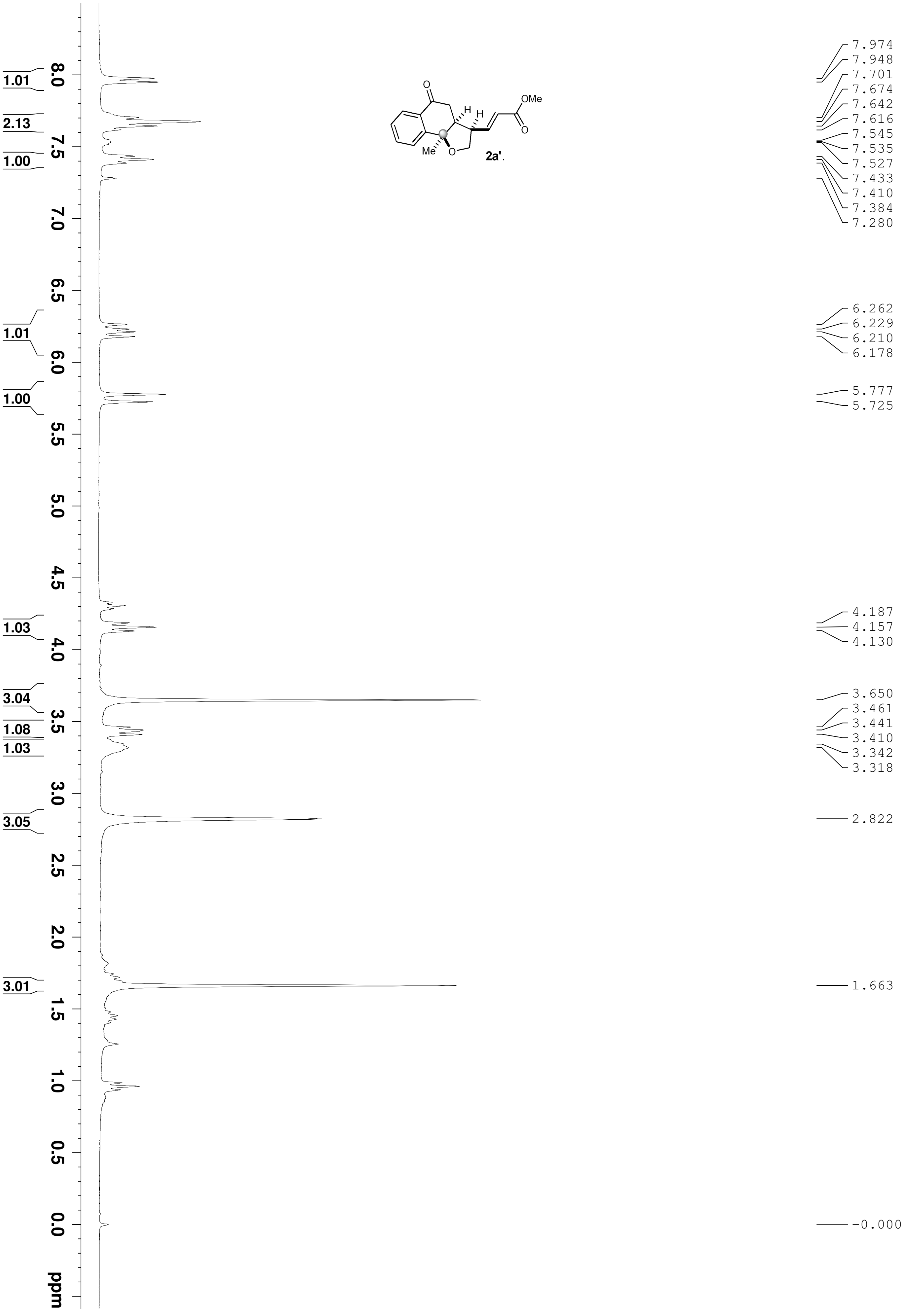

Supplementary Figure 69. ^{13}C NMR spectrum of compound **2b**

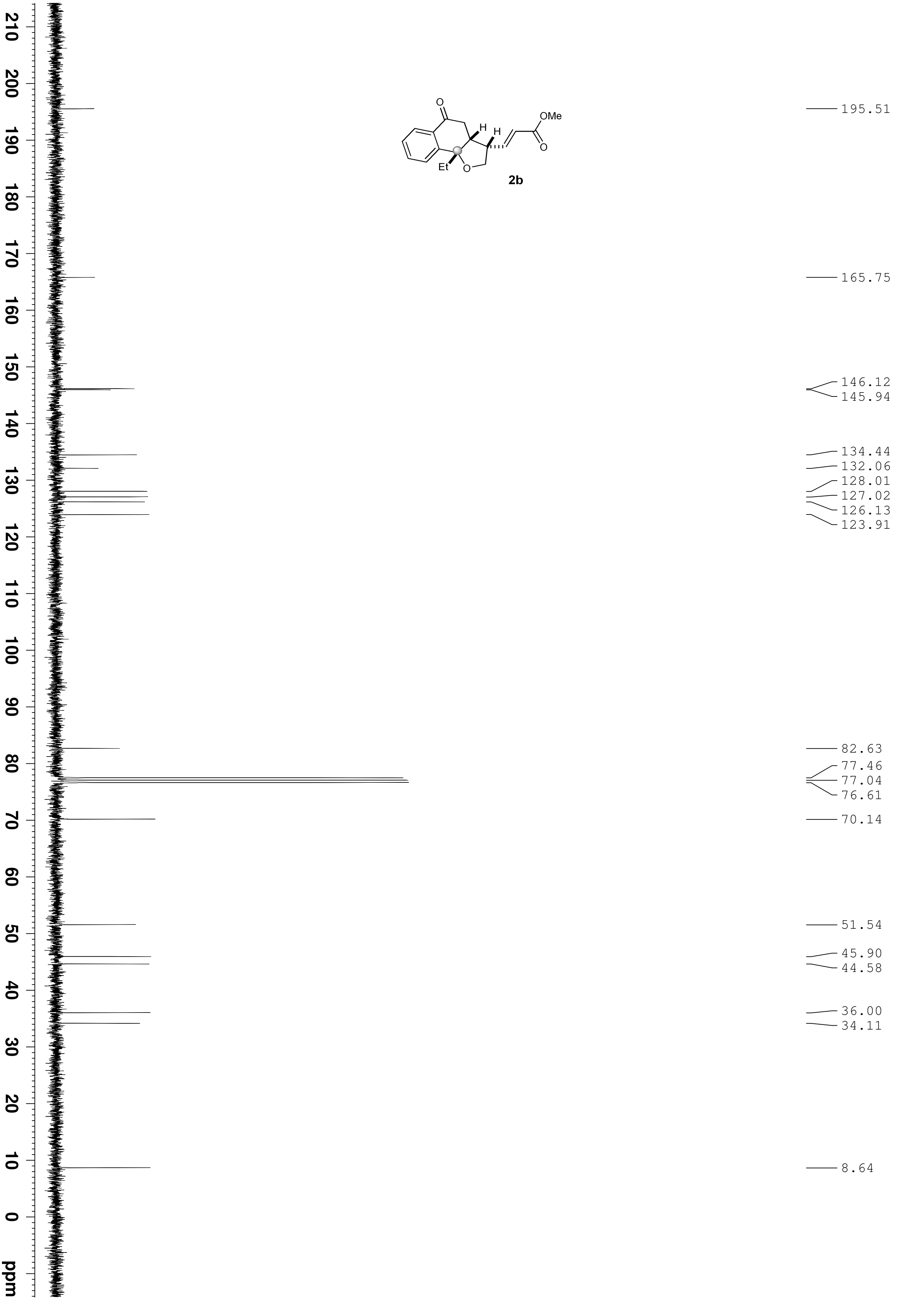

Supplementary Figure 70. ^{1}H NMR spectrum of compound **2b**

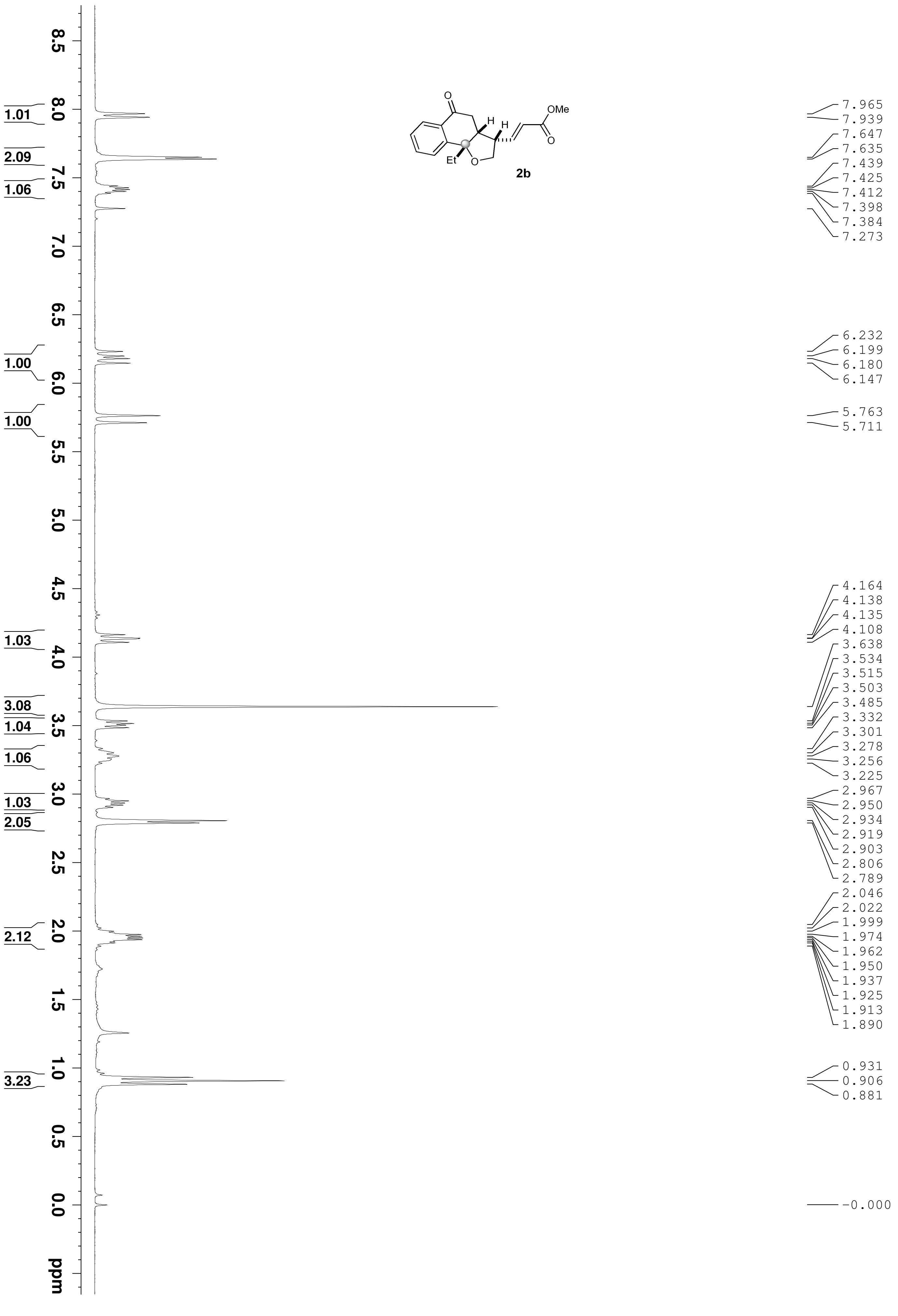

Supplementary Figure 71. ^{13}C NMR spectrum of compound **2c**

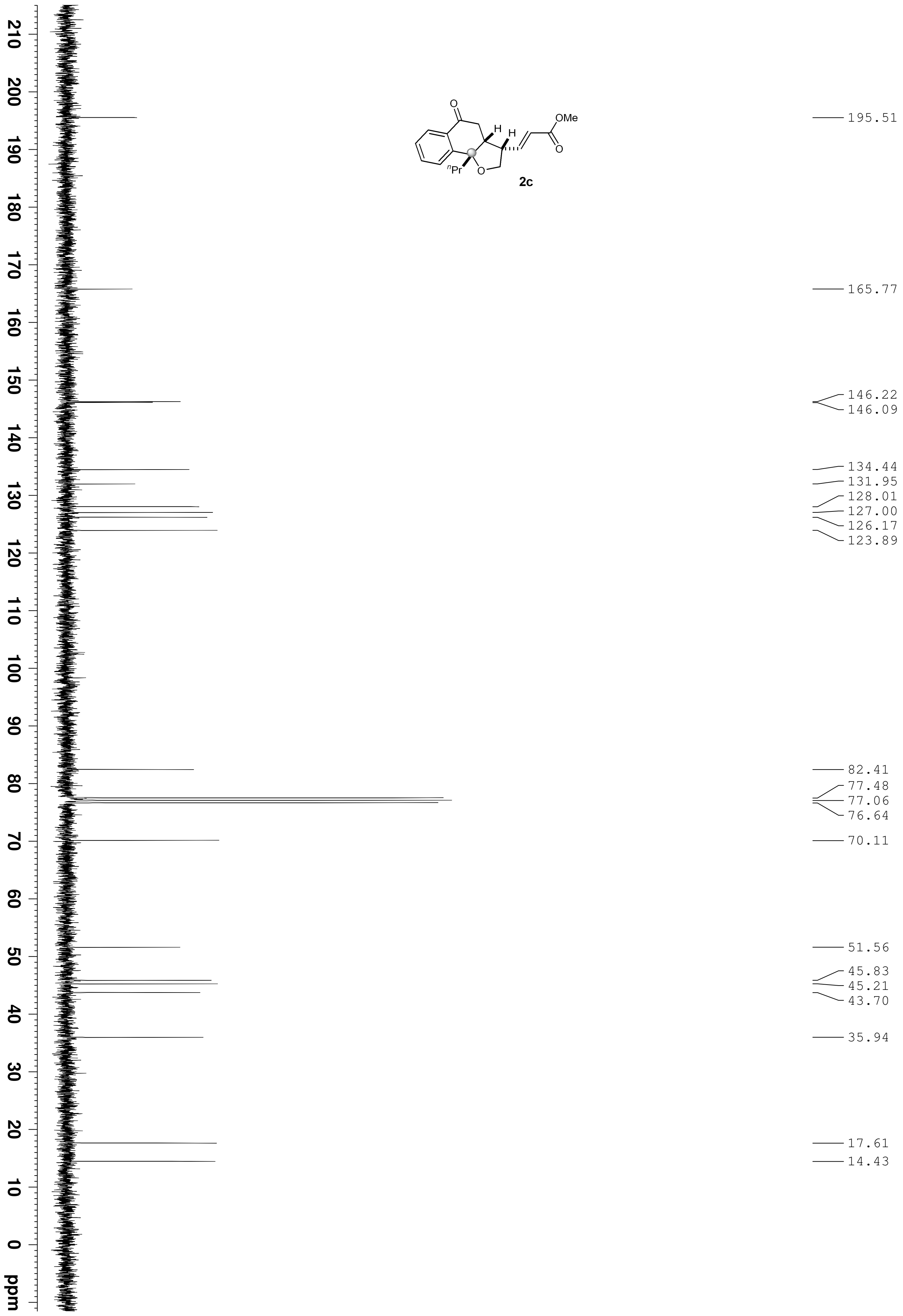

Supplementary Figure 72. ^{1}H NMR spectrum of compound **2c**

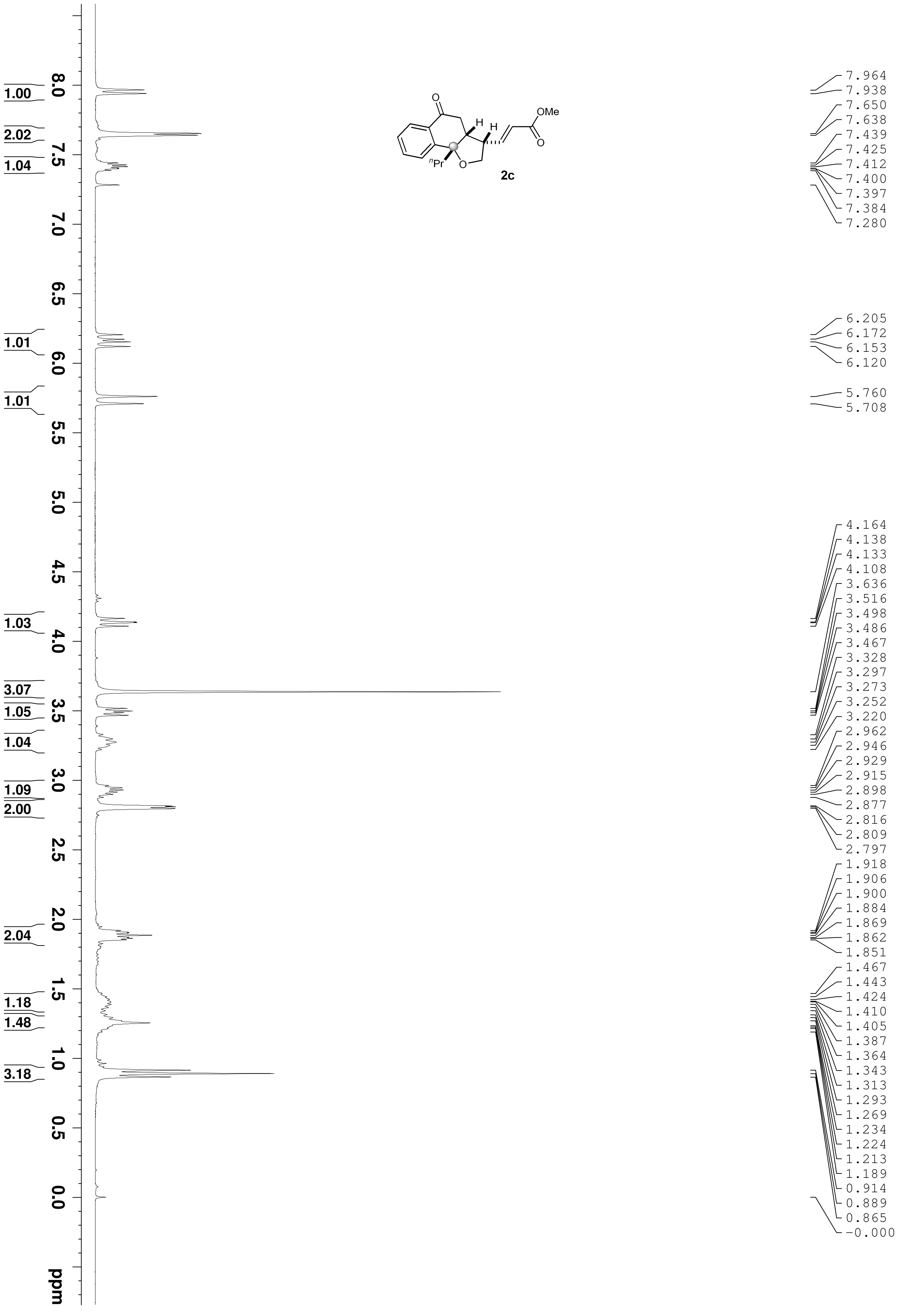

Supplementary Figure 73. ^{13}C NMR spectrum of compound **2d**

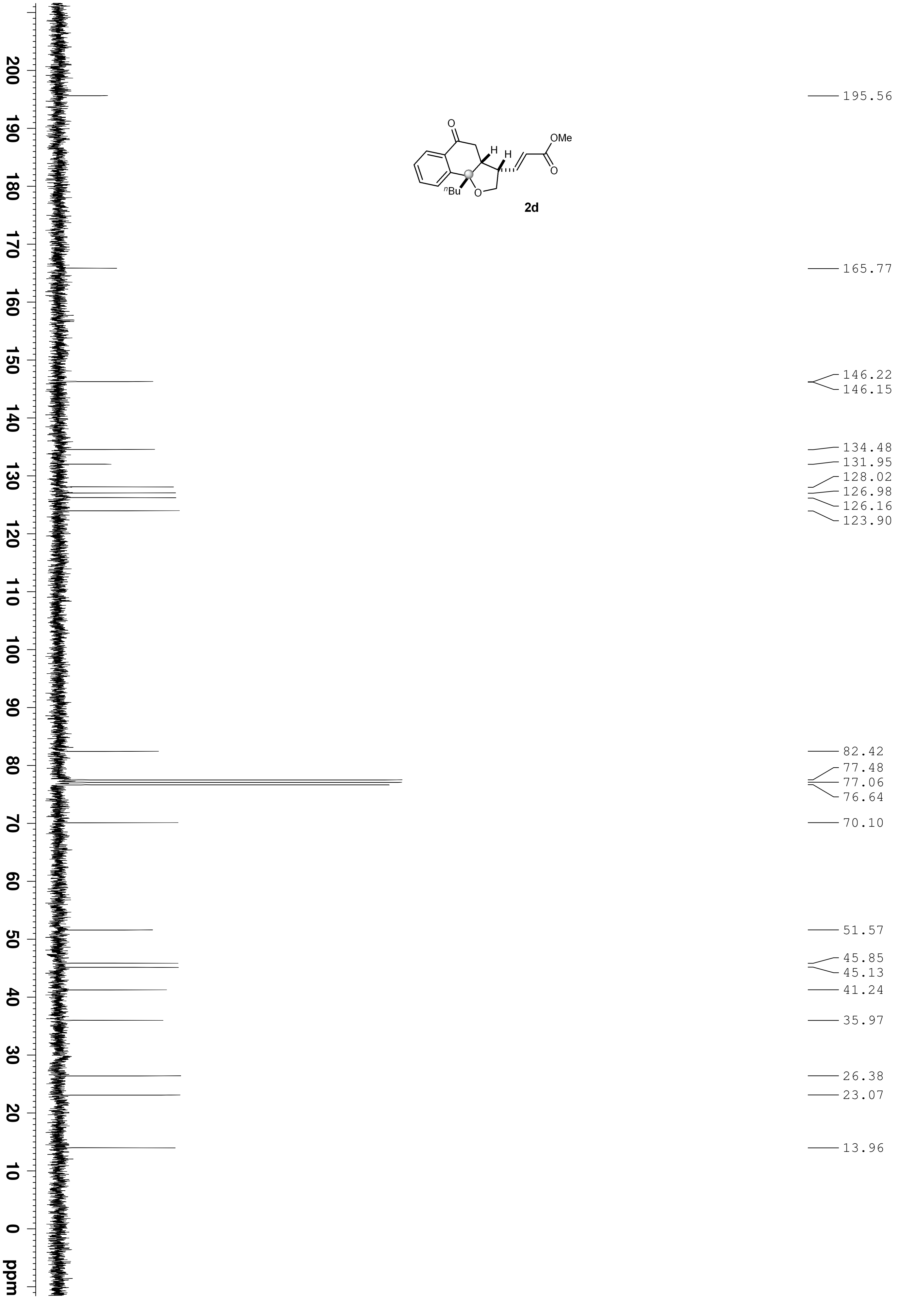

Supplementary Figure 74. ^{1}H NMR spectrum of compound **2d**

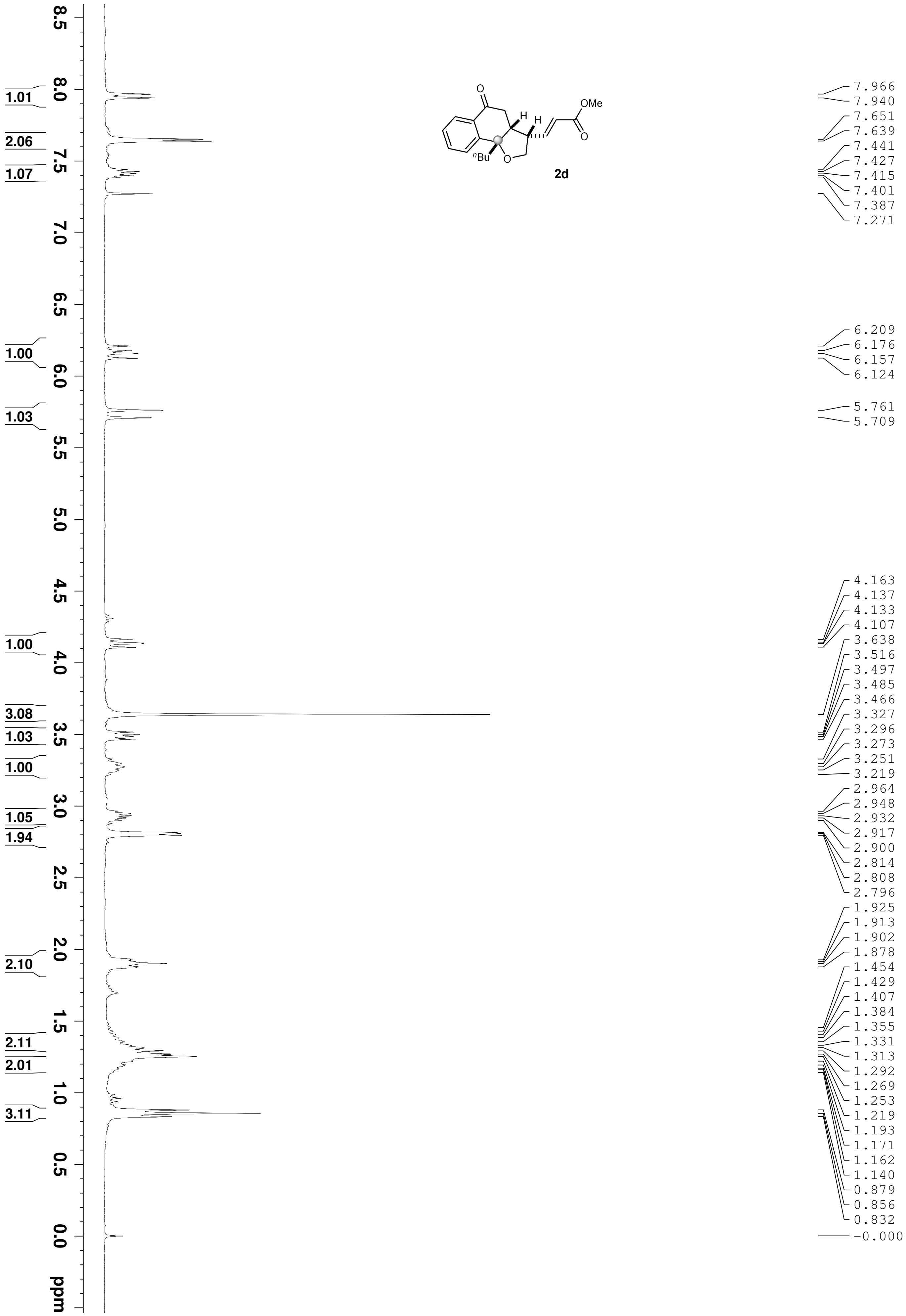

Supplementary Figure 75. ^{13}C NMR spectrum of compound **2e**

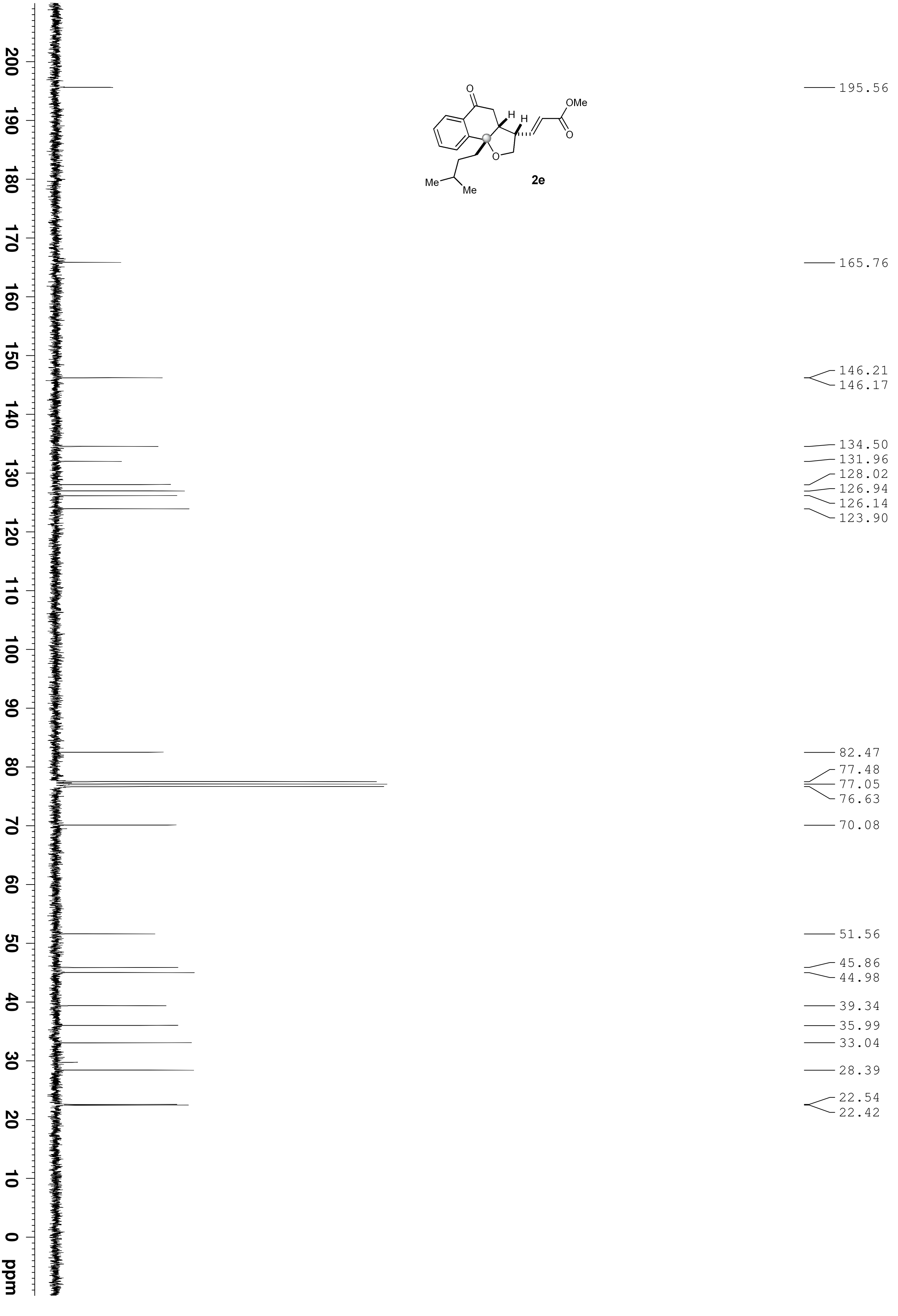

Supplementary Figure 76. ^{1}H NMR spectrum of compound **2e**

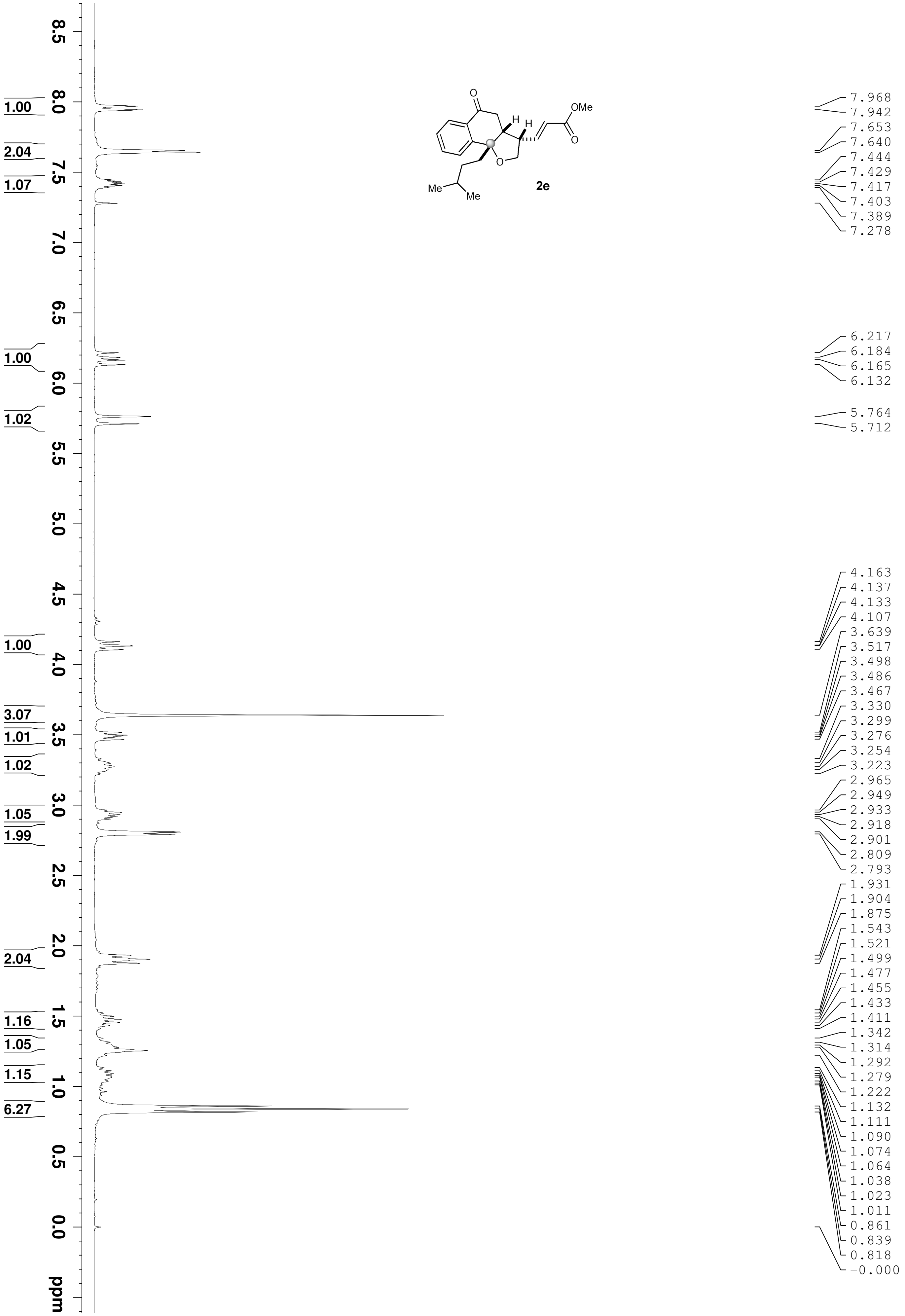

Supplementary Figure 77. ^{13}C NMR spectrum of compound **2f**

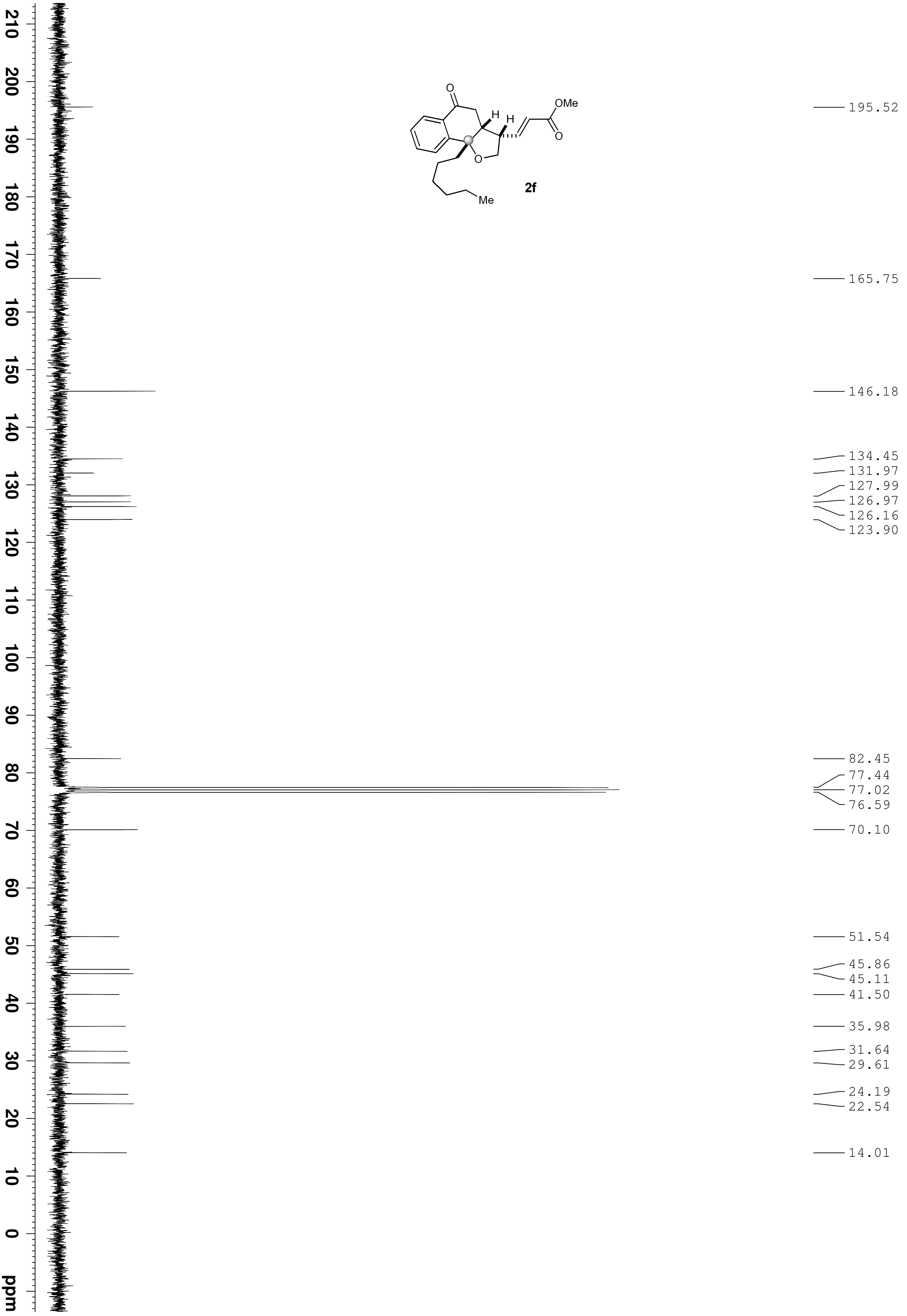

Supplementary Figure 78. ^{1}H NMR spectrum of compound **2f**

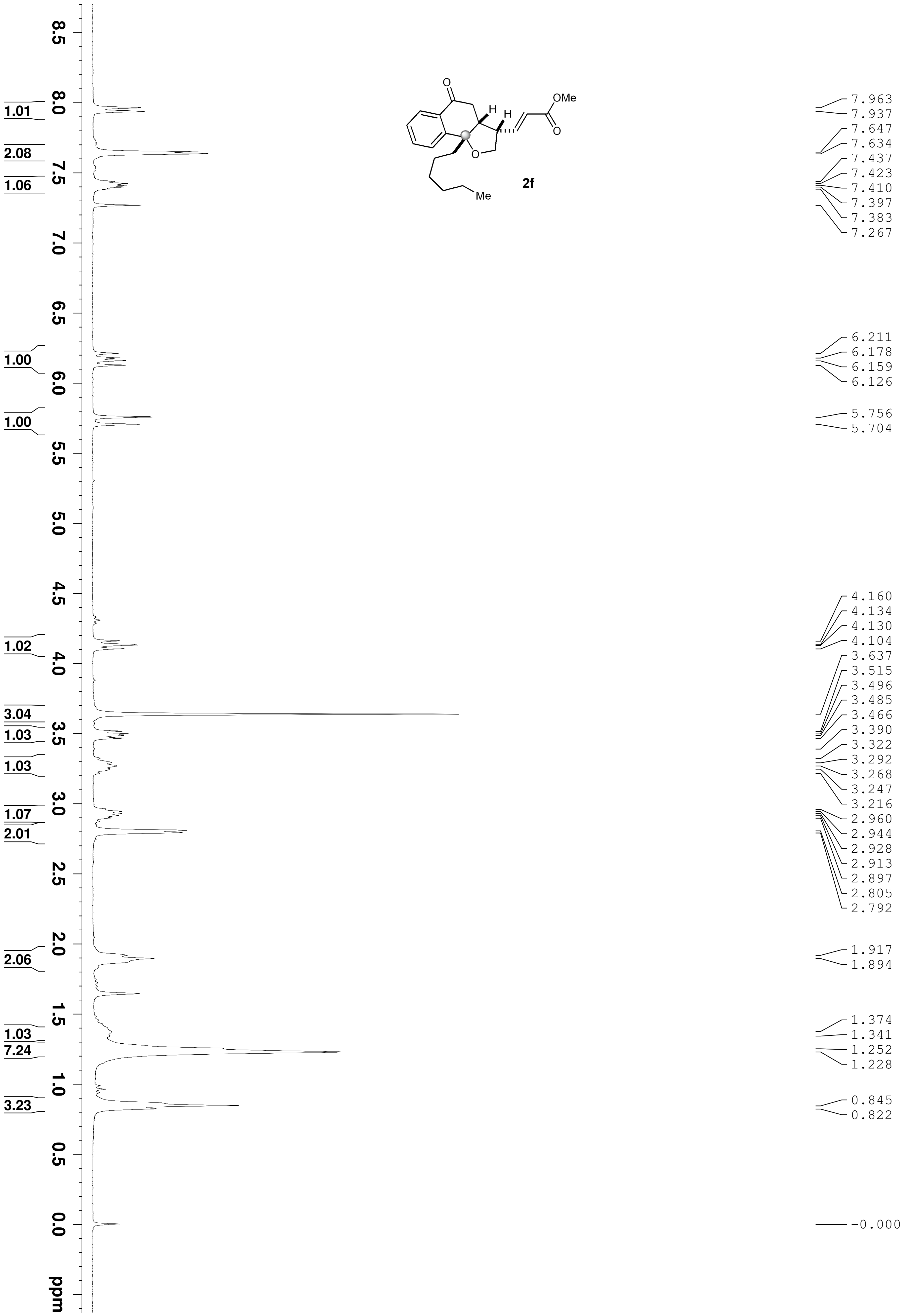

Supplementary Figure 79. ^{13}C NMR spectrum of compound **2g**

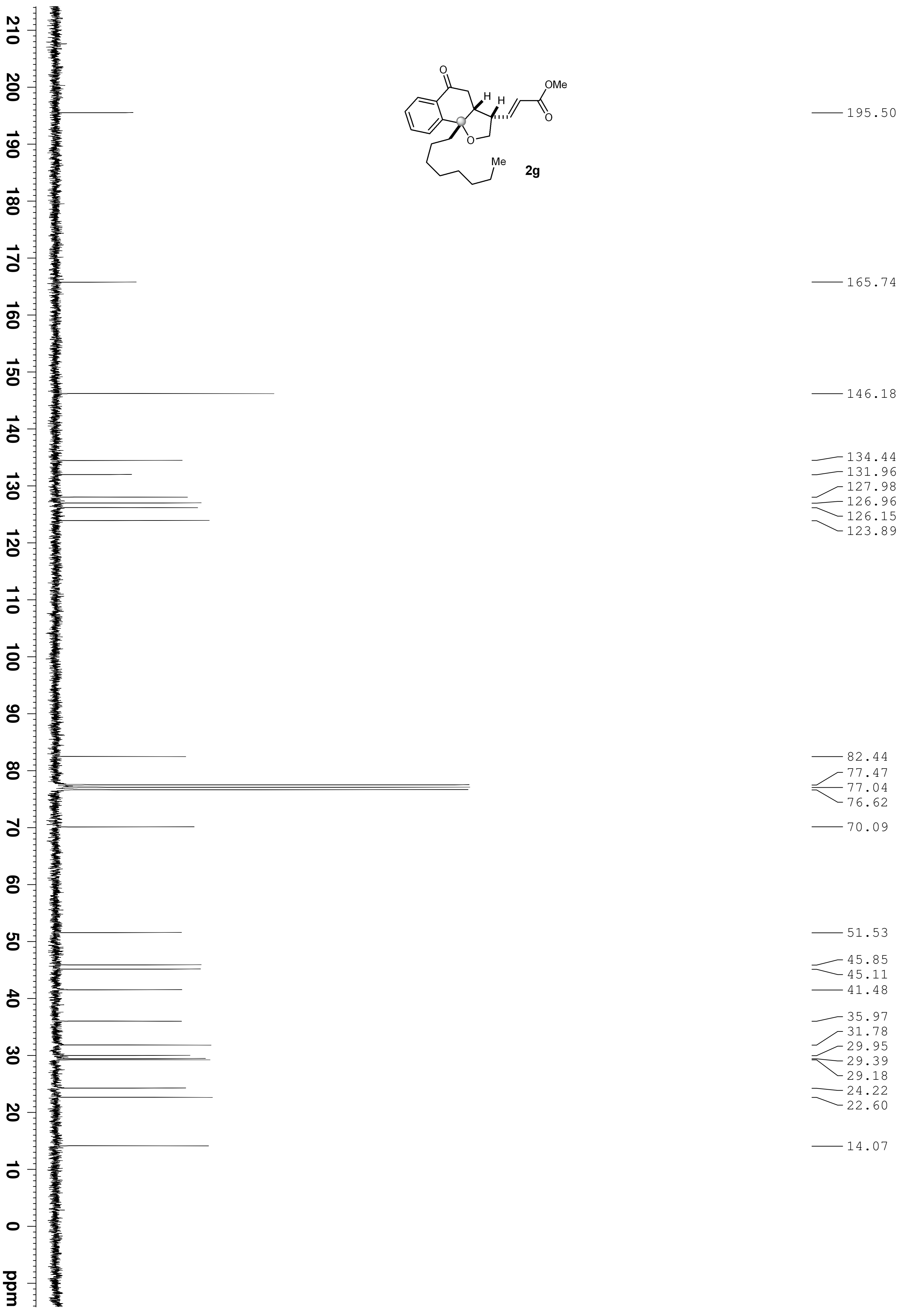

Supplementary Figure 80. ^{1}H NMR spectrum of compound **2g**

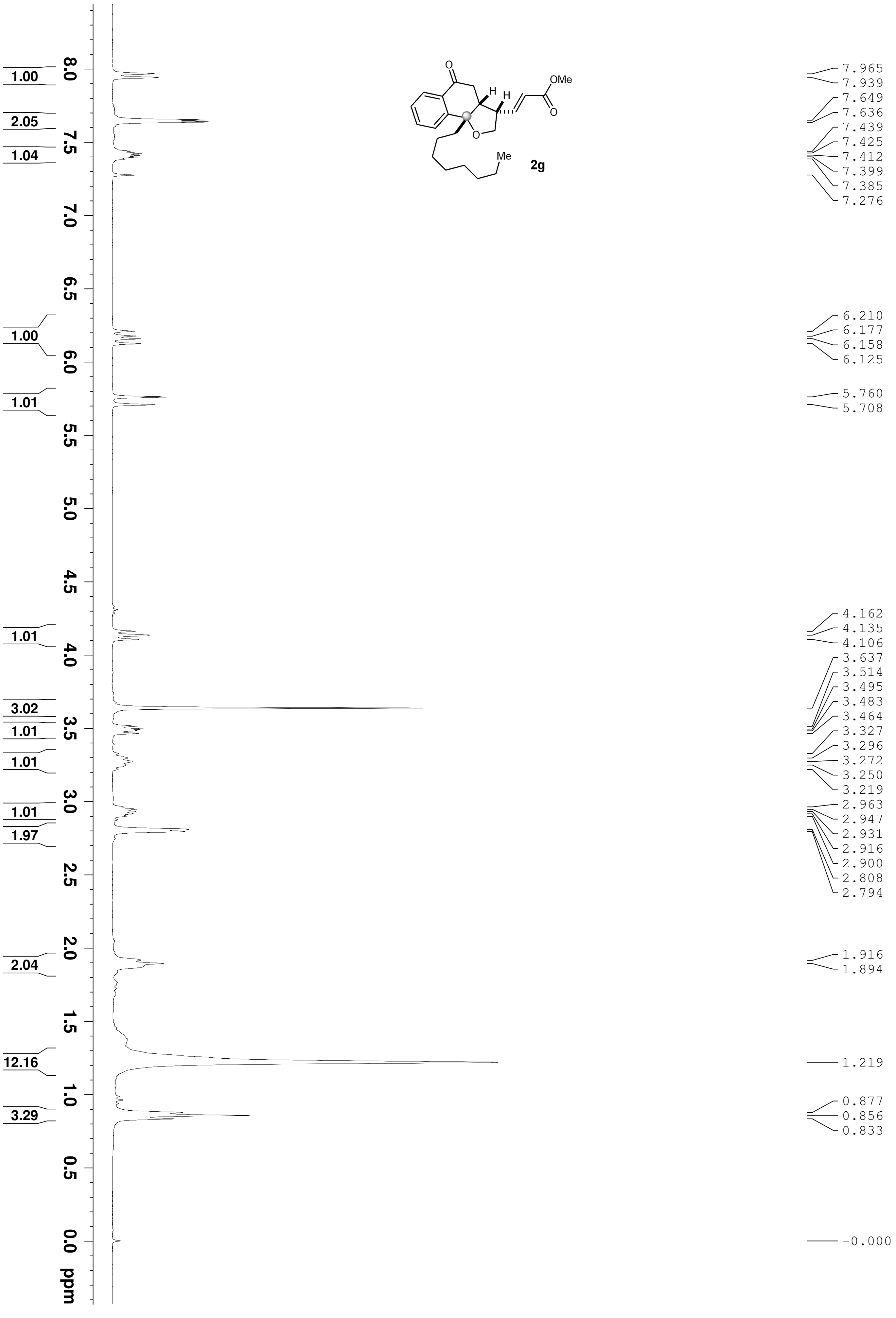

Supplementary Figure 81. ^{13}C NMR spectrum of compound **2h**

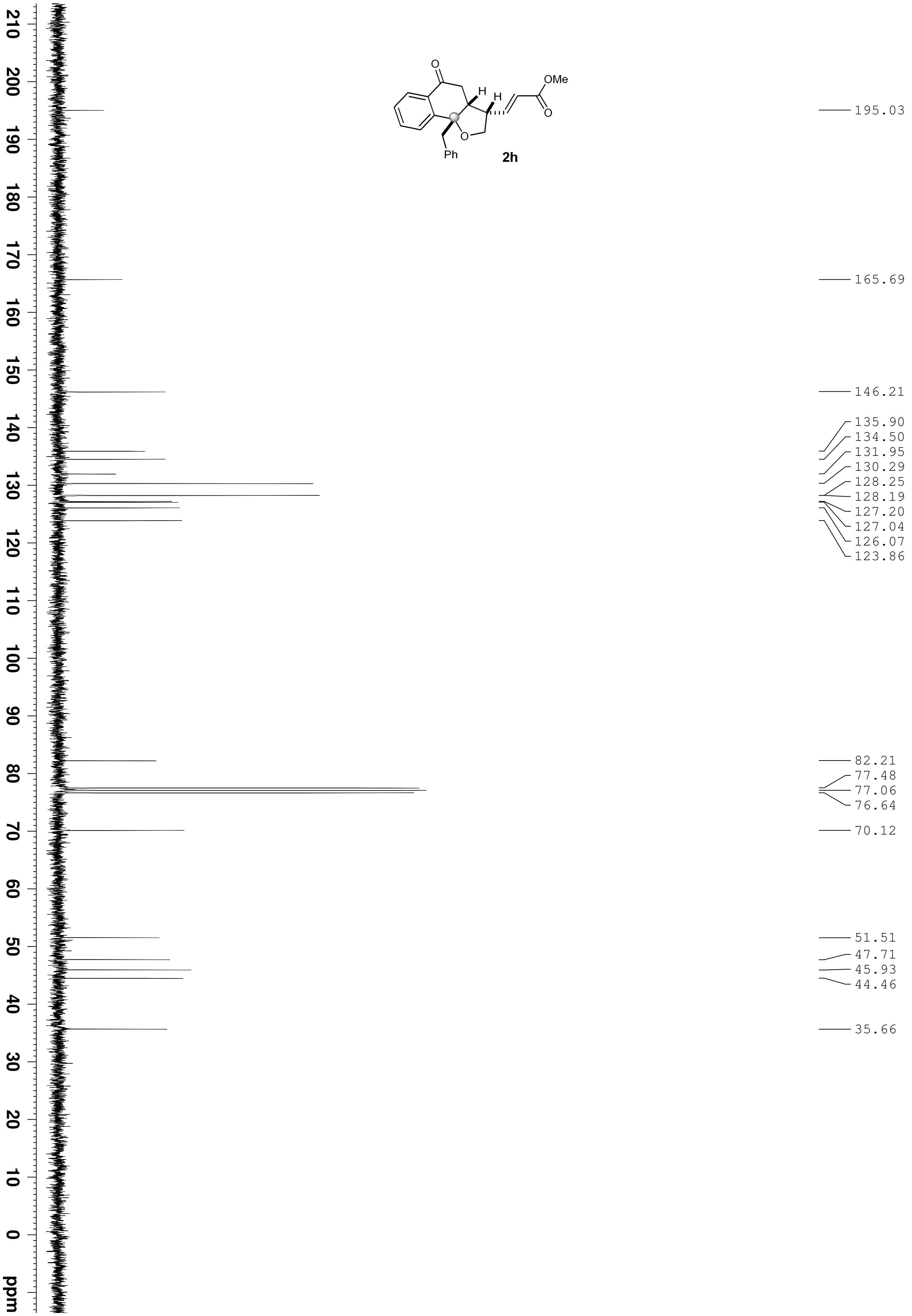

Supplementary Figure 82. ^{1}H NMR spectrum of compound **2h**

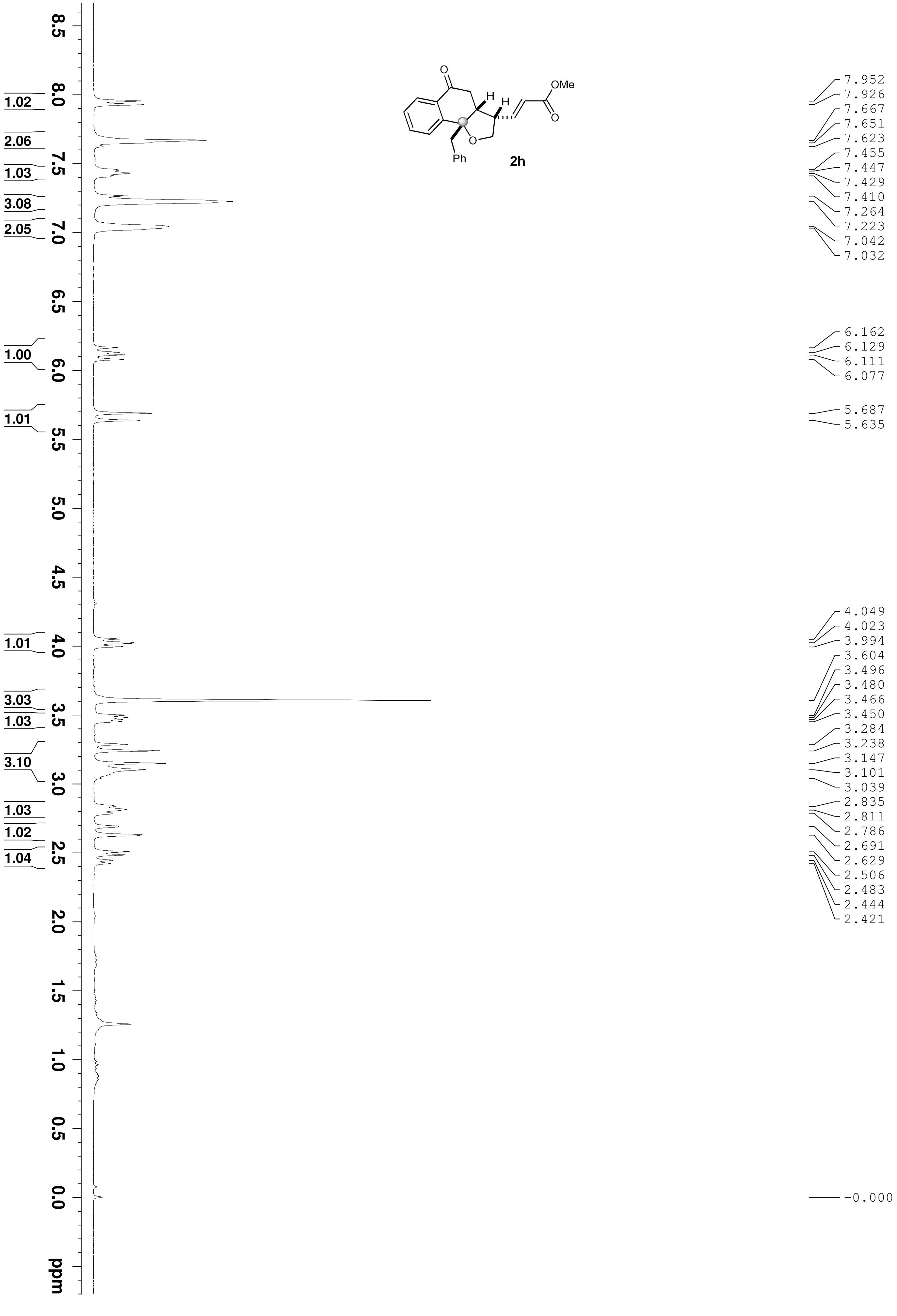

Supplementary Figure 83. ^{13}C NMR spectrum of compound **2i**

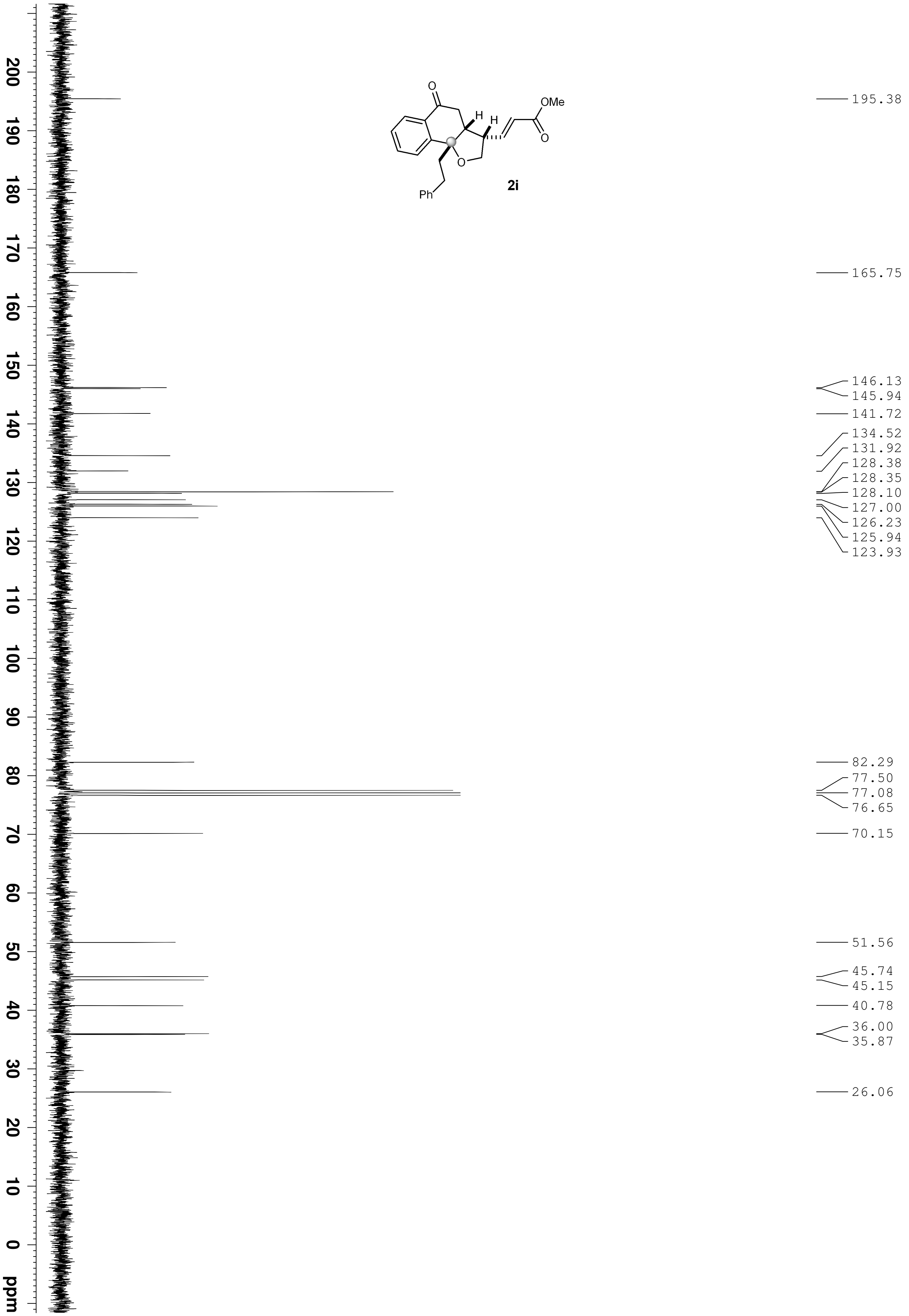

Supplementary Figure 84. ^{1}H NMR spectrum of compound **2i**

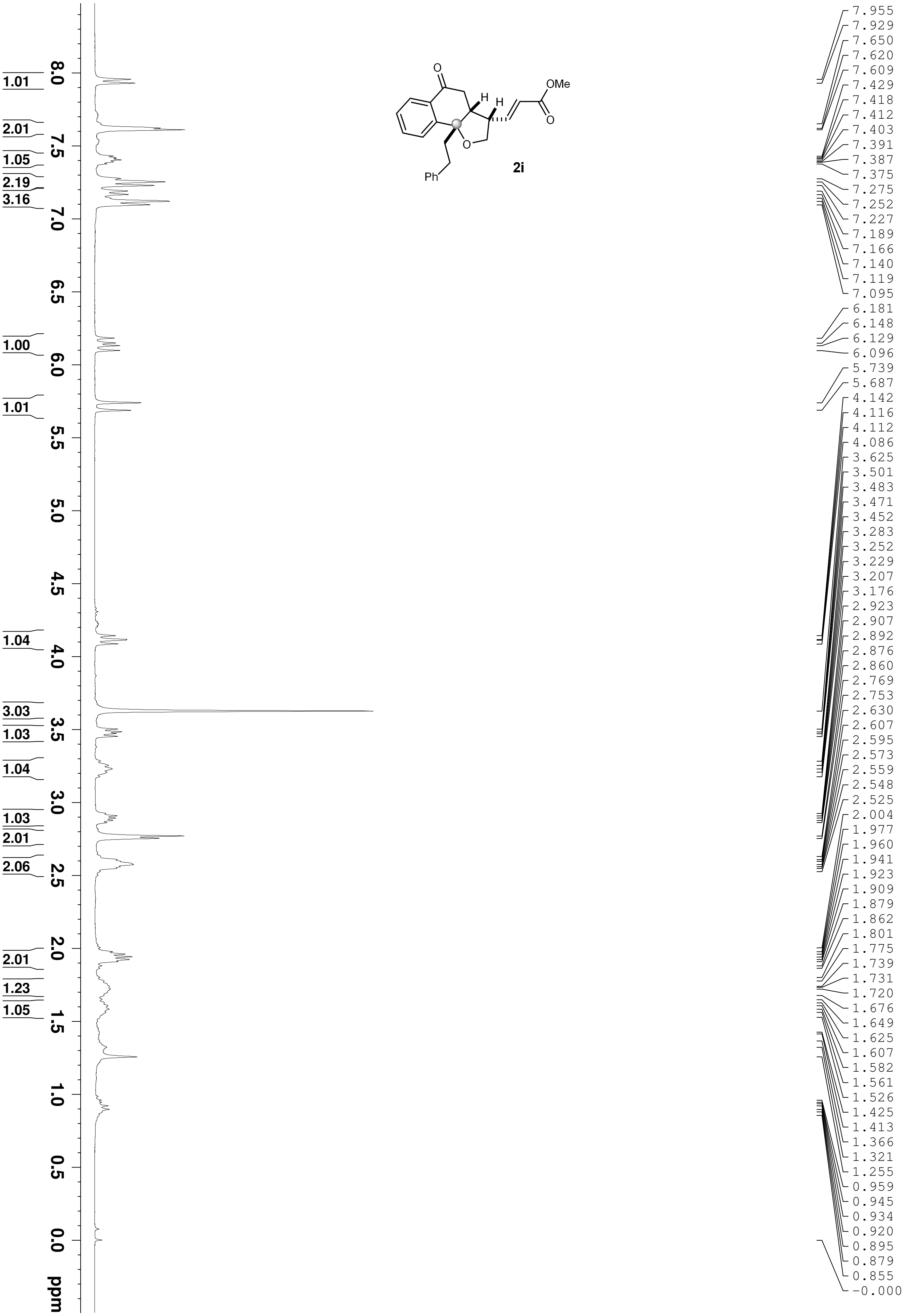

Supplementary Figure 85. ^{13}C NMR spectrum of compound **2j**

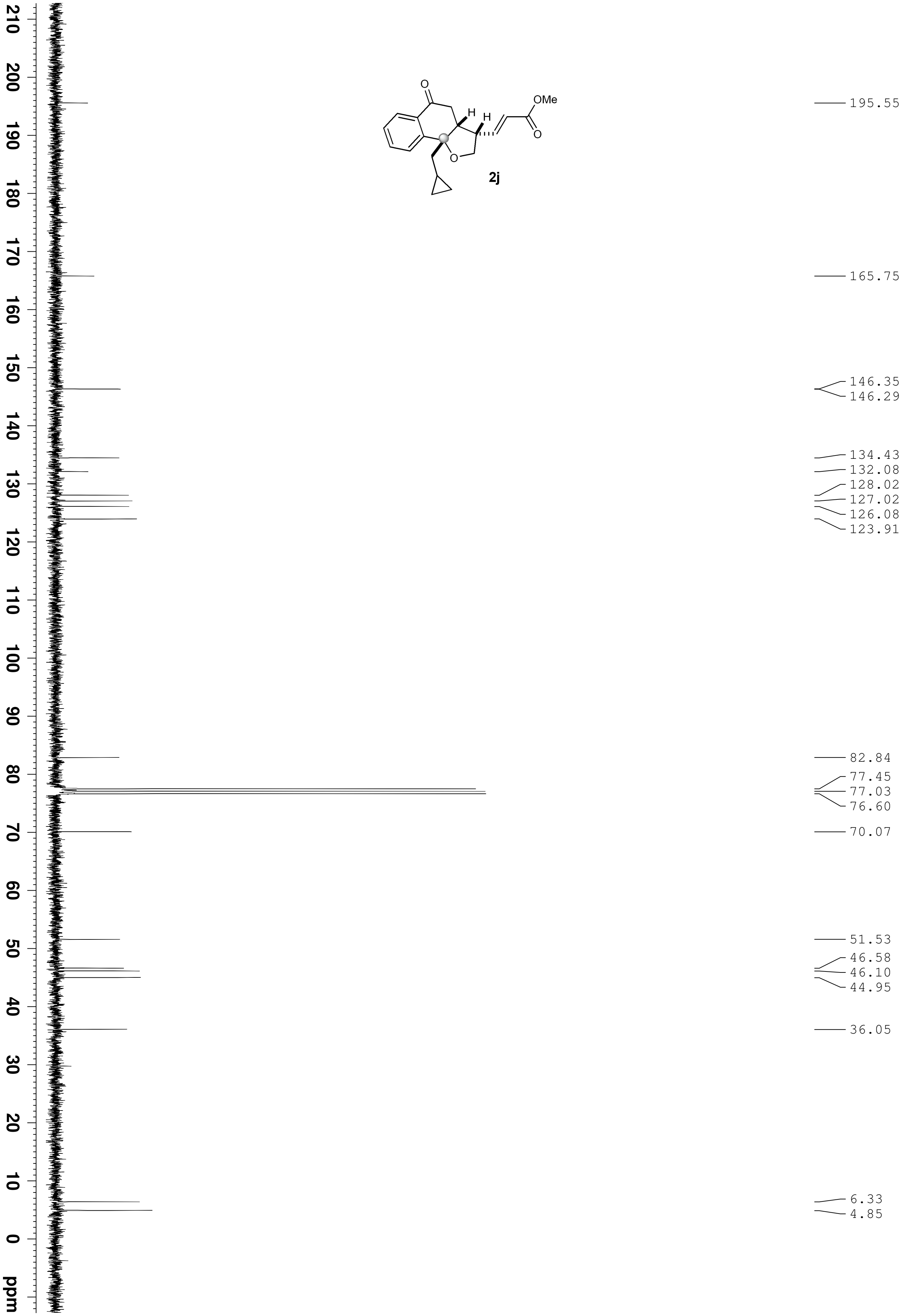

Supplementary Figure 86. ^{1}H NMR spectrum of compound **2j**

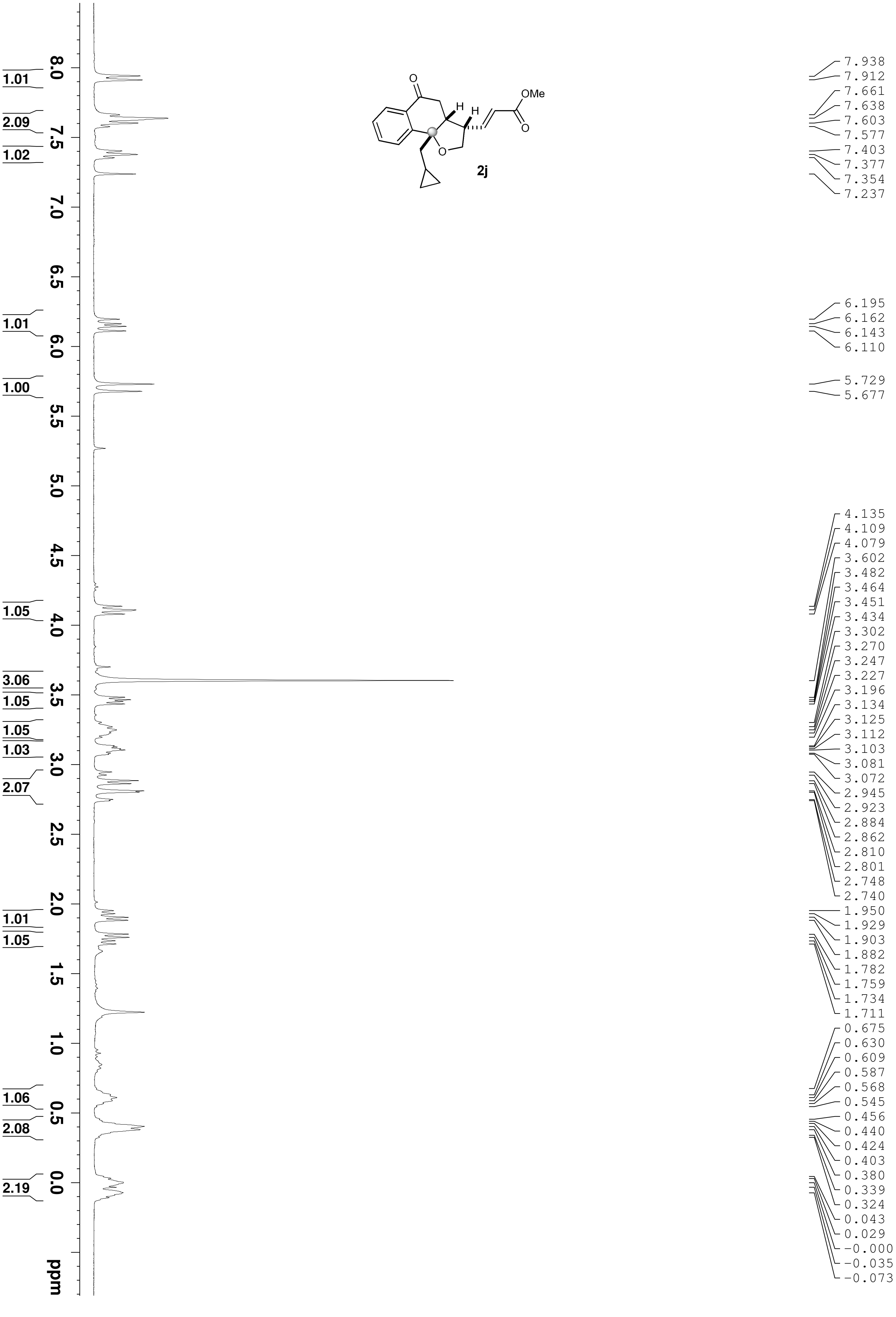

Supplementary Figure 87. ^{13}C NMR spectrum of compound **2k**

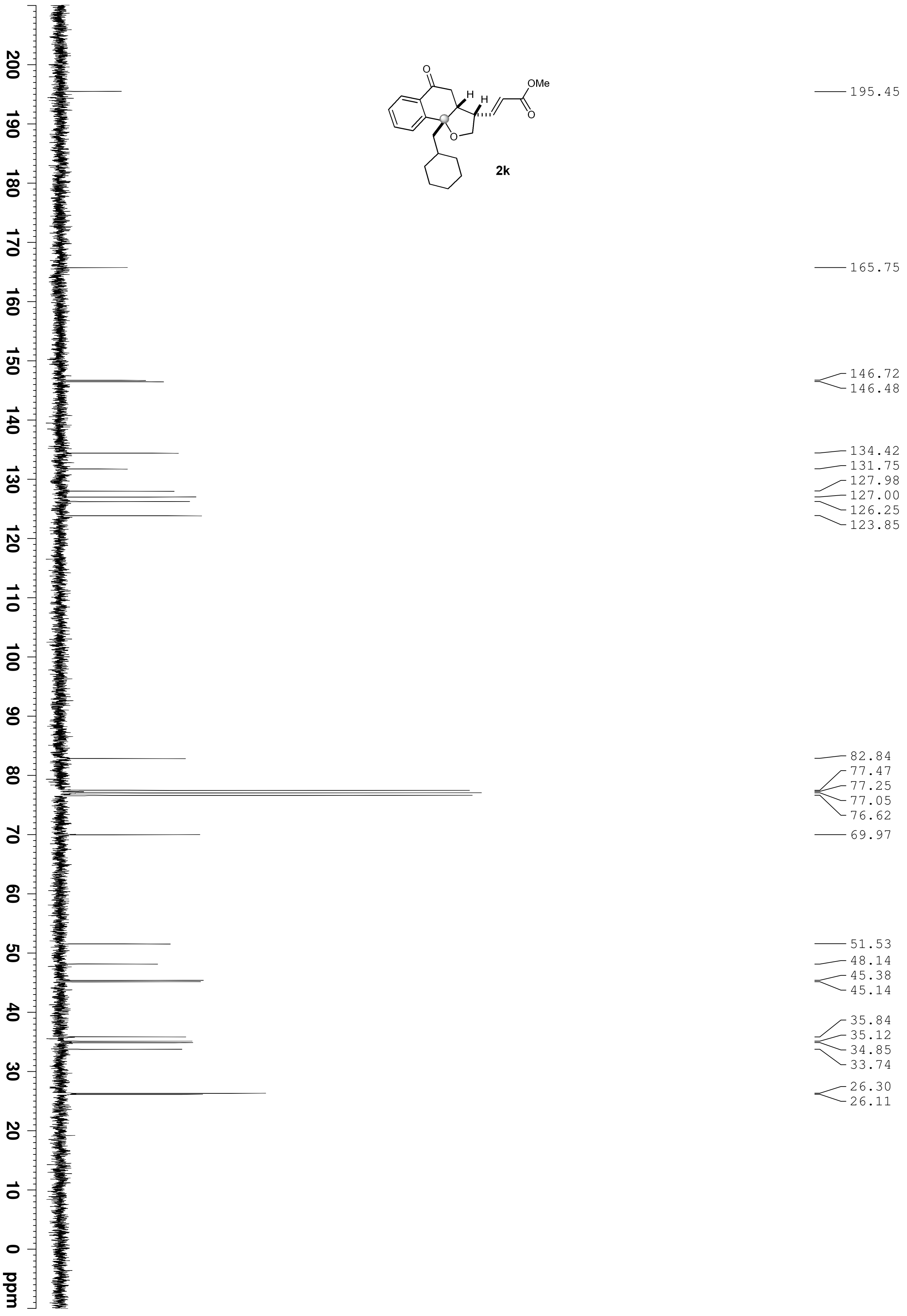

Supplementary Figure 88. ^{1}H NMR spectrum of compound **2k**

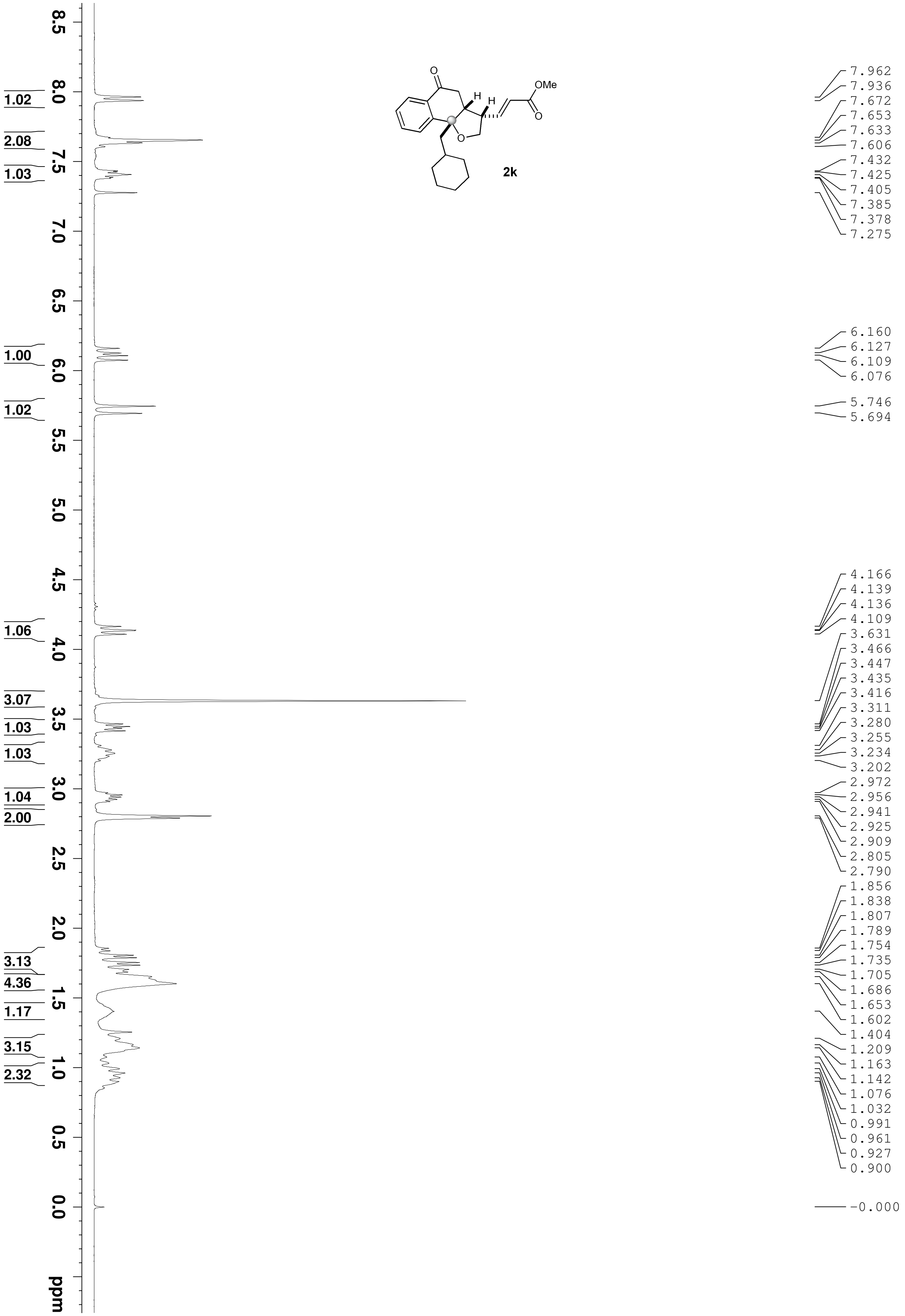

Supplementary Figure 89. ^{13}C NMR spectrum of compound **2l**

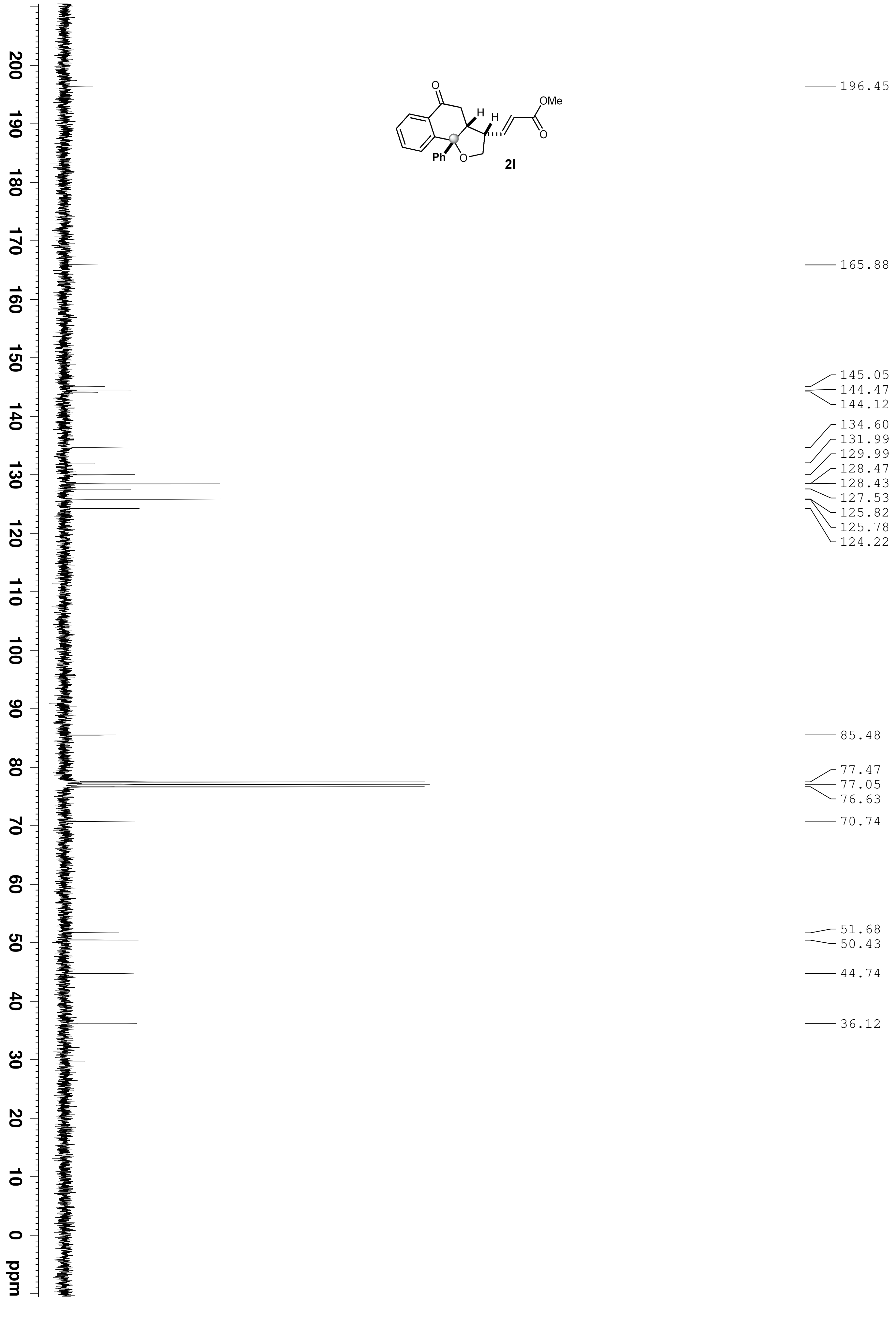

Supplementary Figure 90. ^{1}H NMR spectrum of compound **2l**

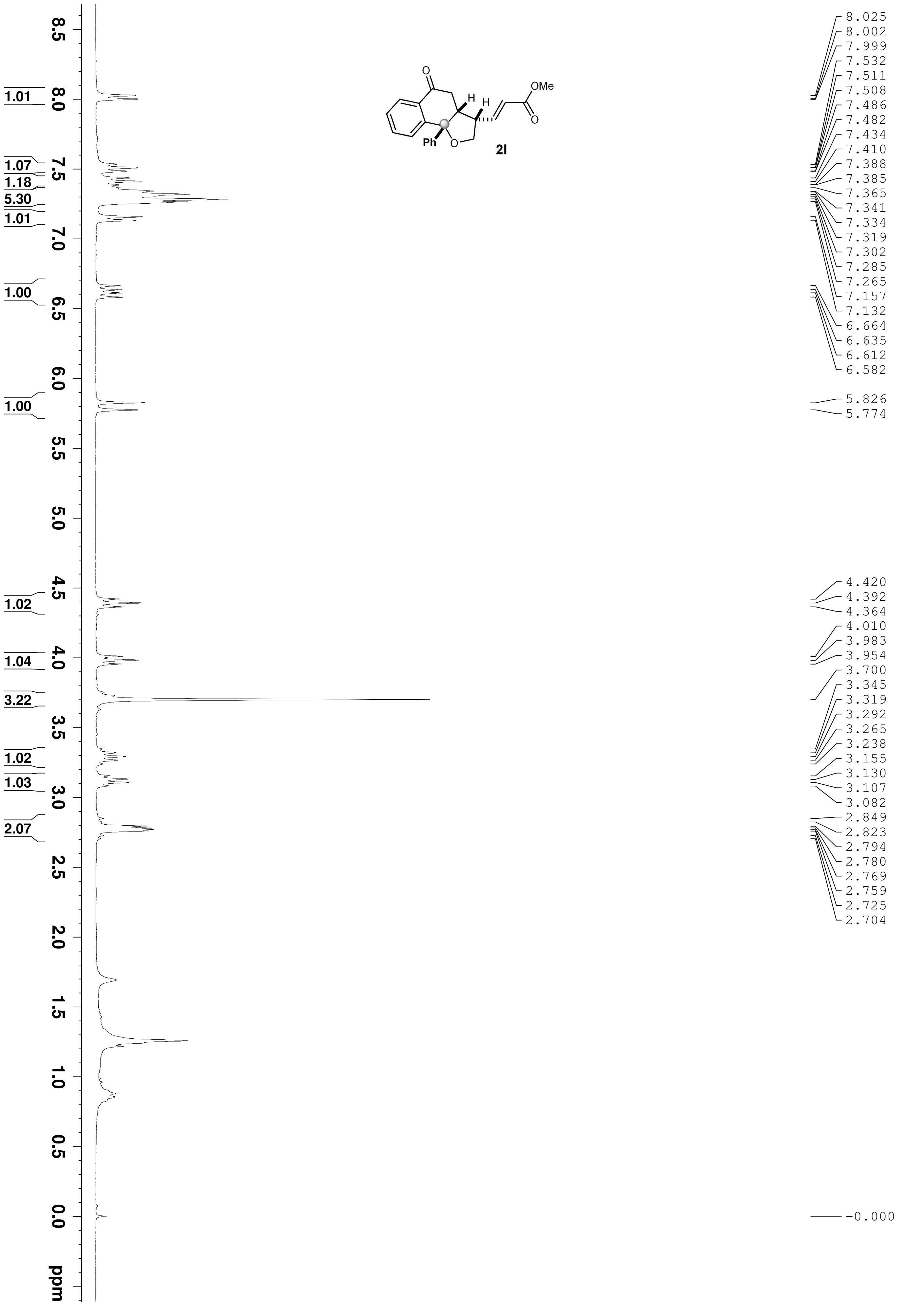

Supplementary Figure 91. ^{13}C NMR spectrum of compound **2m**

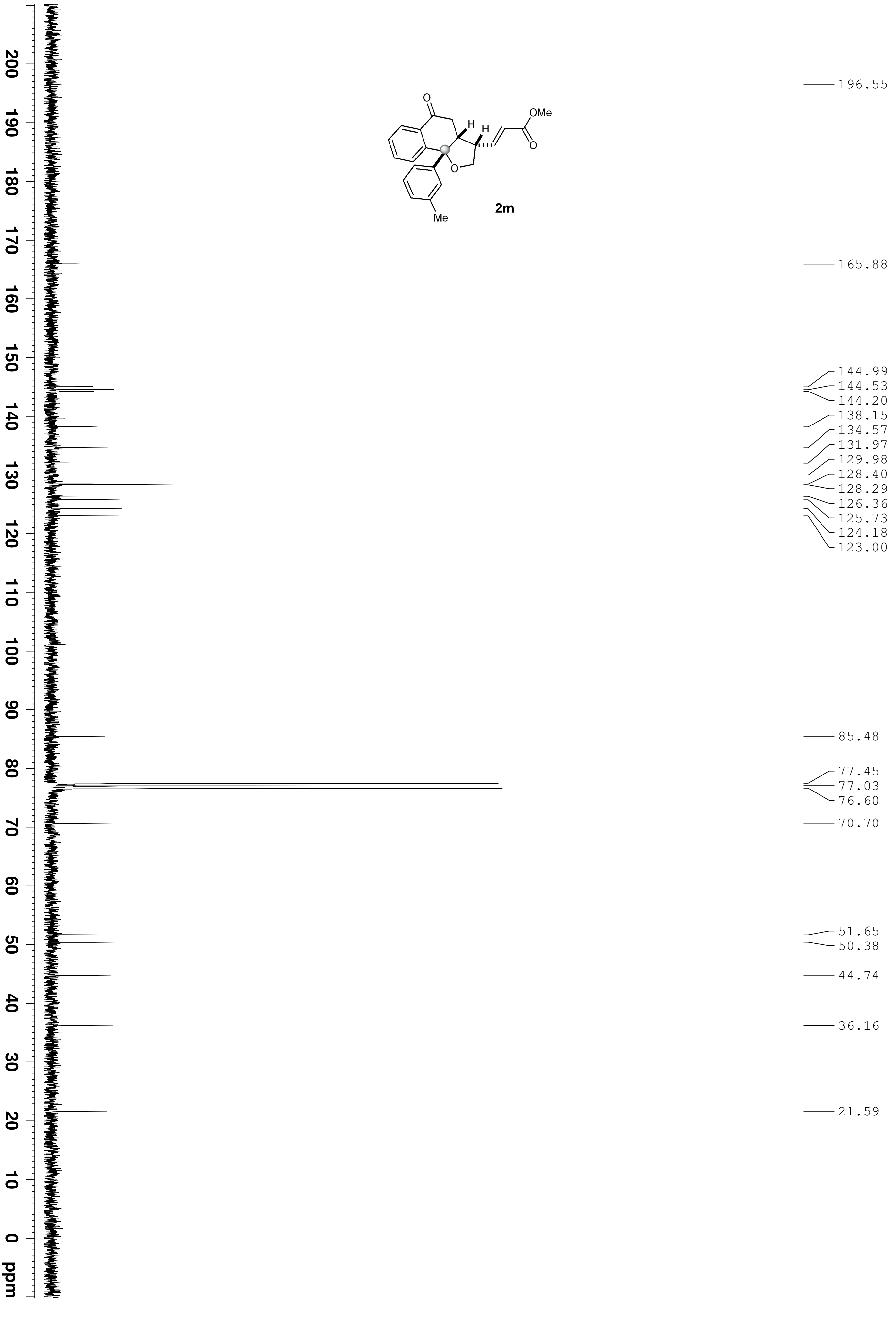

Supplementary Figure 92. ^{1}H NMR spectrum of compound **2m**

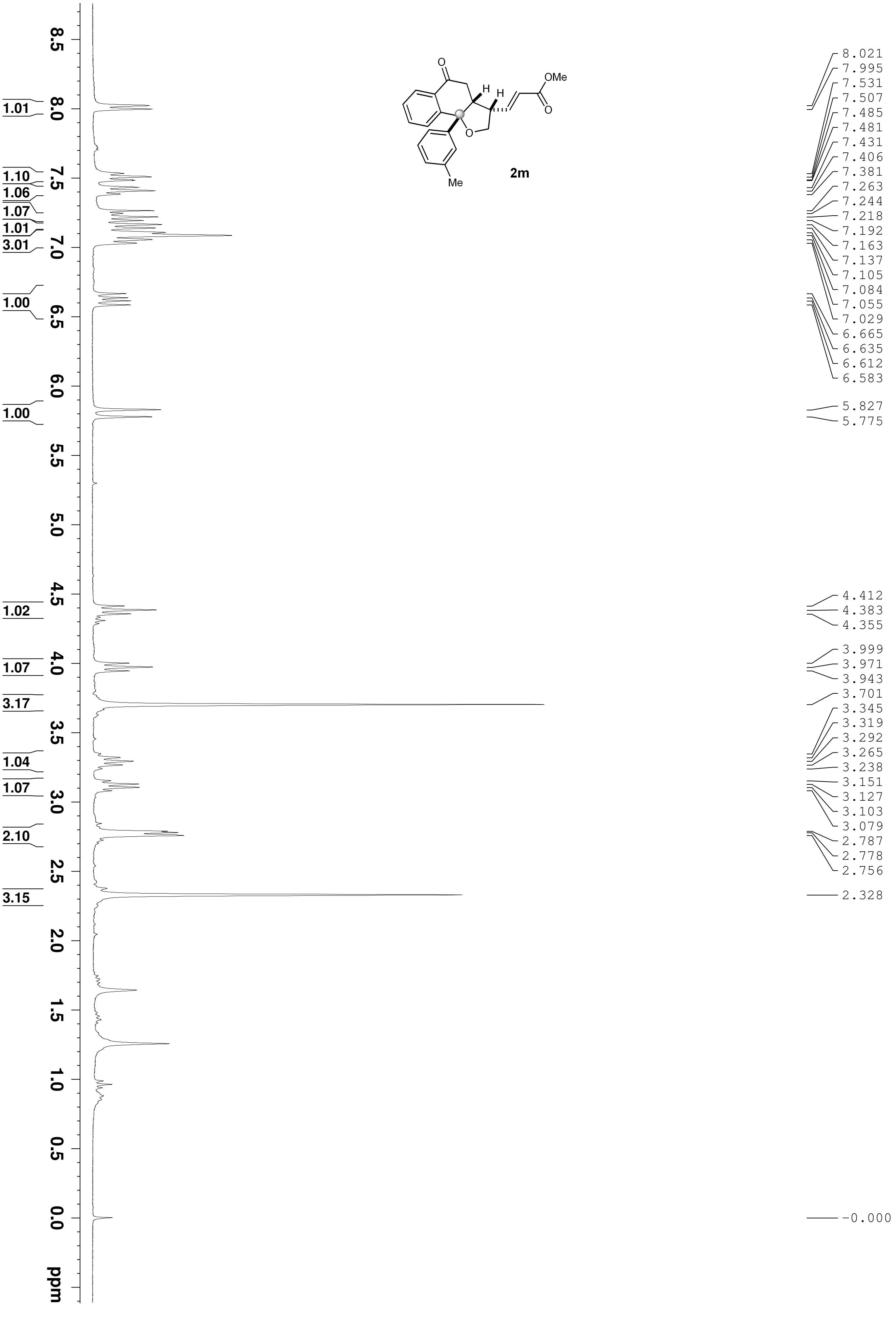

Supplementary Figure 93. ^{13}C NMR spectrum of compound **2n**

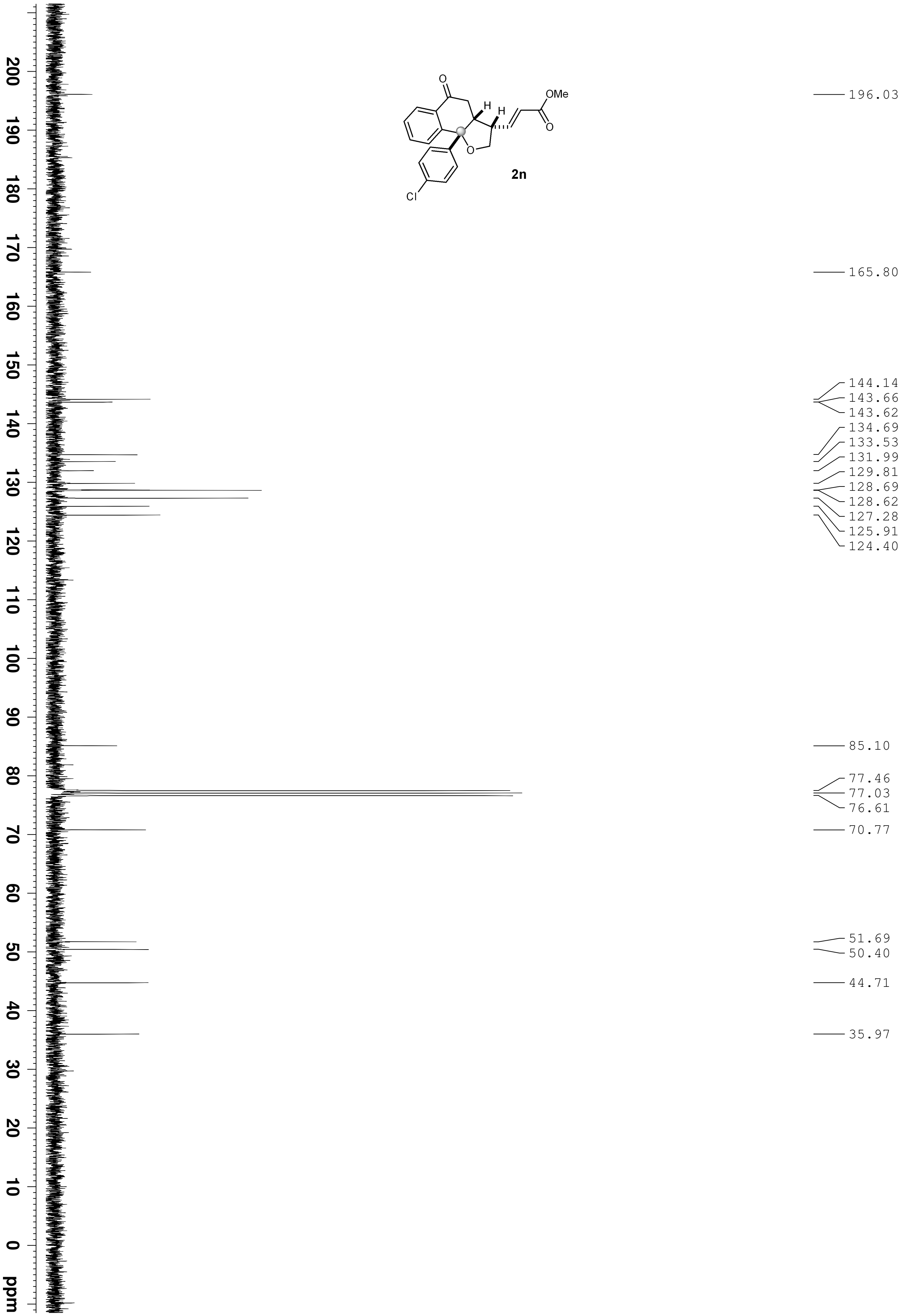

Supplementary Figure 94. ^{1}H NMR spectrum of compound **2n**

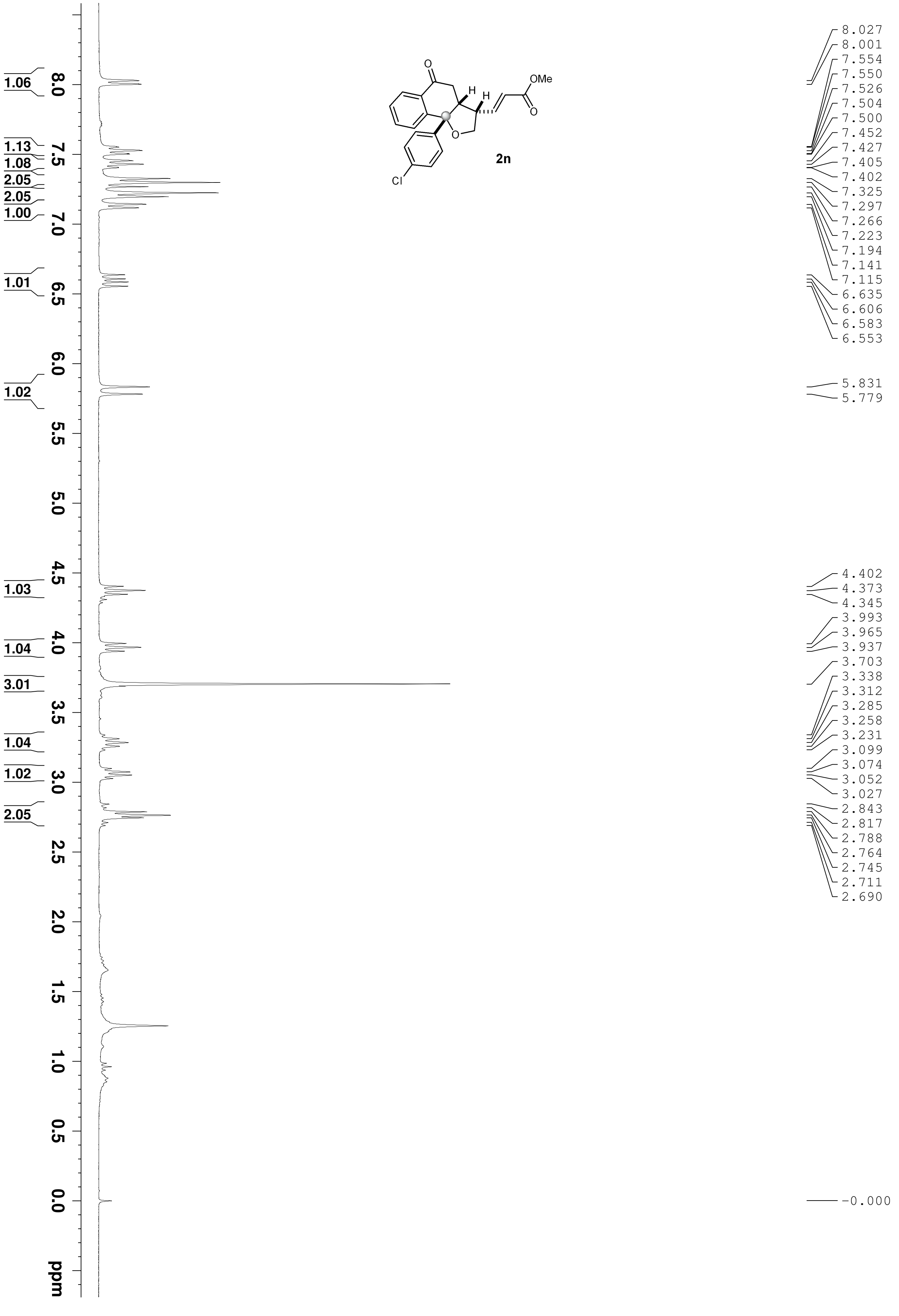

Supplementary Figure 95. ^{13}C NMR spectrum of compound **2o**

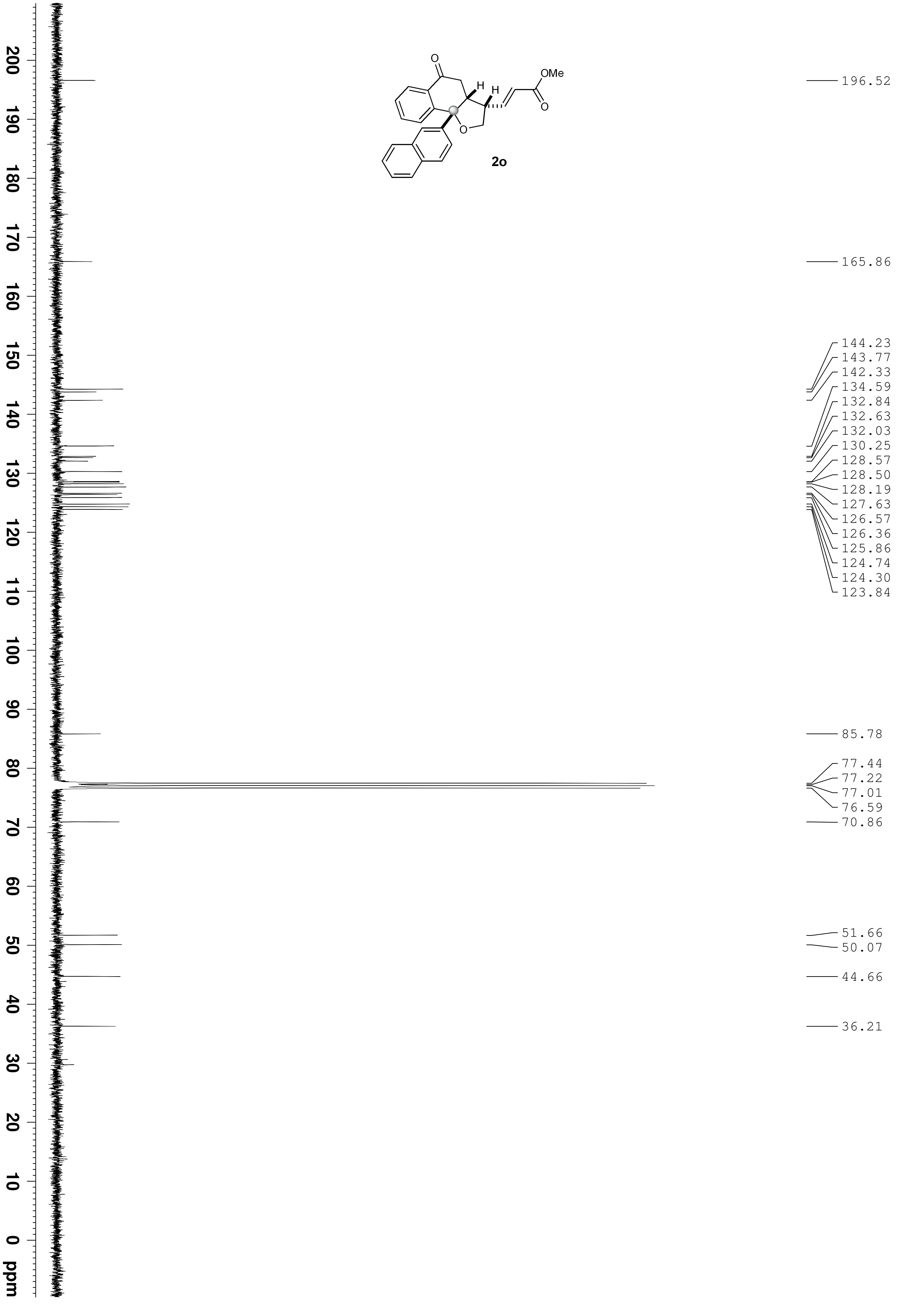

Supplementary Figure 96. ^{1}H NMR spectrum of compound **2o**

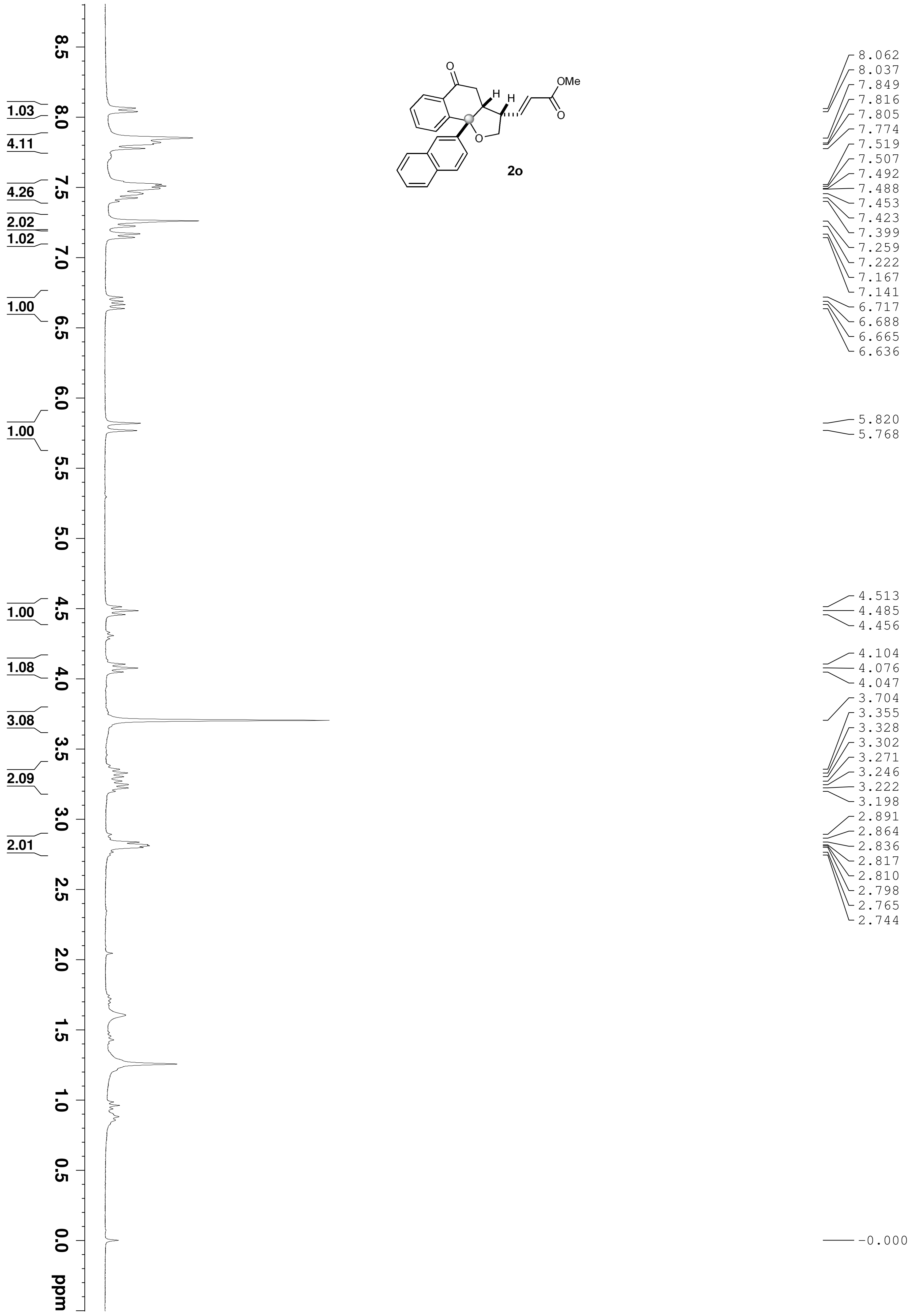

Supplementary Figure 97. ^{13}C NMR spectrum of compound **2p**

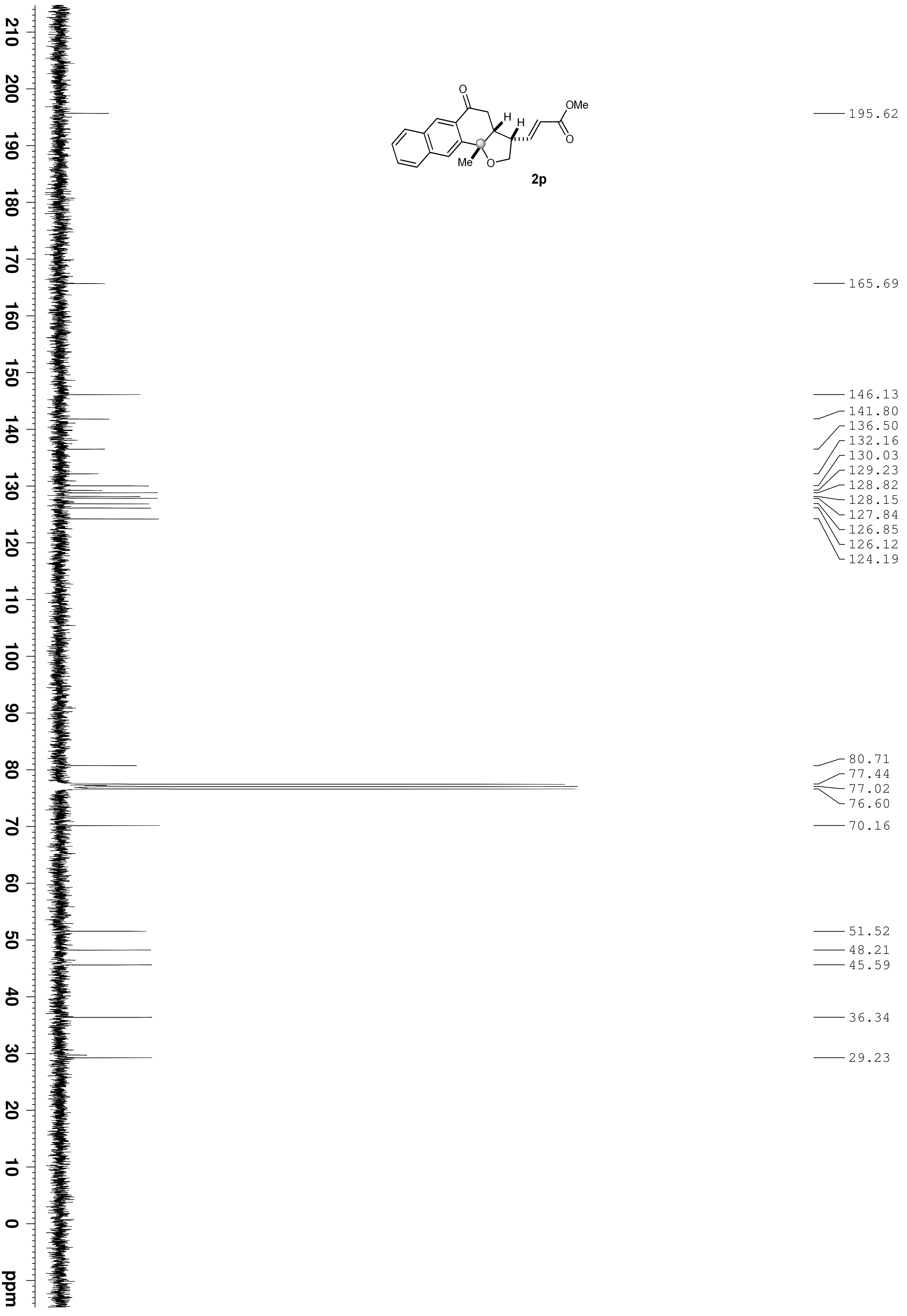

Supplementary Figure 98. ^{1}H NMR spectrum of compound **2p**

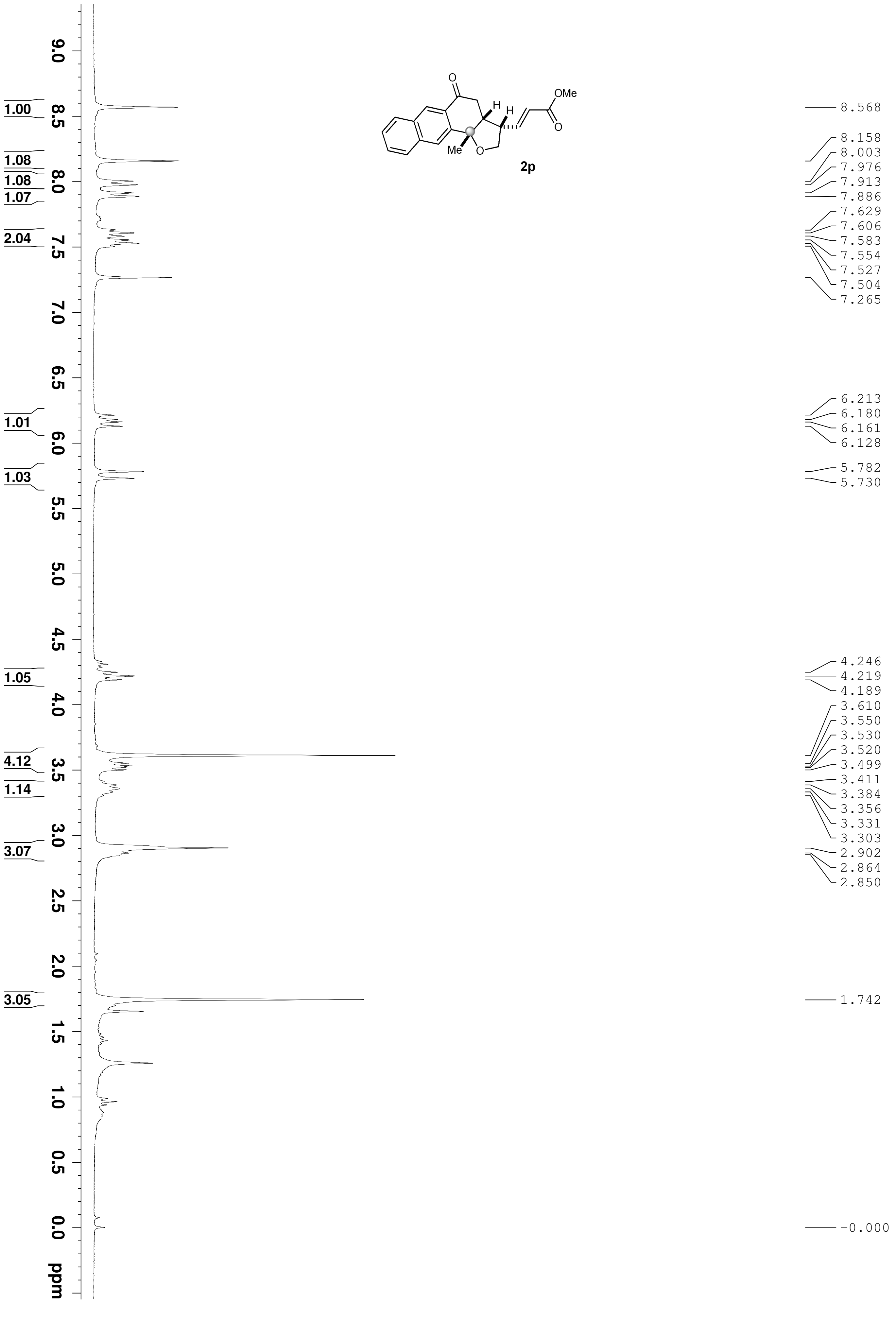

Supplementary Figure 99. ^{13}C NMR spectrum of compound **2q**

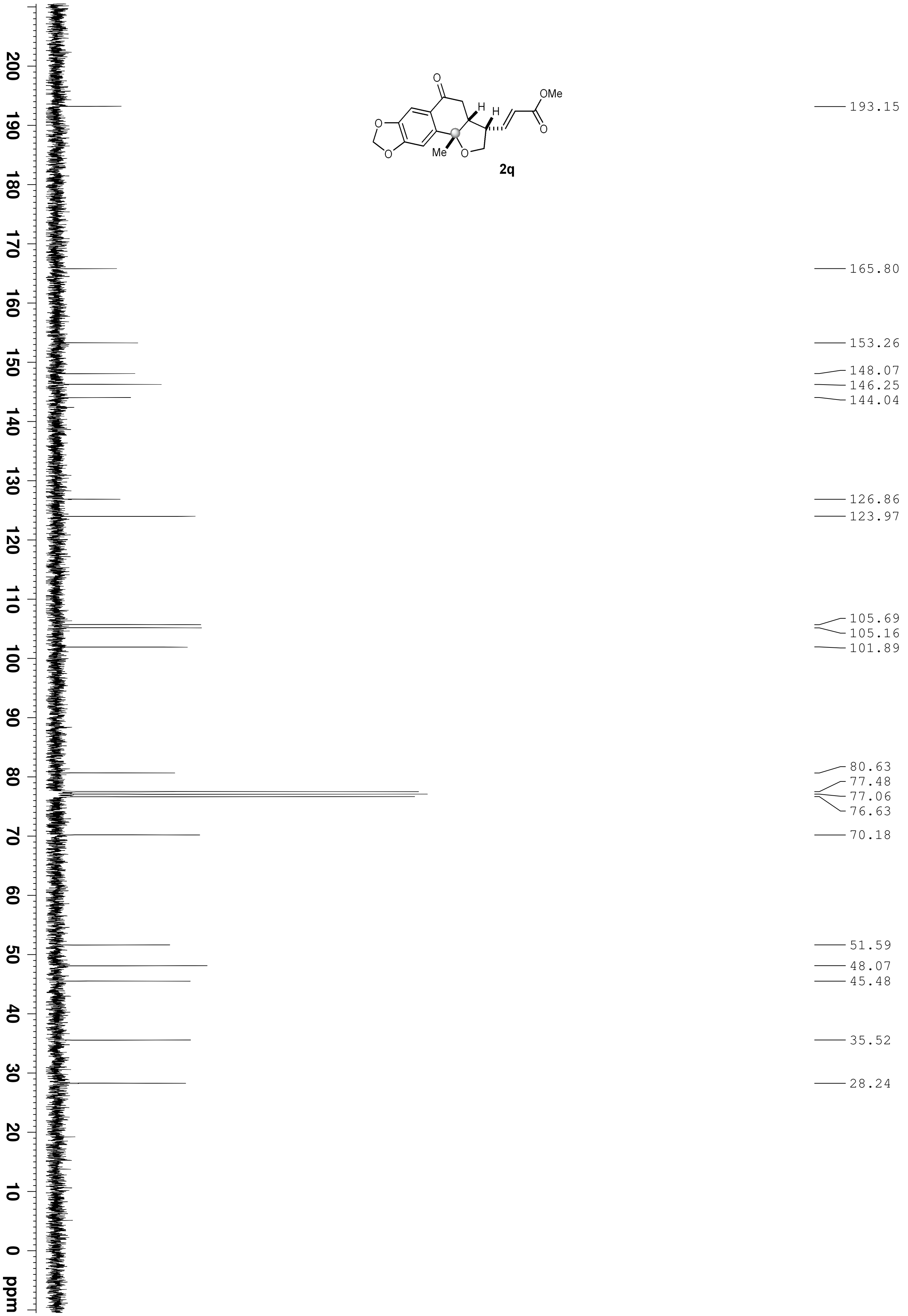

Supplementary Figure 100. ^{1}H NMR spectrum of compound **2q**

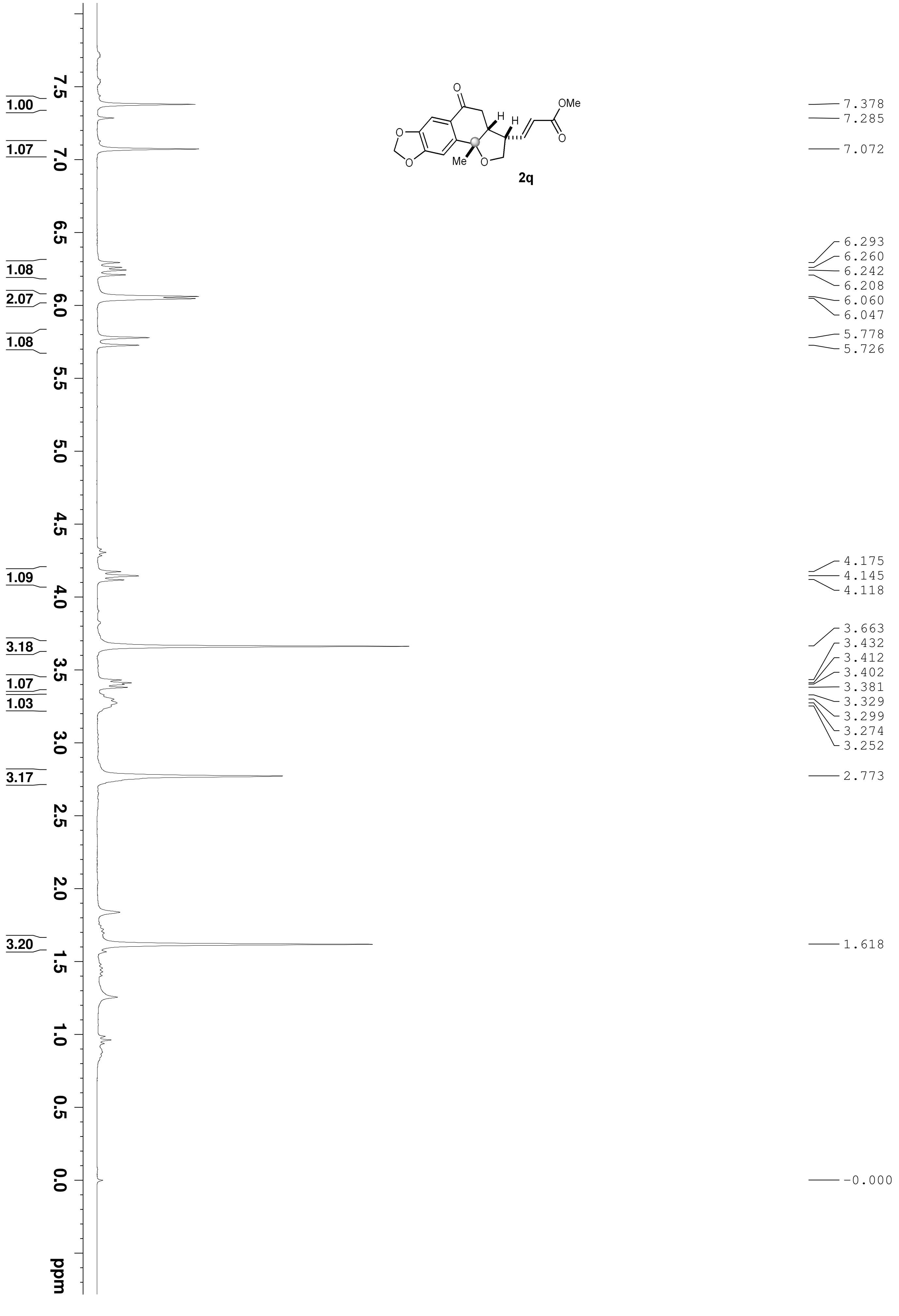

Supplementary Figure 101. ^{13}C NMR spectrum of compound **2r**

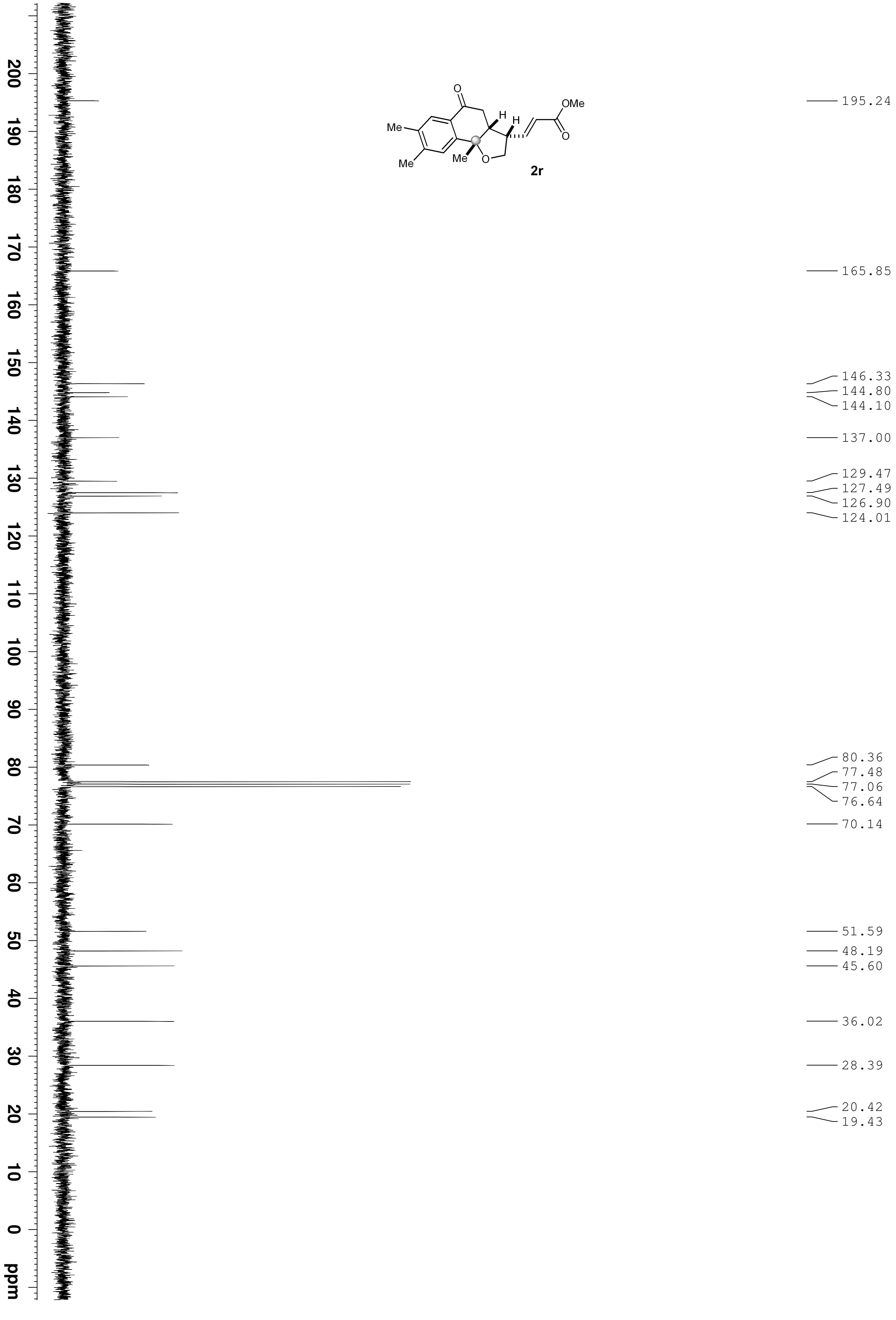

Supplementary Figure 102. ^{1}H NMR spectrum of compound **2r**

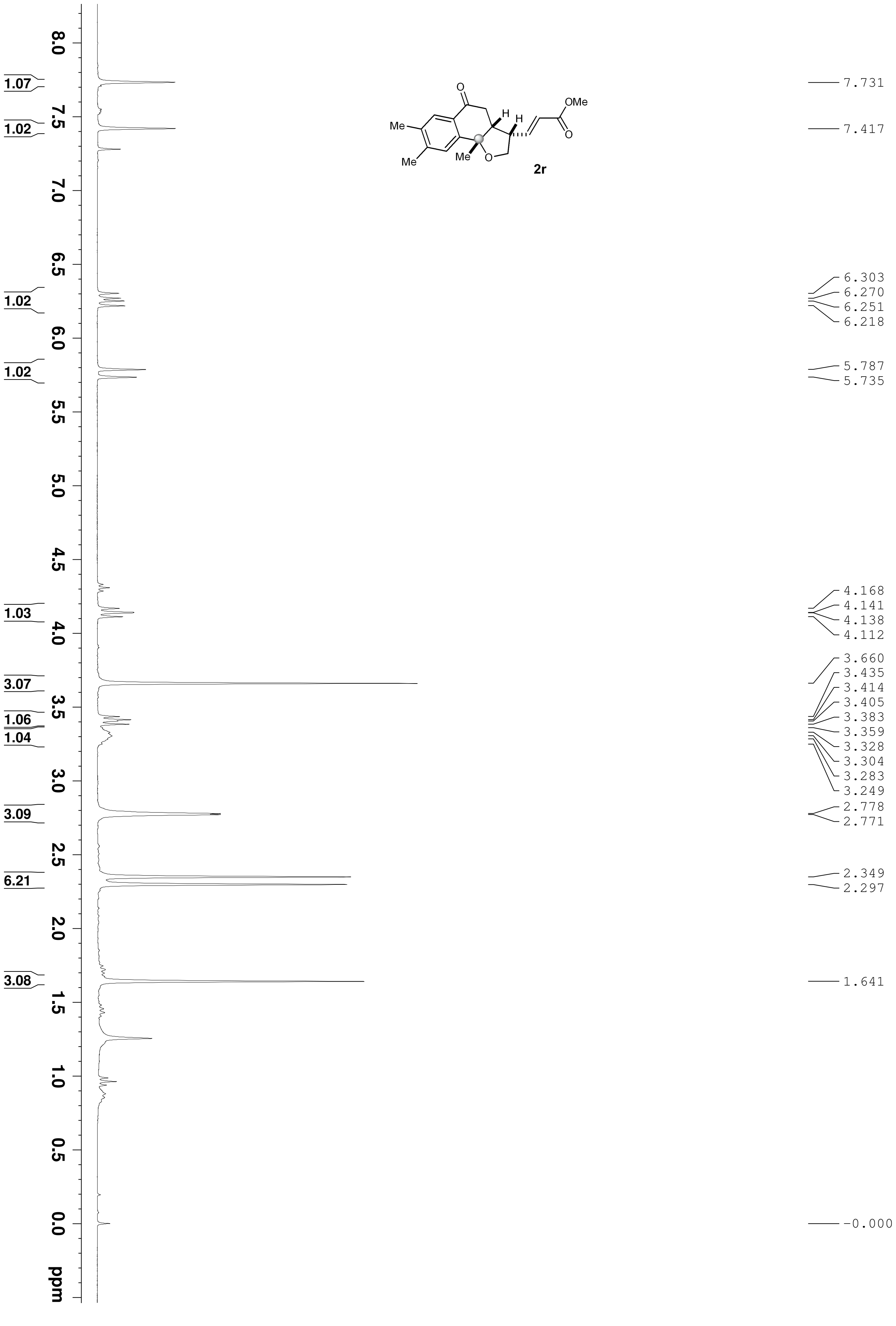

Supplementary Figure 103. ^{13}C NMR spectrum of compound **2s**

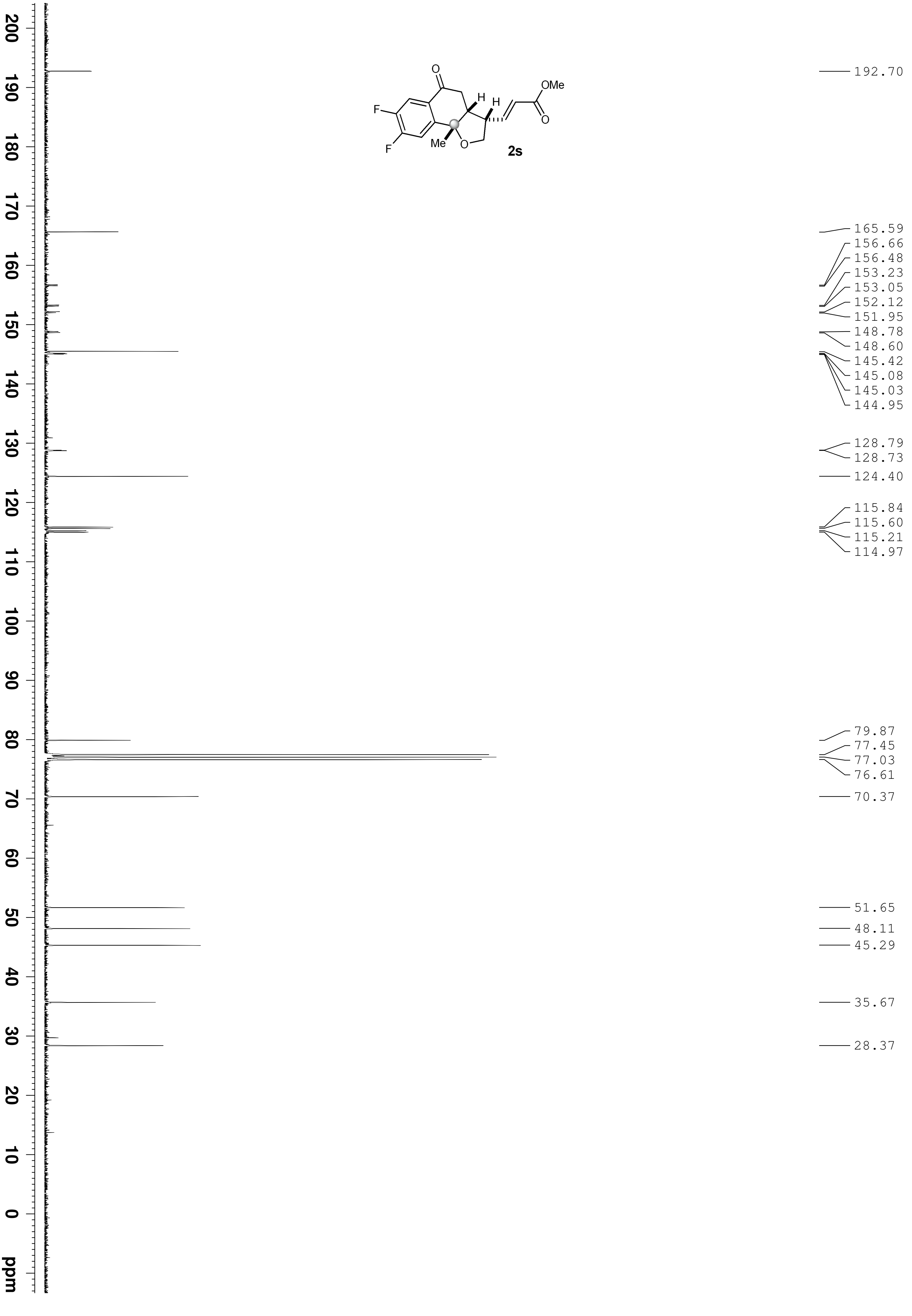

Supplementary Figure 104. ^{1}H NMR spectrum of compound **2s**

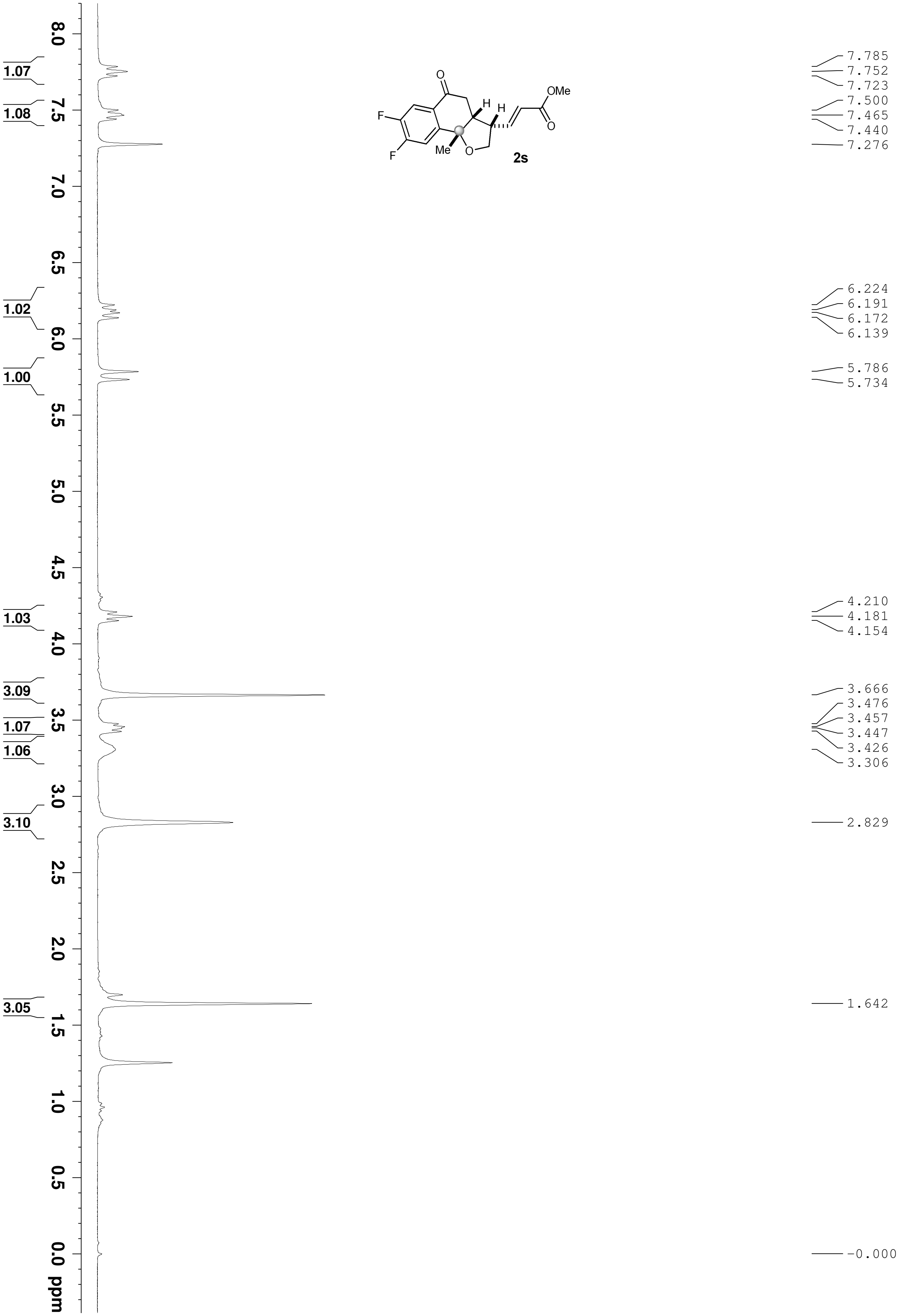

Supplementary Figure 105. ^{13}C NMR spectrum of compound **2t**

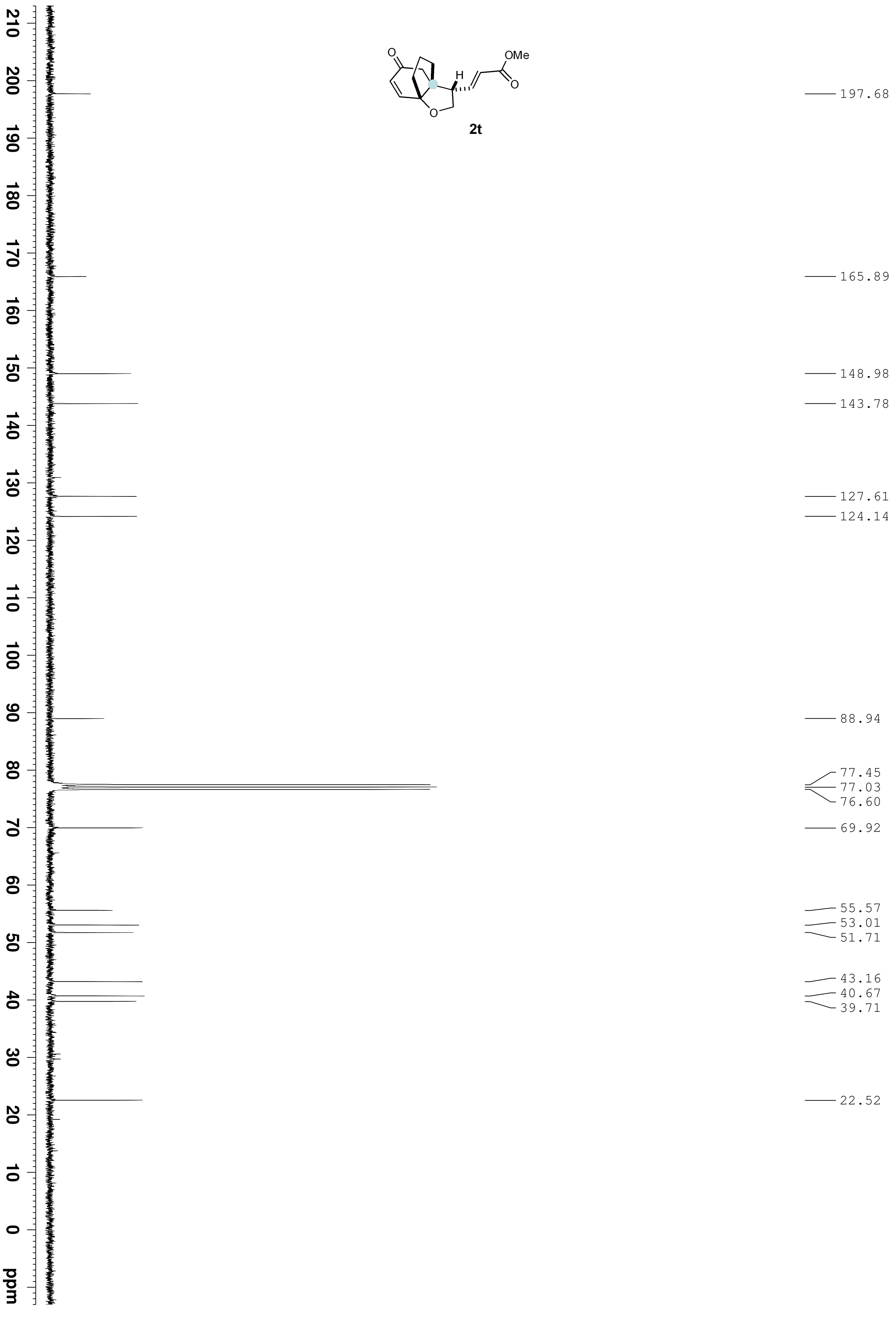

Supplementary Figure 106. ^{1}H NMR spectrum of compound **2t**

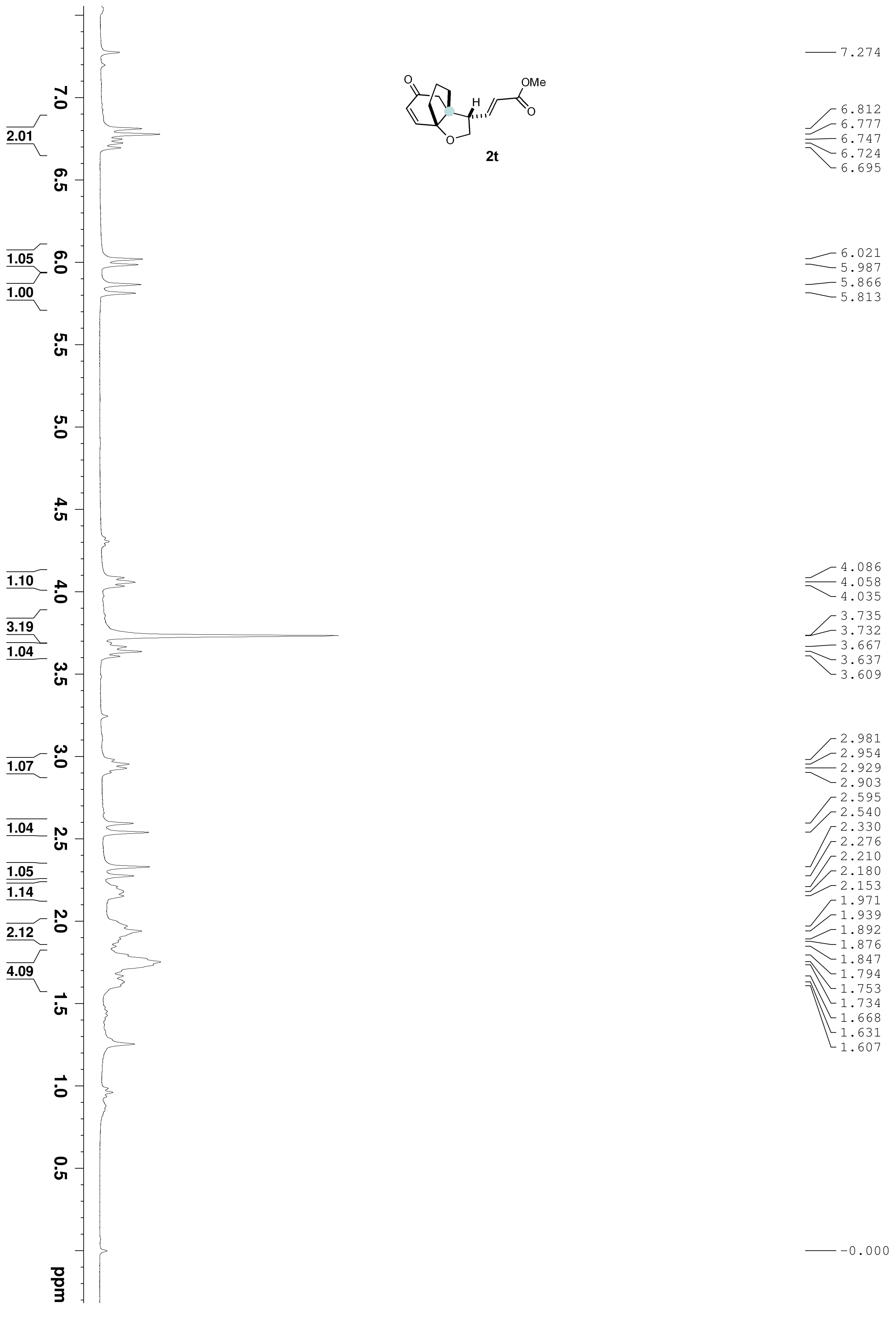

Supplementary Figure 107. ^{13}C NMR spectrum of compound **3**

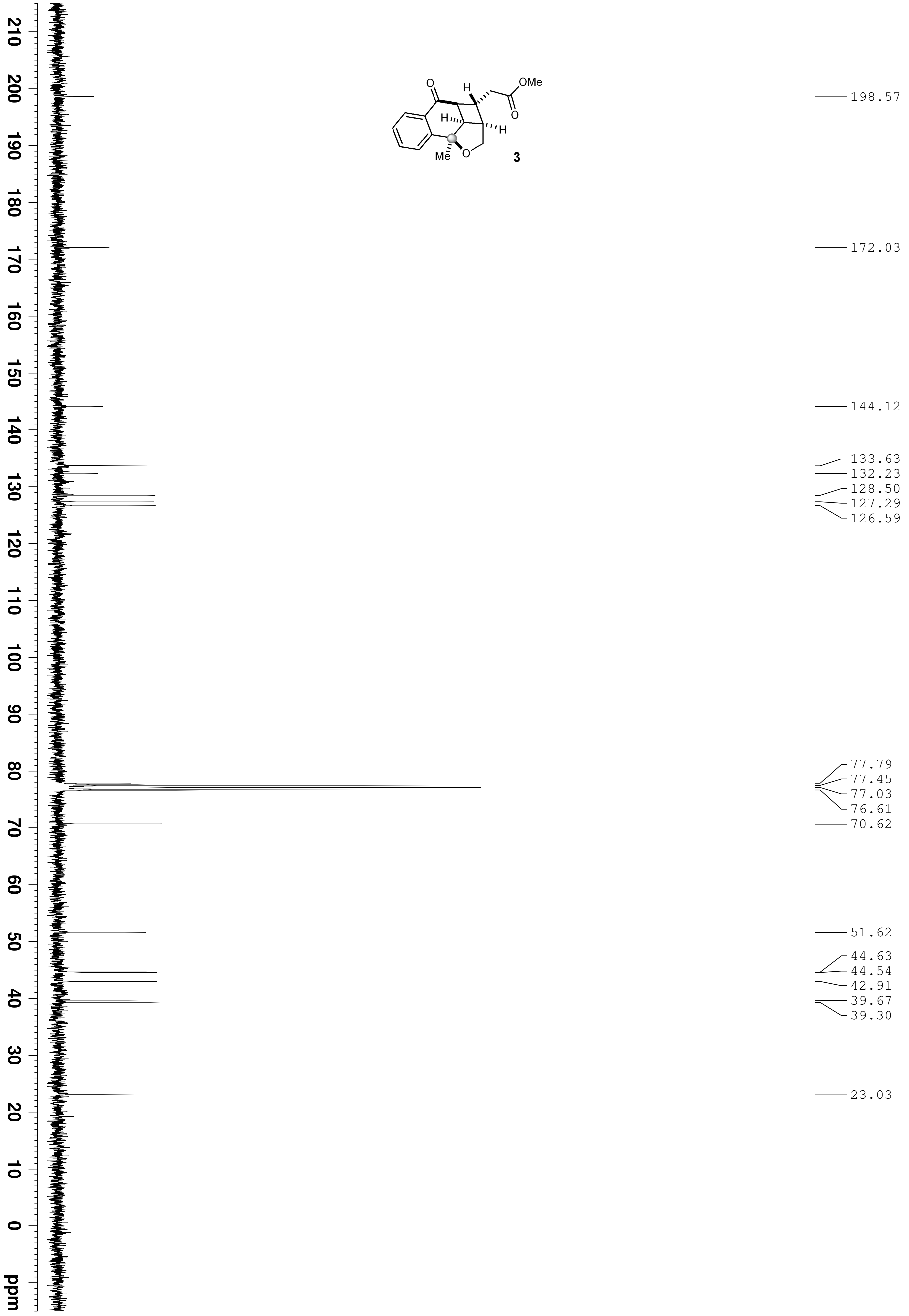

Supplementary Figure 108. ^{1}H NMR spectrum of compound **3**

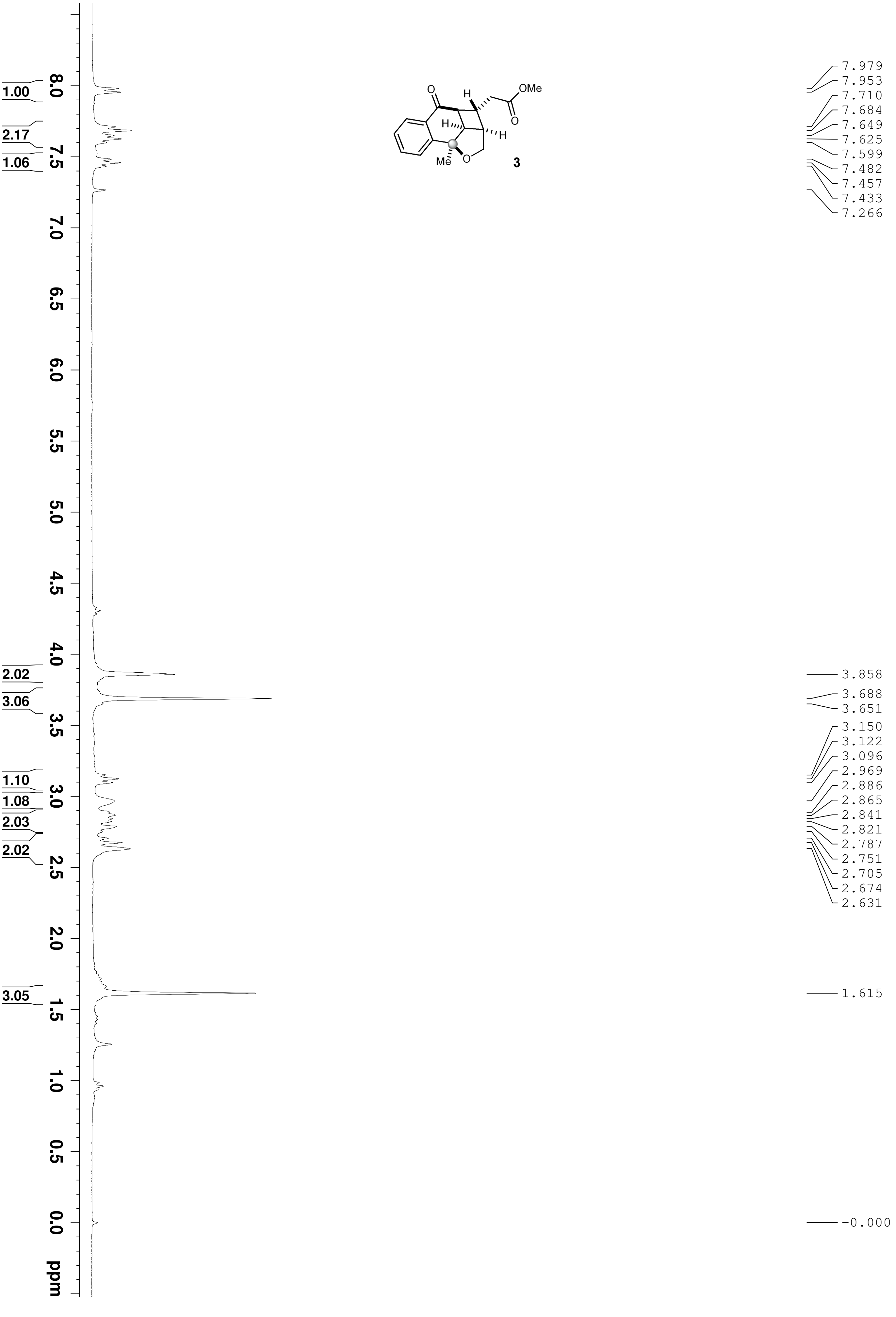

Supplementary Figure 109. ^{13}C NMR spectrum of compound **4**

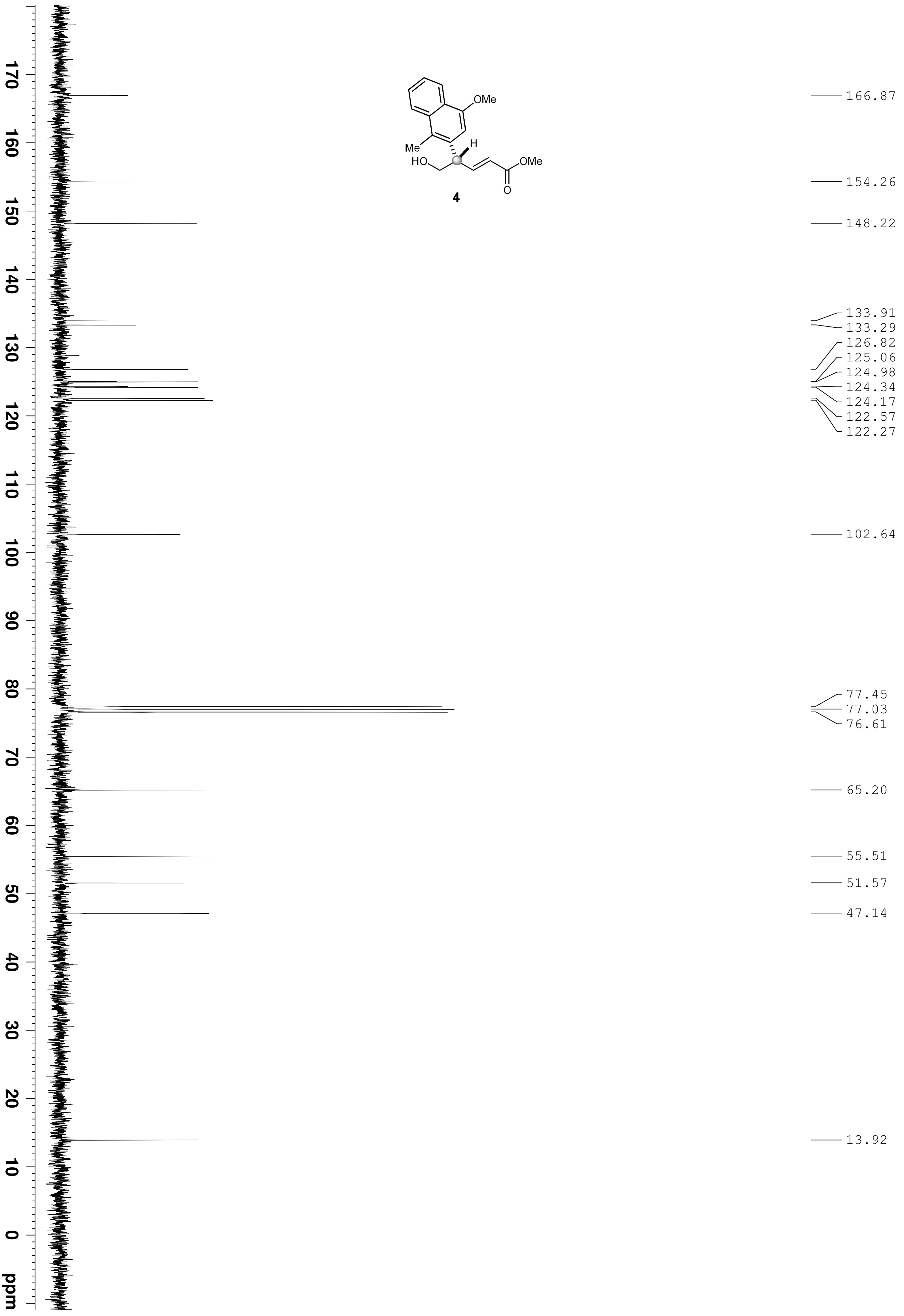

Supplementary Figure 110. ^{1}H NMR spectrum of compound **4**

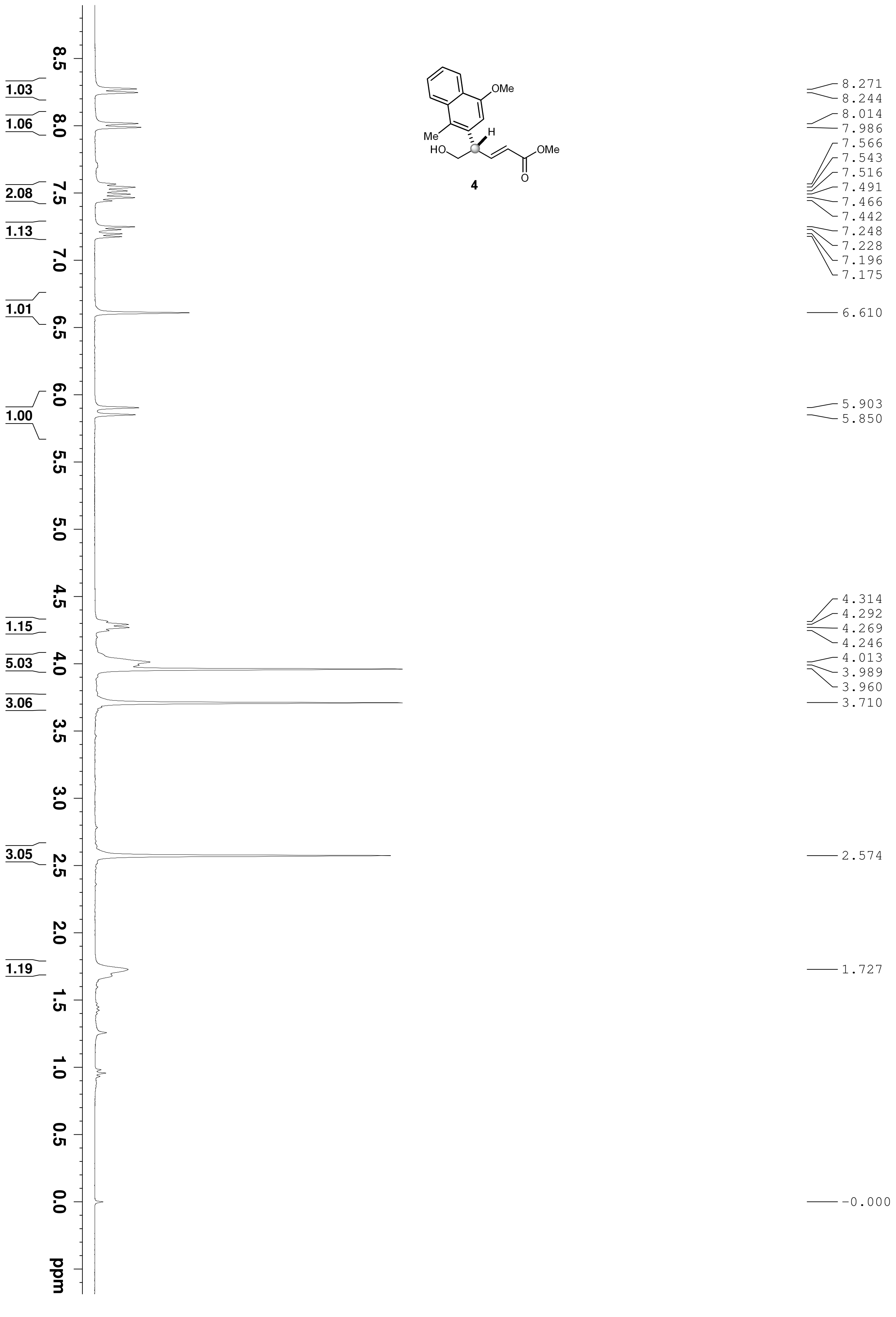

Supplementary Figure 111. ^{13}C NMR spectrum of compound **7**

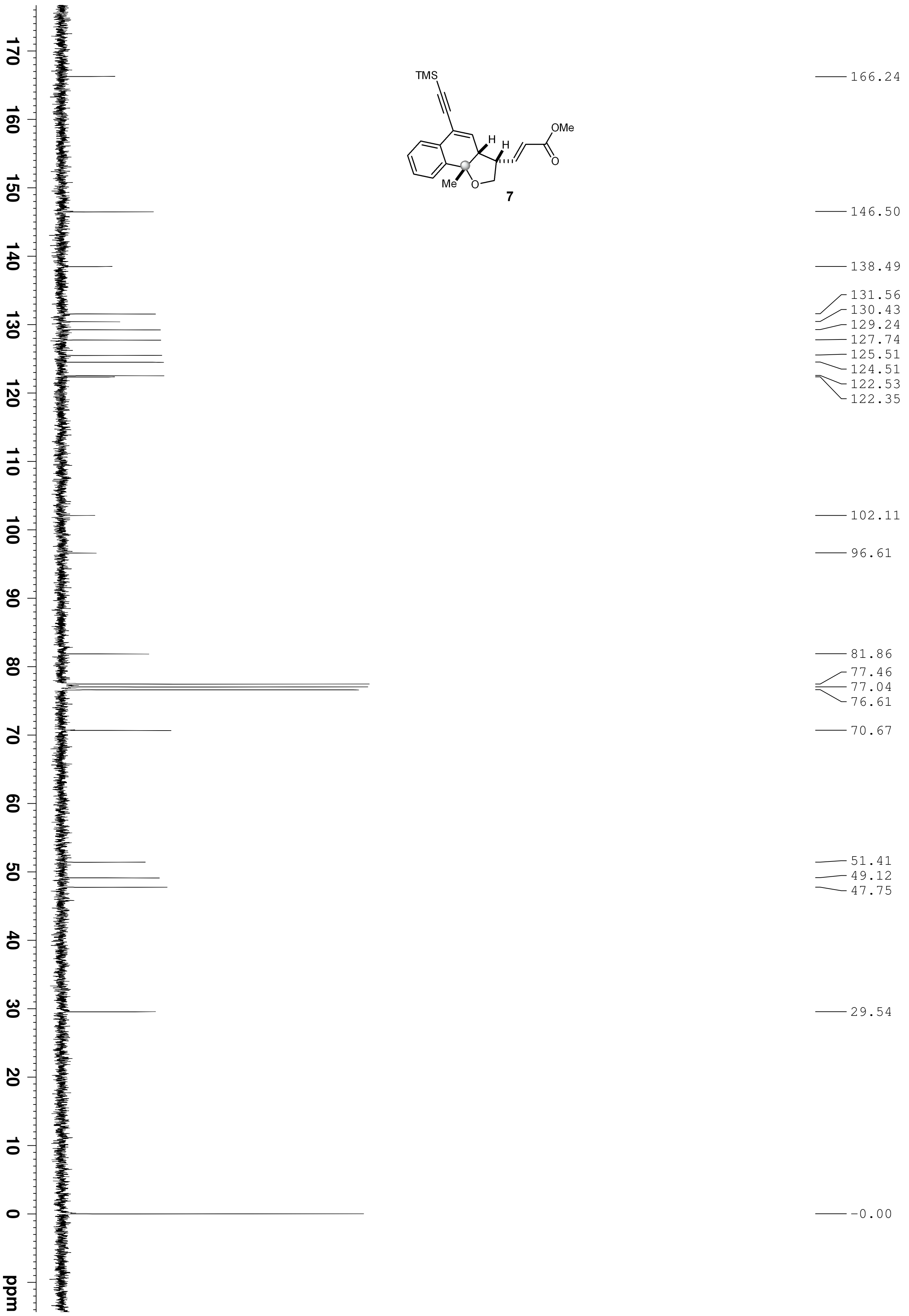

Supplementary Figure 112. ^{1}H NMR spectrum of compound **7**

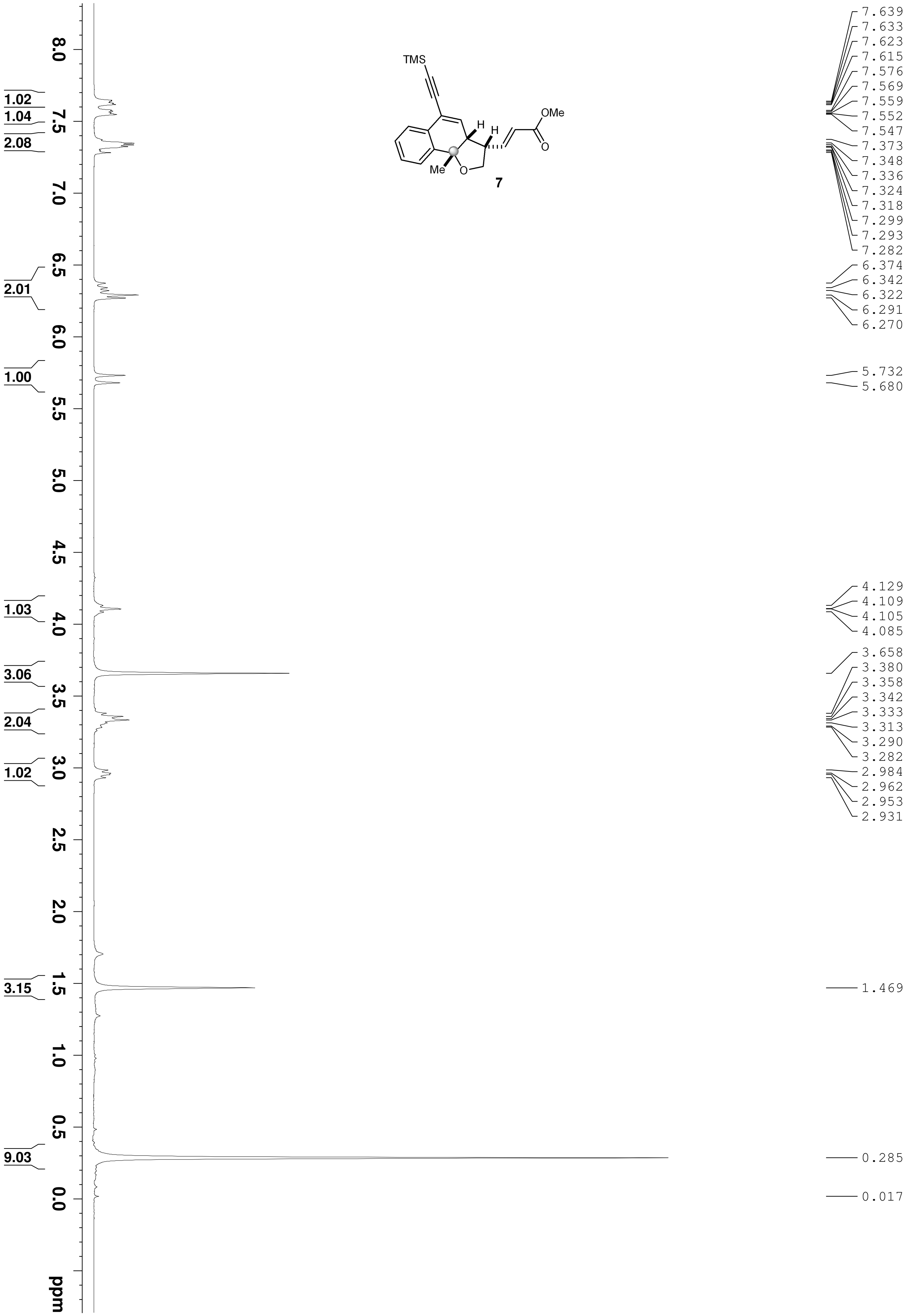

Supplementary Figure 113. ^{13}C NMR spectrum of compound **8**

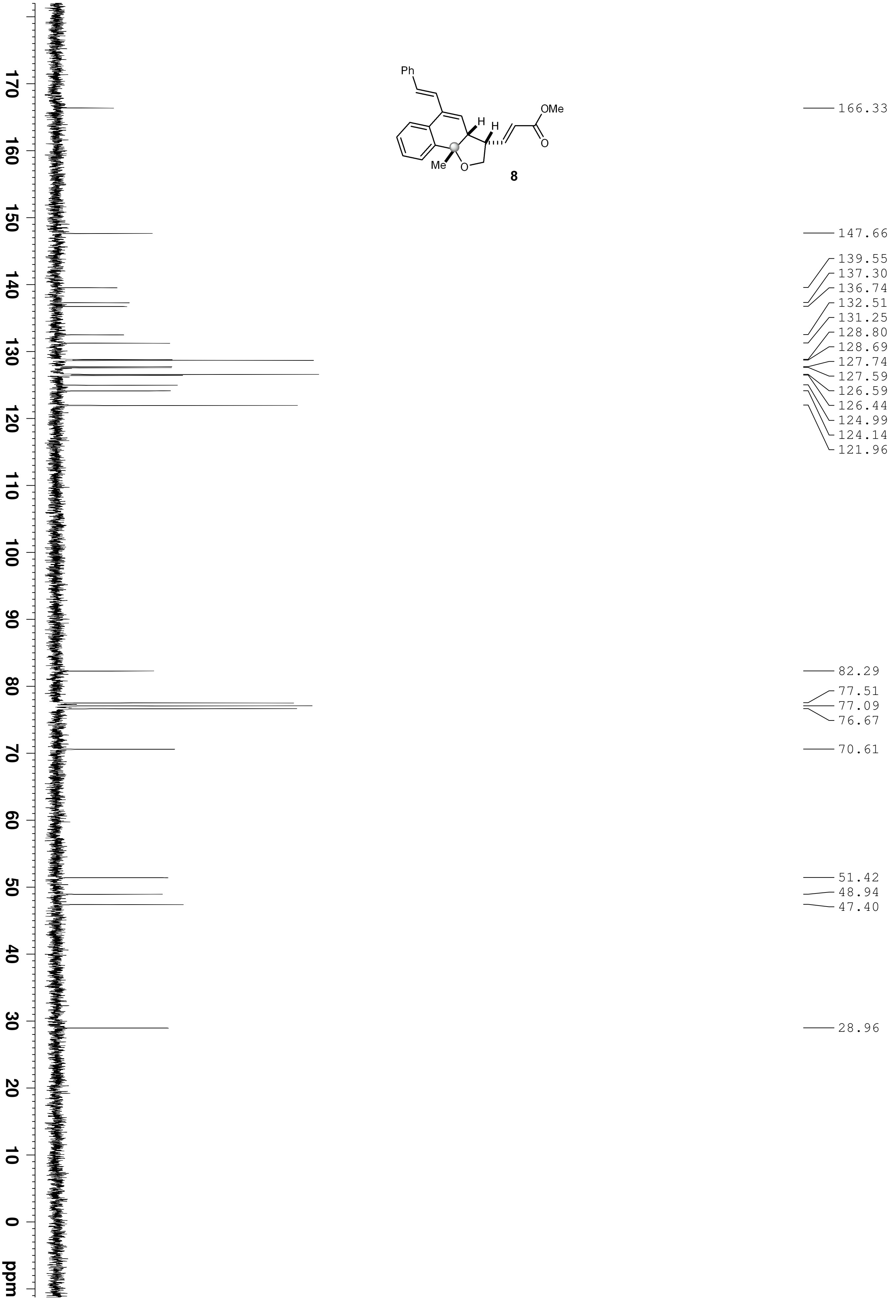

Supplementary Figure 114. ^{1}H NMR spectrum of compound **8**

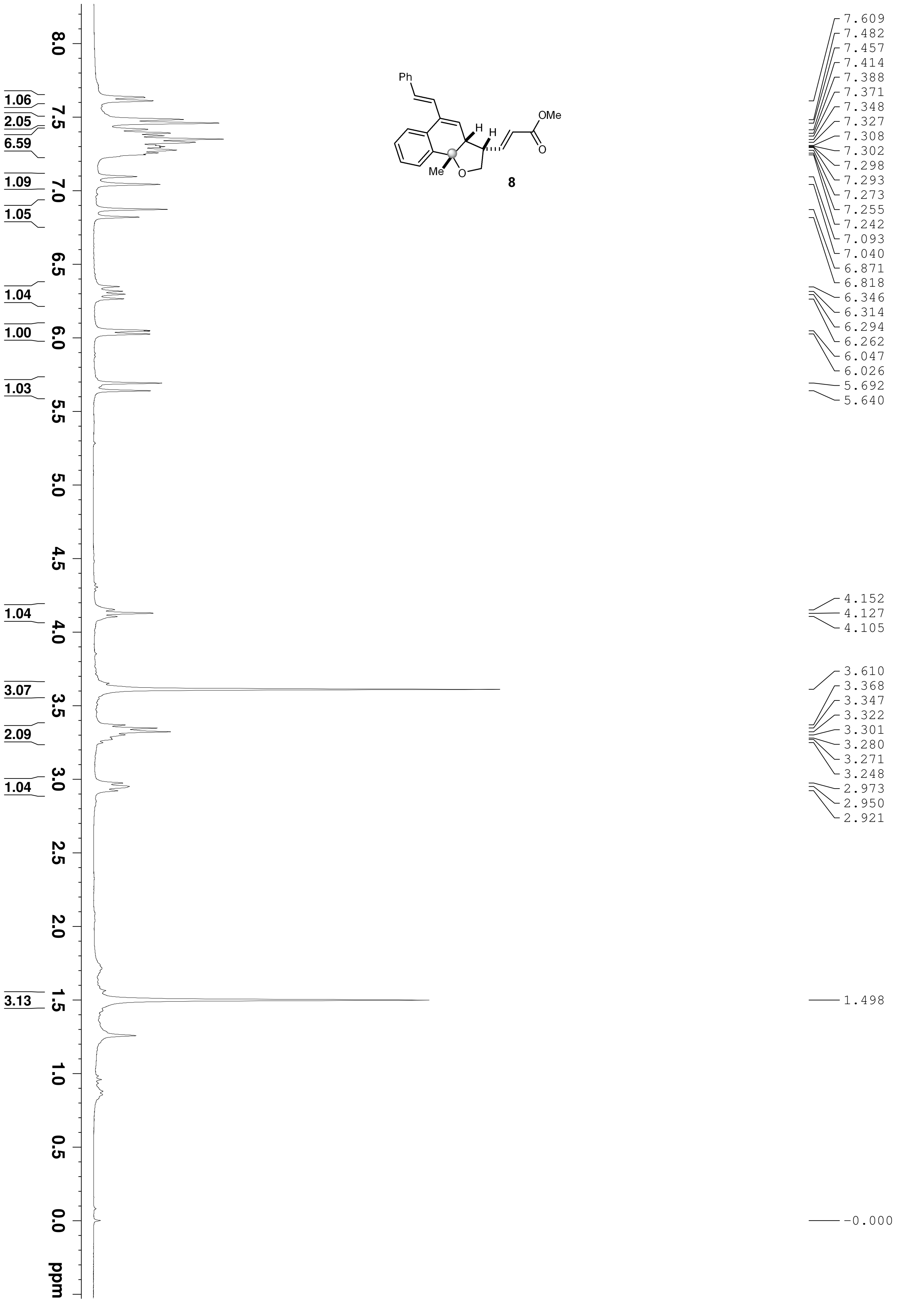

Supplementary Figure 115. ^{13}C NMR spectrum of compound **10**

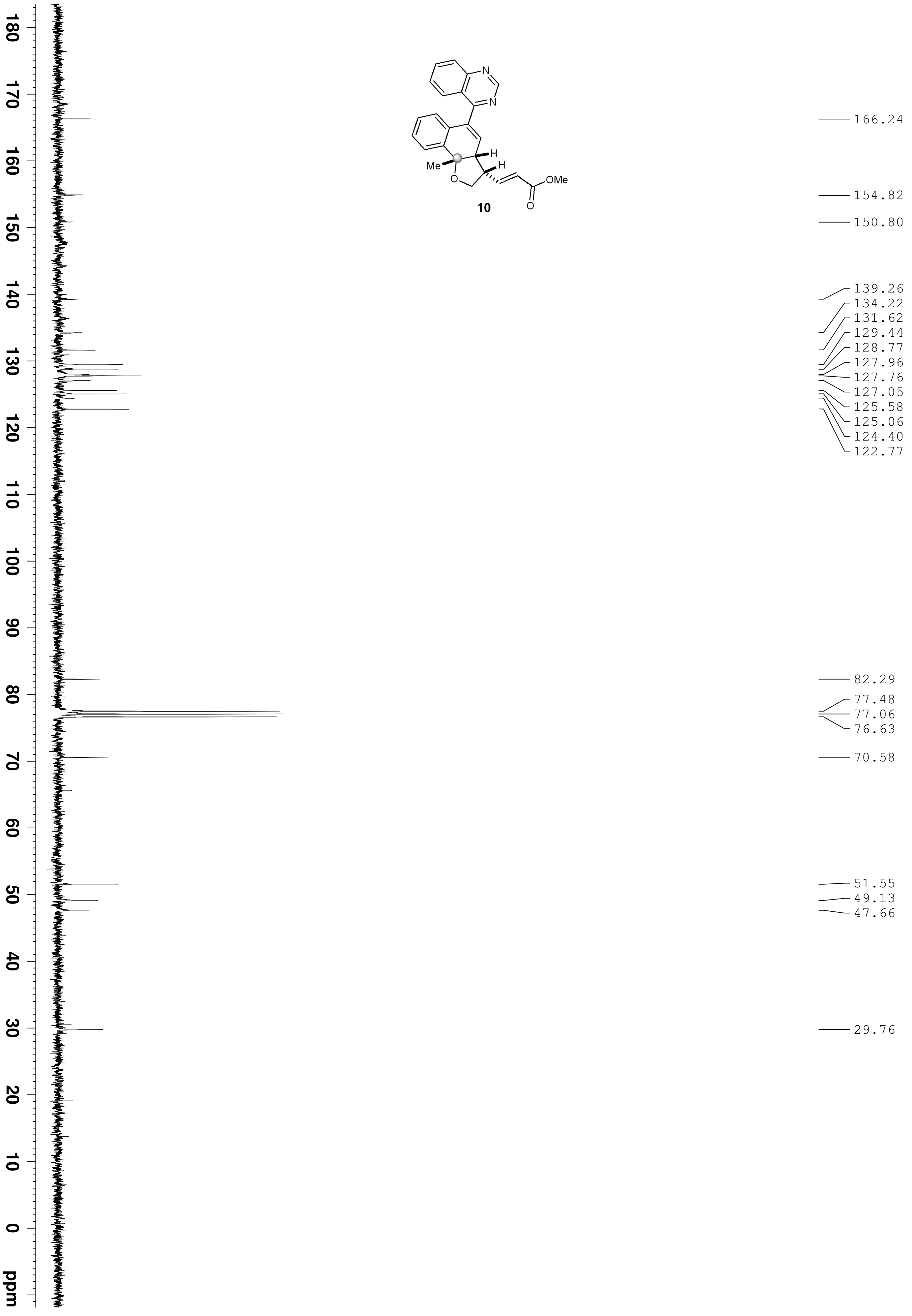

Supplementary Figure 116. ^{1}H NMR spectrum of compound **10**

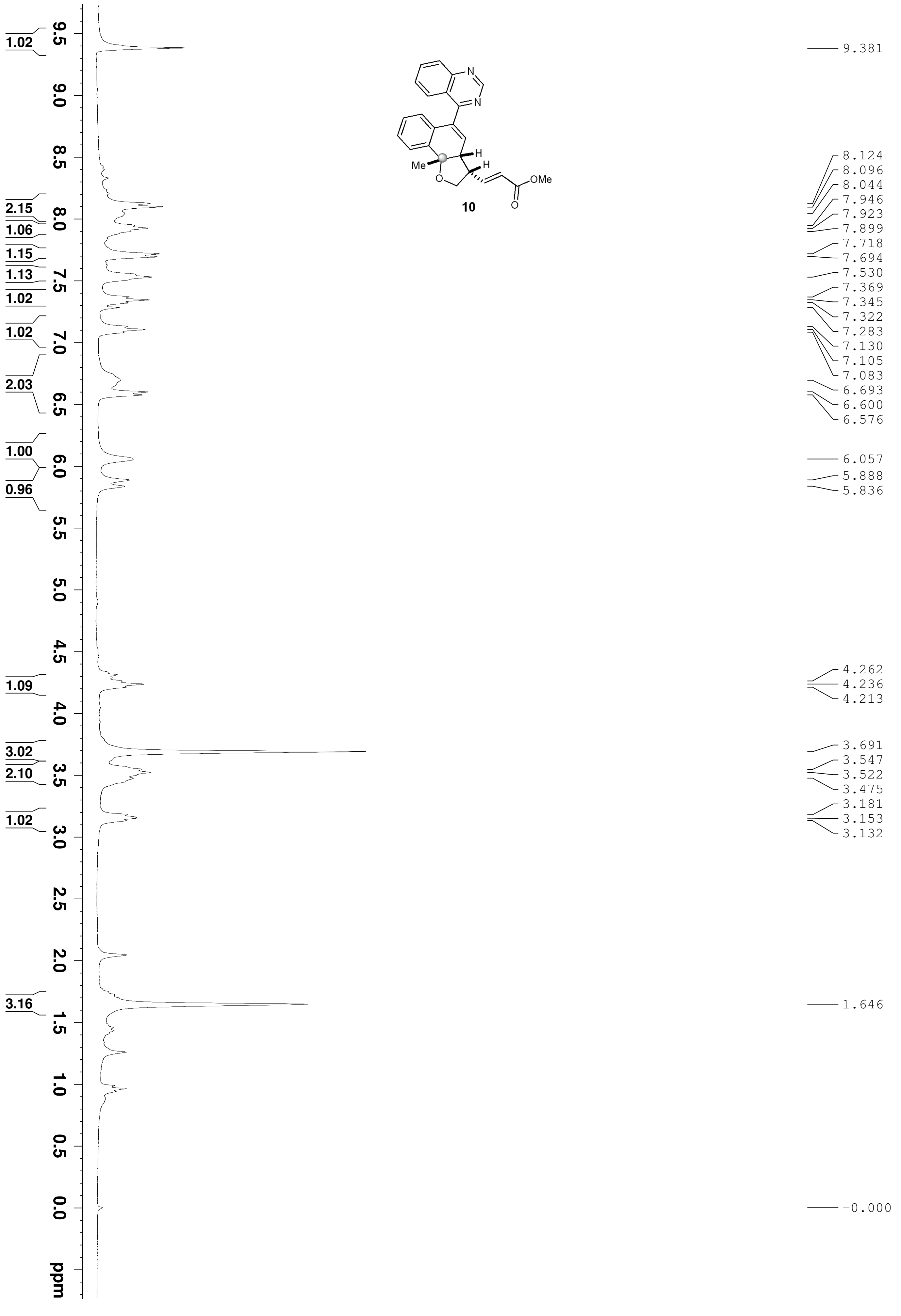

Supplementary Figure 117. ^{13}C NMR spectrum of compound **L10**

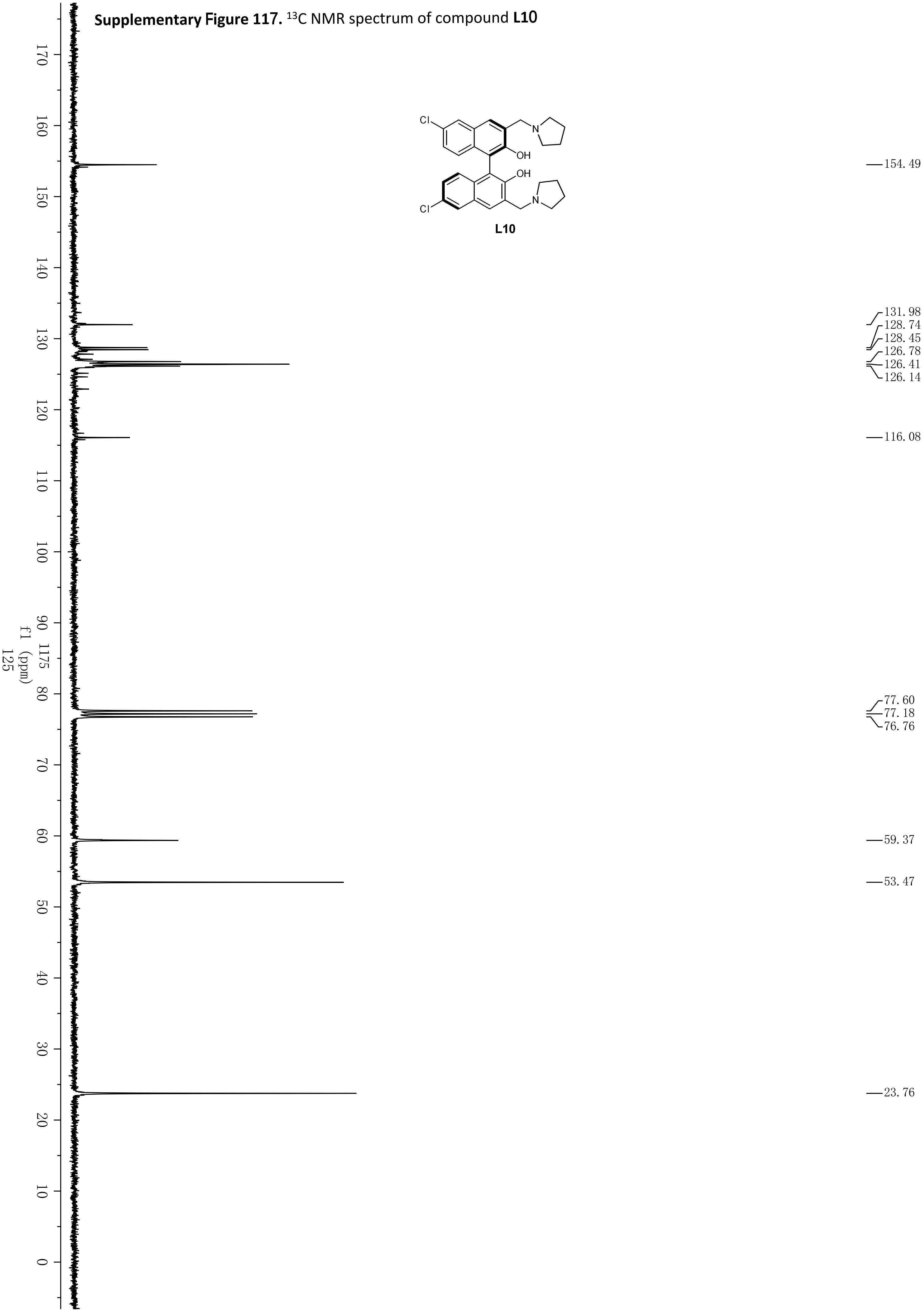

Supplementary Figure 118. ^{1}H NMR spectrum of compound **L10**

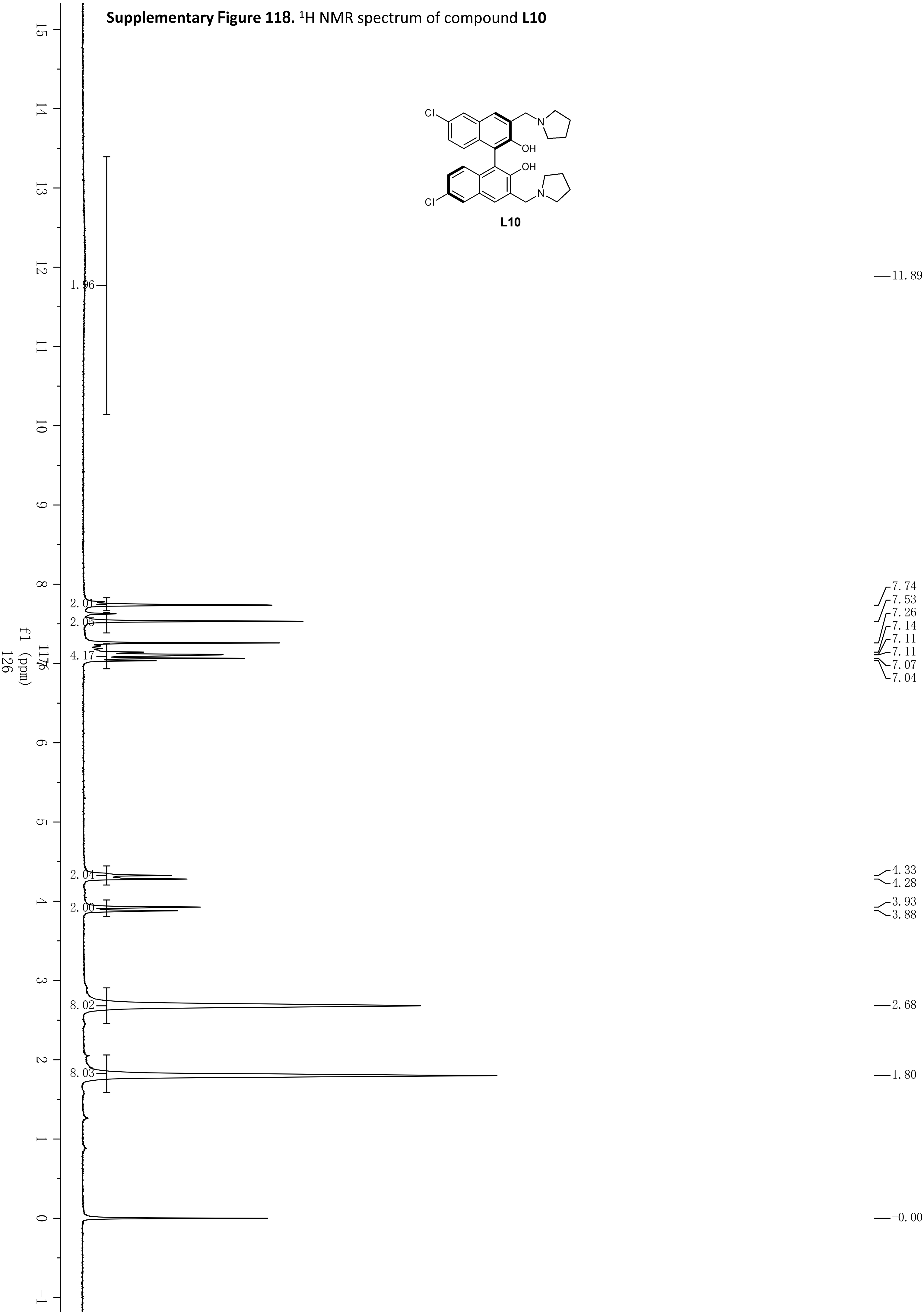

HPLC RESULTS

Supplementary Figure 119. HPLC chromatogram for compound **1a***

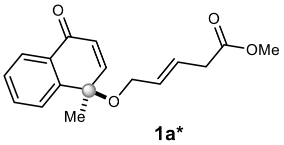

1a*

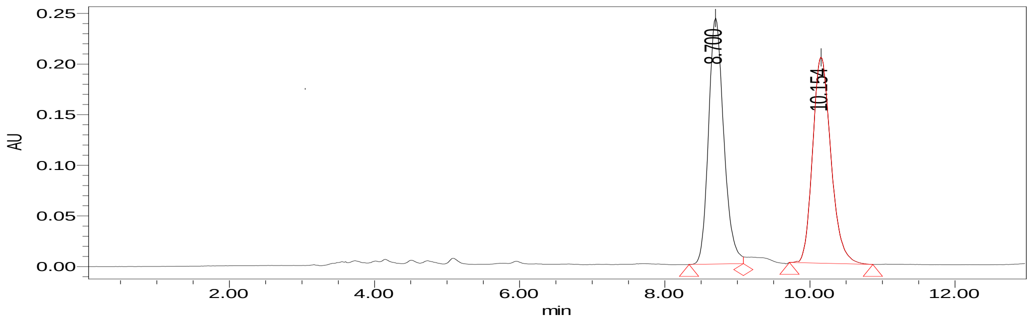

	Retention Time	Area	%Area	Height
1	8.700	3557400	49.97	243068
2	10.154	3561917	50.03	203597

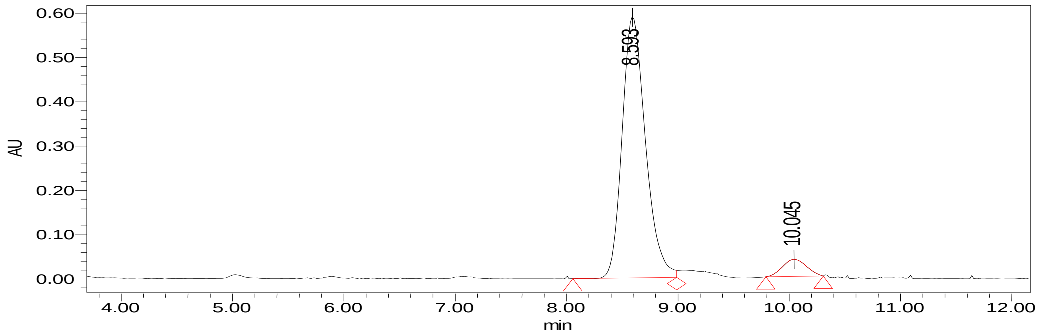

	Retention Time	Area	%Area	Height
1	8.593	8585602	93.74	590156
2	10.045	572940	6.26	38430

Supplementary Figure 120. HPLC chromatogram for compound **2a**

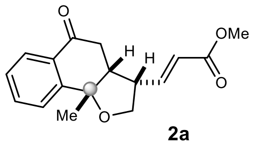

2a

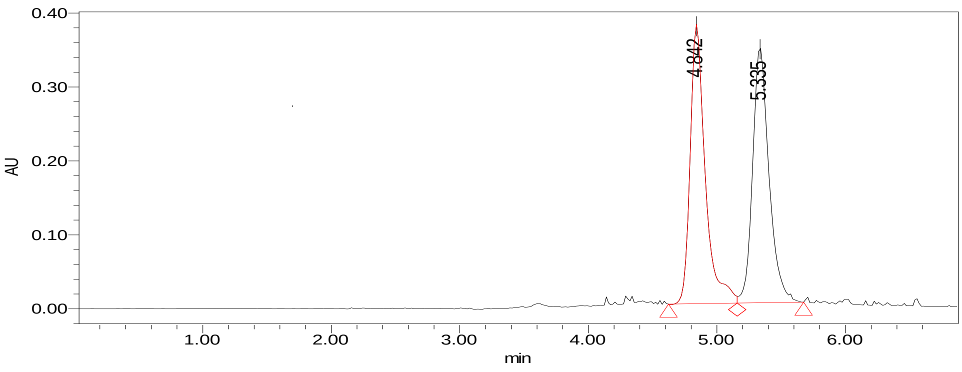

	Retention Time	Area	%Area	Height
1	4.842	3079438	50.08	375894
2	5.335	3069020	49.92	344423

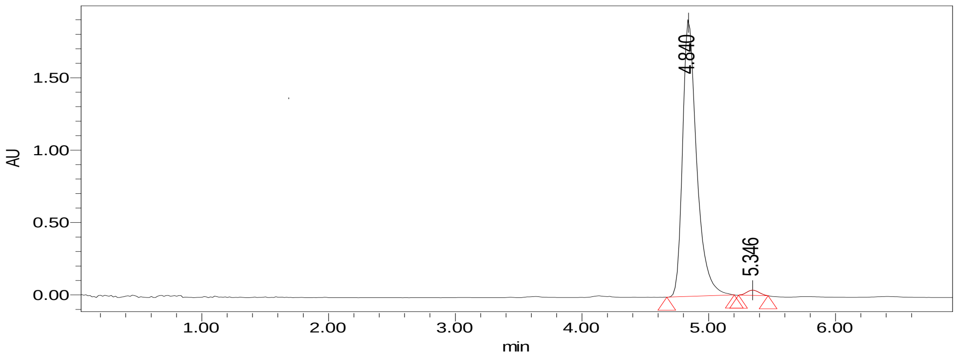

	Retention Time	Area	%Area	Height
1	4.840	14744406	98.35	1915421
2	5.346	247621	1.65	36536

Supplementary Figure 121. HPLC chromatogram for compound **2a'**

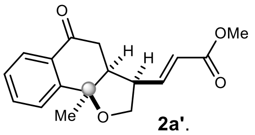

2a'.

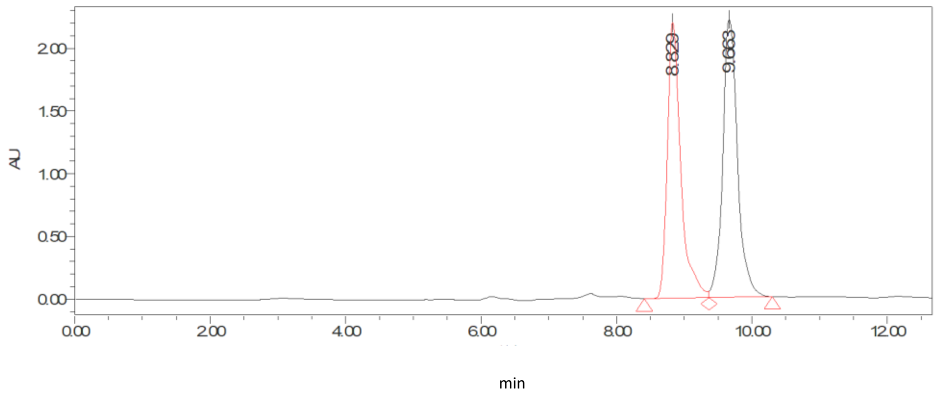

	Retention Time	Area	%Area	Height
1	8.829	30373580	47.48	2195552
2	9.663	33596673	52.52	2214052

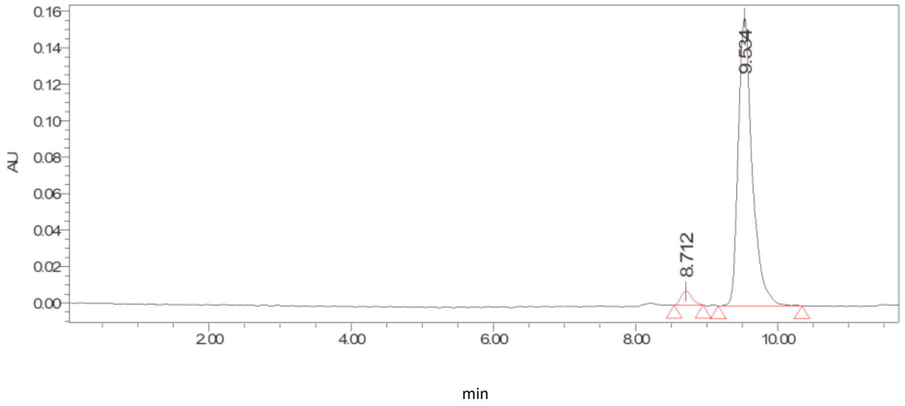

	Retention Time	Area	%Area	Height
1	8.712	82646	3.69	7578
2	9.534	2157858	96.31	157949

Supplementary Figure 122. HPLC chromatogram for compound **1b***

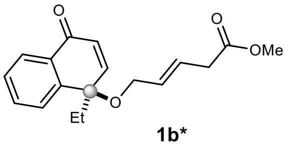

1b*

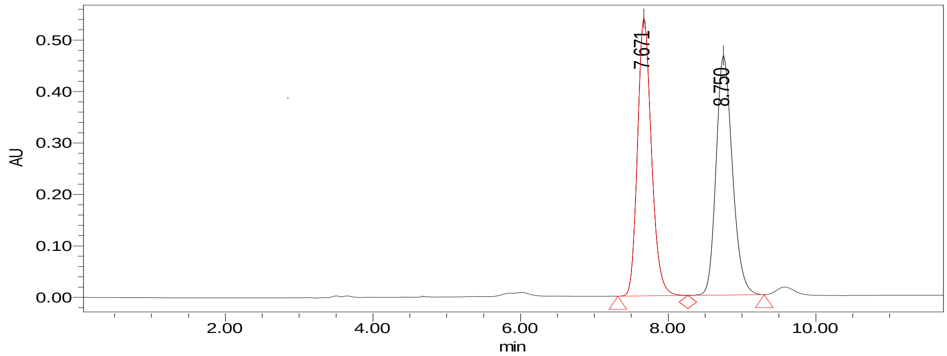

	Retention Time	Area	%Area	Height
1	7.671	7091653	49.85	540663
2	8.750	7133626	50.15	465504

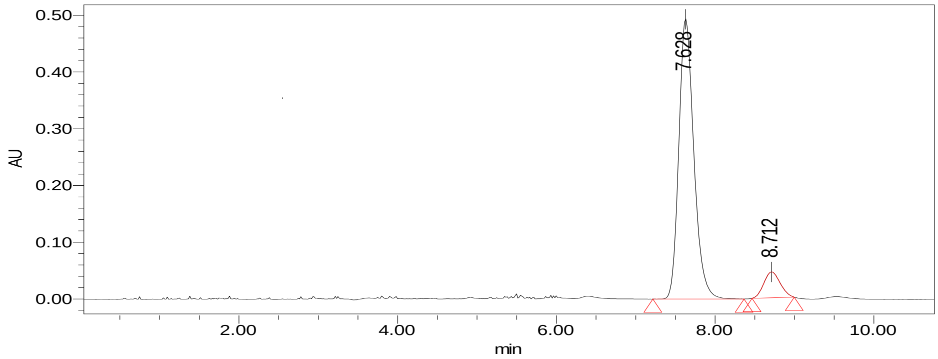

	Retention Time	Area	%Area	Height
1	7.628	6498486	90.86	493543
2	8.712	653801	9.14	45726

Supplementary Figure 123. HPLC chromatogram for compound **2b**

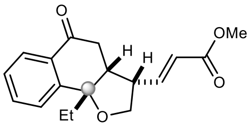

2b

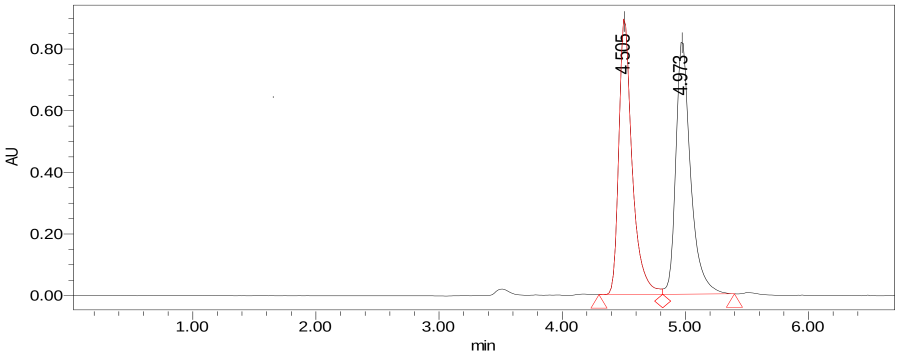

	Retention Time	Area	%Area	Height
1	4.505	6731594	49.64	897518
2	4.973	6828392	50.36	824697

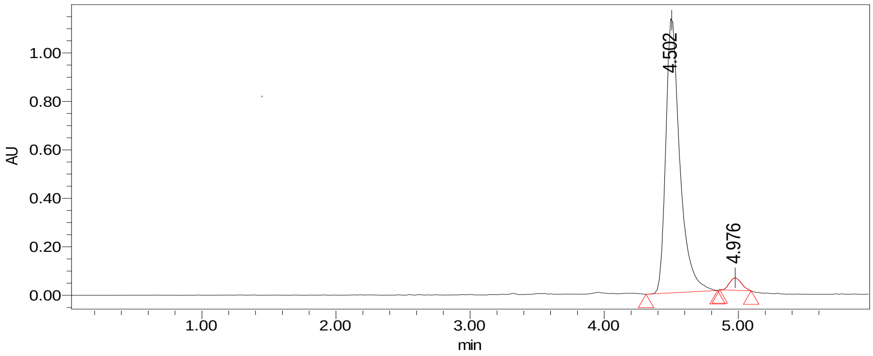

	Retention Time	Area	%Area	Height
1	4.502	8295636	96.29	1140556
2	4.976	319232	3.71	51430

Supplementary Figure 124. HPLC chromatogram for compound **1c***

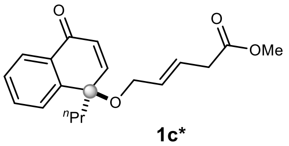

1c*

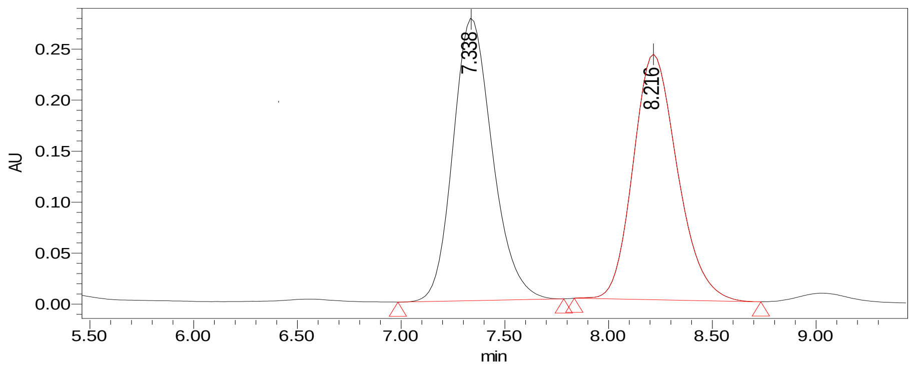

	Retention Time	Area	%Area	Height
1	7.338	3642836	50.43	276952
2	8.216	3580876	49.57	240600

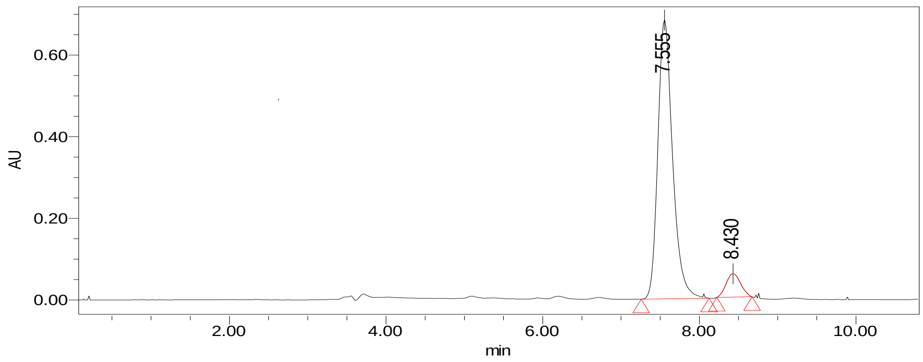

	Retention Time	Area	%Area	Height
1	7.555	8934323	92.32	683950
2	8.430	743224	7.68	57651

Supplementary Figure 125. HPLC chromatogram for compound **2c**

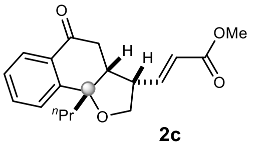

2c

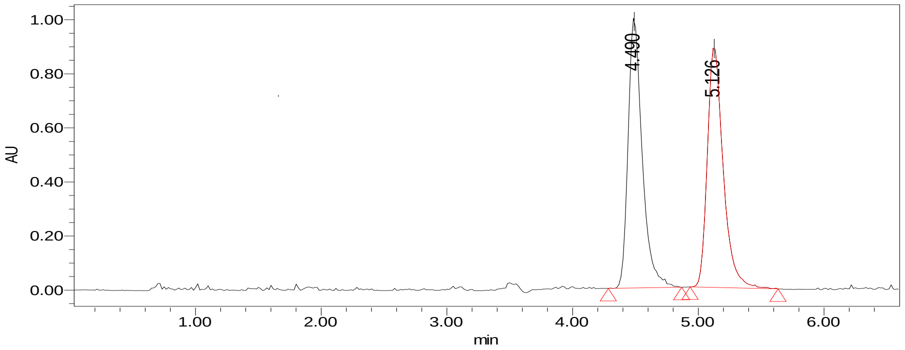

	Retention Time	Area	%Area	Height
1	4.490	7790881	50.00	999072
2	5.126	7789361	50.00	894337

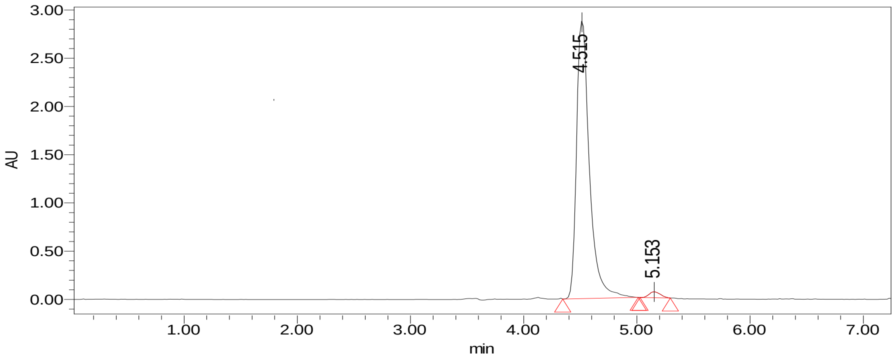

	Retention Time	Area	%Area	Height
1	4.515	22854932	98.13	2910972
2	5.153	435238	1.87	61949

Supplementary Figure 126. HPLC chromatogram for compound **1d***

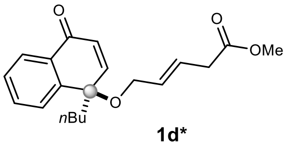

1d*

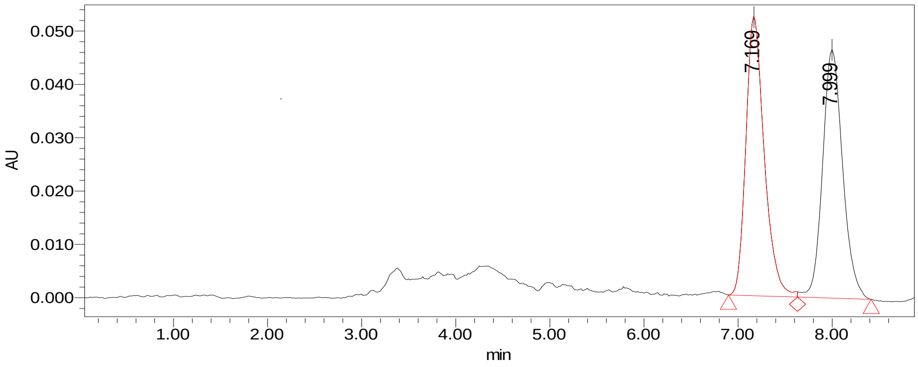

	Retention Time	Area	%Area	Height
1	7.169	713247	50.78	52428
2	7.999	691301	49.22	46576

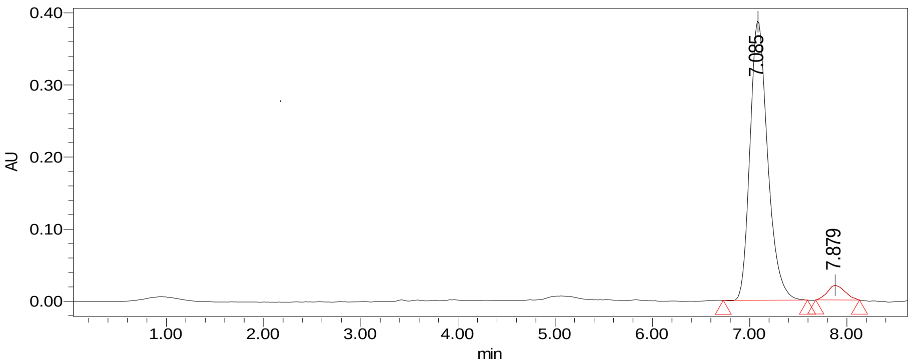

	Retention Time	Area	%Area	Height
1	7.085	4916226	95.12	388288
2	7.879	251964	4.88	20137

Supplementary Figure 127. HPLC chromatogram for compound **2d**

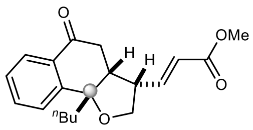

2d

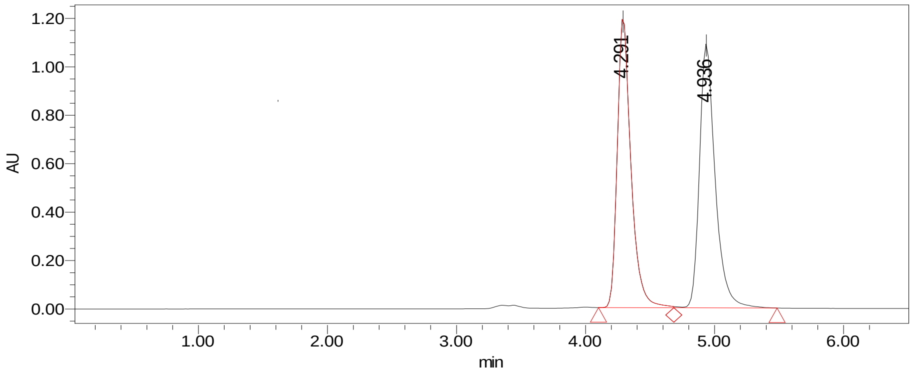

	Retention Time	Area	%Area	Height
1	4.291	8833360	49.01	1196989
2	4.936	9191643	50.99	1087706

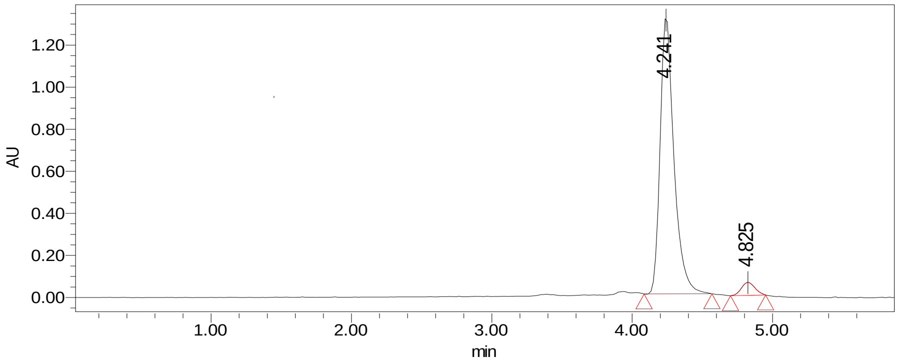

	Retention Time	Area	%Area	Height
1	4.241	9021166	95.61	1317557
2	4.825	414211	4.39	61656

Supplementary Figure 128. HPLC chromatogram for compound **1e***

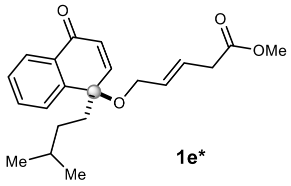

1e*

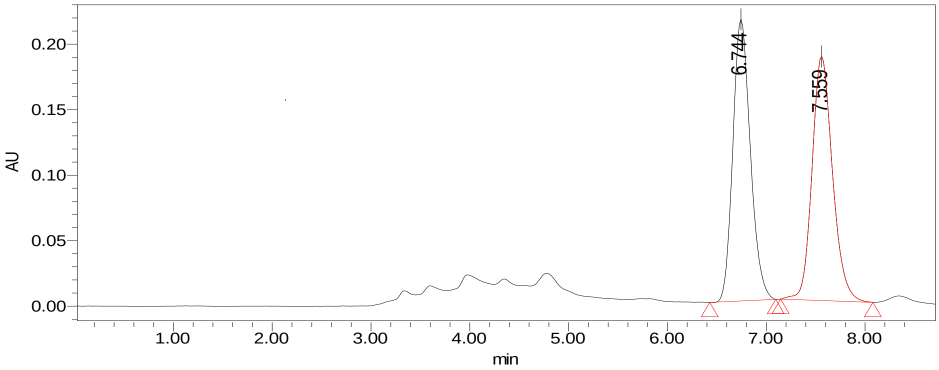

	Retention Time	Area	%Area	Height
1	6.744	2645782	49.62	214909
2	7.559	2686575	50.38	186507

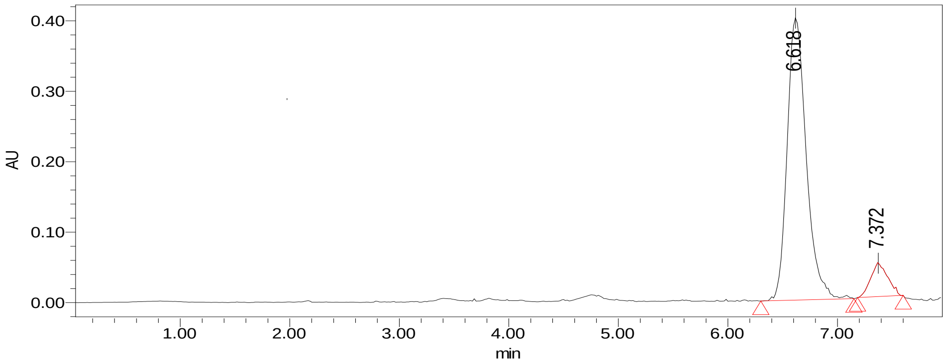

	Retention Time	Area	%Area	Height
1	6.618	4867130	90.44	399923
2	7.372	514477	9.56	47248

Supplementary Figure 129. HPLC chromatogram for compound **2e**

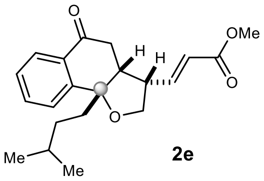

2e

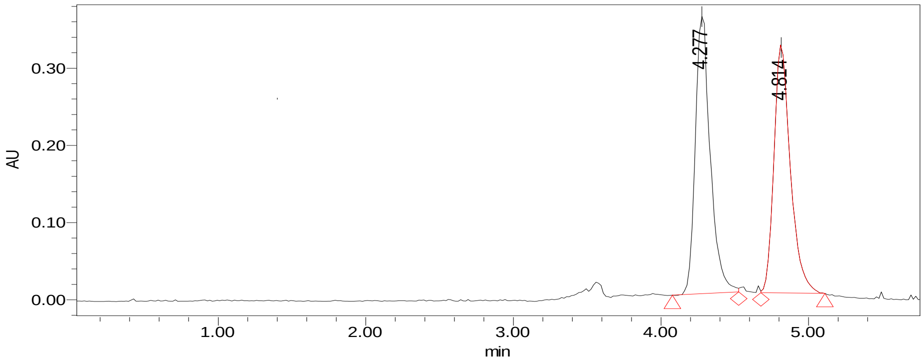

	Retention Time	Area	%Area	Height
1	4.277	2546869	50.54	365016
2	4.814	2491976	49.46	321017

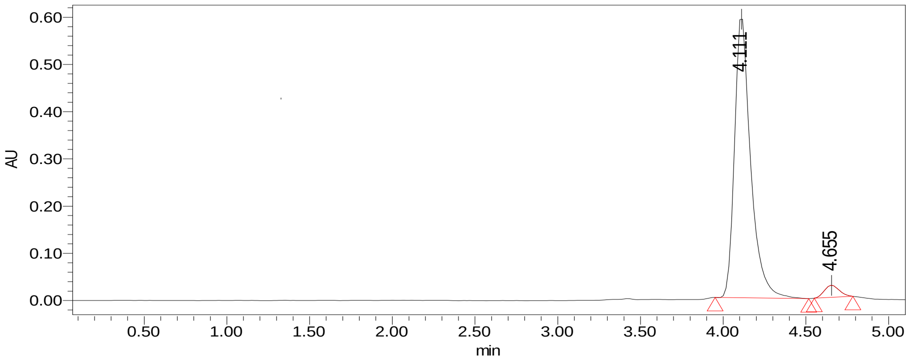

	Retention Time	Area	%Area	Height
1	4.111	4035108	96.16	591708
2	4.655	161076	3.84	25632

Supplementary Figure 130. HPLC chromatogram for compound **1f***

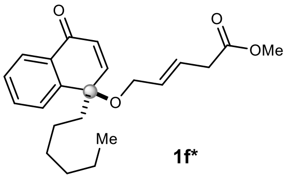

1f*

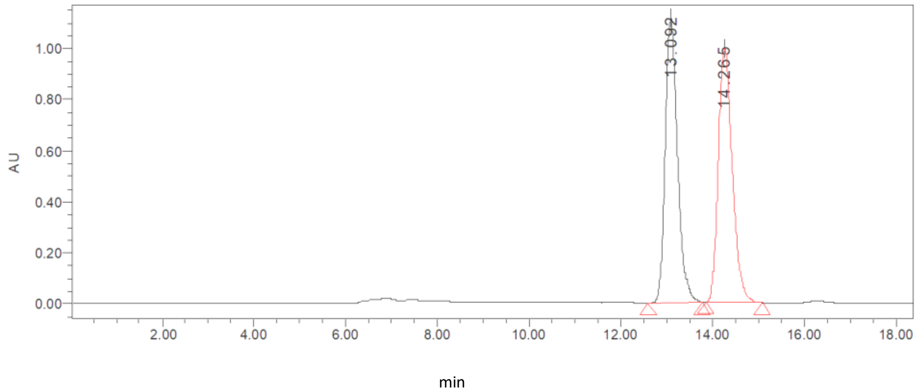

	Retention Time	Area	%Area	Height
1	13.092	21195061	50.26	1115650
2	14.265	20973878	49.74	993582

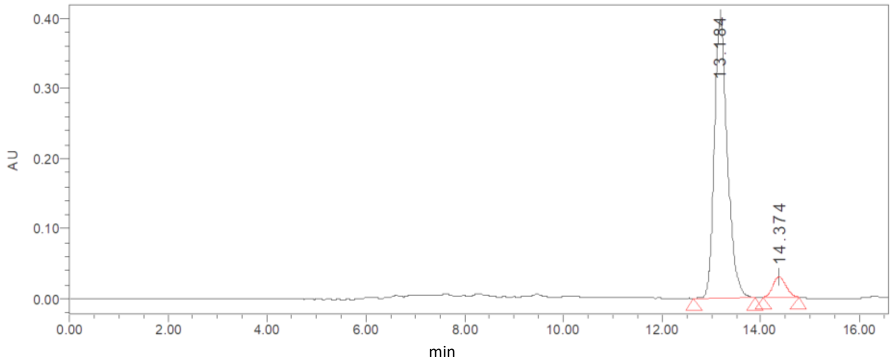

	Retention Time	Area	%Area	Height
1	13.184	7387462	93.03	398268
2	14.374	553539	6.97	29579

Supplementary Figure 131. HPLC chromatogram for compound **2f**

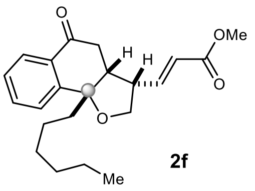

2f

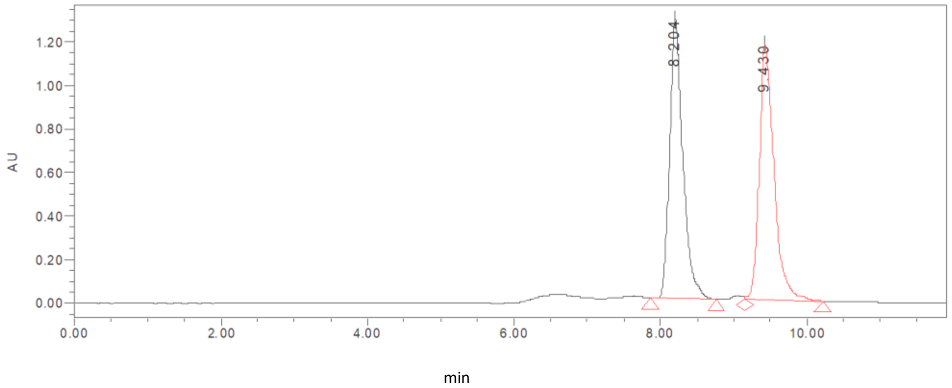

	Retention Time	Area	%Area	Height
1	8.204	16203823	49.64	1284712
2	9.430	16437605	50.36	1172121

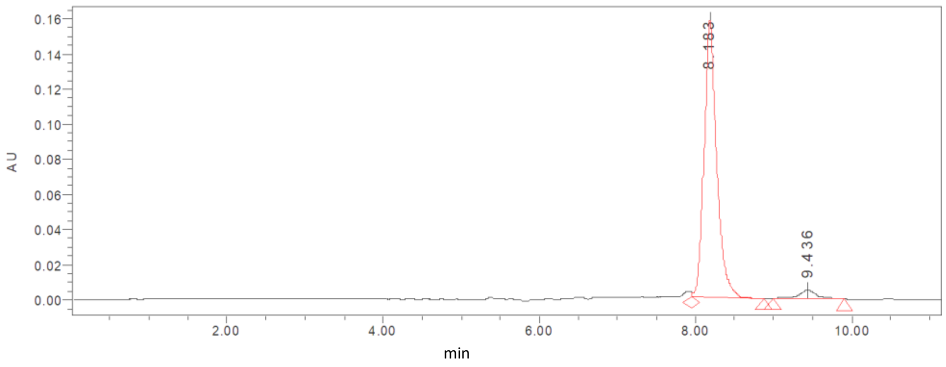

	Retention Time	Area	%Area	Height
1	8.183	1770373	95.67	157198
2	9.436	80154	4.33	4786

Supplementary Figure 132. HPLC chromatogram for compound **1g***

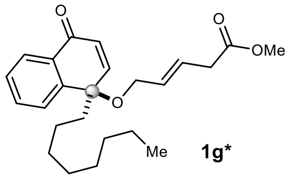

1g*

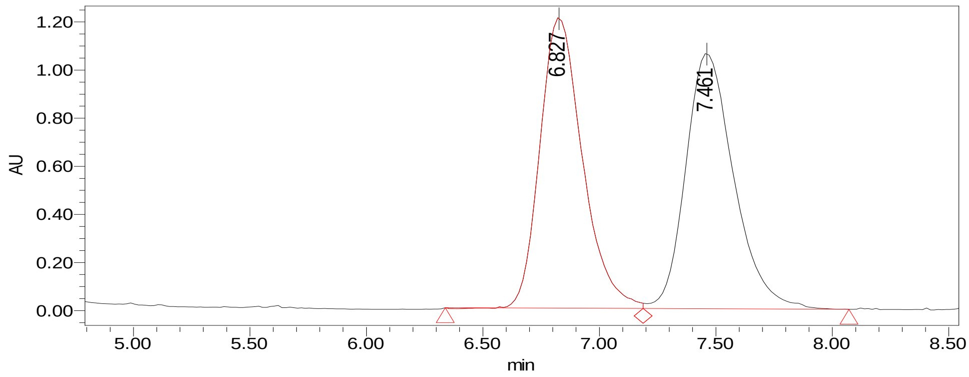

	Retention Time	Area	%Area	Height
1	6.827	15364965	50.15	1206836
2	7.461	15271013	49.85	1062911

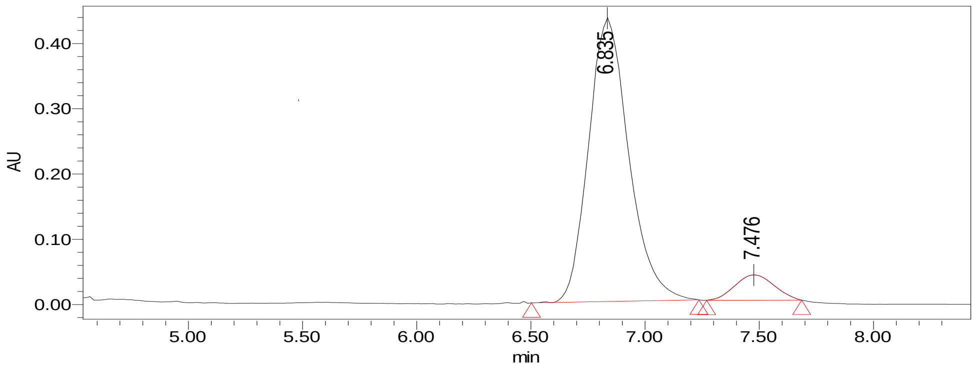

	Retention Time	Area	%Area	Height
1	6.835	5323365	92.00	431176
2	7.476	462725	8.00	38922

Supplementary Figure 133. HPLC chromatogram for compound **2g**

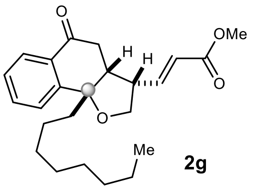

2g

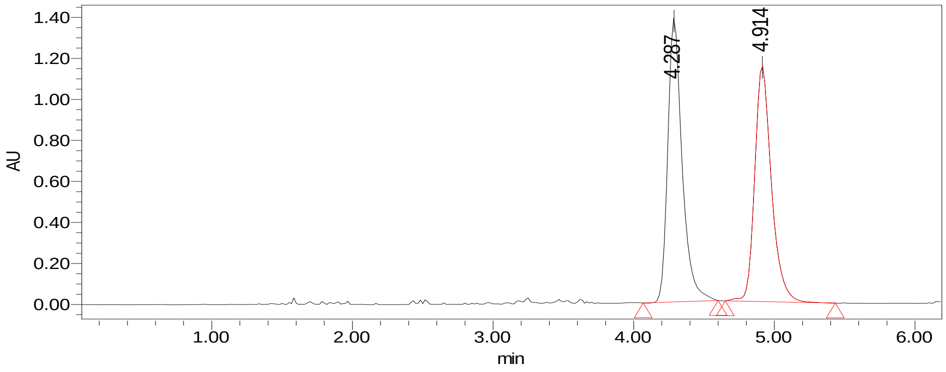

	Retention Time	Area	%Area	Height
1	4.287	9808883	50.83	1379102
2	4.914	9486730	49.17	1146514

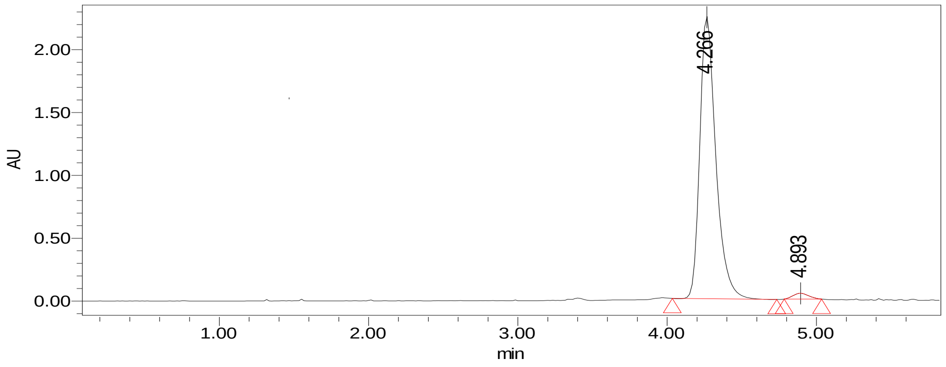

	Retention Time	Area	%Area	Height
1	4.266	16839910	98.06	2243091
2	4.893	333745	1.94	46055

Supplementary Figure 134. HPLC chromatogram for compound **1h***

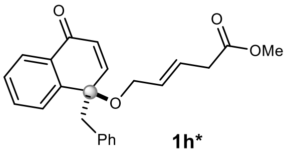

1h*

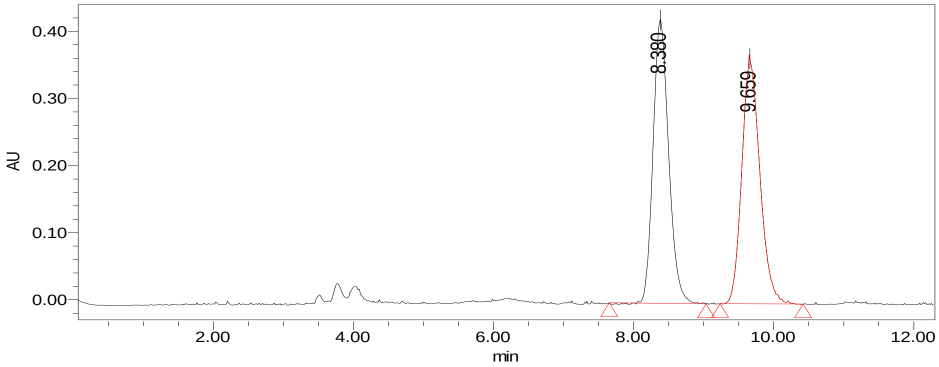

	Retention Time	Area	%Area	Height
1	8.380	6568129	49.98	422081
2	9.659	6573045	50.02	365365

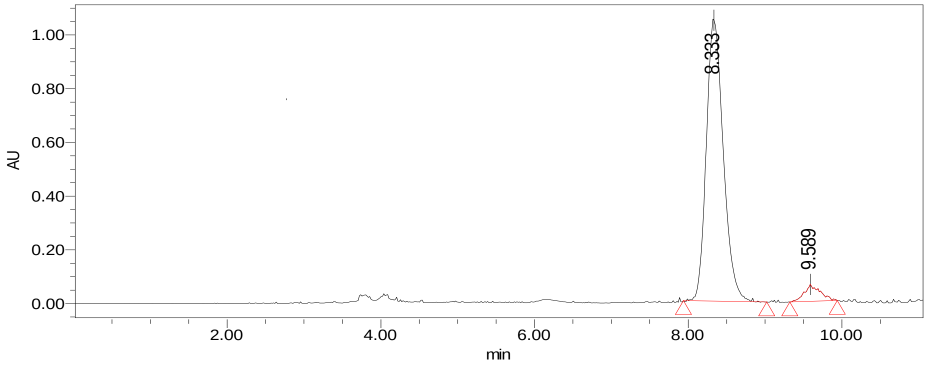

	Retention Time	Area	%Area	Height
1	8.333	16122899	94.74	1050753
2	9.589	894316	5.26	59862

Supplementary Figure 135. HPLC chromatogram for compound **2h**

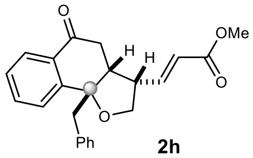

2h

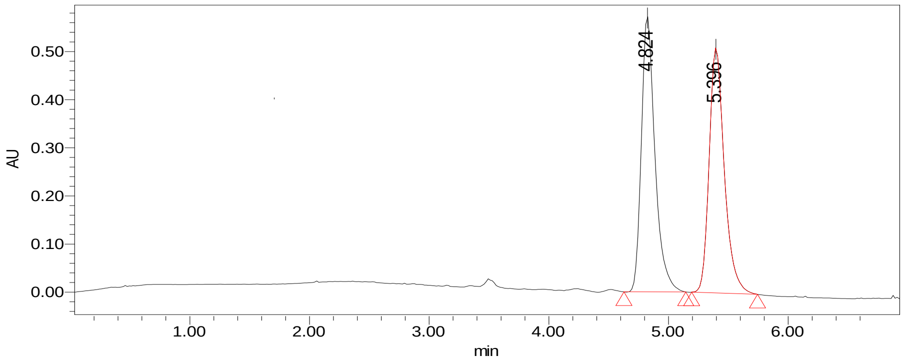

	Retention Time	Area	%Area	Height
1	4.824	4527355	50.24	570810
2	5.396	4484031	49.76	508664

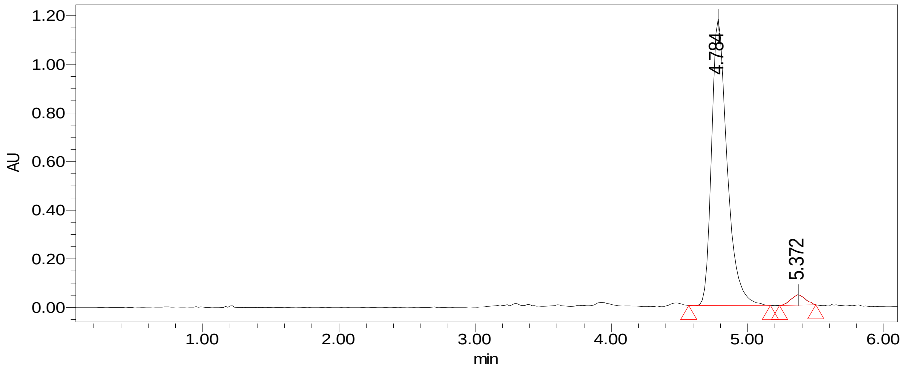

	Retention Time	Area	%Area	Height
1	4.784	9331534	96.75	1174195
2	5.372	313358	3.25	41725

Supplementary Figure 136. HPLC chromatogram for compound **1i***

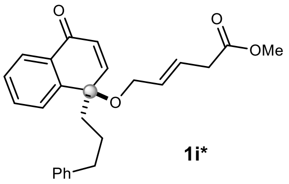

1i*

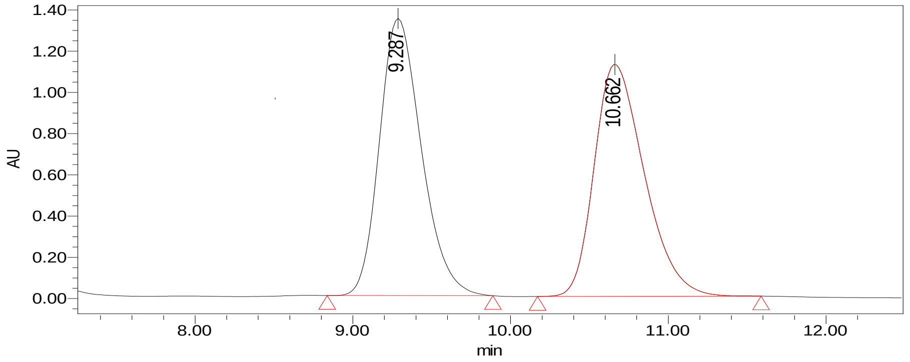

	Retention Time	Area	%Area	Height
1	9.287	24846069	50.06	1344285
2	10.662	24782130	49.94	1126237

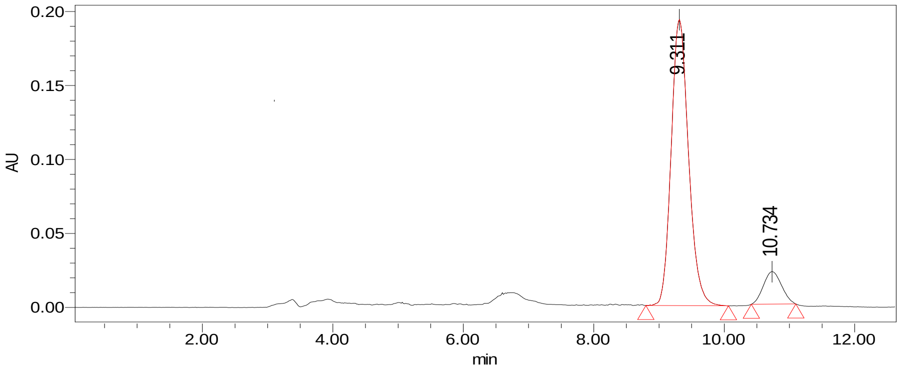

	Retention Time	Area	%Area	Height
1	9.311	3577781	89.40	193334
2	10.734	424315	10.60	21974

Supplementary Figure 137. HPLC chromatogram for compound **2i**

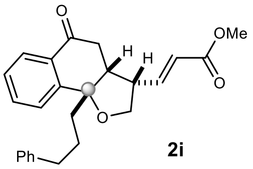

2i

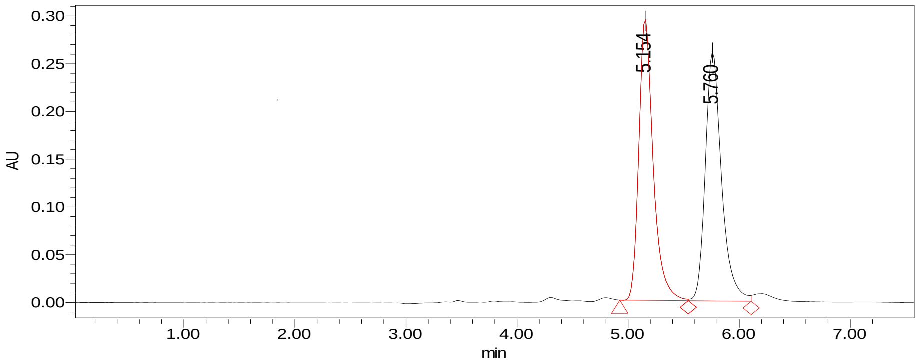

	Retention Time	Area	%Area	Height
1	5.154	2645086	49.91	294214
2	5.760	2654807	50.09	260969

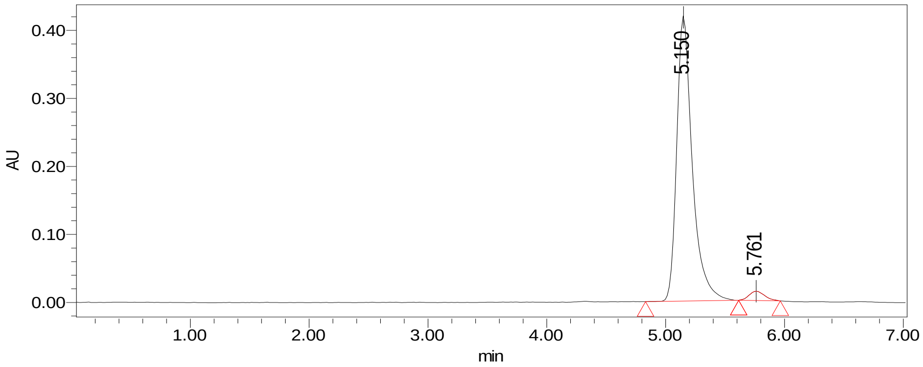

	Retention Time	Area	%Area	Height
1	5.150	3811964	96.82	418493
2	5.761	125168	3.18	13742

Supplementary Figure 138. HPLC chromatogram for compound **1j***

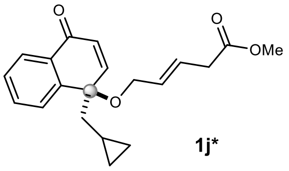

1j*

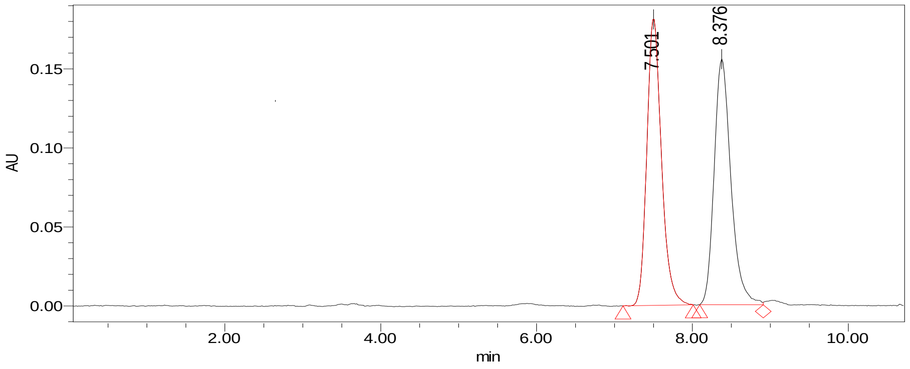

	Retention Time	Area	%Area	Height
1	7.501	2371526	50.42	181616
2	8.376	2332200	49.58	155487

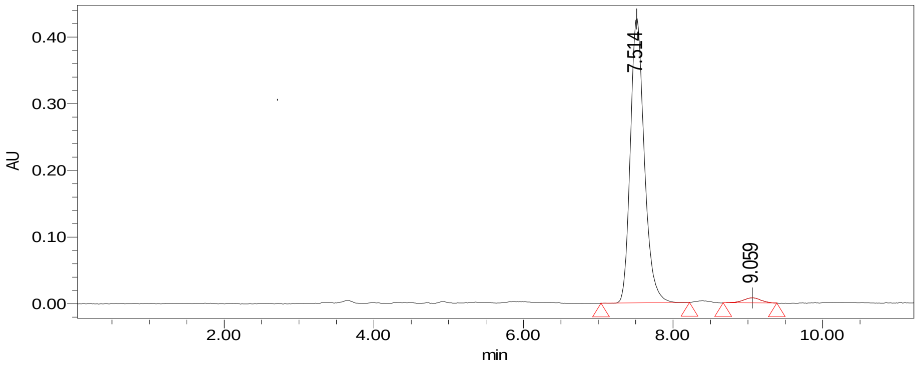

	Retention Time	Area	%Area	Height
1	7.514	5551165	97.85	427054
2	9.059	121886	2.15	7513

Supplementary Figure 139. HPLC chromatogram for compound **2j**

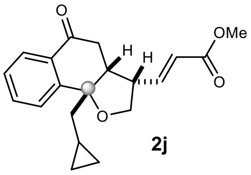

2j

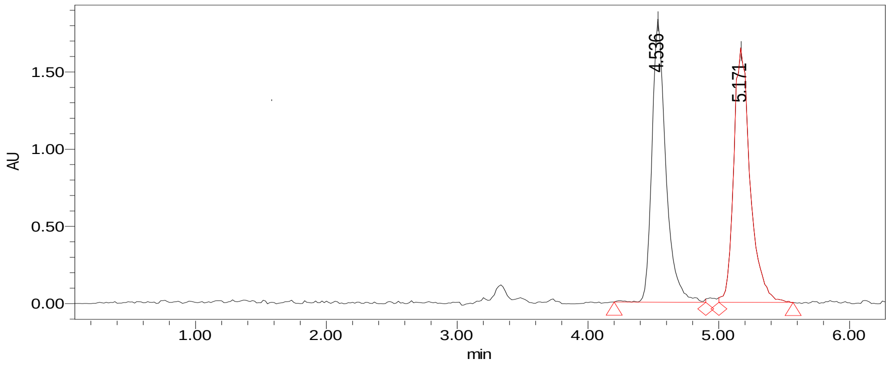

	Retention Time	Area	%Area	Height
1	4.536	13519290	48.47	1802887
2	5.171	14374491	51.53	1586263

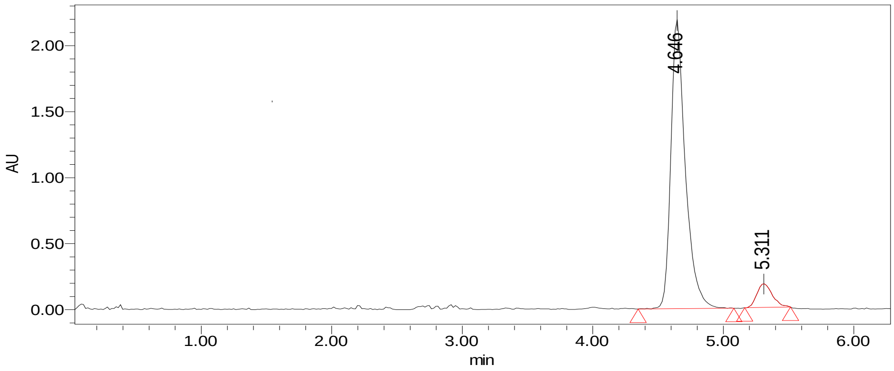

	Retention Time	Area	%Area	Height
1	4.646	16959297	92.00	2183349
2	5.311	1474617	8.00	179291

Supplementary Figure 140. HPLC chromatogram for compound **1k***

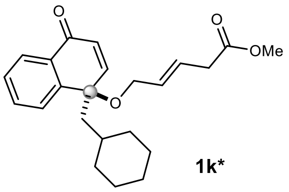

1k*

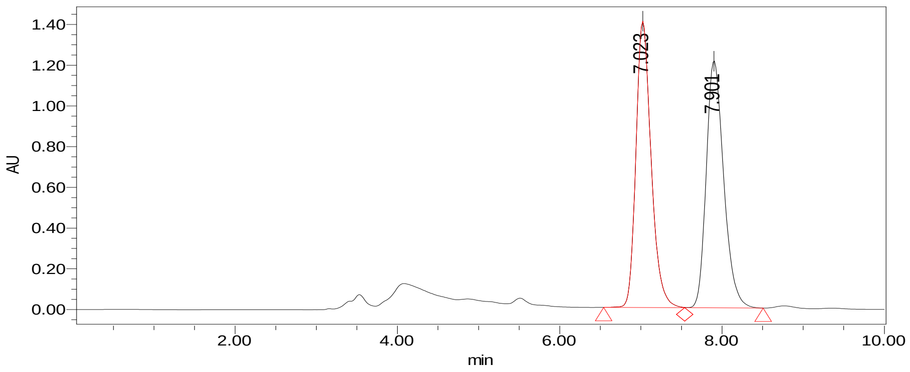

	Retention Time	Area	%Area	Height
1	7.023	18284635	50.18	1404921
2	7.901	18150813	49.82	1213571

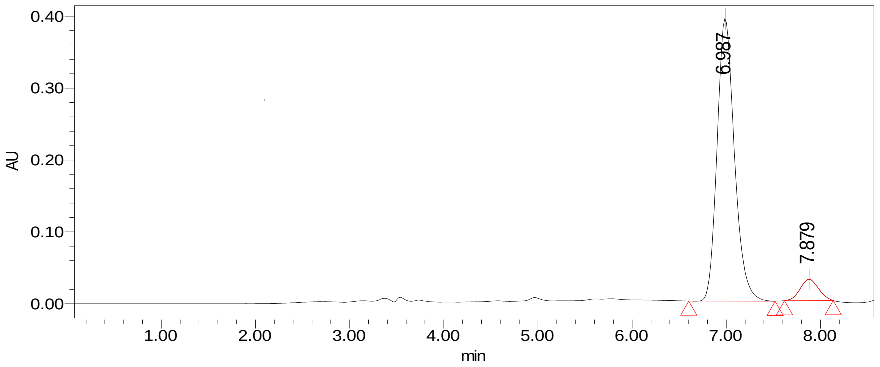

	Retention Time	Area	%Area	Height
1	6.987	5039451	92.65	393039
2	7.879	399791	7.35	29574

Supplementary Figure 141. HPLC chromatogram for compound **2k**

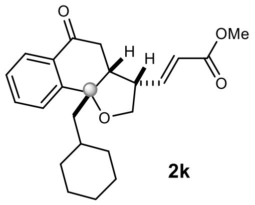

2k

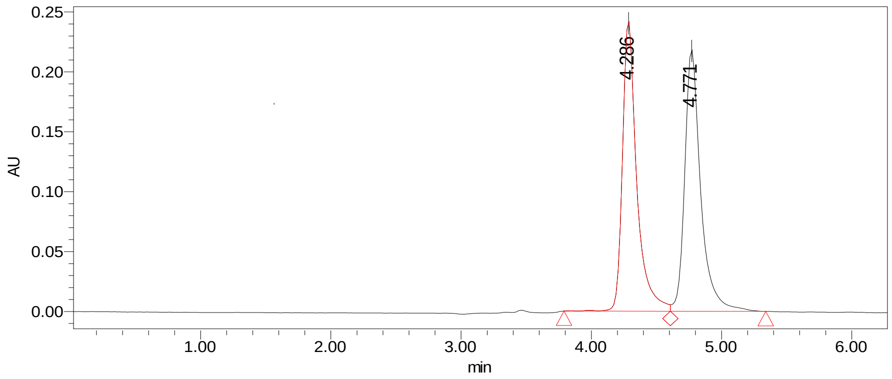

	Retention Time	Area	%Area	Height
1	4.286	1846991	49.70	241181
2	4.771	1869312	50.30	217931

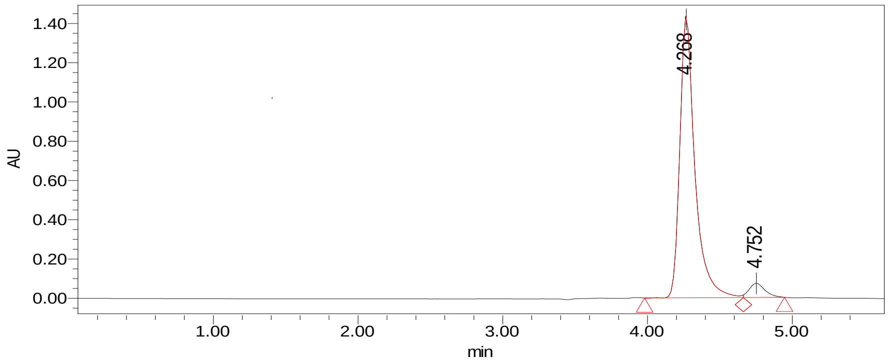

	Retention Time	Area	%Area	Height
1	4.268	10389437	95.00	1430988
2	4.752	546485	5.00	72517

Supplementary Figure 142. HPLC chromatogram for compound **1l***

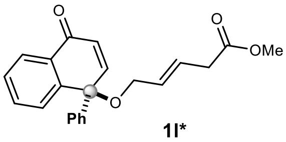

1l*

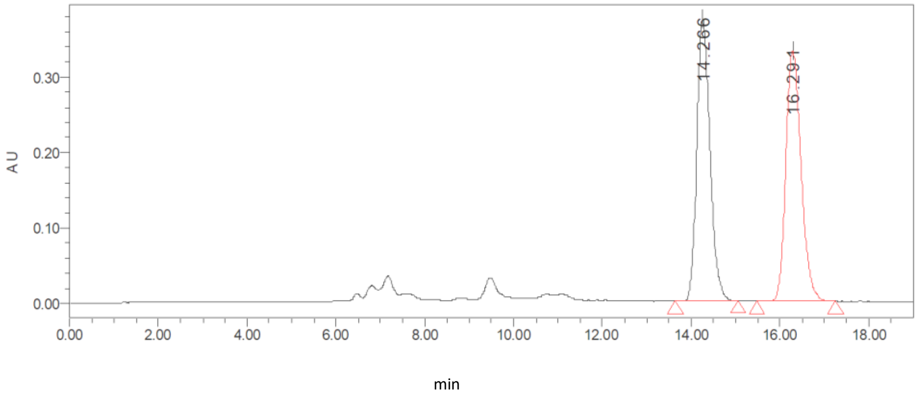

	Retention Time	Area	%Area	Height
1	14.266	7441395	49.06	373348
2	16.291	7725867	50.94	331485

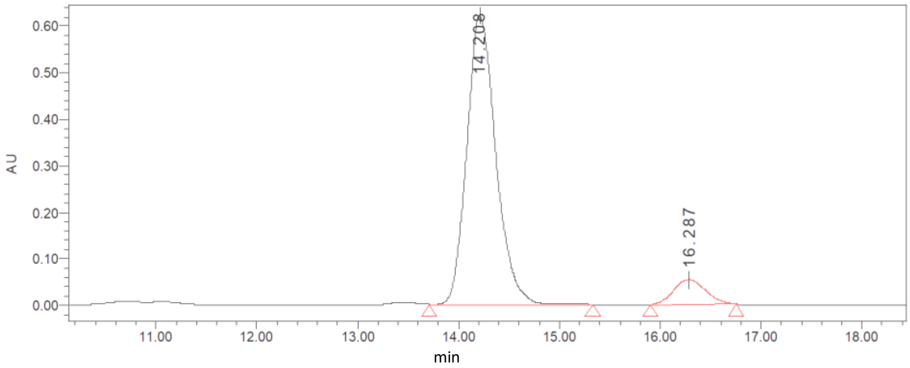

	Retention Time	Area	%Area	Height
1	14.208	12442253	91.40	620468
2	16.287	1170033	8.60	53151

Supplementary Figure 143. HPLC chromatogram for compound **2l**

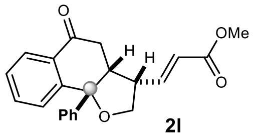

2l

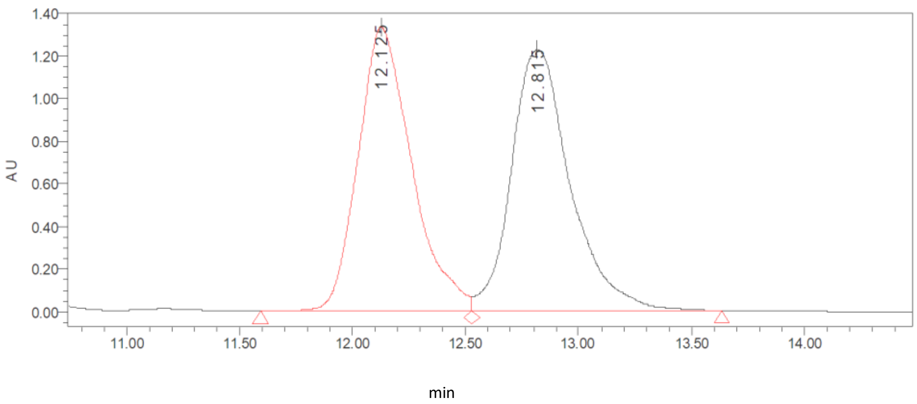

	Retention Time	Area	%Area	Height
1	12.125	22022081	49.75	1332414
2	12.815	22243810	50.25	1224886

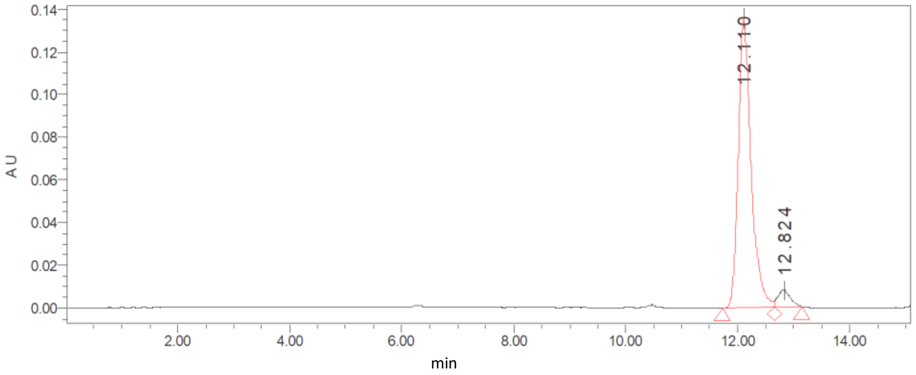

	Retention Time	Area	%Area	Height
1	12.110	2144521	94.87	135447
2	12.824	115925	5.13	7670

Supplementary Figure 144. HPLC chromatogram for compound **1m***

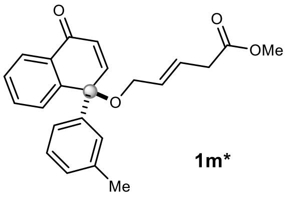

1m*

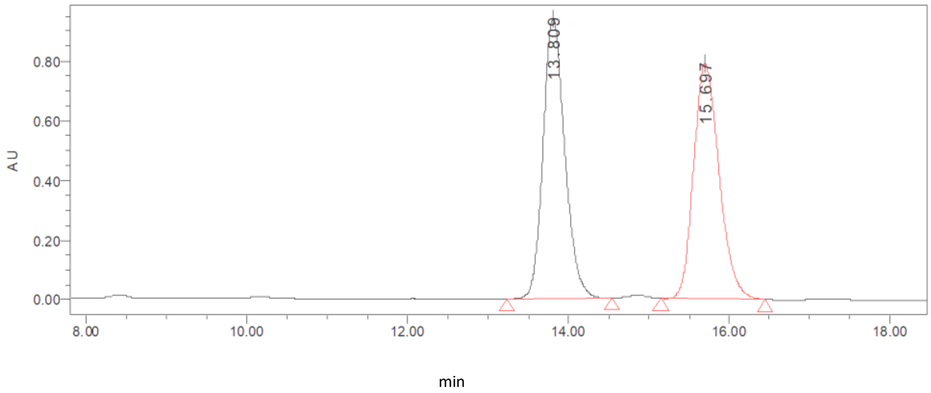

	Retention Time	Area	%Area	Height
1	13.809	17714664	50.19	934518
2	15.697	17577888	49.81	788698

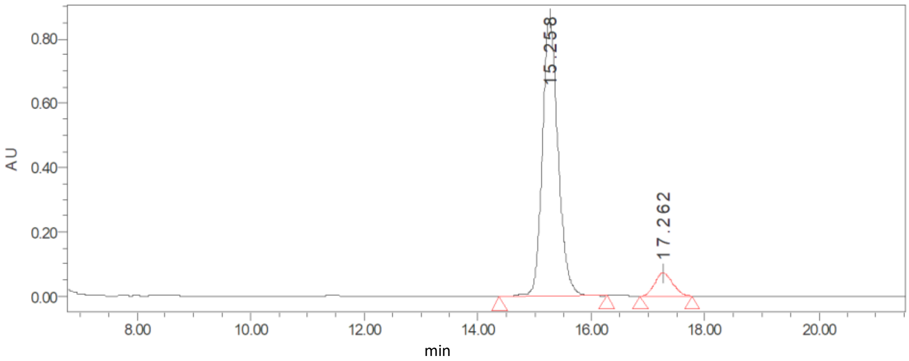

	Retention Time	Area	%Area	Height
1	15.258	16788605	91.54	861330
2	17.262	1551835	8.46	71140

Supplementary Figure 145. HPLC chromatogram for compound **2m**

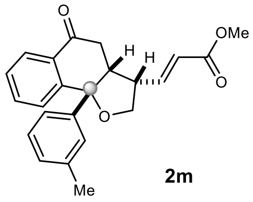

2m

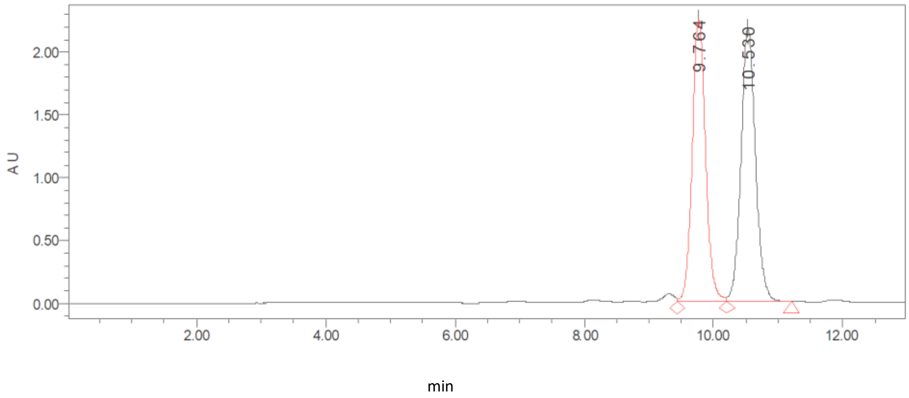

	Retention Time	Area	%Area	Height
1	9.764	32401805	48.69	2247647
2	10.530	34151882	51.31	2175408

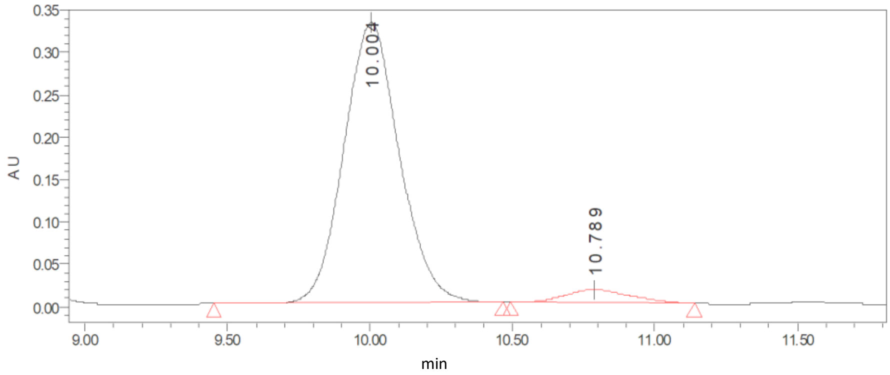

	Retention Time	Area	%Area	Height
1	10.004	4496196	94.72	330897
2	10.789	250407	5.28	15543

Supplementary Figure 146. HPLC chromatogram for compound **1n***

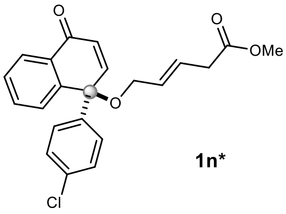

1n*

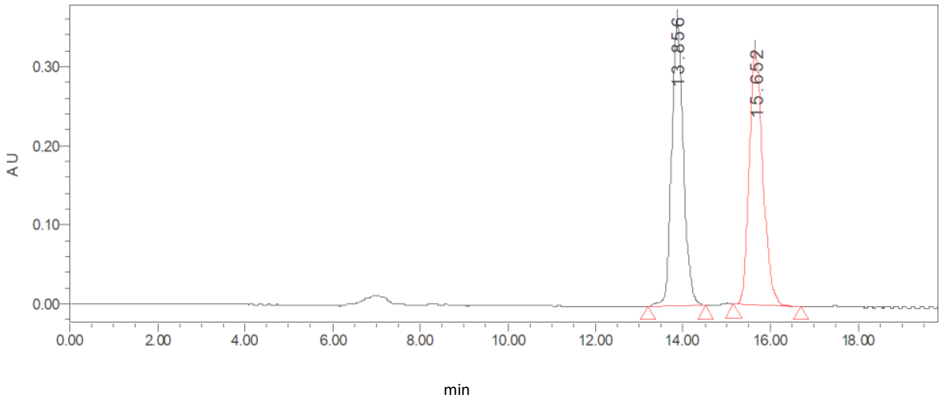

	Retention Time	Area	%Area	Height
1	13.856	6809142	49.31	361935
2	15.652	7000632	50.69	321663

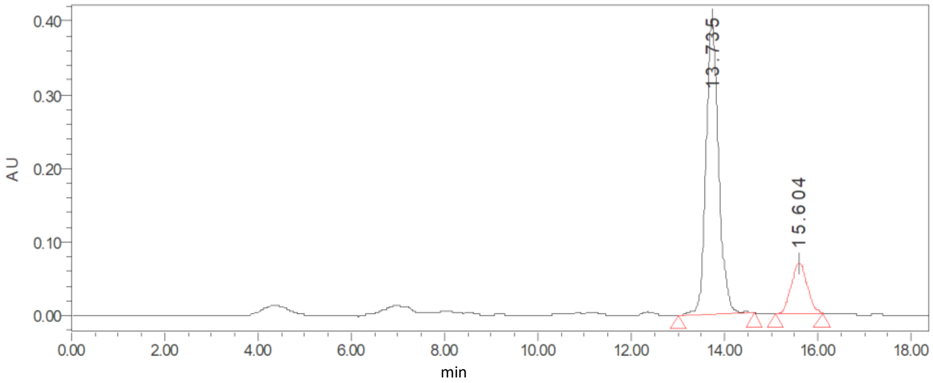

	Retention Time	Area	%Area	Height
1	13.735	7675197	82.28	400316
2	15.604	1653032	17.72	68857

Supplementary Figure 147. HPLC chromatogram for compound **2n**

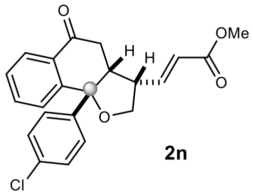

2n

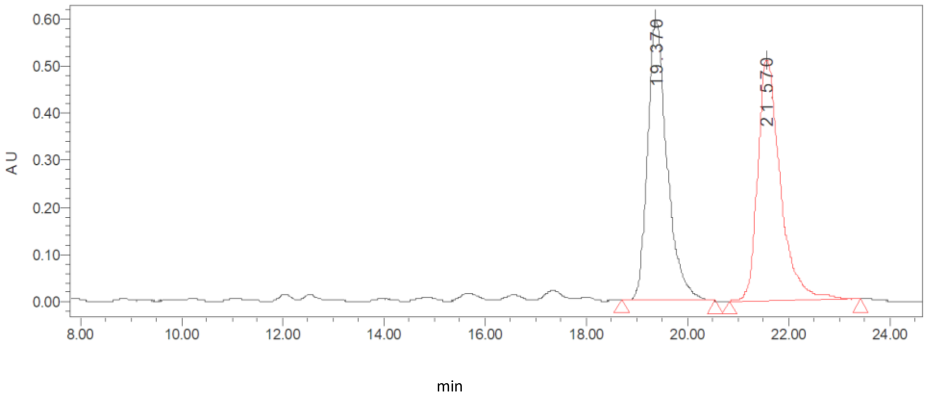

	Retention Time	Area	%Area	Height
1	19.370	16037654	50.25	595001
2	21.570	15876553	49.75	508843

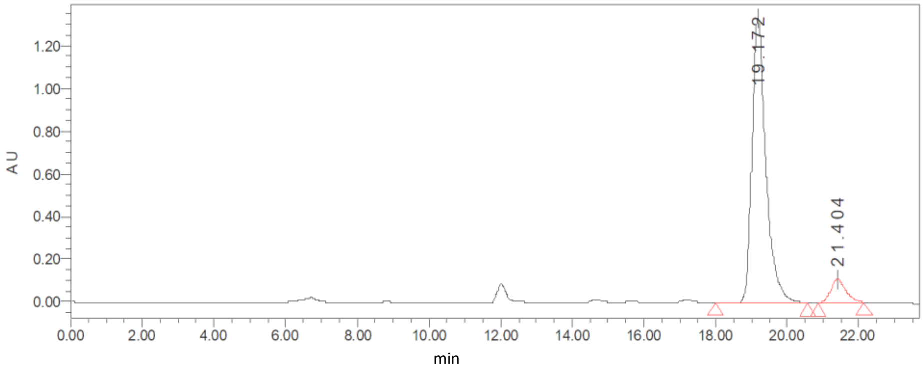

	Retention Time	Area	%Area	Height
1	19.172	36015324	91.92	1328129
2	21.404	3163742	8.08	108654

Supplementary Figure 148. HPLC chromatogram for compound **1o***

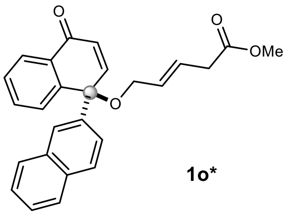

1o*

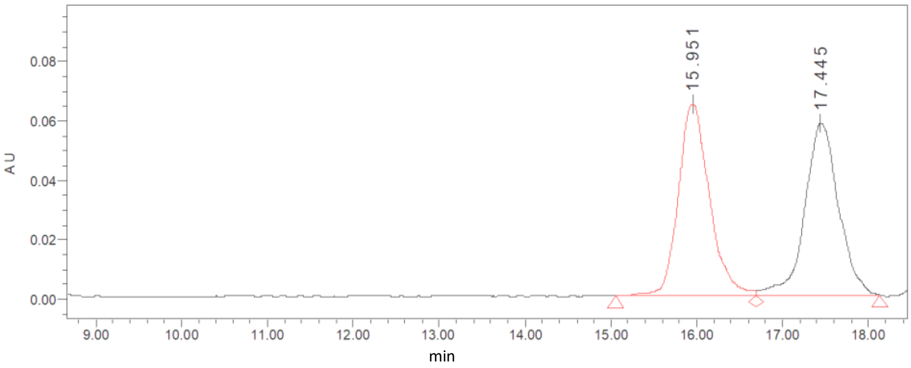

	Retention Time	Area	%Area	Height
1	15.951	1629270	50.68	64517
2	17.445	1585512	49.32	57928

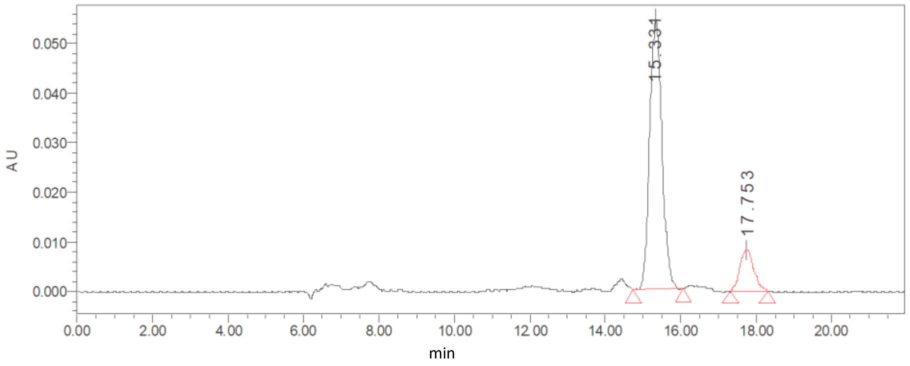

	Retention Time	Area	%Area	Height
1	15.331	1201860	84.91	54464
2	17.753	213658	15.09	8366

Supplementary Figure 149. HPLC chromatogram for compound **2o**

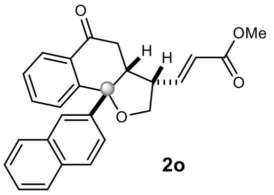

2o

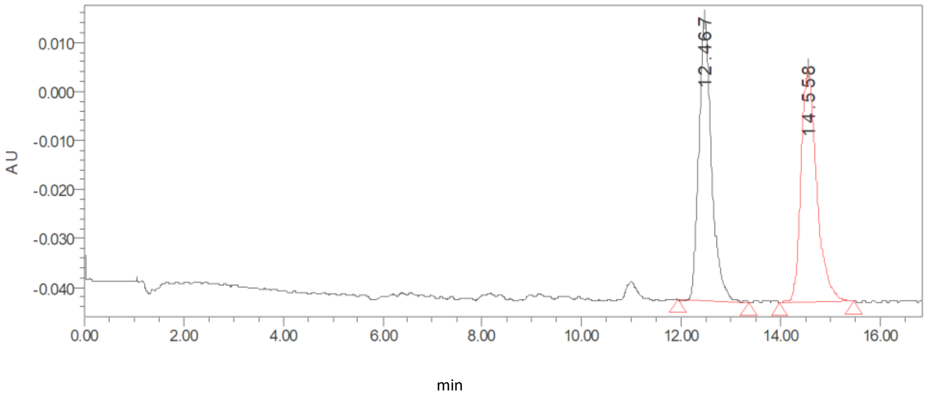

	Retention Time	Area	%Area	Height
1	12.467	1020382	49.71	57274
2	14.558	1032260	50.29	47665

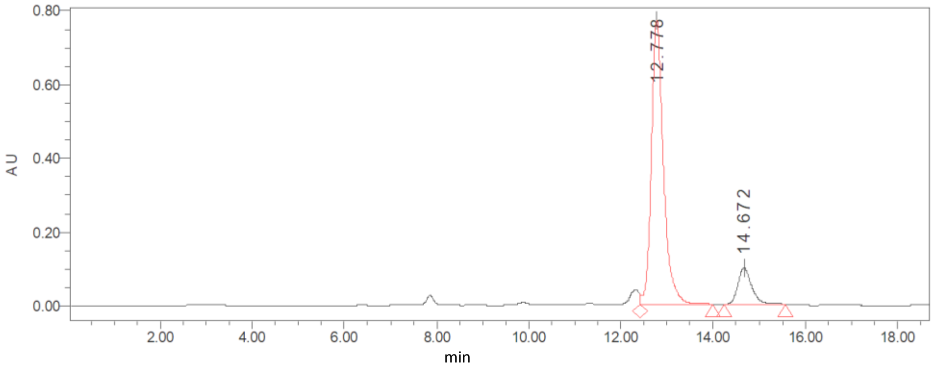

	Retention Time	Area	%Area	Height
1	12.778	14228127	87.29	770679
2	14.672	2070809	12.71	99495

Supplementary Figure 150. HPLC chromatogram for compound **1p***

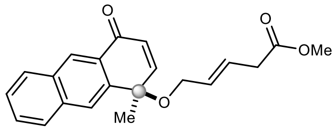

1p*

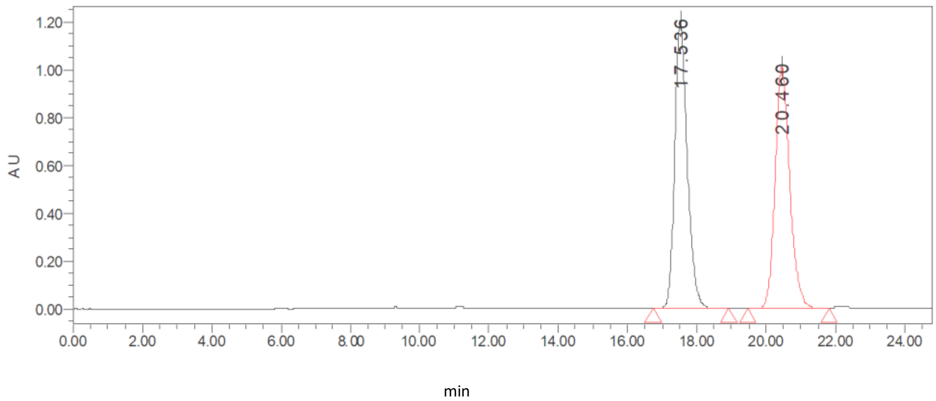

	Retention Time	Area	%Area	Height
1	17.536	30494948	50.25	1203042
2	20.460	30193129	49.75	1009966

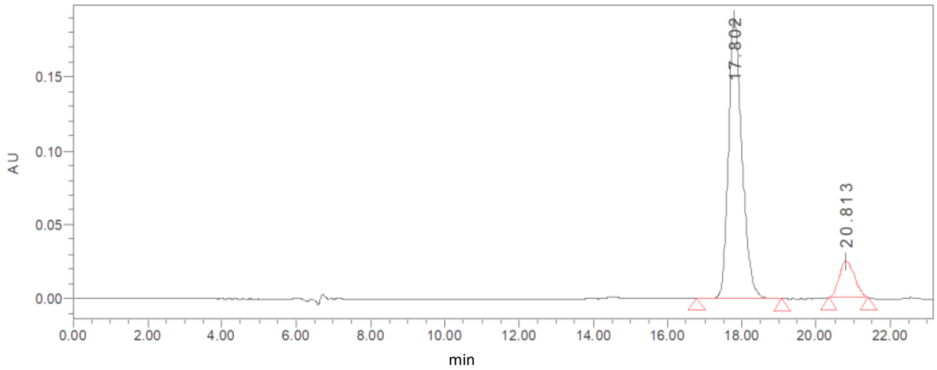

	Retention Time	Area	%Area	Height
1	17.802	4854109	87.35	188657
2	20.813	702972	12.65	24988

Supplementary Figure 151. HPLC chromatogram for compound **2p**

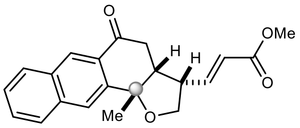

2p

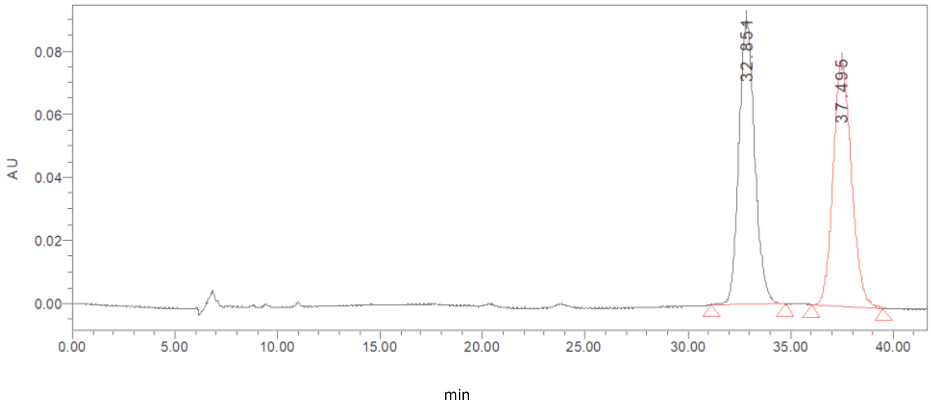

	Retention Time	Area	%Area	Height
1	32.851	4848433	49.99	90045
2	37.495	4849695	50.01	77624

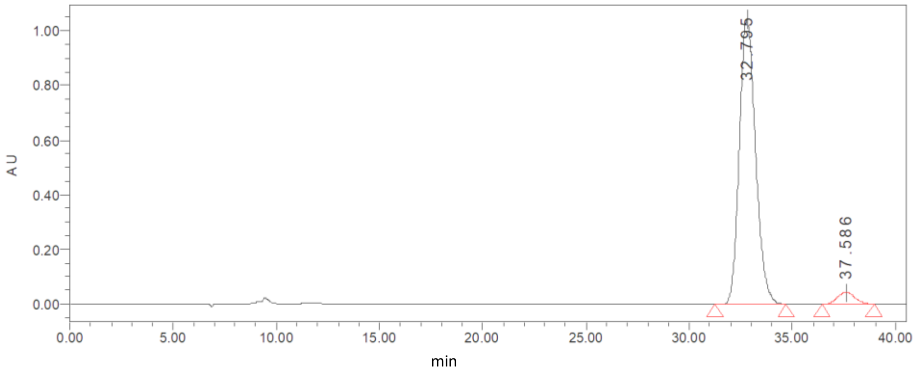

	Retention Time	Area	%Area	Height
1	32.795	56836550	95.66	1042296
2	37.586	2577758	4.34	42421

Supplementary Figure 152. HPLC chromatogram for compound **1q***

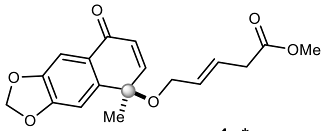

1q*

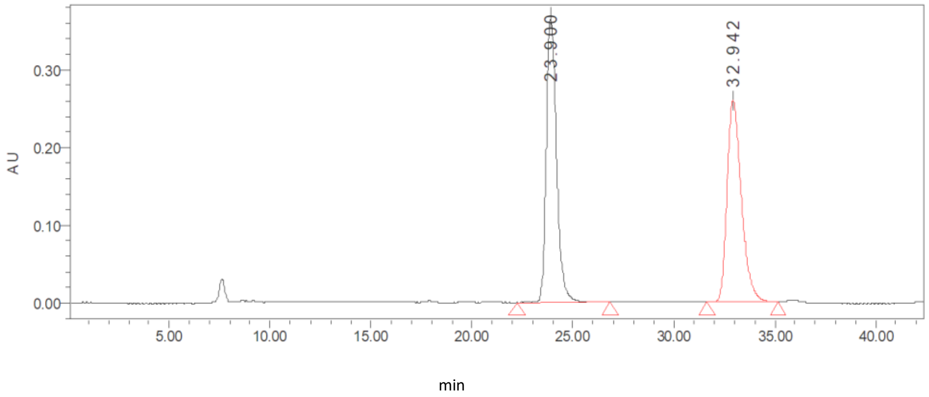

	Retention Time	Area	%Area	Height
1	23.900	12891836	49.98	366568
2	32.942	12904710	50.02	259643

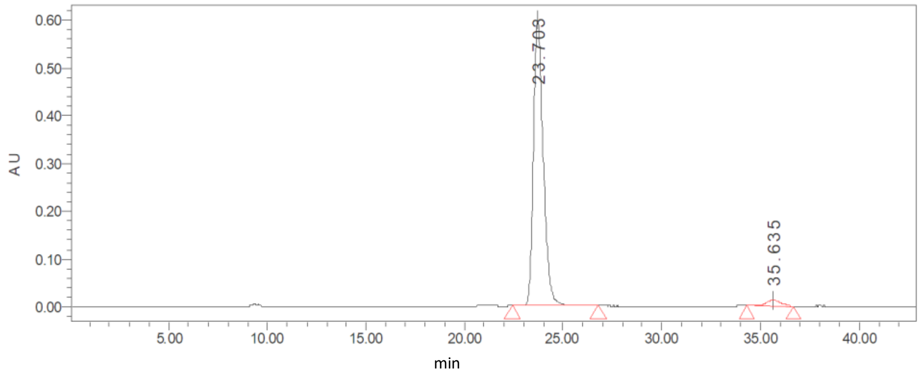

	Retention Time	Area	%Area	Height
1	23.703	20674445	97.08	598857
2	35.635	622272	2.92	12837

Supplementary Figure 153. HPLC chromatogram for compound **2q**

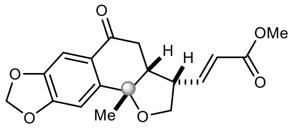

2q

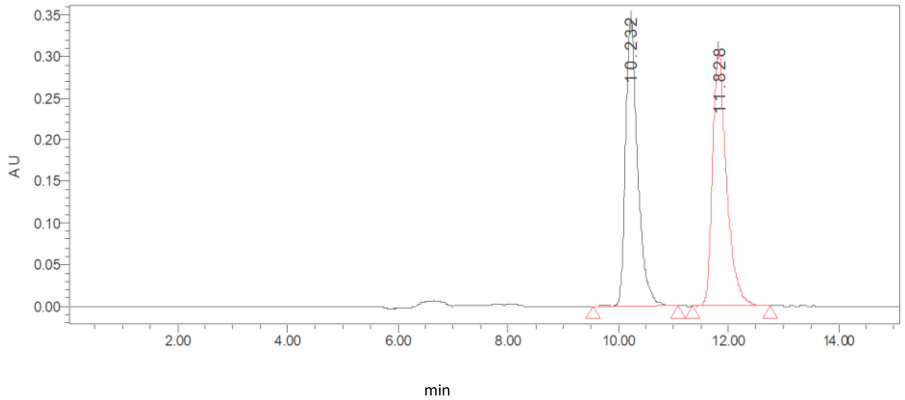

	Retention Time	Area	%Area	Height
1	10.232	5357617	49.94	343630
2	11.828	5369904	50.06	306497

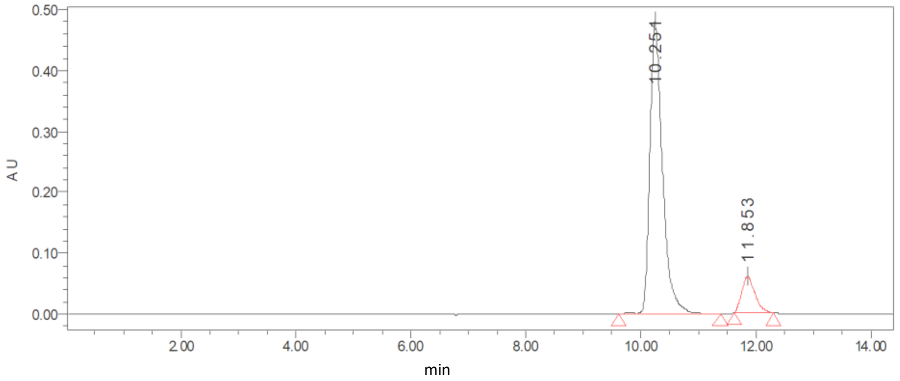

	Retention Time	Area	%Area	Height
1	10.251	7107457	88.17	480056
2	11.853	953443	11.83	60723

Supplementary Figure 154. HPLC chromatogram for compound **1r***

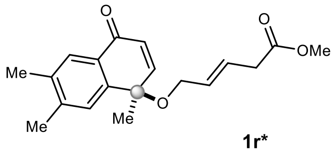

1r*

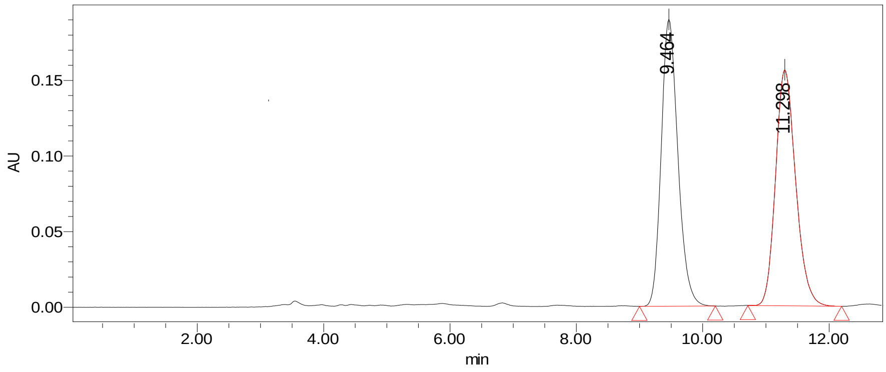

	Retention Time	Area	%Area	Height
1	9.464	3483052	50.24	189690
2	11.298	3449527	49.76	156119

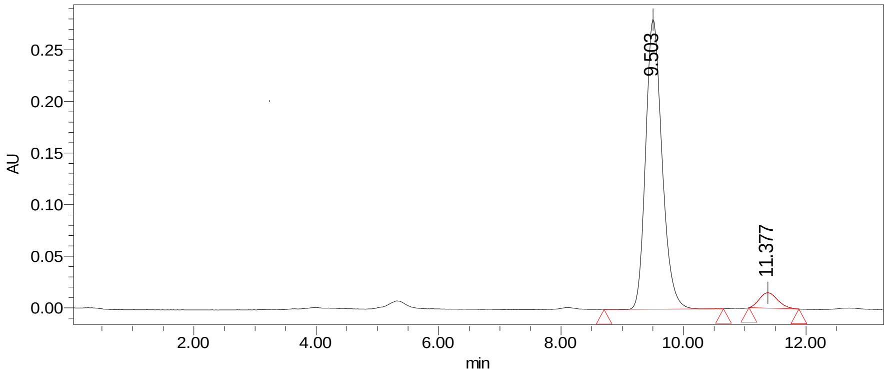

	Retention Time	Area	%Area	Height
1	9.503	5216420	94.39	280793
2	11.377	309928	5.61	14934

Supplementary Figure 155. HPLC chromatogram for compound **2r**

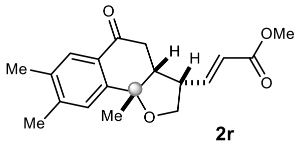

2r

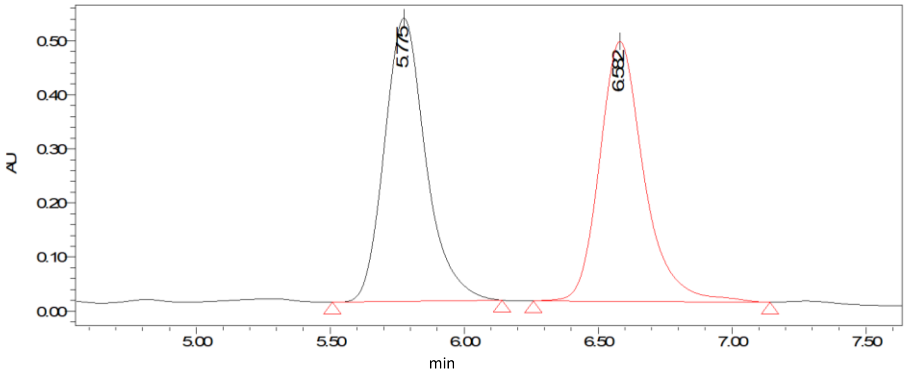

	Retention Time	Area	%Area	Height
1	5.775	5620791	50.32	524538
2	6.582	5548377	49.68	481433

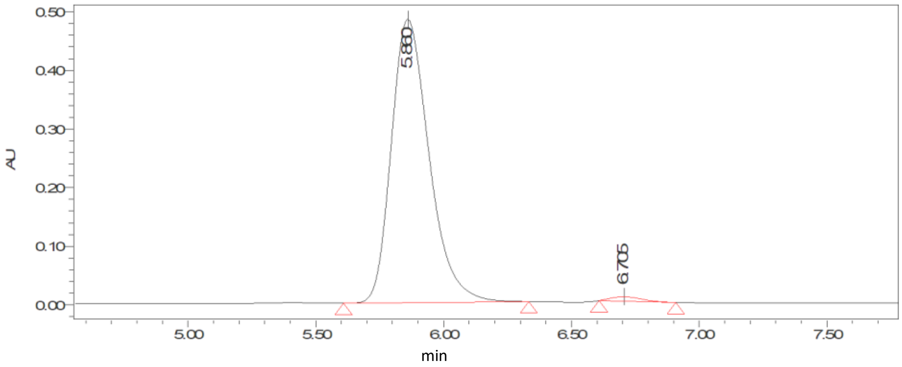

	Retention Time	Area	%Area	Height
1	5.860	4974193	98.77	483975
2	6.705	62046	1.23	7431

Supplementary Figure 156. HPLC chromatogram for compound **1s***

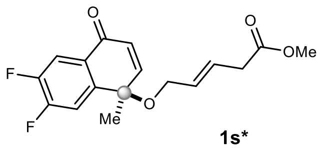

1s*

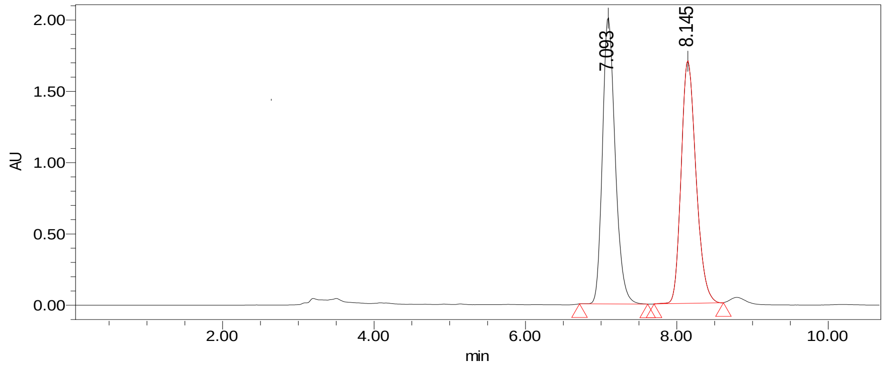

	Retention Time	Area	%Area	Height
1	7.093	23471247	50.21	2010761
2	8.145	23275491	49.79	1702868

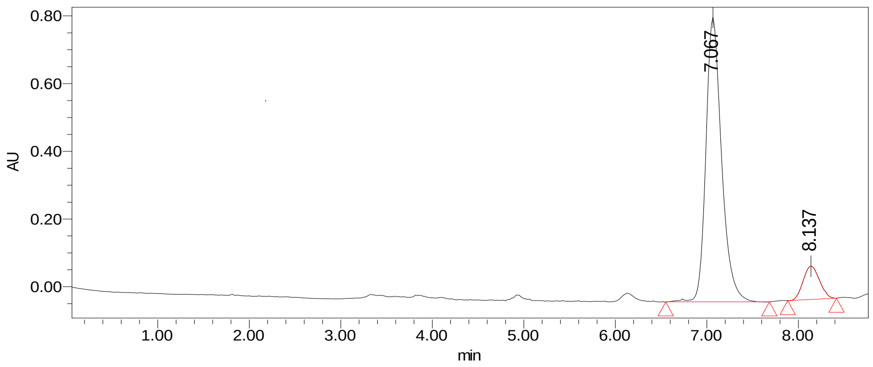

	Retention Time	Area	%Area	Height
1	7.067	10052898	89.18	838772
2	8.137	1219155	10.82	98778

Supplementary Figure 157. HPLC chromatogram for compound **2s**

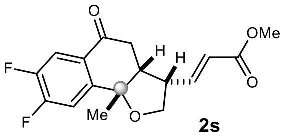

2s

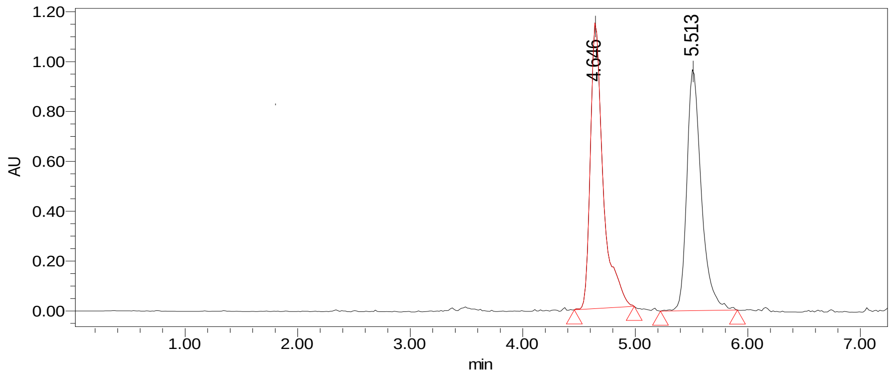

	Retention Time	Area	%Area	Height
1	4.646	9071518	51.05	1143608
2	5.513	8697448	48.95	969294

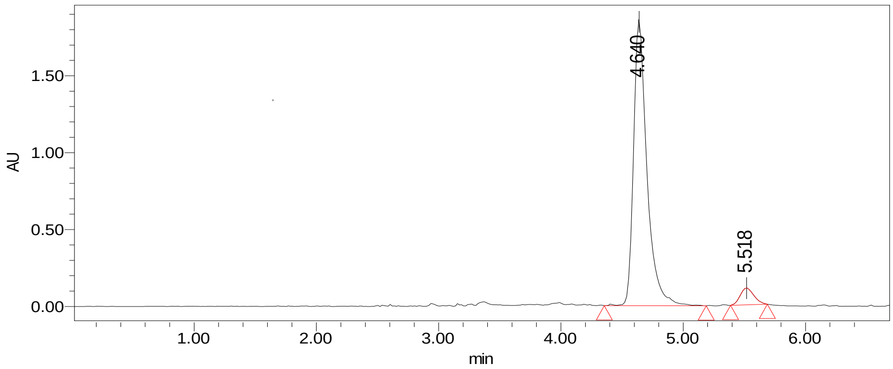

	Retention Time	Area	%Area	Height
1	4.640	14204825	94.32	1858279
2	5.518	855657	5.68	109784

Supplementary Figure 158. HPLC chromatogram for compound **1t***

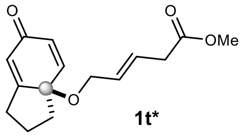

1t*

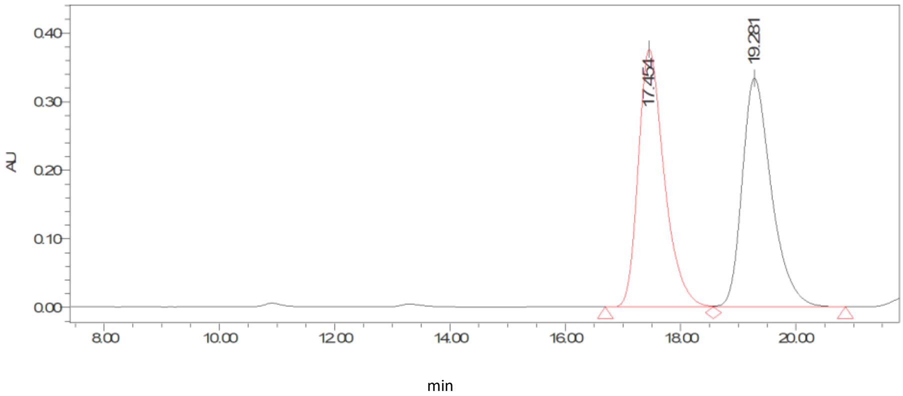

	Retention Time	Area	%Area	Height
1	17.454	11761286	49.91	375732
2	19.281	11801379	50.09	333457

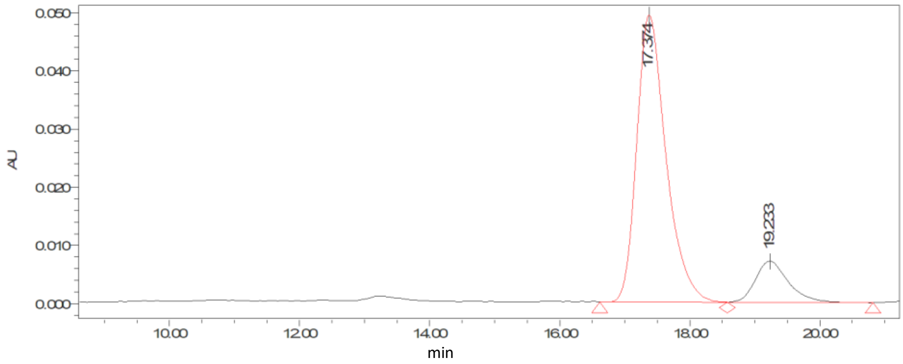

	Retention Time	Area	%Area	Height
1	17.374	1544111	86.13	49309
2	19.233	248612	13.87	7072

Supplementary Figure 159. HPLC chromatogram for compound **2t**

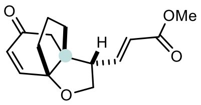

2t

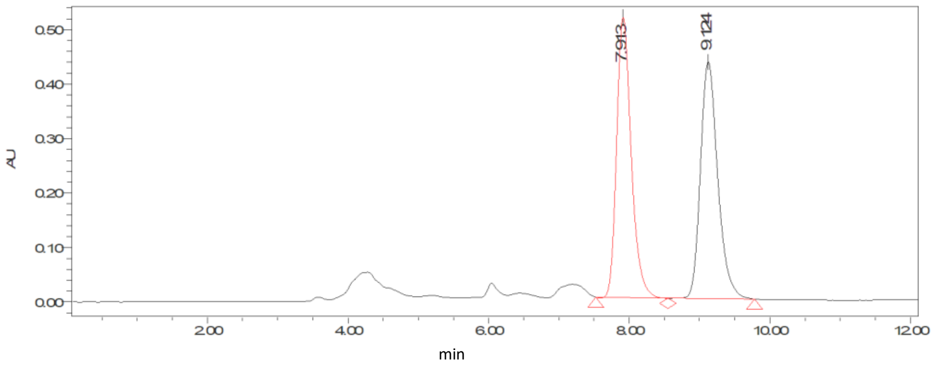

	Retention Time	Area	%Area	Height
1	7.913	7406188	49.93	514545
2	9.124	7427198	50.07	434208

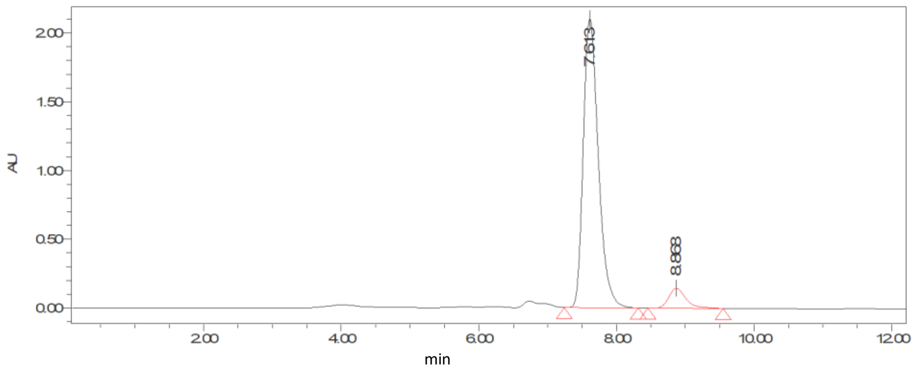

	Retention Time	Area	%Area	Height
1	7.613	31881558	92.81	2100491
2	8.868	2468865	7.19	144535

Supplementary Figure 160. HPLC chromatogram for compound **3**

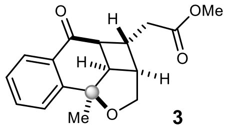

3

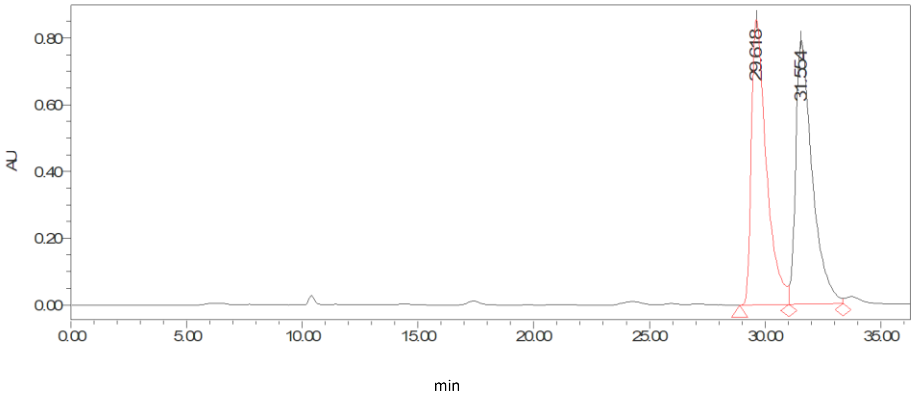

	Retention Time	Area	%Area	Height
1	29.618	38463824	49.40	855879
2	31.554	39396893	50.60	791847

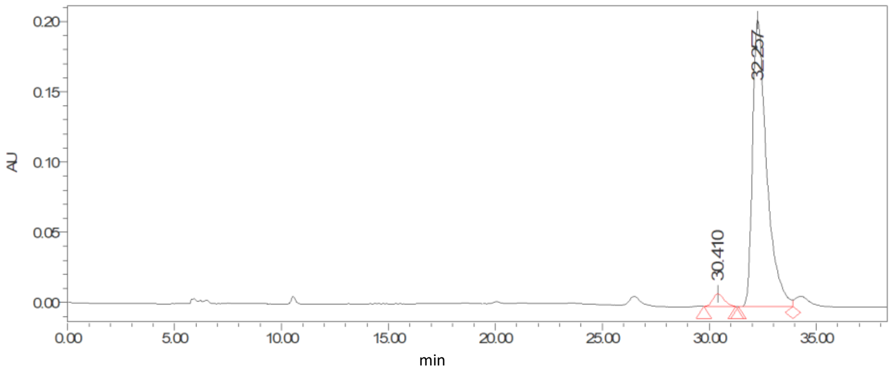

	Retention Time	Area	%Area	Height
1	30.410	323892	3.34	8943
2	32.257	9385552	96.66	204009

Supplementary Figure 161. HPLC chromatogram for compound **4**

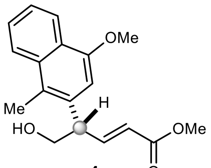

4

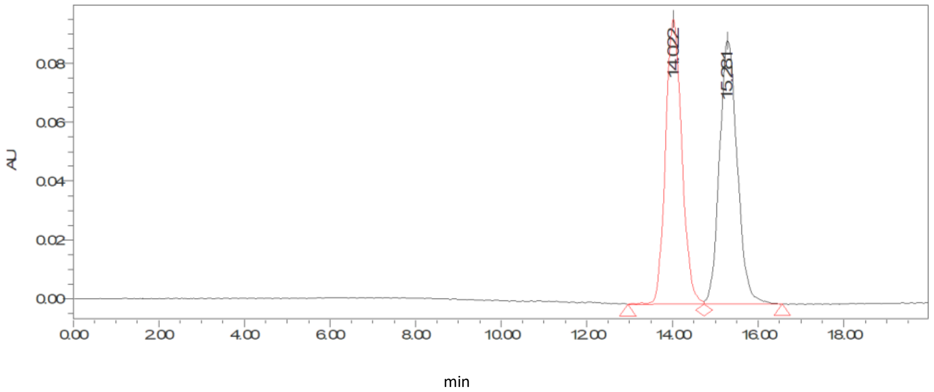

	Retention Time	Area	%Area	Height
1	14.022	2568661	49.75	96877
2	15.281	2594872	50.25	89559

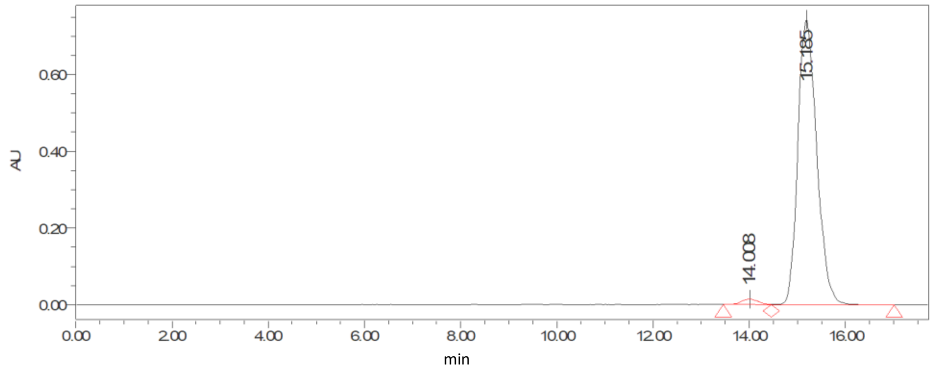

	Retention Time	Area	%Area	Height
1	14.008	345149	1.67	14472
2	15.185	20271981	98.33	742669

Supplementary Figure 162. HPLC chromatogram for compound **7**

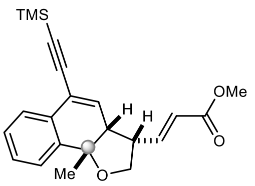

7

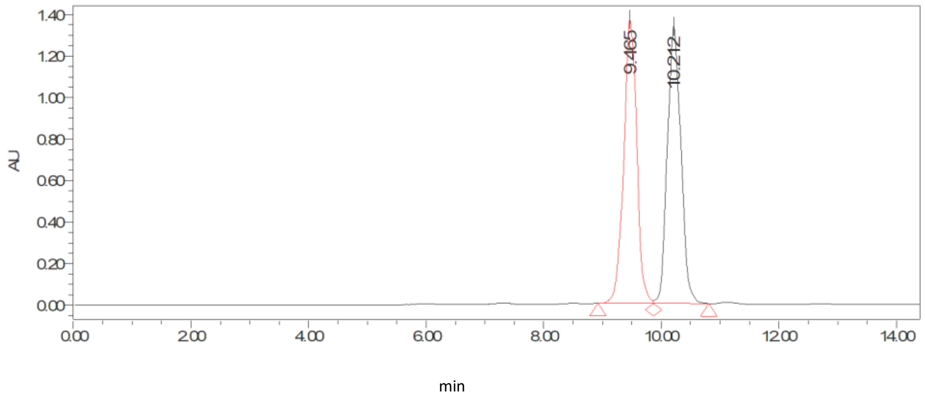

	Retention Time	Area	%Area	Height
1	9.465	21172562	49.81	1365533
2	10.212	21330189	50.19	1335403

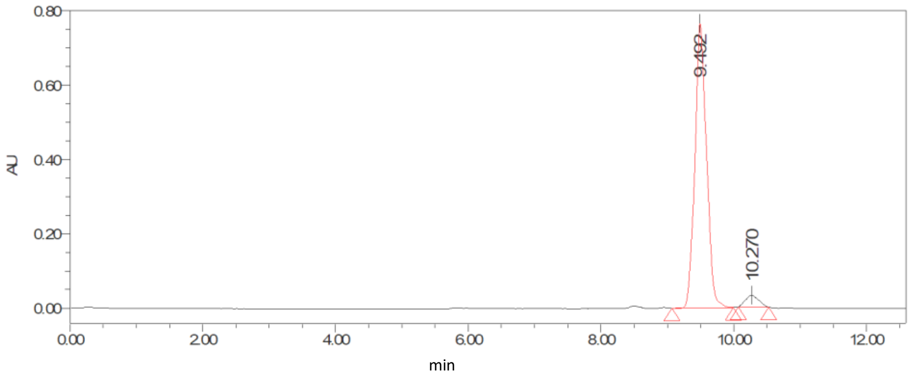

	Retention Time	Area	%Area	Height
1	9.492	9296288	95.43	765199
2	10.270	445552	4.57	32419

Supplementary Figure 163. HPLC chromatogram for compound **8**

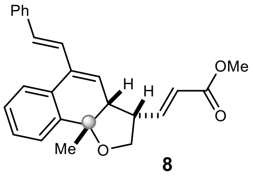

8

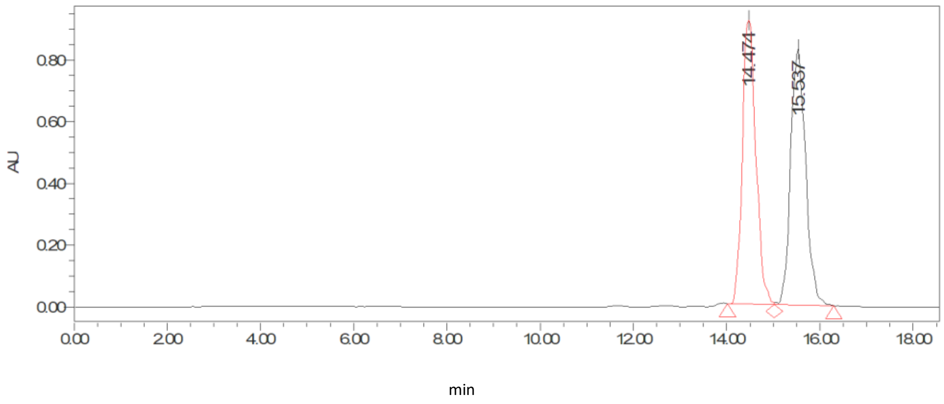

	Retention Time	Area	%Area	Height
1	14.474	18636385	50.06	919404
2	15.537	18592346	49.94	828763

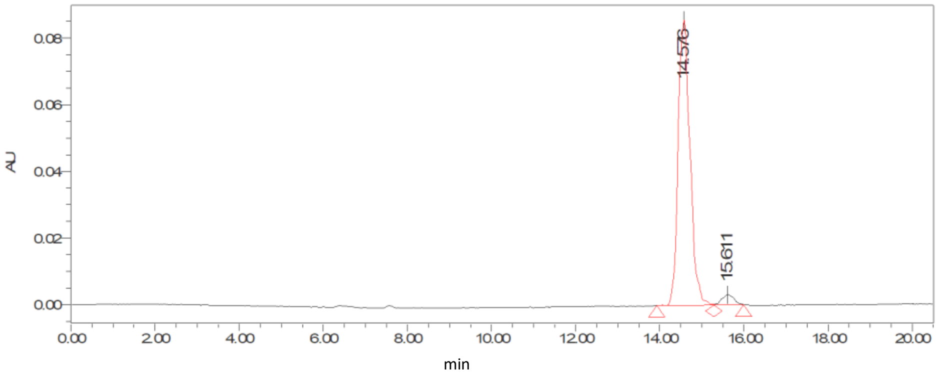

	Retention Time	Area	%Area	Height
1	14.576	1677666	96.43	85584
2	15.611	62106	3.57	3107

Supplementary Figure 164. HPLC chromatogram for compound **10**

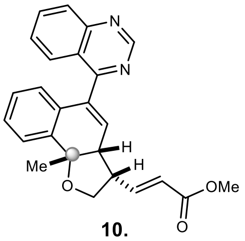

10.

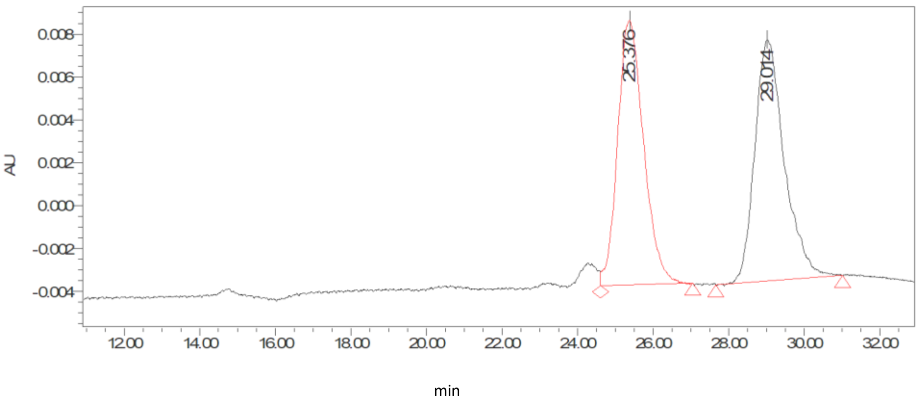

	Retention Time	Area	%Area	Height
1	25.376	587541	49.08	12340
2	29.014	609525	50.92	11256

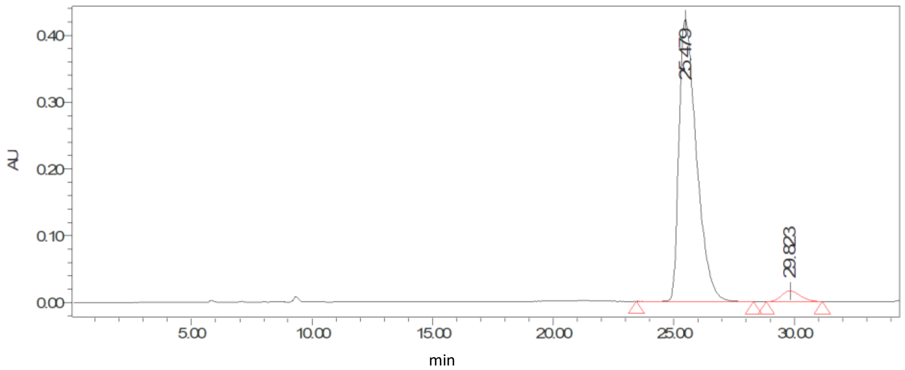

	Retention Time	Area	%Area	Height
1	25.479	21331120	96.06	422425
2	29.823	875960	3.94	16130

Supplementary Figure 165. HRMS chromatogram for compound **1a***

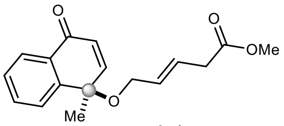

1a*

Acquisition Parameter

Source Type	ESI	Ion Polarity	Positive	Set Nebulizer	0.3 Bar
Focus	Not active	Set Capillary	4500 V	Set Dry Heater	180 °C
Scan Begin	100 m/z	Set End Plate Offset	-500 V	Set Dry Gas	4.0 l/min
Scan End	1200 m/z	Set Collision Cell RF	1500.0 Vpp	Set Divert Valve	Source

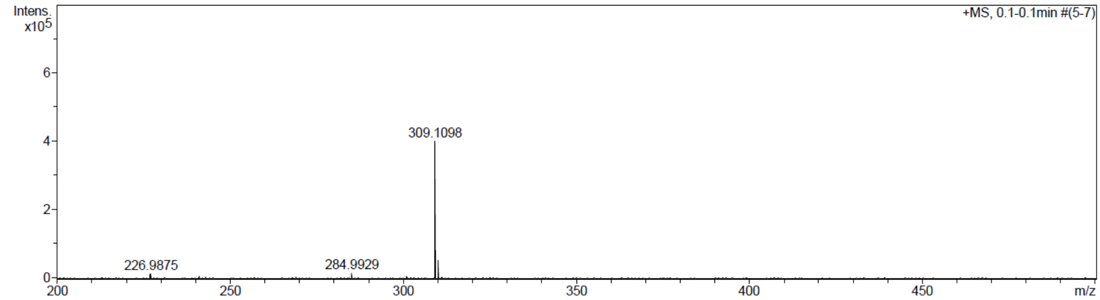

Meas. m/z	#	Formula	Score	m/z	err [mDa]	err [ppm]	mSigma	rdb	e¯ Conf	N-Rule
309.1098	1	C 17 H 18 Na O 4	100.00	309.1097	-0.1	-0.3	30.9	8.5	even	ok

Supplementary Figure 166. HRMS chromatogram for compound **2a**

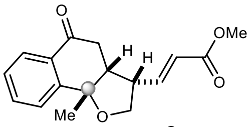

2a

Acquisition Parameter

Source Type	ESI	Ion Polarity	Positive	Set Nebulizer	0.3 Bar
Focus	Not active	Set Capillary	4500 V	Set Dry Heater	180 °C
Scan Begin	100 m/z	Set End Plate Offset	-500 V	Set Dry Gas	4.0 l/min
Scan End	1200 m/z	Set Collision Cell RF	1500.0 Vpp	Set Divert Valve	Source

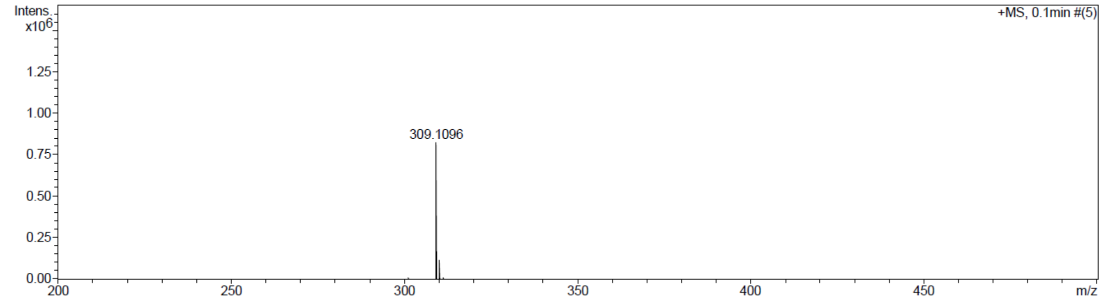

Meas. m/z	#	Formula	Score	m/z	err [mDa]	err [ppm]	mSigma	rdb	e¯ Conf	N-Rule
309.1096	1	C 17 H 18 Na O 4	100.00	309.1097	0.1	0.3	27.1	8.5	even	ok

Supplementary Figure 167. HRMS chromatogram for compound **1b***

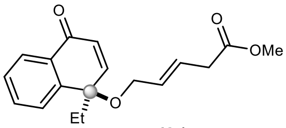

1b*

Acquisition Parameter

Source Type	ESI	Ion Polarity	Positive	Set Nebulizer	0.3 Bar
Focus	Not active	Set Capillary	4500 V	Set Dry Heater	180 °C
Scan Begin	100 m/z	Set End Plate Offset	-500 V	Set Dry Gas	4.0 l/min
Scan End	1200 m/z	Set Collision Cell RF	1500.0 Vpp	Set Divert Valve	Source

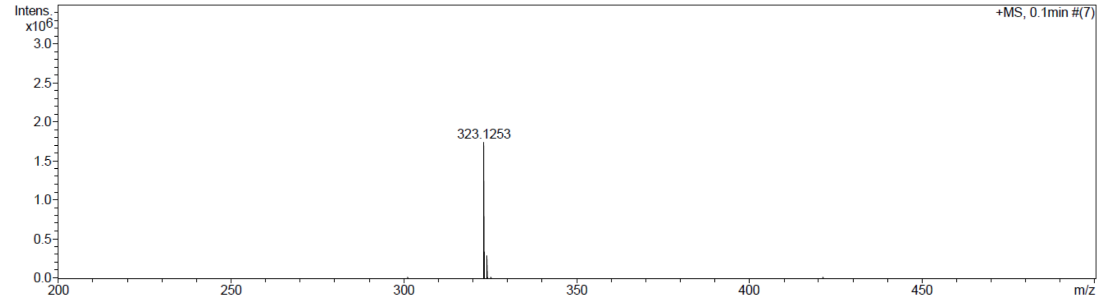

Meas. m/z	#	Formula	Score	m/z	err [mDa]	err [ppm]	mSigma	rdb	e⁻ Conf	N-Rule
323.1253	1	C 18 H 20 Na O 4	100.00	323.1254	0.1	0.4	16.0	8.5	even	ok

Supplementary Figure 168. HRMS chromatogram for compound **2b**

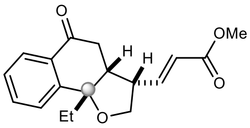

2b

Acquisition Parameter

Source Type	ESI	Ion Polarity	Positive	Set Nebulizer	0.3 Bar
Focus	Not active	Set Capillary	4500 V	Set Dry Heater	180 °C
Scan Begin	100 m/z	Set End Plate Offset	-500 V	Set Dry Gas	4.0 l/min
Scan End	1200 m/z	Set Collision Cell RF	1500.0 Vpp	Set Divert Valve	Source

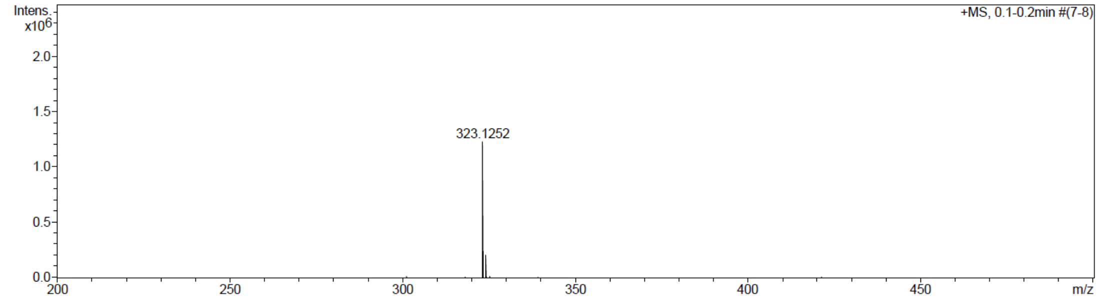

Meas. m/z	#	Formula	Score	m/z	err [mDa]	err [ppm]	mSigma	rdb	e⁻ Conf	N-Rule
323.1252	1	C 18 H 20 Na O 4	100.00	323.1254	0.2	0.6	16.5	8.5	even	ok

Supplementary Figure 169. HRMS chromatogram for compound **1c***

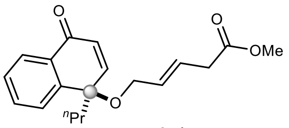

1c*

Acquisition Parameter

Source Type	ESI	Ion Polarity	Positive	Set Nebulizer	0.3 Bar
Focus	Not active	Set Capillary	4500 V	Set Dry Heater	180 °C
Scan Begin	100 m/z	Set End Plate Offset	-500 V	Set Dry Gas	4.0 l/min
Scan End	1200 m/z	Set Collision Cell RF	1500.0 Vpp	Set Divert Valve	Source

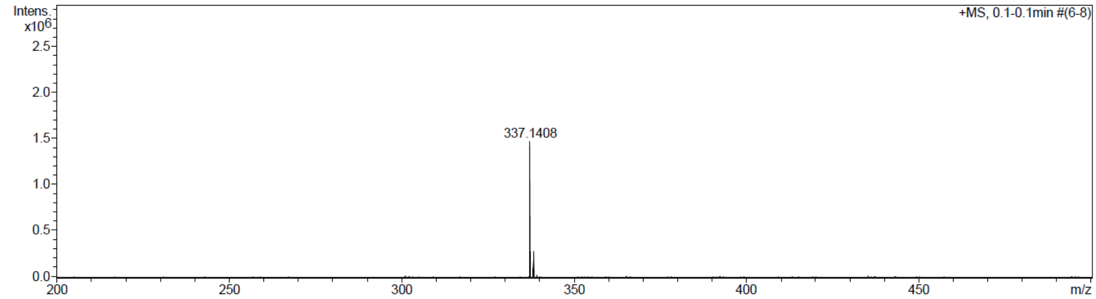

Meas. m/z	#	Formula	Score	m/z	err [mDa]	err [ppm]	mSigma	rdb	e¯ Conf	N-Rule
337.1408	1	C 19 H 22 Na O 4	100.00	337.1410	0.3	0.8	11.3	8.5	even	ok

Supplementary Figure 170. HRMS chromatogram for compound **2c**

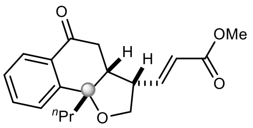

2c

Acquisition Parameter

Source Type	ESI	Ion Polarity	Positive	Set Nebulizer	0.3 Bar
Focus	Not active	Set Capillary	4500 V	Set Dry Heater	180 °C
Scan Begin	100 m/z	Set End Plate Offset	-500 V	Set Dry Gas	4.0 l/min
Scan End	1200 m/z	Set Collision Cell RF	1500.0 Vpp	Set Divert Valve	Source

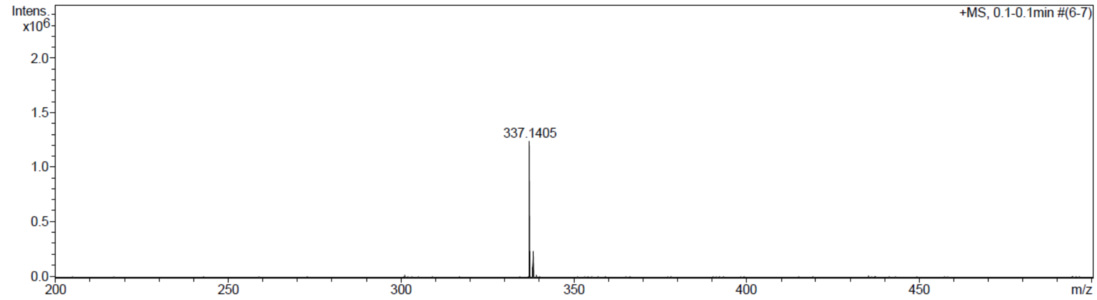

Meas. m/z	#	Formula	Score	m/z	err [mDa]	err [ppm]	mSigma	rdb	e¯ Conf	N-Rule
337.1405	1	C 19 H 22 Na O 4	100.00	337.1410	0.5	1.5	10.9	8.5	even	ok

Supplementary Figure 171. HRMS chromatogram for compound **1d***

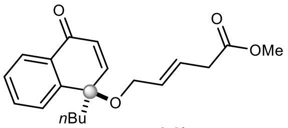

1d*

Acquisition Parameter

Source Type	ESI	Ion Polarity	Positive	Set Nebulizer	0.3 Bar
Focus	Not active	Set Capillary	4500 V	Set Dry Heater	180 °C
Scan Begin	100 m/z	Set End Plate Offset	-500 V	Set Dry Gas	4.0 l/min
Scan End	1200 m/z	Set Collision Cell RF	1500.0 Vpp	Set Divert Valve	Source

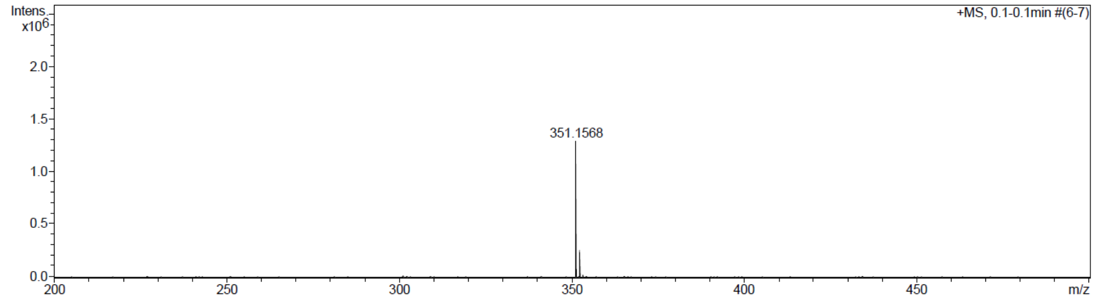

Meas. m/z	#	Formula	Score	m/z	err [mDa]	err [ppm]	mSigma	rdb	e⁻ Conf	N-Rule
351.1568	1	C 20 H 24 Na O 4	100.00	351.1567	-0.1	-0.2	13.8	8.5	even	ok

Supplementary Figure 172. HRMS chromatogram for compound **2d**

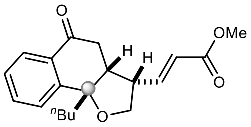

2d

Acquisition Parameter

Source Type	ESI	Ion Polarity	Positive	Set Nebulizer	0.3 Bar
Focus	Not active	Set Capillary	4500 V	Set Dry Heater	180 °C
Scan Begin	100 m/z	Set End Plate Offset	-500 V	Set Dry Gas	4.0 l/min
Scan End	1200 m/z	Set Collision Cell RF	1500.0 Vpp	Set Divert Valve	Source

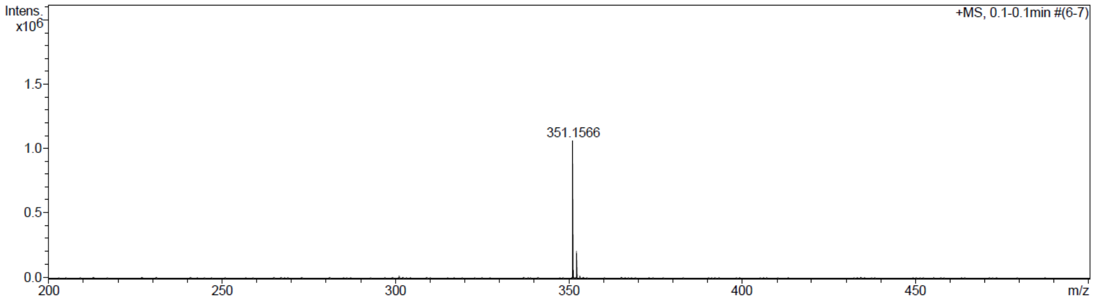

Meas. m/z	#	Formula	Score	m/z	err [mDa]	err [ppm]	mSigma	rdb	e⁻ Conf	N-Rule
351.1566	1	C 20 H 24 Na O 4	100.00	351.1567	0.1	0.3	14.7	8.5	even	ok

Supplementary Figure 173. HRMS chromatogram for compound **1e***

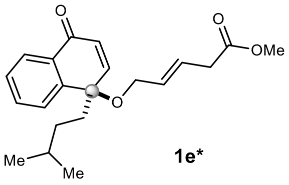

1e*

Acquisition Parameter

Source Type	ESI	Ion Polarity	Positive	Set Nebulizer	0.3 Bar
Focus	Not active	Set Capillary	4500 V	Set Dry Heater	180 °C
Scan Begin	100 m/z	Set End Plate Offset	-500 V	Set Dry Gas	4.0 l/min
Scan End	1200 m/z	Set Collision Cell RF	1500.0 Vpp	Set Divert Valve	Source

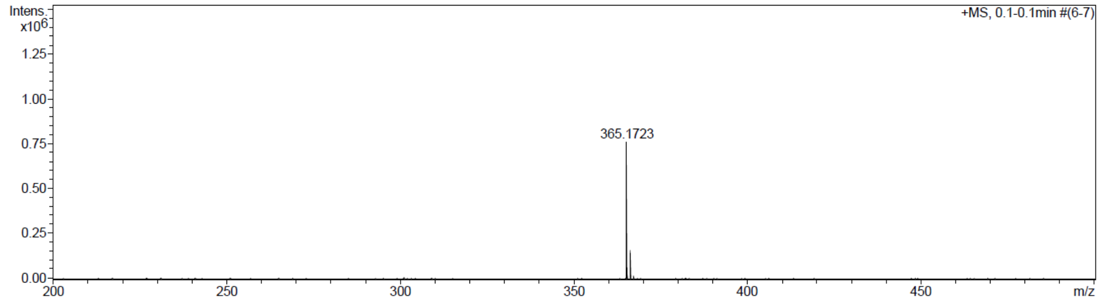

Meas. m/z	#	Formula	Score	m/z	err [mDa]	err [ppm]	mSigma	rdb	e⁻ Conf	N-Rule
365.1723	1	C 21 H 26 Na O 4	100.00	365.1723	0.0	0.1	14.0	8.5	even	ok

Supplementary Figure 174. HRMS chromatogram for compound **2e**

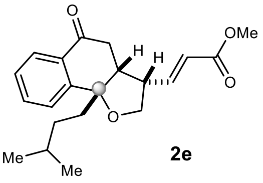

2e

Acquisition Parameter

Source Type	ESI	Ion Polarity	Positive	Set Nebulizer	0.3 Bar
Focus	Not active	Set Capillary	4500 V	Set Dry Heater	180 °C
Scan Begin	100 m/z	Set End Plate Offset	-500 V	Set Dry Gas	4.0 l/min
Scan End	1200 m/z	Set Collision Cell RF	1500.0 Vpp	Set Divert Valve	Source

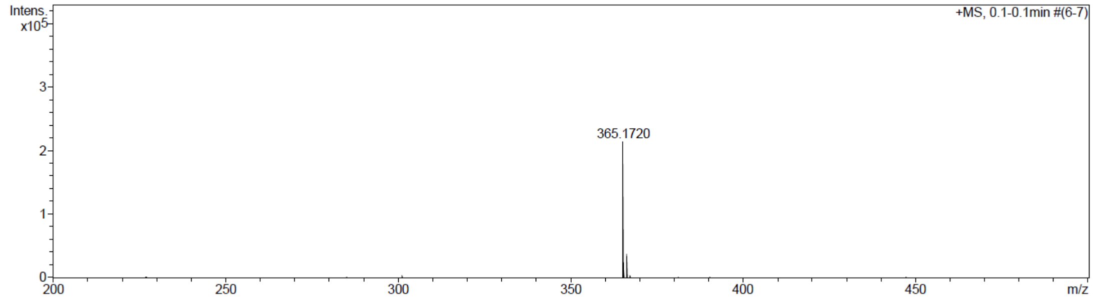

Meas. m/z	#	Formula	Score	m/z	err [mDa]	err [ppm]	mSigma	rdb	e⁻ Conf	N-Rule
365.1720	1	C 21 H 26 Na O 4	100.00	365.1723	0.4	1.0	30.1	8.5	even	ok

Supplementary Figure 175. HRMS chromatogram for compound **1f***

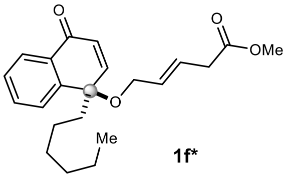

1f*

Acquisition Parameter

Source Type	ESI	Ion Polarity	Positive	Set Nebulizer	0.3 Bar
Focus	Not active	Set Capillary	4500 V	Set Dry Heater	180 °C
Scan Begin	100 m/z	Set End Plate Offset	-500 V	Set Dry Gas	4.0 l/min
Scan End	1200 m/z	Set Collision Cell RF	1500.0 Vpp	Set Divert Valve	Source

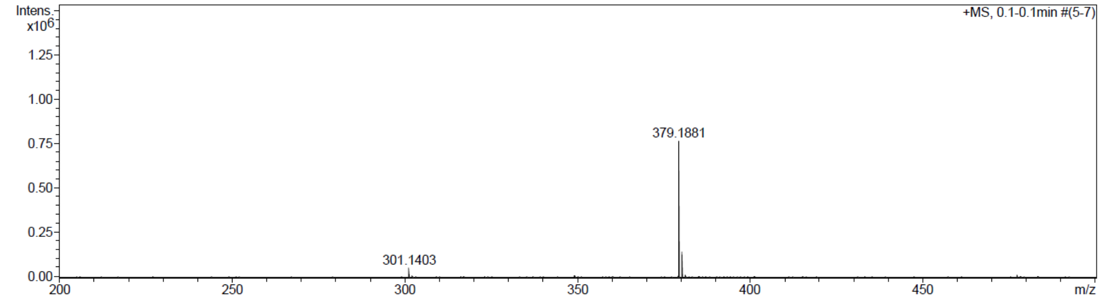

Meas. m/z	#	Formula	Score	m/z	err [mDa]	err [ppm]	mSigma	rdb	e⁻ Conf	N-Rule
379.1881	1	C 22 H 28 Na O 4	100.00	379.1880	-0.1	-0.4	33.6	8.5	even	ok

Supplementary Figure 176. HRMS chromatogram for compound **2f**

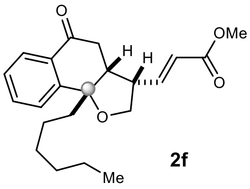

2f

Acquisition Parameter

Source Type	ESI	Ion Polarity	Positive	Set Nebulizer	0.3 Bar
Focus	Not active	Set Capillary	4500 V	Set Dry Heater	180 °C
Scan Begin	100 m/z	Set End Plate Offset	-500 V	Set Dry Gas	4.0 l/min
Scan End	1200 m/z	Set Collision Cell RF	1500.0 Vpp	Set Divert Valve	Source

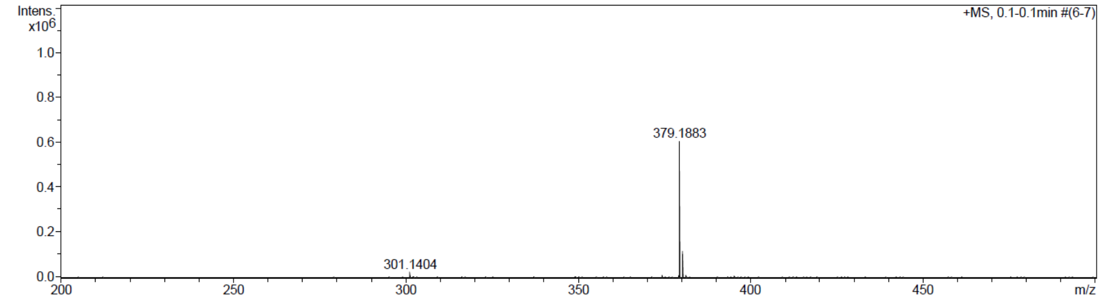

Meas. m/z	#	Formula	Score	m/z	err [mDa]	err [ppm]	mSigma	rdb	e⁻ Conf	N-Rule
379.1883	1	C 22 H 28 Na O 4	100.00	379.1880	-0.3	-0.7	30.3	8.5	even	ok

Supplementary Figure 177. HRMS chromatogram for compound **1g***

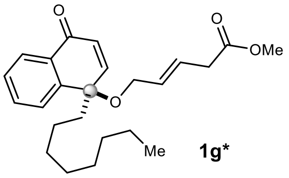

1g*

Acquisition Parameter

Source Type	ESI	Ion Polarity	Positive	Set Nebulizer	0.3 Bar
Focus	Not active	Set Capillary	4500 V	Set Dry Heater	180 °C
Scan Begin	100 m/z	Set End Plate Offset	-500 V	Set Dry Gas	4.0 l/min
Scan End	1200 m/z	Set Collision Cell RF	1500.0 Vpp	Set Divert Valve	Source

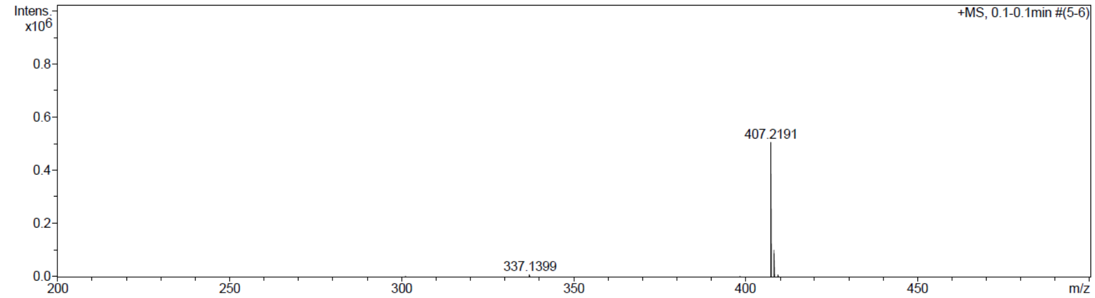

Meas. m/z	#	Formula	Score	m/z	err [mDa]	err [ppm]	mSigma	rdb	e⁻ Conf	N-Rule
407.2191	1	C 24 H 32 Na O 4	100.00	407.2193	0.2	0.4	39.2	8.5	even	ok

Supplementary Figure 178. HRMS chromatogram for compound **2g**

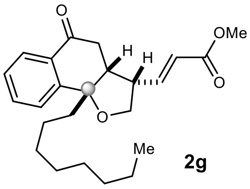

2g

Acquisition Parameter

Source Type	ESI	Ion Polarity	Positive	Set Nebulizer	0.3 Bar
Focus	Not active	Set Capillary	4500 V	Set Dry Heater	180 °C
Scan Begin	100 m/z	Set End Plate Offset	-500 V	Set Dry Gas	4.0 l/min
Scan End	1200 m/z	Set Collision Cell RF	1500.0 Vpp	Set Divert Valve	Source

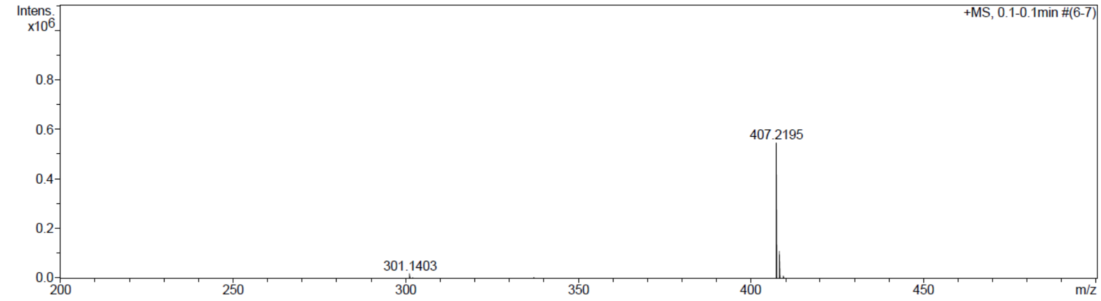

Meas. m/z	#	Formula	Score	m/z	err [mDa]	err [ppm]	mSigma	rdb	e⁻ Conf	N-Rule
407.2195	1	C 24 H 32 Na O 4	100.00	407.2193	-0.3	-0.6	39.7	8.5	even	ok

Supplementary Figure 179. HRMS chromatogram for compound **1h***

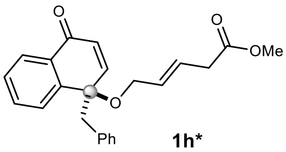

1h*

Acquisition Parameter

Source Type	ESI	Ion Polarity	Positive	Set Nebulizer	0.3 Bar
Focus	Not active	Set Capillary	4500 V	Set Dry Heater	180 °C
Scan Begin	100 m/z	Set End Plate Offset	-500 V	Set Dry Gas	4.0 l/min
Scan End	1200 m/z	Set Collision Cell RF	1500.0 Vpp	Set Divert Valve	Source

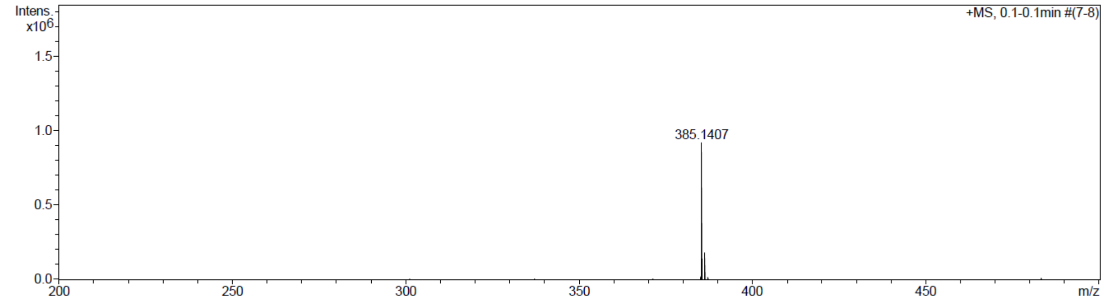

Meas. m/z	#	Formula	Score	m/z	err [mDa]	err [ppm]	mSigma	rdb	e⁻ Conf	N-Rule
385.1407	1	C 23 H 22 Na O 4	100.00	385.1410	0.3	0.7	29.7	12.5	even	ok

Supplementary Figure 180. HRMS chromatogram for compound **2h**

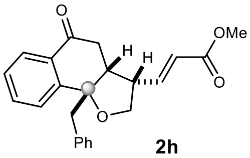

2h

Acquisition Parameter

Source Type	ESI	Ion Polarity	Positive	Set Nebulizer	0.3 Bar
Focus	Not active	Set Capillary	4500 V	Set Dry Heater	180 °C
Scan Begin	100 m/z	Set End Plate Offset	-500 V	Set Dry Gas	4.0 l/min
Scan End	1200 m/z	Set Collision Cell RF	1500.0 Vpp	Set Divert Valve	Source

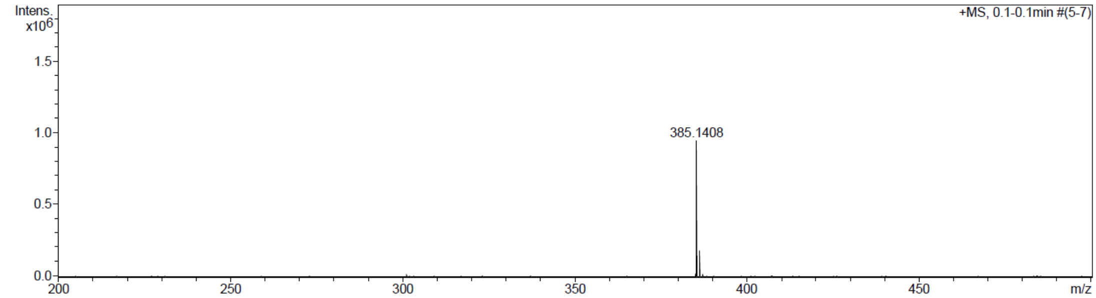

Meas. m/z	#	Formula	Score	m/z	err [mDa]	err [ppm]	mSigma	rdb	e⁻ Conf	N-Rule
385.1408	1	C 23 H 22 Na O 4	100.00	385.1410	0.2	0.6	37.1	12.5	even	ok

Supplementary Figure 181. HRMS chromatogram for compound **1i***

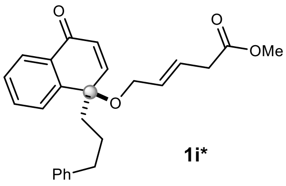

1i*

Acquisition Parameter

Source Type	ESI	Ion Polarity	Positive	Set Nebulizer	0.3 Bar
Focus	Not active	Set Capillary	4500 V	Set Dry Heater	180 °C
Scan Begin	100 m/z	Set End Plate Offset	-500 V	Set Dry Gas	4.0 l/min
Scan End	1200 m/z	Set Collision Cell RF	1500.0 Vpp	Set Divert Valve	Source

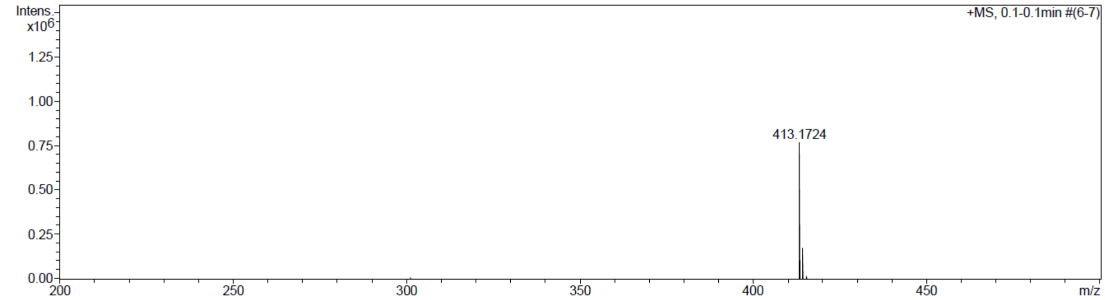

Meas. m/z	#	Formula	Score	m/z	err [mDa]	err [ppm]	mSigma	rdb	e⁻ Conf	N-Rule
413.1724	1	C 25 H 26 Na O 4	100.00	413.1723	-0.0	-0.1	27.0	12.5	even	ok

Supplementary Figure 182. HRMS chromatogram for compound **2i**

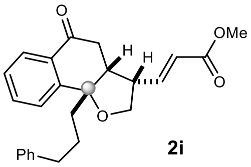

2i

Acquisition Parameter

Source Type	ESI	Ion Polarity	Positive	Set Nebulizer	0.3 Bar
Focus	Not active	Set Capillary	4500 V	Set Dry Heater	180 °C
Scan Begin	100 m/z	Set End Plate Offset	-500 V	Set Dry Gas	4.0 l/min
Scan End	1200 m/z	Set Collision Cell RF	1500.0 Vpp	Set Divert Valve	Source

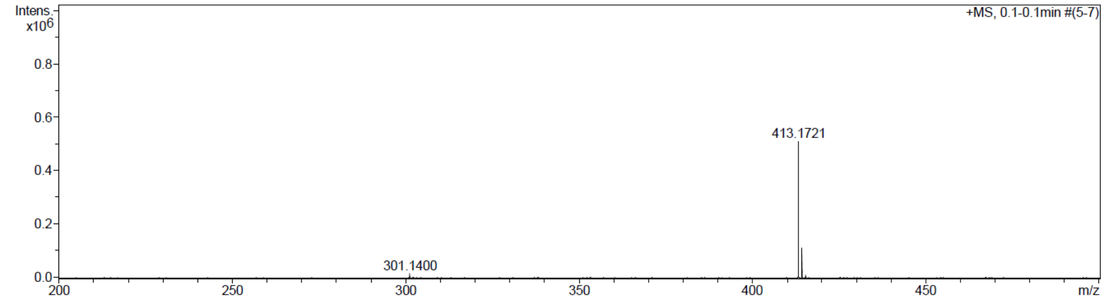

Meas. m/z	#	Formula	Score	m/z	err [mDa]	err [ppm]	mSigma	rdb	e⁻ Conf	N-Rule
413.1721	1	C 25 H 26 Na O 4	100.00	413.1723	0.2	0.5	31.9	12.5	even	ok

Supplementary Figure 183. HRMS chromatogram for compound **1j***

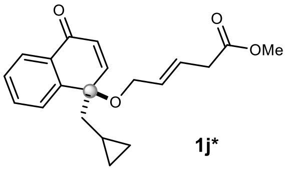

1j*

Acquisition Parameter

Source Type	ESI	Ion Polarity	Positive	Set Nebulizer	0.3 Bar
Focus	Not active	Set Capillary	4500 V	Set Dry Heater	180 °C
Scan Begin	100 m/z	Set End Plate Offset	-500 V	Set Dry Gas	4.0 l/min
Scan End	1200 m/z	Set Collision Cell RF	1500.0 Vpp	Set Divert Valve	Source

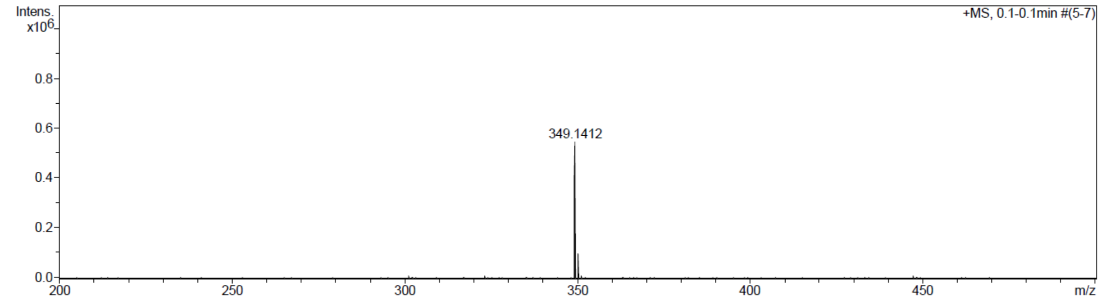

Meas. m/z	#	Formula	Score	m/z	err [mDa]	err [ppm]	mSigma	rdb	e⁻ Conf	N-Rule
349.1412	1	C 20 H 22 Na O 4	100.00	349.1410	-0.2	-0.5	25.3	9.5	even	ok

Supplementary Figure 184. HRMS chromatogram for compound **2j**

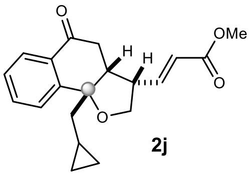

2j

Acquisition Parameter

Source Type	ESI	Ion Polarity	Positive	Set Nebulizer	0.3 Bar
Focus	Not active	Set Capillary	4500 V	Set Dry Heater	180 °C
Scan Begin	100 m/z	Set End Plate Offset	-500 V	Set Dry Gas	4.0 l/min
Scan End	1200 m/z	Set Collision Cell RF	1500.0 Vpp	Set Divert Valve	Source

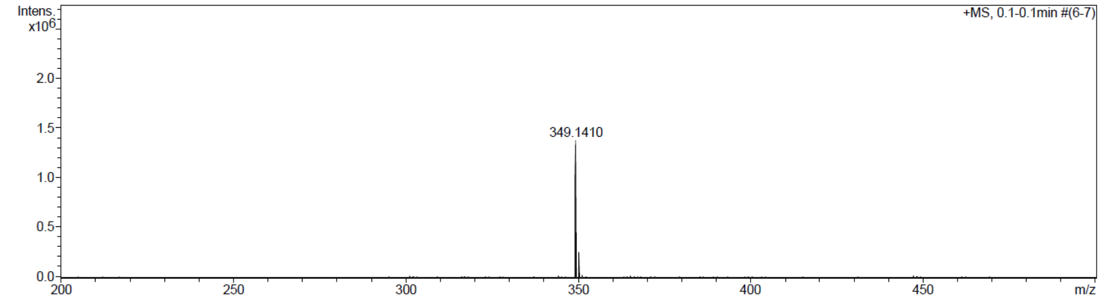

Meas. m/z	#	Formula	Score	m/z	err [mDa]	err [ppm]	mSigma	rdb	e⁻ Conf	N-Rule
349.1410	1	C 20 H 22 Na O 4	100.00	349.1410	0.0	0.0	20.8	9.5	even	ok

Supplementary Figure 185. HRMS chromatogram for compound **1k***

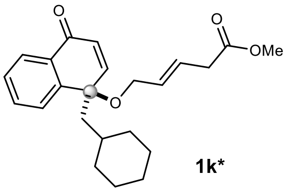

1k*

Acquisition Parameter

Source Type	ESI	Ion Polarity	Positive	Set Nebulizer	0.3 Bar
Focus	Not active	Set Capillary	4500 V	Set Dry Heater	180 °C
Scan Begin	100 m/z	Set End Plate Offset	-500 V	Set Dry Gas	4.0 l/min
Scan End	1200 m/z	Set Collision Cell RF	1500.0 Vpp	Set Divert Valve	Source

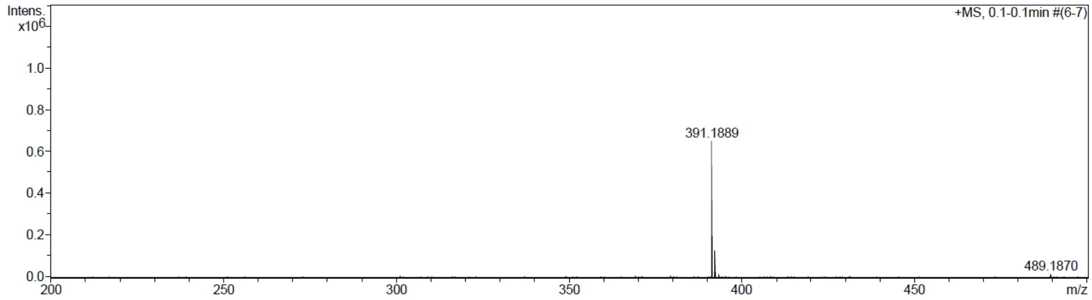

Meas. m/z	#	Formula	Score	m/z	err [mDa]	err [ppm]	mSigma	rdb	e⁻ Conf	N-Rule
391.1889	1	C 23 H 28 Na O 4	100.00	391.1880	-0.9	-2.2	31.5	9.5	even	ok

Supplementary Figure 186. HRMS chromatogram for compound **2k**

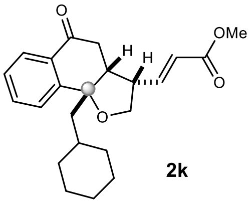

2k

Acquisition Parameter

Source Type	ESI	Ion Polarity	Positive	Set Nebulizer	0.3 Bar
Focus	Not active	Set Capillary	4500 V	Set Dry Heater	180 °C
Scan Begin	100 m/z	Set End Plate Offset	-500 V	Set Dry Gas	4.0 l/min
Scan End	1200 m/z	Set Collision Cell RF	1500.0 Vpp	Set Divert Valve	Source

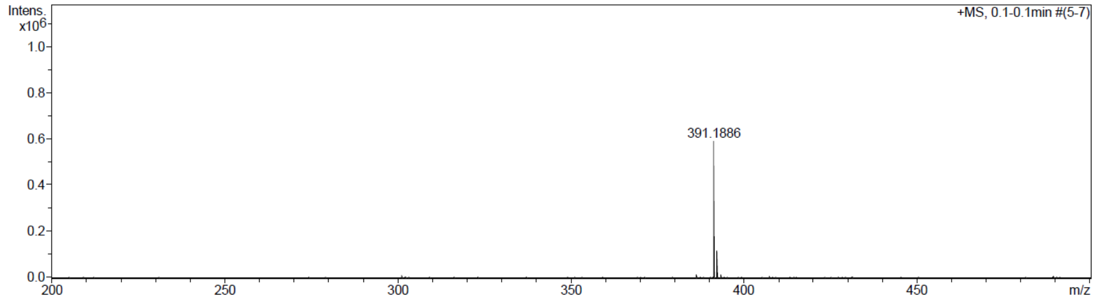

Meas. m/z	#	Formula	Score	m/z	err [mDa]	err [ppm]	mSigma	rdb	e⁻ Conf	N-Rule
391.1886	1	C 23 H 28 Na O 4	100.00	391.1880	-0.7	-1.7	31.4	9.5	even	ok

Supplementary Figure 187. HRMS chromatogram for compound **1l***

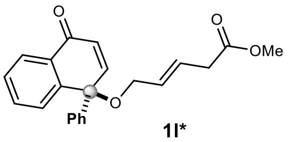

1l*

Acquisition Parameter

Source Type	ESI	Ion Polarity	Positive	Set Nebulizer	0.3 Bar
Focus	Not active	Set Capillary	4500 V	Set Dry Heater	180 °C
Scan Begin	100 m/z	Set End Plate Offset	-500 V	Set Dry Gas	4.0 l/min
Scan End	1200 m/z	Set Collision Cell RF	1500.0 Vpp	Set Divert Valve	Source

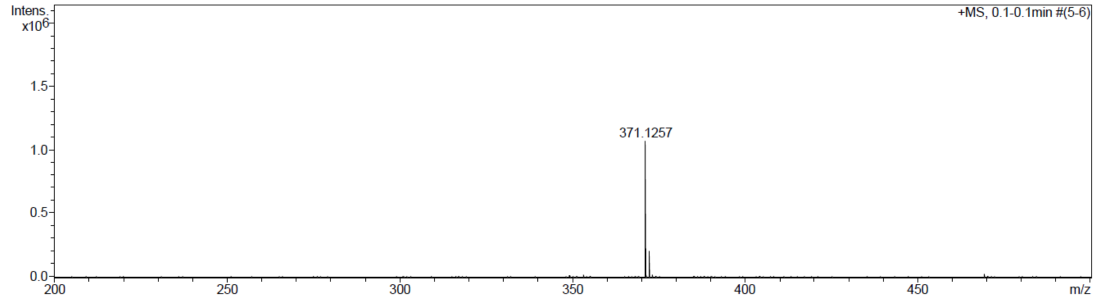

Meas. m/z	#	Formula	Score	m/z	err [mDa]	err [ppm]	mSigma	rdb	e⁻ Conf	N-Rule
371.1257	1	C 22 H 20 Na O 4	100.00	371.1254	-0.3	-0.7	31.6	12.5	even	ok

Supplementary Figure 188. HRMS chromatogram for compound **2l**

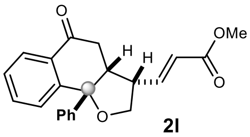

2l

Acquisition Parameter

Source Type	ESI	Ion Polarity	Positive	Set Nebulizer	0.3 Bar
Focus	Not active	Set Capillary	4500 V	Set Dry Heater	180 °C
Scan Begin	100 m/z	Set End Plate Offset	-500 V	Set Dry Gas	4.0 l/min
Scan End	1200 m/z	Set Collision Cell RF	1500.0 Vpp	Set Divert Valve	Source

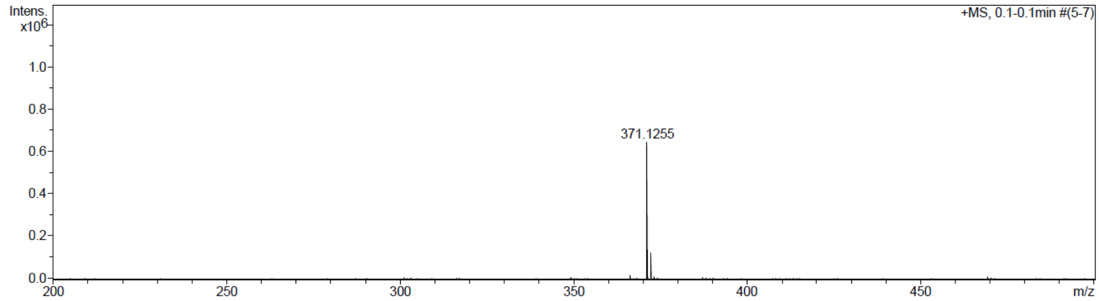

Meas. m/z	#	Formula	Score	m/z	err [mDa]	err [ppm]	mSigma	rdb	e⁻ Conf	N-Rule
371.1255	1	C 22 H 20 Na O 4	100.00	371.1254	-0.1	-0.4	29.6	12.5	even	ok

Supplementary Figure 189. HRMS chromatogram for compound **1m***

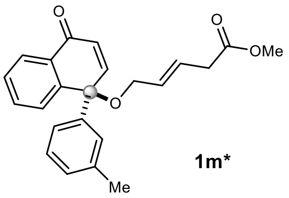

1m*

Acquisition Parameter

Source Type	ESI	Ion Polarity	Positive	Set Nebulizer	0.3 Bar
Focus	Not active	Set Capillary	4500 V	Set Dry Heater	180 °C
Scan Begin	100 m/z	Set End Plate Offset	-500 V	Set Dry Gas	4.0 l/min
Scan End	1200 m/z	Set Collision Cell RF	1500.0 Vpp	Set Divert Valve	Source

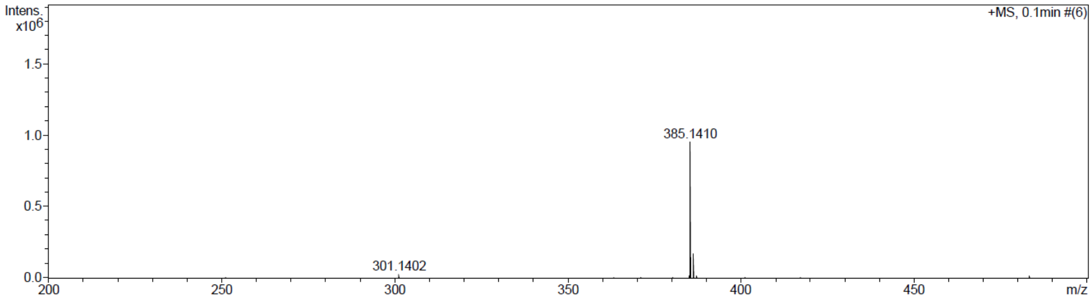

Meas. m/z	#	Formula	Score	m/z	err [mDa]	err [ppm]	mSigma	rdb	e⁻ Conf	N-Rule
385.1410	1	C 23 H 22 Na O 4	100.00	385.1410	-0.0	-0.0	39.2	12.5	even	ok

Supplementary Figure 190. HRMS chromatogram for compound **2m**

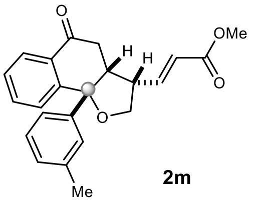

2m

Acquisition Parameter

Source Type	ESI	Ion Polarity	Positive	Set Nebulizer	0.3 Bar
Focus	Not active	Set Capillary	4500 V	Set Dry Heater	180 °C
Scan Begin	100 m/z	Set End Plate Offset	-500 V	Set Dry Gas	4.0 l/min
Scan End	1200 m/z	Set Collision Cell RF	1500.0 Vpp	Set Divert Valve	Source

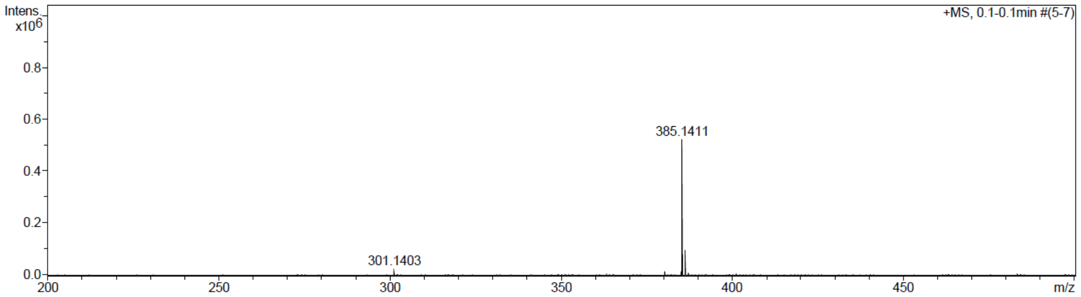

Meas. m/z	#	Formula	Score	m/z	err [mDa]	err [ppm]	mSigma	rdb	e⁻ Conf	N-Rule
385.1411	1	C 23 H 22 Na O 4	100.00	385.1410	-0.0	-0.1	40.0	12.5	even	ok

Supplementary Figure 191. HRMS chromatogram for compound **1n***

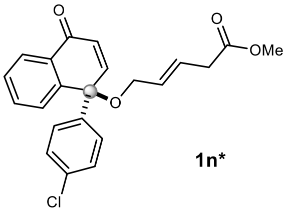

1n*

Acquisition Parameter

Source Type	ESI	Ion Polarity	Positive	Set Nebulizer	0.3 Bar
Focus	Not active	Set Capillary	4500 V	Set Dry Heater	180 °C
Scan Begin	100 m/z	Set End Plate Offset	-500 V	Set Dry Gas	4.0 l/min
Scan End	1200 m/z	Set Collision Cell RF	1500.0 Vpp	Set Divert Valve	Source

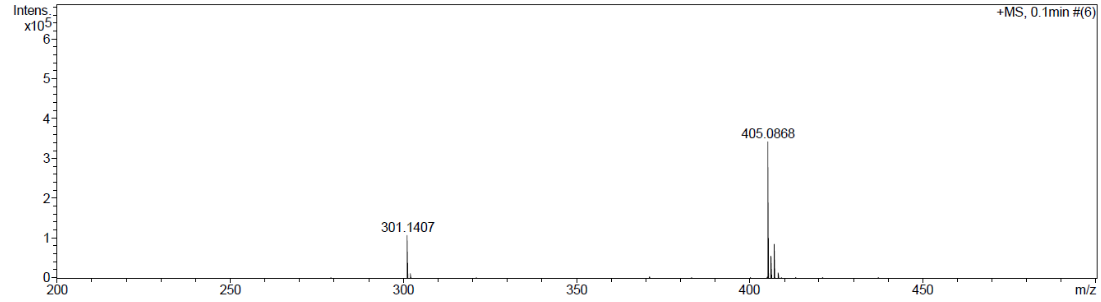

Meas. m/z	#	Formula	Score	m/z	err [mDa]	err [ppm]	mSigma	rdb	e⁻ Conf	N-Rule
405.0868	1	C 22 H 19 Cl Na O 4	100.00	405.0864	-0.4	-1.1	57.8	12.5	even	ok

Supplementary Figure 192. HRMS chromatogram for compound **2n**

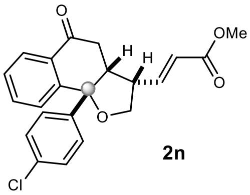

2n

Acquisition Parameter

Source Type	ESI	Ion Polarity	Positive	Set Nebulizer	0.3 Bar
Focus	Not active	Set Capillary	4500 V	Set Dry Heater	180 °C
Scan Begin	100 m/z	Set End Plate Offset	-500 V	Set Dry Gas	4.0 l/min
Scan End	1200 m/z	Set Collision Cell RF	1500.0 Vpp	Set Divert Valve	Source

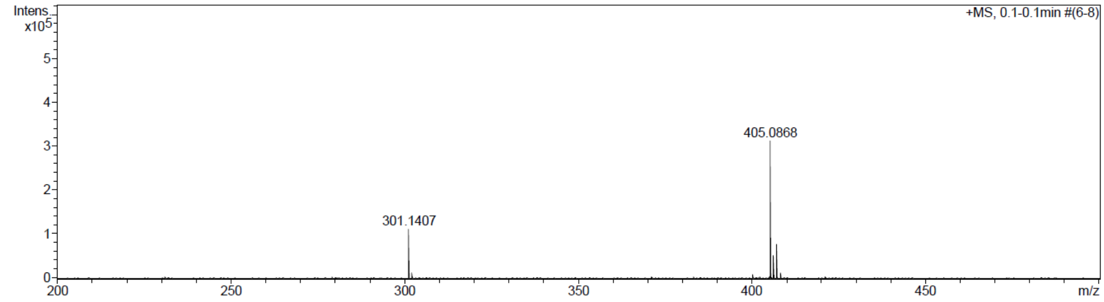

Meas. m/z	#	Formula	Score	m/z	err [mDa]	err [ppm]	mSigma	rdb	e⁻ Conf	N-Rule
405.0868	1	C 22 H 19 Cl Na O 4	100.00	405.0864	-0.4	-1.0	60.3	12.5	even	ok

Supplementary Figure 193. HRMS chromatogram for compound **1o***

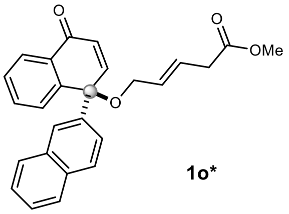

1o*

Acquisition Parameter

Source Type	ESI	Ion Polarity	Positive	Set Nebulizer	0.3 Bar
Focus	Not active	Set Capillary	4500 V	Set Dry Heater	180 °C
Scan Begin	100 m/z	Set End Plate Offset	-500 V	Set Dry Gas	4.0 l/min
Scan End	1200 m/z	Set Collision Cell RF	1500.0 Vpp	Set Divert Valve	Source

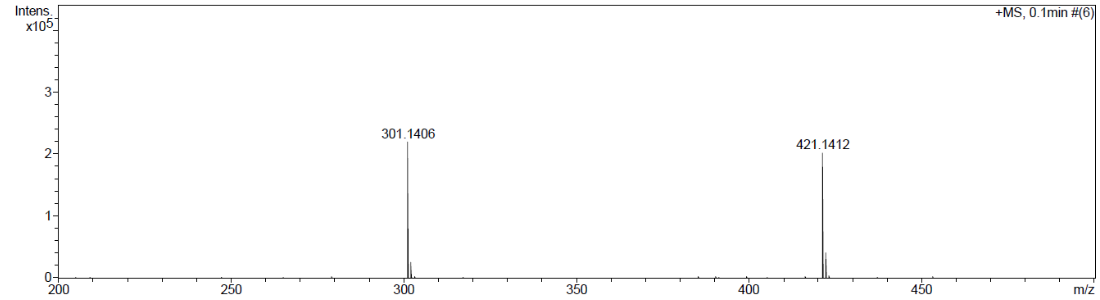

Meas. m/z	#	Formula	Score	m/z	err [mDa]	err [ppm]	mSigma	rdb	e⁻ Conf	N-Rule
421.1412	1	C 26 H 22 Na O 4	100.00	421.1410	-0.2	-0.5	48.0	15.5	even	ok

Supplementary Figure 194. HRMS chromatogram for compound **2o**

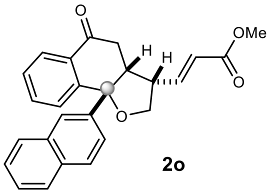

2o

Acquisition Parameter

Source Type	ESI	Ion Polarity	Positive	Set Nebulizer	0.3 Bar
Focus	Not active	Set Capillary	4500 V	Set Dry Heater	180 °C
Scan Begin	100 m/z	Set End Plate Offset	-500 V	Set Dry Gas	4.0 l/min
Scan End	1200 m/z	Set Collision Cell RF	1500.0 Vpp	Set Divert Valve	Source

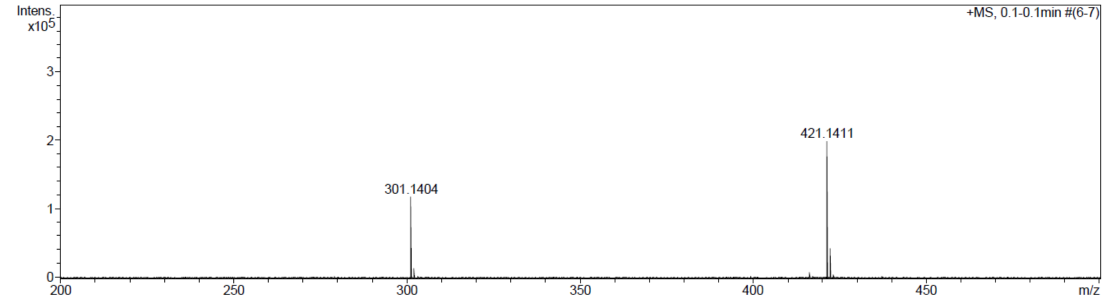

Meas. m/z	#	Formula	Score	m/z	err [mDa]	err [ppm]	mSigma	rdb	e⁻ Conf	N-Rule
421.1411	1	C 26 H 22 Na O 4	100.00	421.1410	-0.1	-0.2	43.5	15.5	even	ok

Supplementary Figure 195. HRMS chromatogram for compound **1p***

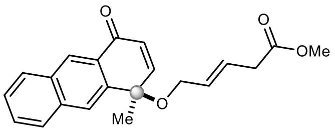

1p*

Acquisition Parameter

Source Type	ESI	Ion Polarity	Positive	Set Nebulizer	0.3 Bar
Focus	Not active	Set Capillary	4500 V	Set Dry Heater	180 °C
Scan Begin	100 m/z	Set End Plate Offset	-500 V	Set Dry Gas	4.0 l/min
Scan End	1200 m/z	Set Collision Cell RF	1500.0 Vpp	Set Divert Valve	Source

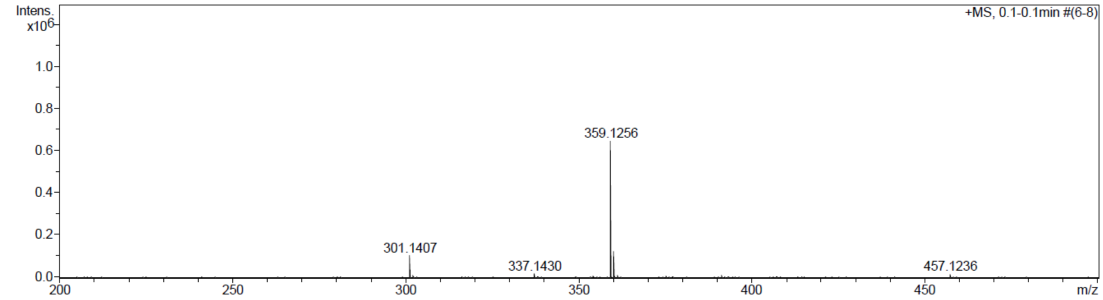

Meas. m/z	#	Formula	Score	m/z	err [mDa]	err [ppm]	mSigma	rdb	e⁻ Conf	N-Rule
359.1256	1	C 21 H 20 Na O 4	100.00	359.1254	-0.2	-0.6	23.0	11.5	even	ok

Supplementary Figure 196. HRMS chromatogram for compound **2p**

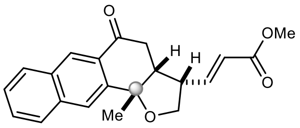

2p

Acquisition Parameter

Source Type	ESI	Ion Polarity	Positive	Set Nebulizer	0.3 Bar
Focus	Not active	Set Capillary	4500 V	Set Dry Heater	180 °C
Scan Begin	100 m/z	Set End Plate Offset	-500 V	Set Dry Gas	4.0 l/min
Scan End	1200 m/z	Set Collision Cell RF	1500.0 Vpp	Set Divert Valve	Source

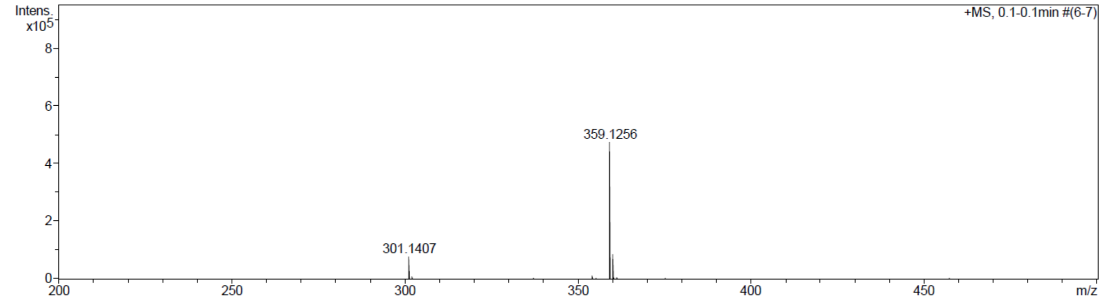

Meas. m/z	#	Formula	Score	m/z	err [mDa]	err [ppm]	mSigma	rdb	e⁻ Conf	N-Rule
359.1256	1	C 21 H 20 Na O 4	100.00	359.1254	-0.2	-0.6	29.1	11.5	even	ok

Supplementary Figure 197. HRMS chromatogram for compound **1q***

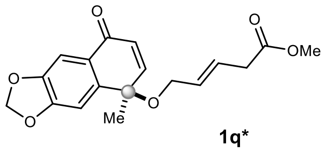

1q*

Acquisition Parameter

Source Type	ESI	Ion Polarity	Positive	Set Nebulizer	0.3 Bar
Focus	Not active	Set Capillary	4500 V	Set Dry Heater	180 °C
Scan Begin	100 m/z	Set End Plate Offset	-500 V	Set Dry Gas	4.0 l/min
Scan End	1200 m/z	Set Collision Cell RF	1500.0 Vpp	Set Divert Valve	Source

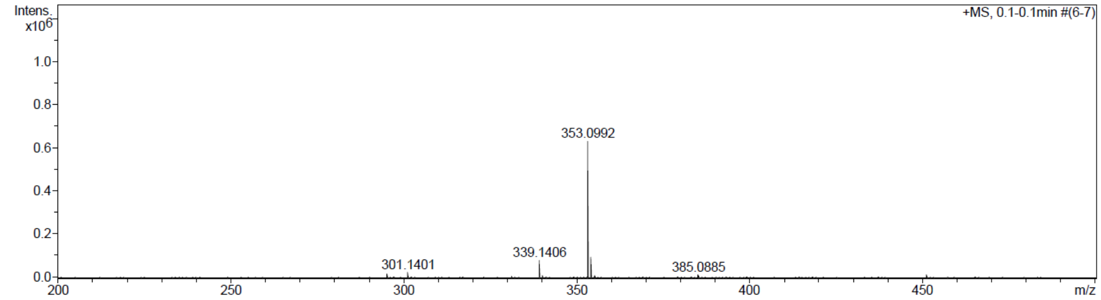

Meas. m/z	#	Formula	Score	m/z	err [mDa]	err [ppm]	mSigma	rdb	e⁻ Conf	N-Rule
353.0992	1	C 18 H 18 Na O 6	100.00	353.0996	0.3	1.0	29.5	9.5	even	ok

Supplementary Figure 198. HRMS chromatogram for compound **2q**

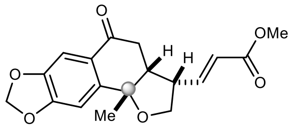

2q

Acquisition Parameter

Source Type	ESI	Ion Polarity	Positive	Set Nebulizer	0.3 Bar
Focus	Not active	Set Capillary	4500 V	Set Dry Heater	180 °C
Scan Begin	100 m/z	Set End Plate Offset	-500 V	Set Dry Gas	4.0 l/min
Scan End	1200 m/z	Set Collision Cell RF	1500.0 Vpp	Set Divert Valve	Source

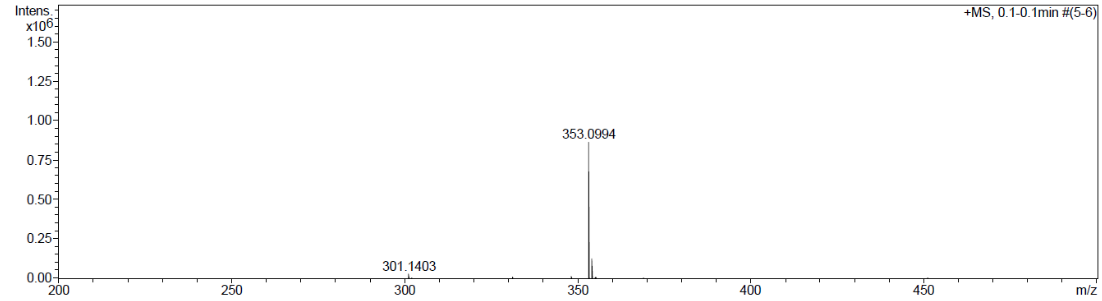

Meas. m/z	#	Formula	Score	m/z	err [mDa]	err [ppm]	mSigma	rdb	e⁻ Conf	N-Rule
353.0994	1	C 18 H 18 Na O 6	100.00	353.0996	0.1	0.4	30.3	9.5	even	ok

Supplementary Figure 199. HRMS chromatogram for compound **1r***

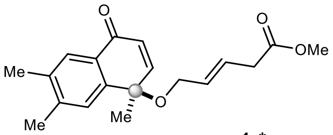

1r*

Acquisition Parameter

Source Type	ESI	Ion Polarity	Positive	Set Nebulizer	0.3 Bar
Focus	Not active	Set Capillary	4500 V	Set Dry Heater	180 °C
Scan Begin	100 m/z	Set End Plate Offset	-500 V	Set Dry Gas	4.0 l/min
Scan End	1200 m/z	Set Collision Cell RF	1500.0 Vpp	Set Divert Valve	Source

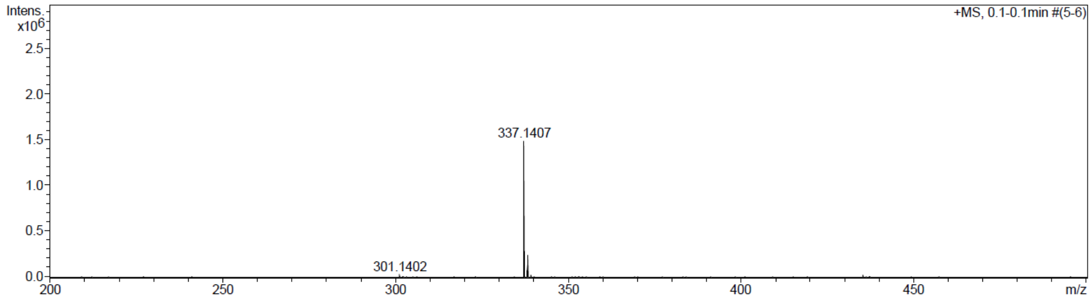

Meas. m/z	#	Formula	Score	m/z	err [mDa]	err [ppm]	mSigma	rdb	e¯ Conf	N-Rule
337.1407	1	C 19 H 22 Na O 4	100.00	337.1410	0.3	0.9	28.5	8.5	even	ok

Supplementary Figure 200. HRMS chromatogram for compound **2r**

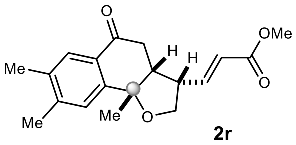

2r

Acquisition Parameter

Source Type	ESI	Ion Polarity	Positive	Set Nebulizer	0.3 Bar
Focus	Not active	Set Capillary	4500 V	Set Dry Heater	180 °C
Scan Begin	100 m/z	Set End Plate Offset	-500 V	Set Dry Gas	4.0 l/min
Scan End	1200 m/z	Set Collision Cell RF	1500.0 Vpp	Set Divert Valve	Source

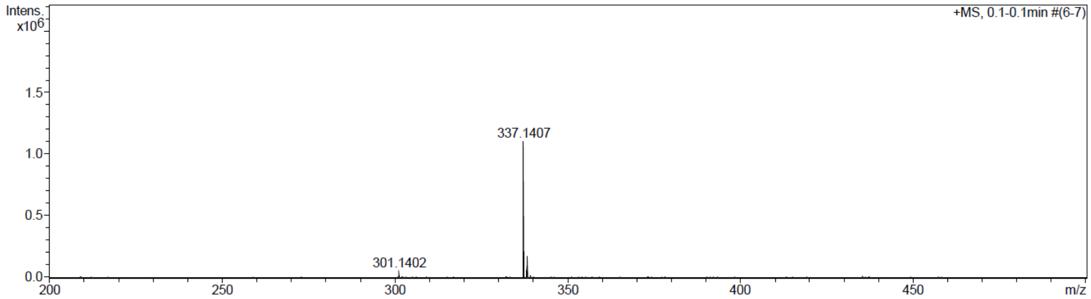

Meas. m/z	#	Formula	Score	m/z	err [mDa]	err [ppm]	mSigma	rdb	e¯ Conf	N-Rule
337.1407	1	C 19 H 22 Na O 4	100.00	337.1410	0.4	1.1	29.8	8.5	even	ok

Supplementary Figure 201. HRMS chromatogram for compound **1s***

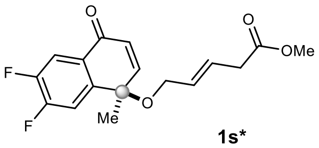

1s*

Acquisition Parameter

Source Type	ESI	Ion Polarity	Positive	Set Nebulizer	0.3 Bar
Focus	Not active	Set Capillary	4500 V	Set Dry Heater	180 °C
Scan Begin	100 m/z	Set End Plate Offset	-500 V	Set Dry Gas	4.0 l/min
Scan End	1200 m/z	Set Collision Cell RF	1500.0 Vpp	Set Divert Valve	Source

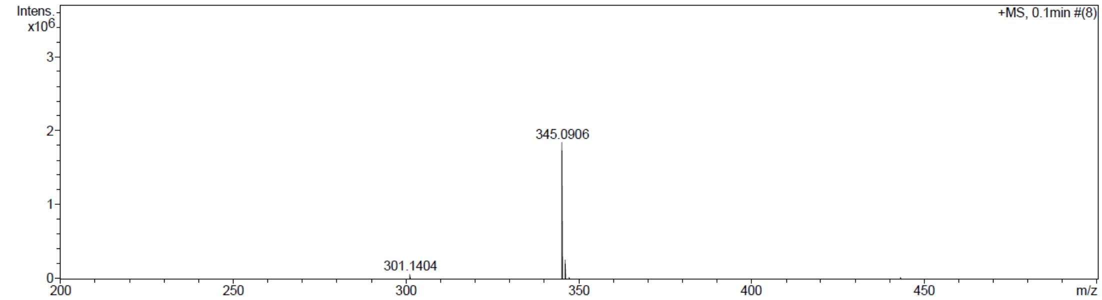

Meas. m/z	#	Formula	Score	m/z	err [mDa]	err [ppm]	mSigma	rdb	e⁻ Conf	N-Rule
345.0906	1	C 17 H 16 F 2 Na O 4	100.00	345.0909	0.2	0.7	25.0	8.5	even	ok

Supplementary Figure 202. HRMS chromatogram for compound **2s**

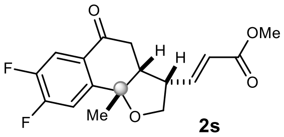

2s

Acquisition Parameter

Source Type	ESI	Ion Polarity	Positive	Set Nebulizer	0.3 Bar
Focus	Not active	Set Capillary	4500 V	Set Dry Heater	180 °C
Scan Begin	100 m/z	Set End Plate Offset	-500 V	Set Dry Gas	4.0 l/min
Scan End	1200 m/z	Set Collision Cell RF	1500.0 Vpp	Set Divert Valve	Source

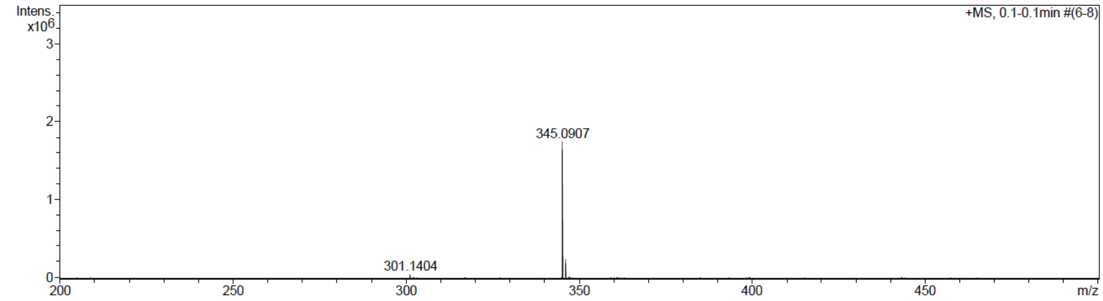

Meas. m/z	#	Formula	Score	m/z	err [mDa]	err [ppm]	mSigma	rdb	e⁻ Conf	N-Rule
345.0907	1	C 17 H 16 F 2 Na O 4	100.00	345.0909	0.2	0.6	27.1	8.5	even	ok

Supplementary Figure 203. HRMS chromatogram for compound **1t***

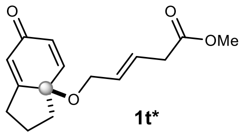

1t*

Acquisition Parameter

Source Type	ESI	Ion Polarity	Positive	Set Nebulizer	0.3 Bar
Focus	Not active	Set Capillary	4500 V	Set Dry Heater	180 °C
Scan Begin	100 m/z	Set End Plate Offset	-500 V	Set Dry Gas	4.0 l/min
Scan End	1200 m/z	Set Collision Cell RF	1500.0 Vpp	Set Divert Valve	Source

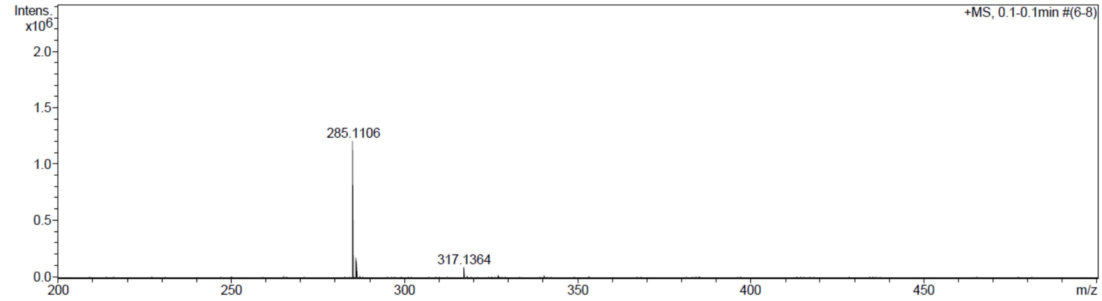

Meas. m/z	#	Formula	Score	m/z	err [mDa]	err [ppm]	mSigma	rdb	e$^-$ Conf	N-Rule
285.1106	1	C 15 H 18 Na O 4	100.00	285.1097	-0.8	-3.0	14.3	6.5	even	ok

Supplementary Figure 204. HRMS chromatogram for compound **2t**

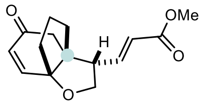

2t

Acquisition Parameter

Source Type	ESI	Ion Polarity	Positive	Set Nebulizer	0.3 Bar
Focus	Not active	Set Capillary	4500 V	Set Dry Heater	180 °C
Scan Begin	100 m/z	Set End Plate Offset	-500 V	Set Dry Gas	4.0 l/min
Scan End	1200 m/z	Set Collision Cell RF	1500.0 Vpp	Set Divert Valve	Source

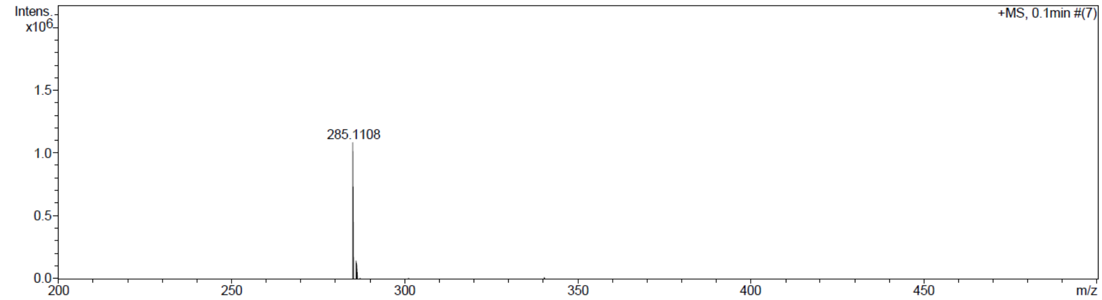

Meas. m/z	#	Formula	Score	m/z	err [mDa]	err [ppm]	mSigma	rdb	e$^-$ Conf	N-Rule
285.1108	1	C 15 H 18 Na O 4	100.00	285.1097	-1.1	-3.7	17.4	6.5	even	ok

Supplementary Figure 205. HRMS chromatogram for compound **2a'**

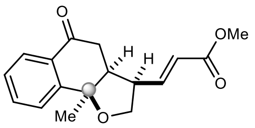

2a'

Acquisition Parameter

Source Type	ESI	Ion Polarity	Positive	Set Nebulizer	0.3 Bar
Focus	Not active	Set Capillary	4500 V	Set Dry Heater	180 °C
Scan Begin	100 m/z	Set End Plate Offset	-500 V	Set Dry Gas	4.0 l/min
Scan End	1200 m/z	Set Collision Cell RF	1500.0 Vpp	Set Divert Valve	Source

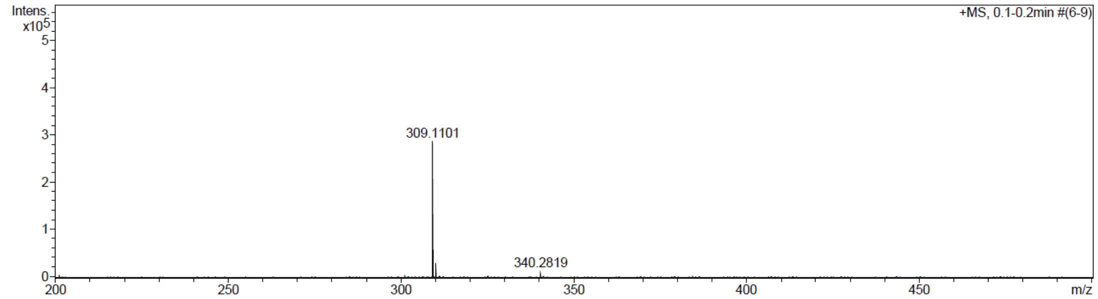

Meas. m/z	#	Formula	Score	m/z	err [mDa]	err [ppm]	mSigma	rdb	e⁻ Conf	N-Rule
309.1101	1	C 17 H 18 Na O 4	100.00	309.1097	-0.3	-1.1	49.7	8.5	even	ok

Supplementary Figure 206. HRMS chromatogram for compound **3**

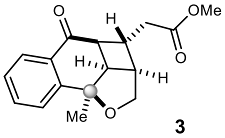

3

Acquisition Parameter

Source Type	ESI	Ion Polarity	Positive	Set Nebulizer	0.3 Bar
Focus	Not active	Set Capillary	4500 V	Set Dry Heater	180 °C
Scan Begin	100 m/z	Set End Plate Offset	-500 V	Set Dry Gas	4.0 l/min
Scan End	1200 m/z	Set Collision Cell RF	1500.0 Vpp	Set Divert Valve	Source

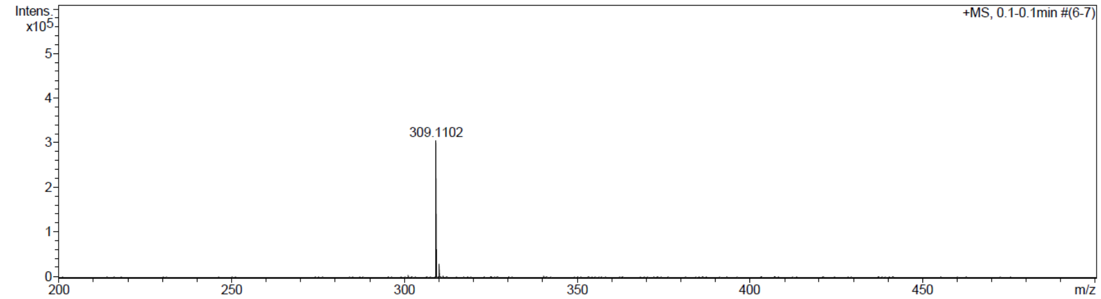

Meas. m/z	#	Formula	Score	m/z	err [mDa]	err [ppm]	mSigma	rdb	e⁻ Conf	N-Rule
309.1102	1	C 17 H 18 Na O 4	100.00	309.1097	-0.5	-1.6	51.5	8.5	even	ok

Supplementary Figure 207. HRMS chromatogram for compound **4**

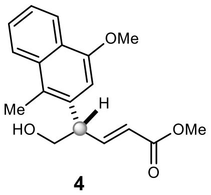

4

Acquisition Parameter

Source Type	ESI	Ion Polarity	Positive	Set Nebulizer	0.3 Bar
Focus	Not active	Set Capillary	4500 V	Set Dry Heater	180 °C
Scan Begin	100 m/z	Set End Plate Offset	-500 V	Set Dry Gas	4.0 l/min
Scan End	1200 m/z	Set Collision Cell RF	1500.0 Vpp	Set Divert Valve	Source

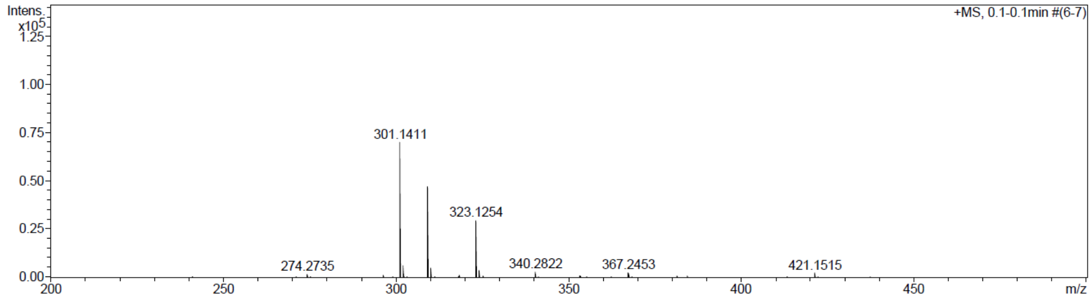

Meas. m/z	#	Formula	Score	m/z	err [mDa]	err [ppm]	mSigma	rdb	e⁻ Conf	N-Rule
323.1254	1	C 18 H 20 Na O 4	100.00	323.1254	-0.0	-0.0	40.7	8.5	even	ok

Supplementary Figure 208. HRMS chromatogram for compound **7**

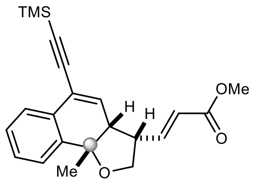

7

Acquisition Parameter

Source Type	ESI	Ion Polarity	Positive	Set Nebulizer	0.3 Bar
Focus	Not active	Set Capillary	4500 V	Set Dry Heater	180 °C
Scan Begin	100 m/z	Set End Plate Offset	-500 V	Set Dry Gas	4.0 l/min
Scan End	1200 m/z	Set Collision Cell RF	1500.0 Vpp	Set Divert Valve	Source

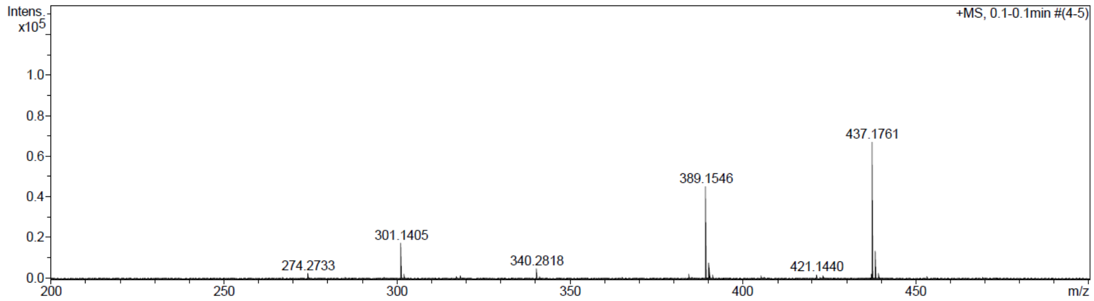

Meas. m/z	#	Formula	Score	m/z	err [mDa]	err [ppm]	mSigma	rdb	e⁻ Conf	N-Rule
389.1546	1	C 22 H 26 Na O 3 Si	100.00	389.1543	-0.3	-0.8	65.4	10.5	even	ok

Supplementary Figure 209. HRMS chromatogram for compound **8**

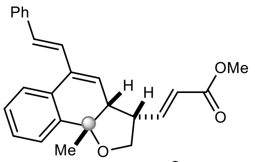

8

Acquisition Parameter

Source Type	ESI	Ion Polarity	Positive	Set Nebulizer	0.3 Bar
Focus	Not active	Set Capillary	4500 V	Set Dry Heater	180 °C
Scan Begin	100 m/z	Set End Plate Offset	-500 V	Set Dry Gas	4.0 l/min
Scan End	1200 m/z	Set Collision Cell RF	1500.0 Vpp	Set Divert Valve	Source

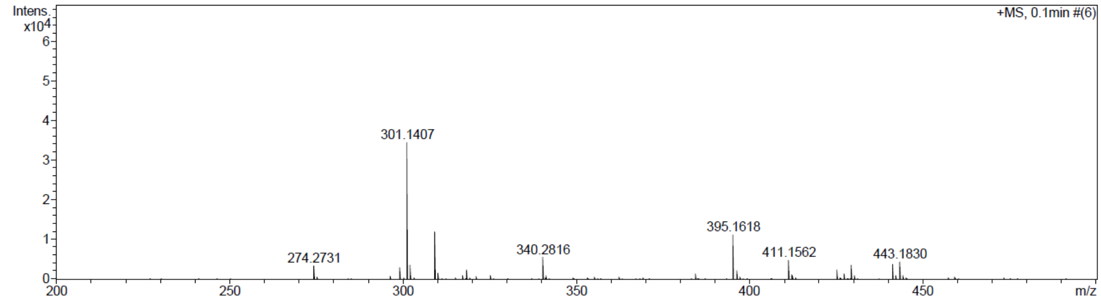

Meas. m/z	#	Formula	Score	m/z	err [mDa]	err [ppm]	mSigma	rdb	e¯ Conf	N-Rule
395.1618	1	C 25 H 24 Na O 3	100.00	395.1618	0.0	0.0	48.2	13.5	even	ok

Supplementary Figure 210. HRMS chromatogram for compound **10**

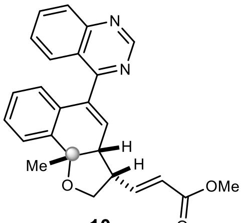

10

Acquisition Parameter

Source Type	ESI	Ion Polarity	Positive	Set Nebulizer	0.3 Bar
Focus	Not active	Set Capillary	4500 V	Set Dry Heater	180 °C
Scan Begin	100 m/z	Set End Plate Offset	-500 V	Set Dry Gas	4.0 l/min
Scan End	1200 m/z	Set Collision Cell RF	1500.0 Vpp	Set Divert Valve	Source

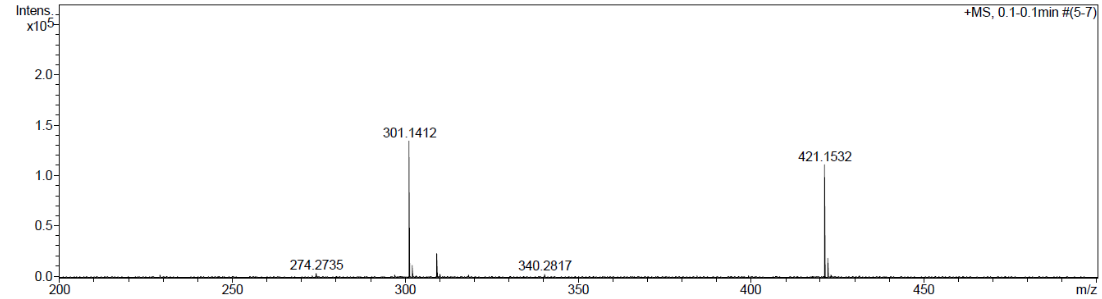

Meas. m/z	#	Formula	Score	m/z	err [mDa]	err [ppm]	mSigma	rdb	e¯ Conf	N-Rule
421.1532	1	C 25 H 22 N 2 Na O 3	100.00	421.1523	-1.0	-2.3	68.6	15.5	even	ok

Supplementary Figure 211. HRMS chromatogram for compound **L10**

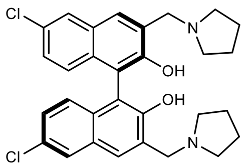

L10

Acquisition Parameter

Source Type	ESI	Ion Polarity	Positive	Set Nebulizer	0.3 Bar
Focus	Not active	Set Capillary	4500 V	Set Dry Heater	180 °C
Scan Begin	100 m/z	Set End Plate Offset	-500 V	Set Dry Gas	4.0 l/min
Scan End	1200 m/z	Set Collision Cell RF	1500.0 Vpp	Set Divert Valve	Source

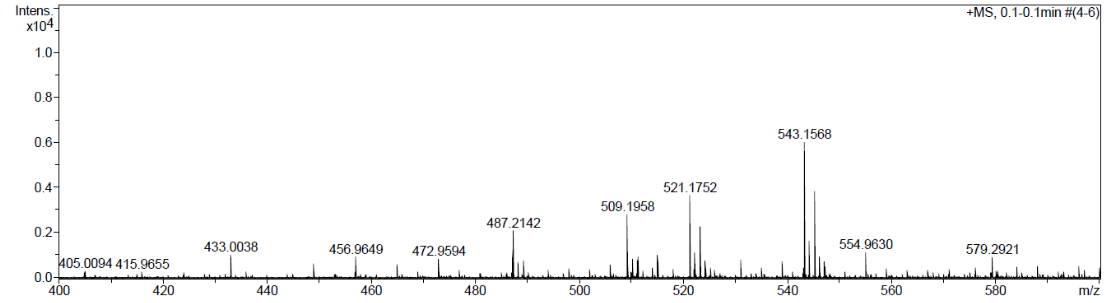

Meas. m/z	#	Formula	Score	m/z	err [mDa]	err [ppm]	mSigma	rdb	e⁻ Conf	N-Rule
543.1568	1	C 30 H 30 Cl 2 N 2 Na O 2	100.00	543.1577	0.9	1.6	46.9	15.5	even	ok
